# *N*-Cyanorhodamines: cell-permeant, photostable and bathochromically shifted analogues of fluoresceins†

**DOI:** 10.1039/d2sc02448a

**Published:** 2022-06-27

**Authors:** Lukas Heynck, Jessica Matthias, Mariano L. Bossi, Alexey N. Butkevich, Stefan W. Hell

**Affiliations:** Department of Optical Nanoscopy, Max Planck Institute for Medical Research 69120 Heidelberg Germany alexey.butkevich@mr.mpg.de; Department of NanoBiophotonics, Max Planck Institute for Multidisciplinary Sciences 37077 Göttingen Germany

## Abstract

Fluorescein and its analogues have found only limited use in biological imaging because of the poor photostability and cell membrane impermeability of their *O*-unprotected forms. Herein, we report rationally designed *N*-cyanorhodamines as orange- to red-emitting, photostable and cell-permeant fluorescent labels negatively charged at physiological pH values and thus devoid of off-targeting artifacts often observed for cationic fluorophores. In combination with well-established fluorescent labels, self-labelling protein (HaloTag, SNAP-tag) ligands derived from *N*-cyanorhodamines permit up to four-colour confocal and super-resolution STED imaging in living cells.

## Abstract 2

*N*-Cyanorhodamines – photostable, cell-permeant analogues of fluoresceins – provide fast labelling kinetics with the HaloTag protein and background-free images in multicolour super-resolution microscopy.

## Graphical abstract

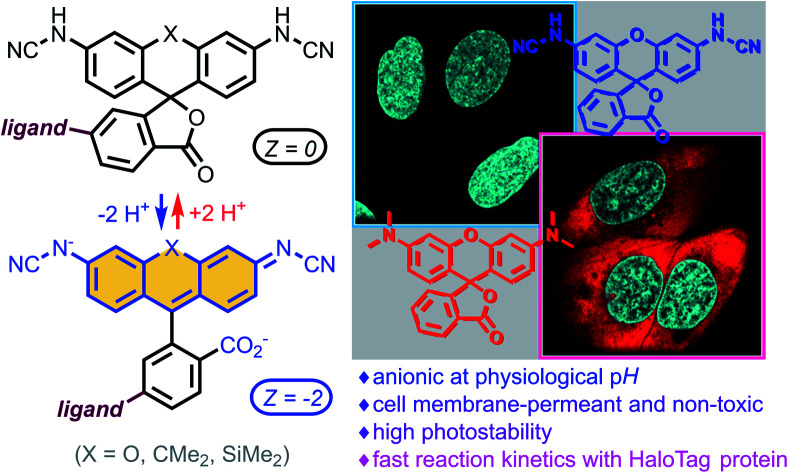

## Introduction

One of the most important advantages of small molecule fluorescent probes over genetically encoded fluorescent proteins is their superior photostability,^1^ which becomes essential under the demanding conditions of super-resolution microscopy (nanoscopy).^2^ Recent advances in fluorescence nanoscopy have prompted the rational design of small molecule fluorescent labels.^3^ Achieving live-cell compatibility with high target specificity,^4^ and the ability to control the net charge of a fluorescent probe,^5^ while maintaining high brightness, chemical stability and low photoreactivity of the fluorophore^6^ remain considerable challenges.

**Fig. 1:**
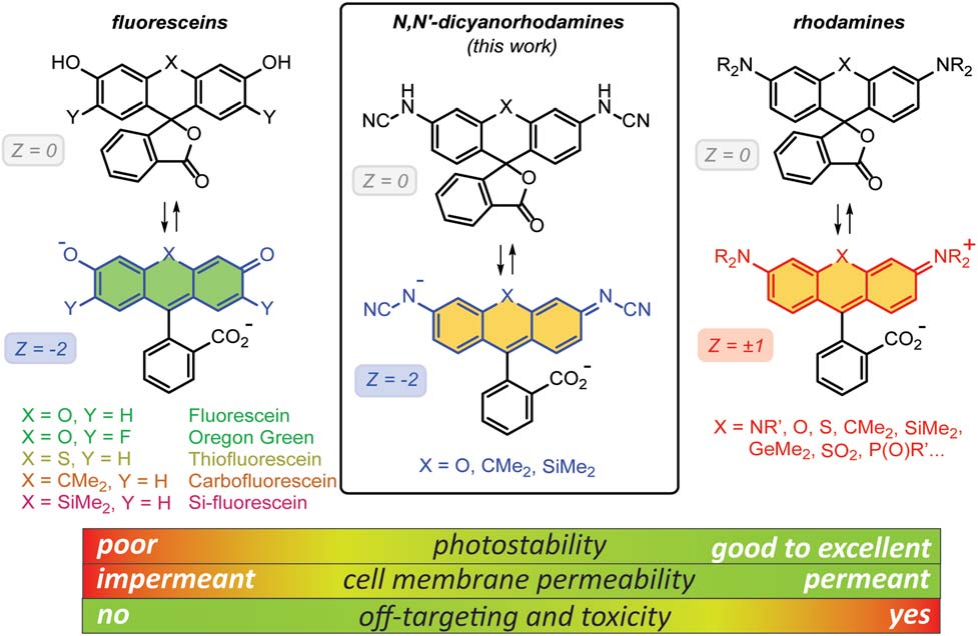
General chemical structures of fluoresceins, rhodamines and newly designed *N*,*N*′-dicyanorhodamines and the rationale behind their use in live-cell imaging.

Fluorescein (resorcinolphthalein) is a green-emitting (*λ*_exc_ = 498 nm, *λ*_em_ = 517 nm, fluorescence quantum yield *Φ* = 0.90 in 0.1 M NaOH)^7^ fluorescent dye from the phthalein family of triarylmethane dyes, first reported by von Baeyer in 1871.^8^ While its p*K*_a_ value of 6.3 makes it brightly fluorescent at cytosolic pH = ∼7.2,^9^ the anionic form does not cross the cell membranes of mammalian and plant cells.^10^ On the contrary, its non-fluorescent *O*-acyl esters,^10^ in particular fluorescein diacetate, enter living cells and are hydrolyzed by cell esterases into free fluorescein. Employing self-labelling protein tags such as HaloTag,^11^ CLIP-tag^12^ and SNAP-tag,^13^ fluorescein ester-derived cell-permeant and fluorogenic live-cell probes targeting specific fusion proteins^14^ have been developed. Fluorescein itself, however, undergoes comparatively rapid photobleaching with a quantum yield of *Φ*_bl_ = 3 × 10^−5^ in water.^15^ Its 2′,7′-difluoro derivative (Oregon Green) is somewhat more photostable and has found wider use in fluorescence microscopy. Besides *O*-acylation, photocleavable^16^ and enzymatically cleavable^17^*O*-protecting groups have been used to render fluorescein and Oregon Green dyes and their derived probes cell-permeant. Replacing the oxygen bridge (X = O) in the xanthene core of fluorescein affords its analogues (thiofluorescein^18^ (X = S), carbofluorescein^19^ (X = CMe_2_) and Si-fluorescein^20^ (X = SiMe_2_)) with lower LUMO energies and red shifts of both fluorescence excitation and emission maxima (Fig. 1). Their photostabilities, however, have not been systematically studied.

Rhodamine dyes are among the most widely used fluorescent labels in super-resolution live-cell microscopy^21^ due to their ability to cross intact mammalian cell membranes. Their cell permeability has been attributed to the equilibrium between the colourless spirolactone form prevailing in non-polar, aprotic media and the coloured, fluorescent zwitterionic form dominating in aqueous environment. This behaviour masks the potential off-targeting of lipophilic rhodamine-based ligands to cell membrane structures such as the endoplasmic reticulum (ER), but makes these probes unsuitable for labelling transmembrane proteins (*e.g.* β-adrenoceptors^22^). Thus, fluoresceins or negatively charged sulfonated rhodamine labels are often preferred in this context.^23^ Moreover, the use of positively charged rhodamine probes in live-cell imaging is severely limited by their off-targeting to mitochondria.^24^ Unlike fluoresceins, rhodamine labels remain fluorescent in acidic media and will appear as bright granules whenever accumulated in lysosomes (pH 4.6–5.0) and endosomes (pH 5–6),^9^ which prompted the development of more complex self-quenching fluorogenic rhodamine–tetrazine conjugates^25^ for wash-free imaging.^25*b*,*c*^

The superior brightness and photostability of rhodamines, especially those lacking *N*-alkyl substituents^26^ prompted us to develop their analogues bearing a negative charge delocalised across the xanthene core. Indeed, a recent report by Sharma *et al.*^27^ demonstrated that rhodols and rhodamines with electron-deficient *N*-(2,2,2-trifluoroethanesulfonyl) substituents, unlike colourless *N*-acylrhodamines,^28^ retain fluorescence in neutral and basic aqueous solutions. In the pioneering work of Wang *et al.*,^29^*N*-cyanoamide group was employed to increase the NH-acidity of rhodamine amides so as to fine-tune their spirocyclization behaviour. Here, we describe the synthesis and characterization of *N*-cyano- and *N*,*N*′-dicyanorhodamines as red-shifted, cell-permeant and biocompatible analogues of fluorescein dyes with excellent photostability, and demonstrate their application in stimulated emission depletion (STED) nanoscopy in living mammalian cells.

## Results and discussion

### Synthesis of *N*-cyanorhodamines and *N*,*N*′-dicyanorhodamines

**Fig. 2:**
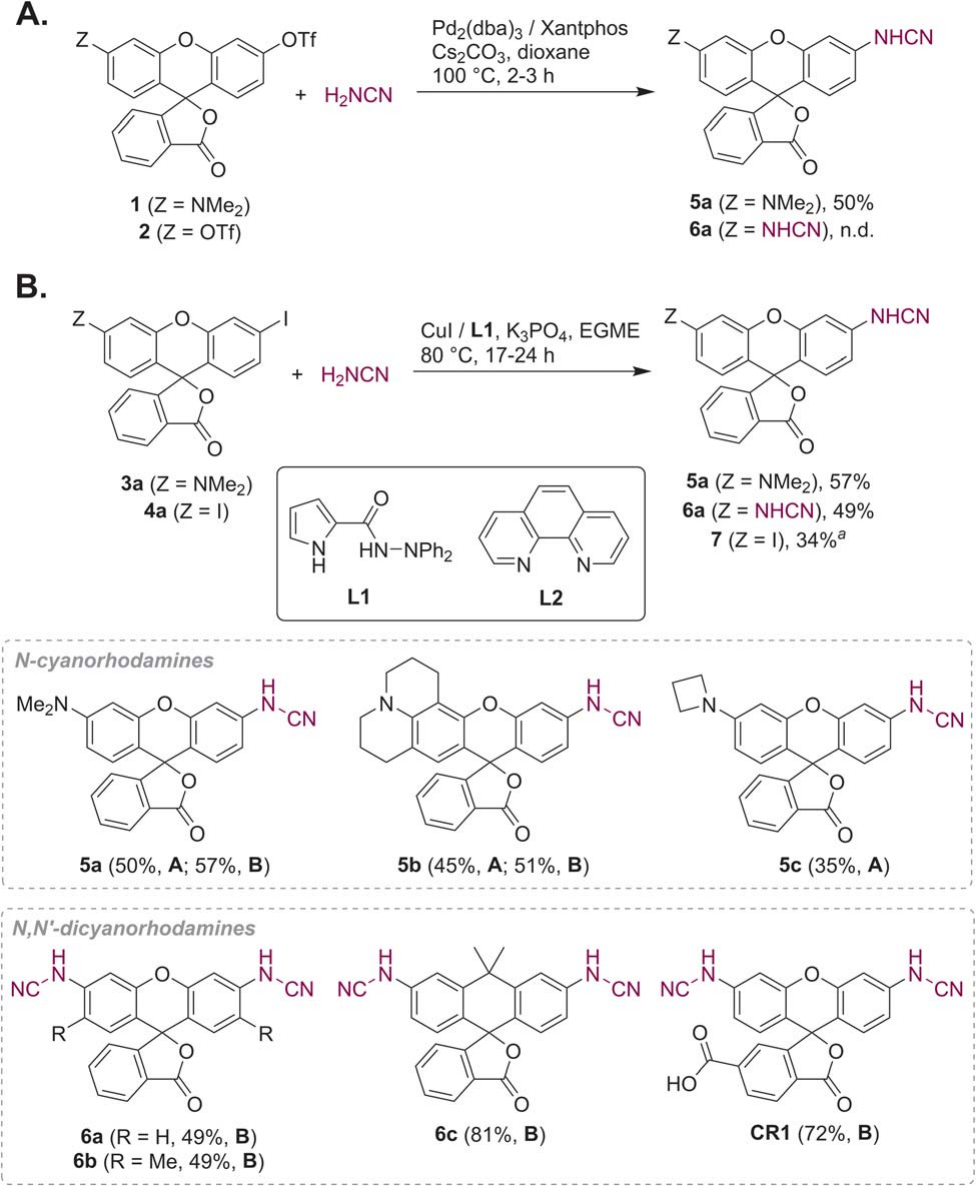
Synthesis of *N*-cyano- and *N*,*N*′-dicyanorhodamine fluorophores under Buchwald–Hartwig (A) and Ullmann (B) reaction conditions. ^*a*^ Ligand L2 was used instead of L1; EGME – ethylene glycol monomethyl ether.

Unsubstituted cyanamide is a weak acid (p*K*_a_ = 16.9 in DMSO), comparable to sulfonamides (p*K*_a_ = 10–18) but much stronger than most primary amides (p*K*_a_ = 17–25).^30^ It is ambiphilic and, as a nucleophile, less reactive than primary or secondary amines,^31^ which makes it a problematic cross-coupling partner. Based on a literature precedent,^32^ our initial attempts aimed at Pd-catalyzed Buchwald–Hartwig arylation of cyanamide. We have found, however, that this transformation is only successful with rhodol triflate 1 as substrate, but fails for fluorescein ditriflate 2 (Fig. 2A). This prompted us to look into Ullmann coupling for alternative reaction conditions^33^ using halofluorans 3, 4 as starting materials.^26^ Employing the catalytic system first reported by Ding *et al.*,^34^ we were able to obtain the asymmetric *N*-cyanorhodamines 5a and 5b from the corresponding monoiodides as well as the target *N*,*N*′-dicyanorhodamines (6a–c) from known 3′,6′-diiodofluorans (Fig. 2B). The use of a less active catalytic system (1,10-phenanthroline/CuI) resulted in formation of the mono-cyanamide product 7 in modest yield (see Table S1† for optimization details).

**Fig. 3:**
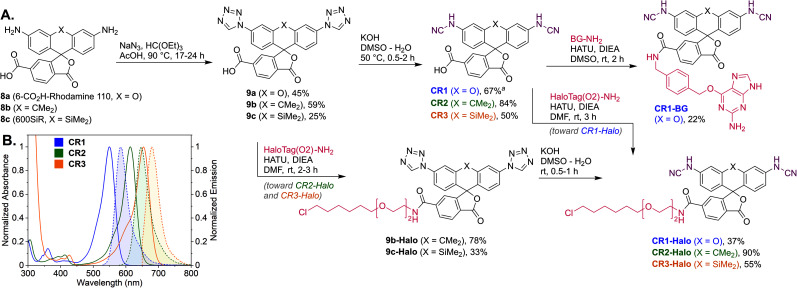
(A) Synthesis of *N*,*N*′-dicyanorhodamine dyes CR1–CR3 and fluorescent ligands for live-cell imaging. (B) Normalized absorption and fluorescence emission spectra of the dyes CR1–CR3 in phosphate buffer (pH 10). ^*a*^ Yield over 2 steps from 8a (without isolation of the intermediate 9a). BG – *O*^6^-benzylguanine (SNAP-tag ligand).

Regioisomerically pure 6-carboxyfluorescein analogue CR1, ready for derivatization to fluorescent ligands, could also be prepared under these conditions; however, the transformation proved difficult for 6-carboxylated 3′,6′-diiodocarbo- and Si-fluorans. An alternative strategy based on the base-induced degradation of 1-aryltetrazoles into *N*-arylcyanamides was therefore designed for these fluorophores (Fig. 3A). The unprotected 6-carboxyrhodamine 110 8a and its carbo- and Si-rhodamine analogues 8b and 8c were condensed with sodium azide and orthoformate ester to yield 3′,6′-bis-(1-tetrazolyl)fluoranes 9a–c in moderate yields (except for *600SiR*^5^). ω-Chloroalkane group (HaloTag ligand) was then introduced under peptide coupling conditions followed by treatment with KOH to unmask the *N*-cyanamido substituents. In the case of CR1, the direct coupling with the suitable self-labelling protein ligands was possible (albeit with lower yield) in the presence of free cyanamido substituents, but it is generally to be avoided because of the side reactivity of cyanamido groups.

### Photophysical characterization

**Fig. 4:**
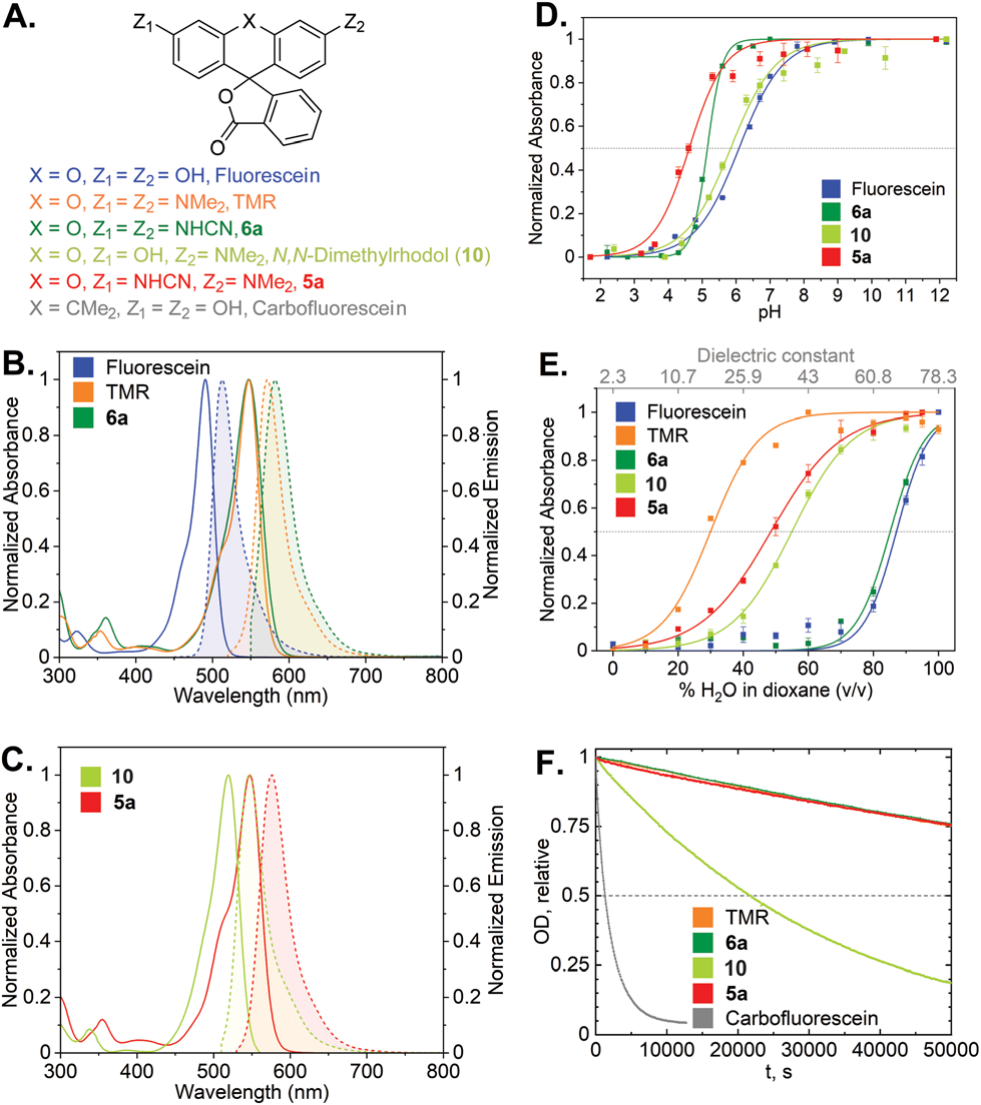
(A) Structures of cyanorhodamines 5a, 6a and related fluorophores. (B) Normalized absorption and fluorescence emission spectra of fluorescein, TMR and 6a (phosphate buffer, pH 9). (C) Normalized absorption and fluorescence emission spectra of rhodol and 5a (phosphate buffer, pH 9). (D) Normalized absorbance at the absorption maxima (Table 1) of fluorescein, rhodol, 5a (Fig. S5†) and 6a (Fig. S3†) at pH 2–12. (E) Normalized absorbance at the absorption maxima of fluorescein, rhodol, 5a (Fig. S6†) and 6a (Fig. S4†) in dioxane–water mixtures with varying water content. (F) Photobleaching curves (relative optical density at 530 nm over time) showing relative photostability of carbofluorescein, rhodol, TMR, 5a and 6a upon continuous irradiation at 530 nm in air-saturated borate buffer (pH 9.9).

With the synthetic access to the diverse *N*-cyano- and *N*,*N*′-dicyanorhodamines established, we next studied their optical and physicochemical properties. We first determined their absorption and fluorescence emission spectra (Fig. S1 and S2†), quantum yields and fluorescence lifetimes as well as the positions of relevant prototropic equilibria (Fig. S3–S6†) for comparison with the established cationic fluorophores used in cellular imaging (rhodamines) and cell-impermeant anionic fluorophores (fluoresceins). A summary of these properties is presented in Table 1 and Fig. 4A–E. According to our expectations, in neutral phosphate buffer *N*,*N*′-dicyanorhodamine 6a demonstrated absorption and emission maxima nearly exactly matching those of tetramethylrhodamine (TMR) with a 10 nm larger Stokes shift, showing ∼60 nm bathochromic shifts compared to fluorescein (Table 1, Fig. 4B). For the corresponding *N*-cyanorhodamine analogue 5a, the observed ∼30 nm bathochromic shift (as compared to *N*,*N*-dimethylrhodol 10) represented the effect of introducing a single *N*-cyanamido substituent (Fig. 4C).

**Table 1:** Summary of photophysical properties of *N*-cyano- and *N*,*N*′-dicyanorhodamines and reference fluorophores

Dye	*λ* ^abs^_max_ (nm) [PB/EtOH]^a^	*λ* ^em^_max_ (nm) [PB/EtOH]^a^	*ε* × 10^3^ (M^−1^ cm^−1^) [PB/EtOH]^a^	*Φ*_fl_ b [PB]^a^	*τ*_fl_ c (ns) [PB]^a^	*Φ*_bl_ d [PB]^a^	p*K*_a_	*D*_0.5_ e
Fluorescein	491^f^/502	512^f^/521	88/102	0.90 (ref. 7b)	4.1 (ref. 38)	n.d.*	6.1	69
TMR	547/548^g^	571/574^g^	123/113^g^	0.55^g^	2.7^g^	3.0 × 10^−7^	n.d.	24
*N*,*N*-Dimethylrhodol (10)	518^f^/511	547^f^/536	81^f^/78	0.22	1.0	2.2 × 10^−6^	5.8	43
5a	547^f^/553	577^f^/584	90^f^/86	0.38	1.7	6.3 × 10^−7^	4.6	39
5b	560/565	597/589	55/84	0.55	3.5	2.2 × 10^−6^	4.9	12
5c	547/554	576/583	62/72	0.61	2.9	1.2 × 10^−6^	4.7	43
6a	547^f^/568	582^f^/597	101^f^/111	0.24	1.2	1.6 × 10^−7^	5.1	63
6b	552^f^/574	584^f^/600	65^f^/79	0.30	2.2	6.8 × 10^−7^	5.1	50
6c	611/633	644/658	77/30	0.36	2.0	n.d.	7.0	67
CR1	549/569	584/597	105/119	0.23	1.2	n.d.	4.6	72
CR2	614/633	647/658	94/35	0.34	1.9	n.d.	7.0	n.d.
CR3	654/675	679/694	n.d.	0.22	1.3	n.d.	7.3	n.d.

a Optical properties were measured in 0.1 M phosphate buffer (PB, pH 10, unless indicated otherwise) and in ethanol + 1% (*v*/*v*) triethylamine (EtOH).

b Fluorescence quantum yield (absolute values, measured using an integrating sphere; see ESI).

c Fluorescence lifetime.

d Photobleaching quantum yield (determined from the initial rate upon irradiation at 530 nm; see ESI).

e Dielectric constant (interpolated) of a dioxane/water mixture, at which the normalized absorption of the dye is equal to one-half of the maximal value observed across the entire dioxane/water gradient (see Fig. 4E).

f pH 9.0.

g Values measured in ethanol + 0.1% (*v*/*v*) trifluoroacetic acid.^26^ n.d. = not determined. * Photobleaching quantum yield of carbofluorescein: 3.5 × 10^−5^.

For the *N*,*N*′-dicyanocarbo- and Si-rhodamines CR2, CR3, a similar trend was noted providing potential labelling options for multicolour fluorescence imaging, with fluorophore emission maxima reaching 690 nm for CR3 (Fig. 3B). The fluorescence quantum yield and lifetime values of 6a were lower than for fluorescein and TMR, but related well to those of rhodol 10 (Table 1). Chemical stability of the *N*-cyanamido group in the context of *N*-cyanorhodamines within the biologically relevant pH 5 to 10 range was also confirmed for 6a (Fig. S7†).

The dye 6a (p*K*_a_ = 5.1) was found to be significantly more acidic than fluorescein (p*K*_a_ = 6.1) and *N*,*N*-dimethylrhodol 10 (p*K*_a_ = 5.8), corresponding to 6a existing nearly completely in its colourless protonated form at pH ≤ 4 (Fig. 4D). Mono-*N*-cyanorhodamines 5a–c demonstrated an even higher acidity (p*K*_a_ = 4.6–4.9). The molecular brightness of *N*-cyanorhodamines could be increased by the introduction of an *N*-azetidinyl auxochromic group (5c) reported to suppress the transition into a twisted internal charge transfer (TICT) excited state,^6^ which undergoes non-radiative relaxation. Following the alternative strategy of rigidizing the dialkylamino substituent within the julolidine context of 5b (ref. 35) led to a similar improvement.

There is a general consensus that intact cell membrane permeability of rhodamine-type fluorophores depends on their propensity to reversibly close into colourless and non-fluorescent spirolactone forms^21^ (Fig. 1). The electrophilicity of the C-9 atom of the fluorescent xanthylium form of rhodamines and their analogues, responsible for the lactone ring closure, generally increases with the introduction of electron-withdrawing substituents at the amino groups. For every dye, the spirolactone-zwitterion equilibrium can be characterized by the *D*_0.5_ value,^36^ which is easily tested by recording a series of absorption or fluorescence emission spectra of the fluorophores in 1,4-dioxane/water mixtures with varying water content (Table 1, Fig. 4E). All studied *N*,*N*′-dicyanorhodamines demonstrated *D*_0.5_ ≥ 50, approaching the high value of the iconic Si-rhodamine dye (*D*_0.5_ = 64.5),^36^ widely appreciated in biological imaging for its fluorogenicity and far-red fluorescence emission (*λ*_max_ = ∼660 nm).^37^ For *N*-cyanorhodamines (except for the electron-rich 5b), lower *D*_0.5_ values were obtained (∼40), similar to carborhodamine dyes *580CP* and *610CP*.^36^

The stability of the cyanorhodamine dyes against photolysis was tested by continuous irradiation of their dilute (3.3 μM) solutions in basic sodium borate buffer (pH 9.9) under air with intermittent recording of absorption spectra. Under these conditions, fluorescein dyes are known to rapidly decompose into intractable mixtures of low-molecular weight polar products, while rhodamines are significantly more photostable. For benchmarking, the solutions of 5a and 6a were irradiated with a 530 nm light-emitting diode (LED), and their photobleaching rates were compared with those of TMR, *N*,*N*-dimethylrhodol (10) and carbofluorescein (accounting for the varying optical densities at the excitation wavelength, Fig. S8†). As evident from these data (Fig. 4F), the anionic forms of 5a and 6a are as photostable as the cationic TMR, while the rhodol and especially fluorescein dyes undergo rapid photodegradation.

### Fluorogenicity and binding kinetics of HaloTag ligands

The HaloTag protein,^11^ an engineered version of the *Rhodococcus rhodochrous* dehalogenase DhaA, forms a covalent ester bond between its active site aspartate residue and linear ω-chloroalkanes in an S_N_2-type reaction. Since the uncatalyzed reactivity of simple chloroalkanes is very low, using HaloTag fusion proteins allows for selective bioorthogonal labelling with only a small molecular weight adjunct. As HaloTag enzyme variants were initially optimized for the TMR chloroalkane (TMR-Halo, Table 2) substrate for fluorescent tagging, their reaction rates with cationic and zwitterionic rhodamine-type ligands are unprecedentedly high for self-labelling covalent tags, approaching the diffusion limits for ligands derived from the dyes *610CP* and *abberior LIVE 580*.^39^ On the contrary, the apparent second-order reaction rates reported for uncharged small molecular weight chloroalkanes and the negatively charged sulfonated rhodamine *Alexa Fluor 488* were ∼1000 times lower (*k*_app_ 10^4^–10^5^ M^−1^ s^−1^ with HaloTag7).^39^ We therefore considered important to evaluate the labelling kinetics of *N*,*N*′-dicyanorhodamine HaloTag ligands, negatively charged at physiological pH values, prior to performing fluorescence imaging experiments. The specific labelling of the HaloTag7 protein with CR1-Halo and CR2-Halo was confirmed by sodium dodecyl sulfate polyacrylamide gel electrophoresis (SDS PAGE; Fig. S9†), and chemoselectivity of the labelling reaction was verified by mass spectrometry (Fig. S10†). Fortunately, all three fluorescent substrates CR1-Halo–CR3-Halo demonstrated *k*_app_ = ∼10^6^ M^−1^ s^−1^ with the HaloTag7 protein (Table 2, Fig. S11†), a kinetic behaviour comparable to the SNAP-tag protein/*O*^6^-benzylguanine ligand system^13^ widely used in live-cell fluorescent labelling. As we anticipated, the *k*_app_ values for *N*,*N*′-dicyanorhodamine-derived ligands laid between those of TMR (bearing a cationic 3,6-diaminoxanthylium core in its fluorescent zwitterionic form) and fluorescein (with anionic 3-hydroxy-6-fluorone chromophore).

**Table 2:** Apparent second-order labelling rate constants (*k*_app_) with HaloTag7 for fluorescent substrates CR1-Halo–CR3-Halo and reference dye ligands (TMR, fluorescein)

Substrate	*k*_app_ (M^−1^ s^−1^) (value ± standard deviation)
CR1-Halo	(4.07 ± 0.02) × 10^6^
CR2-Halo	(3.92 ± 0.03) × 10^6^
CR3-Halo	(2.85 ± 0.05) × 10^6^
Fluorescein-Halo	(1.01 ± 0.01) × 10^6^
TMR-Halo	(2.86 ± 0.01) × 10^7^
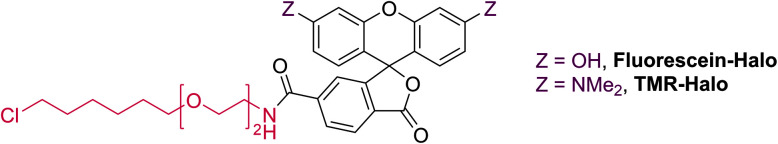	

Numerous carborhodamine and Si-rhodamine-based HaloTag ligands have demonstrated a fluorogenic response, *i.e.* an increase in fluorescence intensity upon covalent binding to the HaloTag protein.^3^ In the rhodamine series, only fluorinated dyes with electron-withdrawing *N*-substituents (2,2,2-trifluoroethyl, 3,3-difluoroazetidinyl) or with a lactone-to-lactam modification^3,36^ showed a similar behaviour. Accordingly, only the CR3-Halo ligand demonstrated moderate fluorogenicity, comparable to the Si-rhodamine HaloTag ligand.^36,37^ The two other cyanorhodamine ligands and Fluorescein-Halo showed no fluorogenic response upon binding to HaloTag7 (Fig. S12†). It has been previously noticed that this response correlates with an increase in emission intensity of fluorescent HaloTag ligands bound non-specifically to bovine serum albumin (BSA) in buffered solutions upon addition of anionic surfactant sodium dodecyl sulfate (SDS).^36^ Conversely, upon addition of cationic cetyltrimethylammonium bromide (CTAB) detergent, the fluorescence intensity of fluorogenic rhodamine ligands decreases. We hypothesized that this relation would be reverted for negatively charged *N*,*N*′-dicyanorhodamine derivatives. Indeed, the fluorescence of BSA-bound CR2-Halo and CR3-Halo markedly decreased upon addition of SDS to the medium, likely due to unfavourable electrostatic interactions forcing spirolactonization under local environment conditions (Fig. S13†). However, virtually no response to the presence of SDS or CTAB was observed for CR1-Halo and Fluorescein-Halo. Since zwitterionic rhodamines, rhodols and fluoresceins have previously been developed into a series of transmembrane potential sensors,^40^ cell-permeant and non-fluorogenic *N*-cyanorhodamines with distinct environmental sensitivity may provide additional options for the synthetic design of similar fluorescent reporters for functional imaging.

### Biocompatibility and cellular imaging

**Fig. 5:**
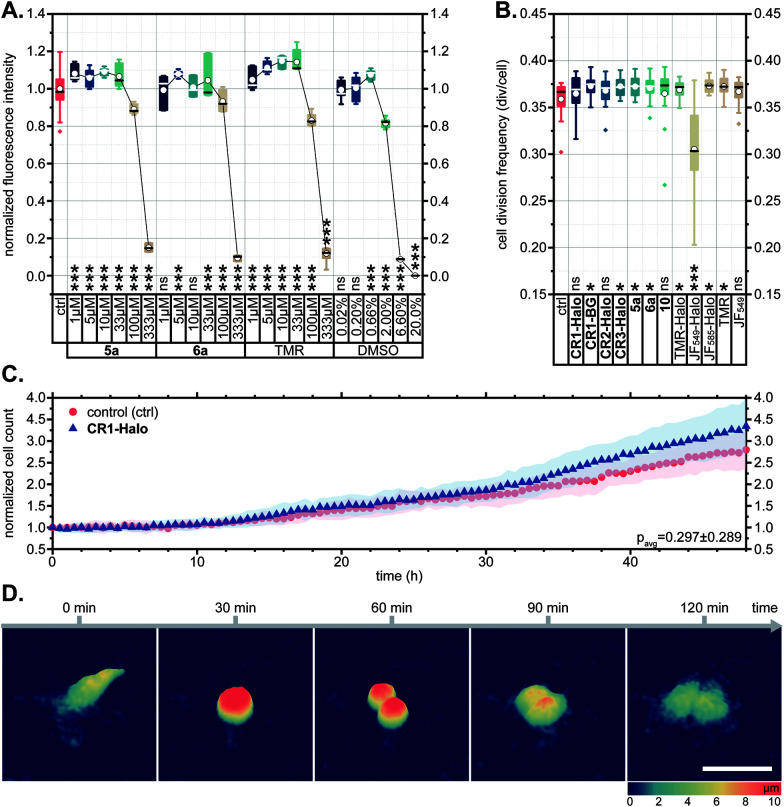
(A) CellTiter-Blue cell viability assay showing no toxicity for 5a, 6a and TMR in comparison with DMSO vehicle control (U-2 OS cells, over 24 h). (B–D) U-2 OS cell proliferation as followed by holographic time-lapse imaging cytometry. (B) Cell division frequency over 48 h in presence of 5 μM dye. (C) Unaltered cell count and (D) visualization of a normal cell division in the presence of 5 μM CR1-Halo. Scale bar: 50 μm. Statistical significance: ns – no significant difference to control, *p < 0.05, **p < 0.005, ***p < 0.0005 (see Tables S3 and S4†).

In several reports describing the cytotoxicity of rhodamine dyes (*e.g.* Rhodamine 123,^41^ Rhodamine 6G^42^), cytotoxic effects have been attributed to the cationic form accumulating in mitochondria and disrupting the synthesis of adenosine triphosphate (ATP), the primary renewable energy source of the mammalian cell. For this reason, we first evaluated the effects of *N*-cyanorhodamine core compounds 5a, 6a on human bone osteosarcoma epithelial (U-2 OS) cell viability and noted the absence of cytotoxicity at concentrations up to 100 μM in the medium over 24 h (Fig. 5A). These dye loadings far exceed the usual ≤5 μM concentrations employed in cellular imaging; indeed, the toxic concentrations were comparable to that of the DMSO vehicle and did not surpass the tolerated concentration limits for TMR. The cell morphology and proliferation rates of living U-2 OS cells, monitored by means of holographic imaging cytometry in the presence of 5 μM of 5a, 6a or various CR1–CR3 derivatives in the culture medium, were unaffected over at least 48 h (Fig. 5B–D and S14†).

**Fig. 6:**
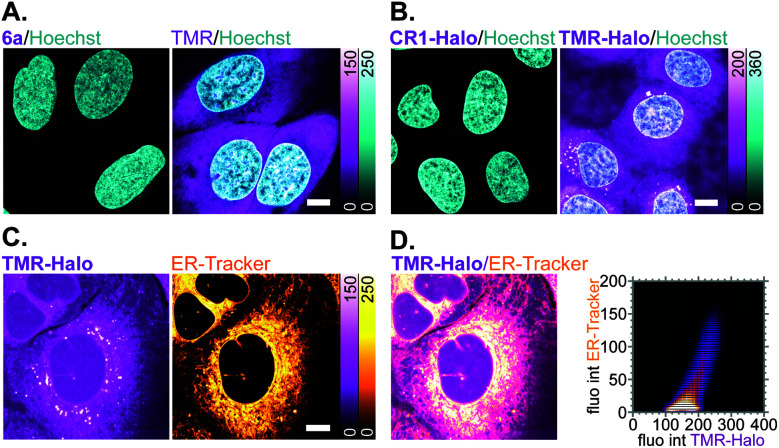
(A and B) Off-target affinity of TMR- *vs. N*,*N*′-dicyanorhodamine-based fluorophores and HaloTag ligands in living U-2 OS cells treated with 5 μM of (A) 6a or TMR, (B) CR1-Halo or TMR-Halo over 18 h and counter-stained with Hoechst 33342 (8 μM, 10 min). (C) Colocalization of off-target labelling in living U-2 OS cells with TMR-Halo (5 μM) and *ER-Tracker Blue-White DPX* (1 μM). (D) Overlay image of (C) and corresponding fluorogram for colocalization analysis. Scale bars: 10 μm.

Off-target labelling artefacts, most commonly observed as diffuse fluorescent staining of mitochondria, lysosomes and/or plasma membrane structures such as the ER, are the main deterrent in the development of selective fluorescent labels for living cells. To compare the off-target affinity of *N*,*N*′-dicyanorhodamines with commonly used TMR-based probes, living U-2 OS cells were treated overnight with identical (5 μM) concentrations of fluorophores 6a, TMR and the corresponding HaloTag ligands CR1-Halo and TMR-Halo. While neither 6a nor CR1-Halo demonstrated any intracellular staining, TMR-treated samples showed diffuse fluorescence of membrane structures including the plasma membrane (Fig. 6A), and TMR-Halo predominantly accumulated in the ER (Fig. 6B), which was confirmed by the successful colocalization with *ER-Tracker Blue-White DPX* probe (average Pearson's correlation coefficient 0.62 ± 0.05 (*N* = 9), Fig. 6C and D).

**Fig. 7:**
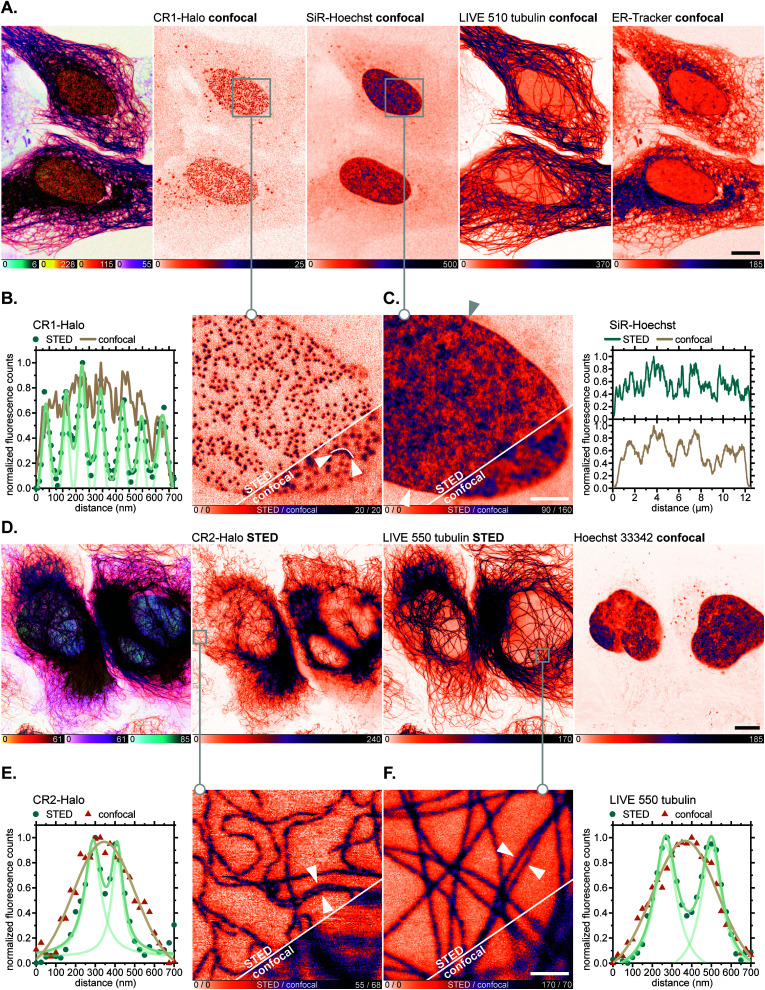
Live-cell super-resolution imaging with *N*,*N*′-dicyanorhodamine-derived HaloTag labels. (A–C) Four-colour (2× STED, 2× confocal) image of living U-2 OS-NUP96-Halo cells labelled with CR1-Halo (5 μM, Nup96), 6-SiR-Hoechst^44^ (200 nM, DNA), *abberior LIVE 510 tubulin* (250 nM, β-tubulin) and *ER-Tracker Blue-White DPX* (250 nM, ER) over 6 h. (D–F) Three-colour (2× STED, 1× confocal) image of living U-2 OS-Vim-Halo cells labelled with CR2-Halo (1 μM, vimentin), *abberior LIVE 550 tubulin* (4-TMR-LTX,^4*b*^ 500 nM, β-tubulin) for 5 h and Hoechst 33342 (8 μM, DNA) for 10 min. Overview image of whole cells showing individual colour channels (A and D), zoom-in of confocal *vs.* STED images with CR1-Halo (B), SiR-Hoechst (C), CR2-Halo (E) and *abberior LIVE 550 tubulin* (F) showing intensity profiles across selected regions of the images (marked with arrows). Scale bars: 10 μm (A and D), 1 μm (B and C).

Having verified the absence of both fluorophore-directed off-targeting and cytotoxicity for *N*-cyanorhodamines, and good photostability and fast labelling kinetics for our *N*-cyanorhodamine HaloTag probes, we finally performed multicolour confocal and STED fluorescence microscopy in living mammalian cells in combination with previously established live-cell and STED-compatible fluorophores. To this end, several new label combinations permitting up to 4-colour imaging (2× confocal, 2× STED) were proposed and evaluated in genetically modified U-2 OS cell lines expressing fusion proteins of suitable cellular structures targeted with a HaloTag. In one example, the HaloTag-fused nuclear pore complex protein Nup96 was labelled in living U-2 OS-NUP96-Halo cells^43^ (engineered with the clustered regularly interspaced short palindromic repeats (CRISPR) technique) with the CR1-Halo ligand (5 μM, 6 h), co-stained for nuclear chromatin with the far-red label SiR-Hoechst,^44^ for microtubular cytoskeleton with *abberior LIVE 510 tubulin*, and for ER with *ER-Tracker Blue-White DPX* commercial probes at submicromolar concentrations (Fig. 7A). Significantly improved resolution of individual nucleoporin clusters was achieved with 775 nm STED nanoscopy with little to no diffuse background in the cells (Fig. 7B). The density of SiR-Hoechst-labelled chromatin in the nucleus could be simultaneously evaluated with subdiffraction precision (Fig. 7C). Furthermore, fused HaloTag-vimentin protein in living CRISPR-engineered U-2 OS-Vim-Halo cells^35^ was tagged with the CR2-Halo probe (1 μM, 5 h; spectrally identical with a widely utilized carborhodamine fluorophore *610CP*),^36*a*,45^ together with the *abberior LIVE 550 tubulin* probe and Hoechst 33342 for co-staining nuclear DNA (Fig. 7D). Both vimentin (Fig. 7E) and tubulin filaments (Fig. 7F) were resolved with subdiffraction resolution and without cross-talk between the two red-fluorescent labels. On the contrary, while the CR3-Halo ligand provided good quality confocal images of vimentin in living U-2 OS-Vim-Halo cells and was STED-compatible (Fig. S15†), it was impossible to record continuous signal from individual filaments under STED conditions, since the majority of CR3 fluorophore population remained in the colourless spirolactone form under the physiological conditions of the live cell sample.

**Fig. 8:**
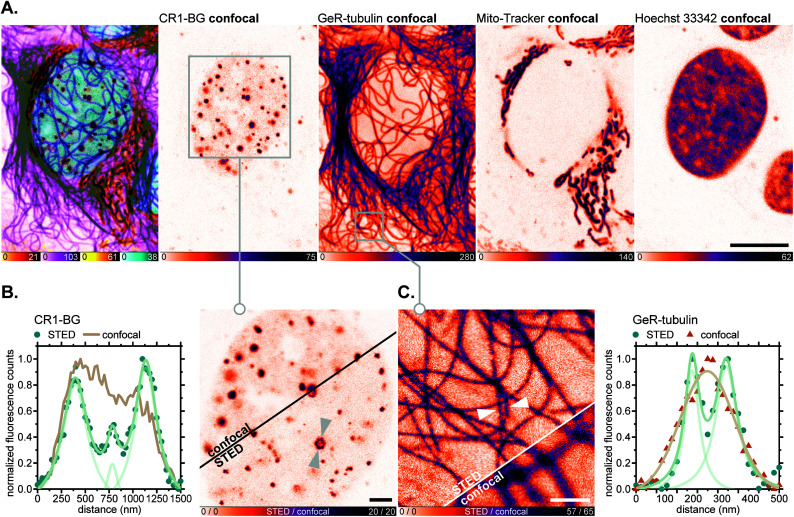
Live-cell super-resolution imaging with *N*,*N*′-dicyanorhodamine-derived SNAP-tag label CR1-BG. (A–C) Four-colour (2× STED, 2× confocal) image of living U-2 OS cells transiently expressing PMLIII-SNAP and labelled with CR1-BG (5 μM, PML-nuclear bodies), GeR-tubulin^36*b*^ (1 μM, β-tubulin), *MitoTracker Green FM* (2 μM, mitochondria) for 5 h and Hoechst 33342 (8 μM, DNA) for 10 min. Overview image of whole cells showing individual colour channels (A), zoom-in of confocal *vs.* STED images with CR1-BG (B) and GeR-tubulin (C) showing intensity profiles across individual nuclear bodies (B) or filaments (C) (marked with arrows). Scale bars: 10 μm (A), 2 μm (B), 1 μm (C).

For fluorescence imaging with the SNAP-tag ligand CR1-BG, U-2 OS cells were transiently transfected to achieve overexpression of a SNAP-tag fusion with the promyelocytic leukemia protein (PML),^46*a*^ which, after a series of post-translational modifications and oligomerization, forms distinct nuclear subcompartments (nuclear bodies up to 1 μm in diameter).^46*b*^ Upon labelling these with CR1-BG (5 μM, 5 h) and co-staining with GeR-tubulin,^36*b*^*Mito-Tracker Green FM* (for mitochondria) and Hoechst 33342 (for DNA; Fig. 8A), the hollow-spherical structure of PML-nuclear bodies was resolved with 775 nm STED nanoscopy (Fig. 8B). Subdiffraction resolution of individual microtubules labelled with GeR-tubulin was simultaneously achieved (Fig. 8C).

In all of the multicolour imaging examples above, very little to no background or appreciable off-targeting artefacts were observed with *N*,*N*′-dicyanorhodamine fluorescent ligands. We consider this selectivity remarkable given the high fluorophore concentrations (1–5 μM) in the medium, relatively long incubation times and the absence of innate fluorogenic behaviour of the new HaloTag labels. These observations support our alternative approach to high-contrast live-cell labelling, employing the negatively charged cell-permeant xanthene fluorophores instead of fluorogenic rhodamine amides with decreased content of the fluorescent zwitterionic form in the equilibrium.^3*b*,29^

## Conclusions

Rhodamine fluorophores, bearing a net negative charge at neutral pH values due to the presence of anionic groups (sulfonate^47*a*^ or carboxylate^47*b*^), have been previously recognized as cell-impermeant and utilized solely in the development of specific probes for extracellular targets or for labelling fixed cells. While significant efforts have focused on the development of medium-polarity-sensitive fluorogenic probes,^3,29,36^ the unselective binding of these probes is simply masked by their reduced fluorescence in non-polar environments such as lipid-rich membrane structures. The main drawback of this approach is that the fluorescence of the zwitterionic form of rhodamines is maintained or even enhanced in acidic compartments, and that accumulation of lipophilic rhodamines in their non-fluorescent spirolactone form may at least partially be responsible for their cytotoxicity. These effects become the main limiting factor determining the labelling conditions such as probe concentration and incubation time, and may easily become prohibitive for fluorescent ligands with lower binding affinities.

On the other hand, the live-cell application of anionic fluorescein-based probes is free from these drawbacks but requires chemical protection (usually in the form of acetate ester or acetoxymethyl ether) and has to rely on enzymatic cleavage to recover the fluorescent label. In addition, all reported fluoresceins and rhodols show hypsochromic absorption and emissions shifts and poor photostability as compared to the corresponding rhodamine dyes.

In our work, we have proposed a class of *N*-cyanorhodamine fluorophores, which maintain live cell permeability despite being negatively charged within the physiological pH range. In particular, *N*,*N*′-dicyanorhodamine dyes demonstrated absence of toxicity, high photostability and sufficient spectral diversity in the orange- to far-red emission range permitting their use in long-term labelling and multicolour super-resolution microscopy. We have performed the initial evaluation of *N*-cyanorhodamine label combinations for three- and four-colour live-cell imaging, and anticipate the development of photoactivatable and/or enzymatically activatable fluorogenic probes designed around these original core structures.

## Data availability

Full experimental and characterization data is available in the ESI.†

## Author contributions

L. Heynck: investigation, methodology, formal analysis, validation; J. Matthias: investigation, formal analysis, visualization; M. L. Bossi: investigation, data curation, formal analysis, methodology, software; A. N. Butkevich: conceptualization, project administration, resources, investigation, writing (original draft, review & editing); S. W. Hell: supervision, funding acquisition. All authors have given approval to the final version of the manuscript.

## Conflicts of interest

S. W. H. owns shares of Abberior GmbH and Abberior Instruments GmbH whose dyes and STED microscope, respectively, have been used in this study.

## Supplementary Material

SC-013-D2SC02448A-s001
