# Supplementary material for: N-Cyanorhodamines: cell-permeant, photostable and bathochromically shifted analogues of fluoresceins
Source: Chem Sci. 2022 Jun 27;13(28):8297–306. doi: 10.1039/d2sc02448a (PMC9297387; doi:10.1039/d2sc02448a)
Supplement: SC-013-D2SC02448A-s001 [file SC-013-D2SC02448A-s001.pdf]

Electronic Supplementary Material (ESI) for Chemical Science.
This journal is © The Royal Society of Chemistry 2022

Supplementary Information

N-Cyanorhodamines: cell-permeant, photostable and bathochromically shifted analogues of fluoresceins

Lukas Heynck[a], Jessica Matthias[a], Mariano L. Bossi[b], Alexey N. Butkevich*[a,b] and Stefan W. Hell[a,b]

[a] Department of Optical Nanoscopy, Max Planck Institute for Medical Research, 69120 Heidelberg, Germany

[b] Department of NanoBiophotonics, Max Planck Institute for Multidisciplinary Sciences, 37077 Göttingen, Germany

* E-mail: alexey.butkevich@mr.mpg.de

Table of Contents

Table of Contents 2

Supplementary Figures 6

Figure S1. Absorption and emission spectra of *N*,*N′*-dicyanorhodamines compared to their fluorescein and rhodamine analogues. 6

Figure S2. Absorption and emission spectra of *N*-cyanorhodamines compared to their rhodol analogues. 7

Figure S3. Absorption and emission spectra of *N*,*N′*-dicyanorhodamine (**6a**) in 0.1M sodium phosphate buffer (pH 2-12). 8

Figure S4. Absorption and emission spectra of *N*,*N′*-dicyanorhodamine (**6a**) in aqueous dioxane solutions with varying water content (0-100%). 8

Figure S5. Absorption and emission spectra of *N*-cyanorhodamine **5a** in 0.1M sodium phosphate buffer (pH 2-12). 9

Figure S6. Absorption and emission spectra of *N*-cyanorhodamine **5a** in aqueous dioxane solutions with varying water content (0-100%). 9

Figure S7. Absorption spectra of *N*,*N′*-dicyanorhodamine (**6a**) solutions in air-saturated 0.1M phosphate buffer (+20% v/v DMSO) at pH 5, 7 and 10 recorded over 20 h. 10

Figure S8. Initial photobleaching rates and photobleaching quantum yields of *N*-cyanorhodamines (**5a-c**), *N,N′*-dicyanorhodamines (**6a,b**) and reference dyes in borate buffer (pH 9.9). 11

Figure S9. SDS PAGE of HaloTag7 labeled with **CR1-Halo** and **CR2-Halo**. 12

Figure S10. Mass spectrometry of HaloTag7 labeled with **CR1-Halo**. 13

Figure S11. Labeling kinetics of HaloTag7 with fluorescent ω-chloroalkane ligands. 15

Figure S12. Fluorescence intensity changes upon binding to HaloTag7 protein. 16

Figure S13. Fluorescence intensity changes upon addition of surfactants (SDS or CTAB) to the solutions of HaloTag ligands containing 0.1 mg/mL BSA. 17

Figure S14. Assessment of cytotoxicity. 18

Figure S15. **CR3-Halo** confocal and STED imaging. 19

Supplementary Tables 20

Table S1. Ullmann amination of 3',6'-diiodofluoran (**4a**) with cyanamide: screening of the reaction conditions. 20
Table S2. Imaging parameters used for the acquisition of live cell STED and confocal microscopy data. 21
Table S3. Statistical information on CellTiter-Blue cell viability assay. 23
Table S4. Statistical information on holographic time-lapse imaging. 24
Supplementary Methods 25
General experimental information 25
Thin layer chromatography 25
Preparative flash column chromatography 25
High-Performance Liquid Chromatography (HPLC) and Mass Spectrometry (MS) 25
NMR spectra 26
Optical spectroscopy 27
Chemometric analysis of the photobleaching reaction kinetics 28
HaloTag7 labeling specificity 29
HaloTag7 labeling kinetics 30
Fluorescence intensity changes of substrates in the presence of a HaloTag7 protein 30
Fluorescence intensity changes of substrates in the presence of surfactants 31
Cell culture 32
Holographic time-lapse imaging 33
Cell viability assay 33
Live cell STED and confocal imaging 35
Statistical Aspects 36
Synthesis and properties of new compounds 37
Preparation of the starting materials 37
N,*N*-Dimethylrhodol triflate (**1**) 37
3'-(Dimethylamino)-6'-iodofluoran (**3a**) 38
6-Carboxyrhodamine 110 (**8a**) 39
6-Carboxycarborhodamine **8b** 40
Compound **S15** 42

Compound **S16** 43
Preparation of *N*-cyanorhodamines by Buchwald-Hartwig amination 43
Compound **5a** 43
Compound **5b** 44
Compound **5c** 45
Preparation of *N*-cyanorhodamines by Ullmann coupling 46
Compound **5a** 46
Compound **5b** 47
Compound **6a** 48
Compound **6b** 49
Compound **6c** 50
Compound **7** 51
Compound **CR1** 52
Preparation of *N*-cyanorhodamines via base-induced degradation of 1-aryltetrazoles 53
Compound **9a** 53
Compound **9b** 53
Compound **9c** 54
Compound **CR1** 55
Compound **CR2** 56
Compound **CR3** 56
Preparation of self-labelling tag ligands from the dyes **CR1-CR3** 57
CR1-Halo 57
CR1-BG 58
9b-Halo 59
CR2-Halo 60
9c-Halo 60
CR3-Halo 61
Supplementary References 63
NMR spectra 65
S13 65

S14 66
S15 68
S16 70
1a 72
3a 75
5a 77
5b 79
5c 81
6a 83
6b 85
6c 87
7 89
8a 91
8b 93
9a 95
9b 97
9c 99
9b-Halo 101
9c-Halo 103
CR1 105
CR2 107
CR3 109
CR1-Halo 111
CR1-BG 113
CR2-Halo 115
CR3-Halo 117

Supplementary Figures

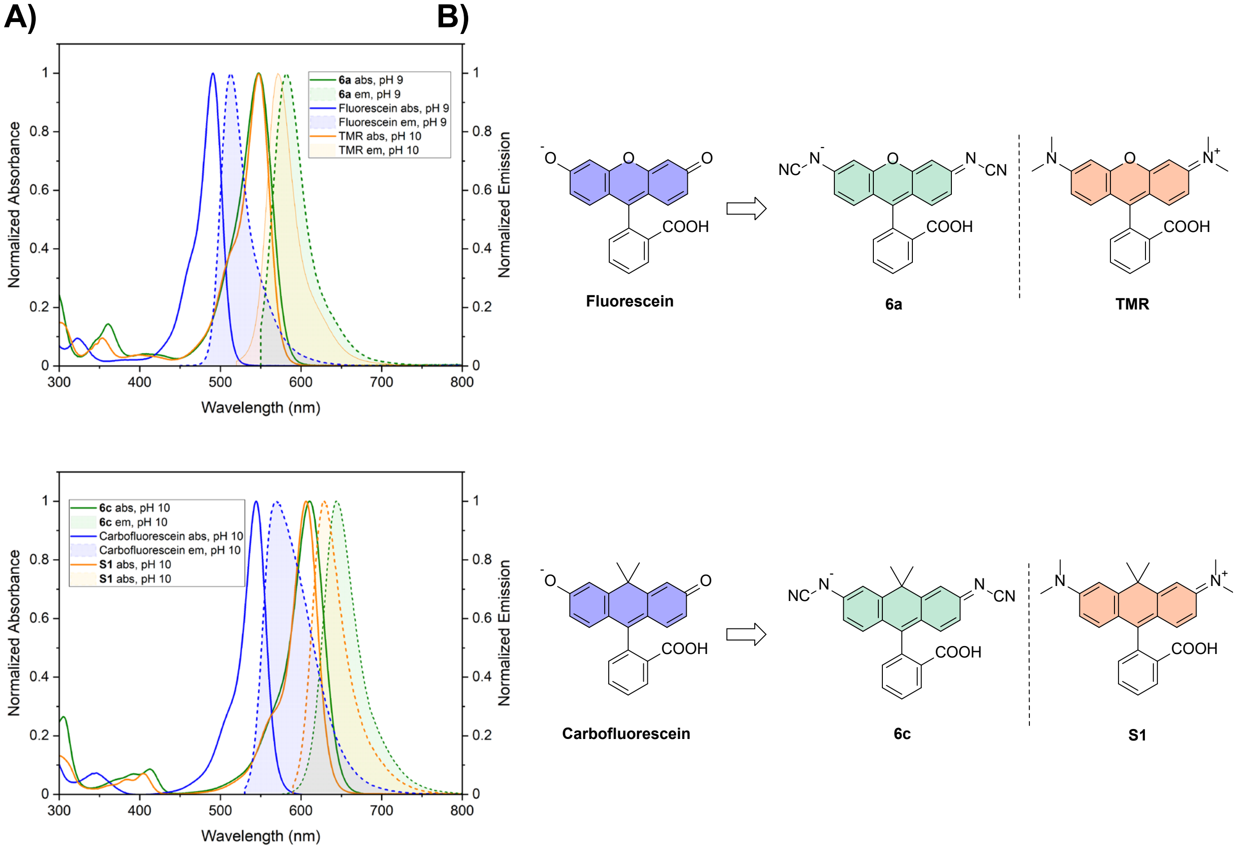

Figure S1. Absorption and emission spectra of *N*,*N′*-dicyanorhodamines compared to their fluorescein and rhodamine analogues. A) Normalized absorption and emission spectra in 0.1M phosphate buffer (pH 9 *or* 10); B) chemical structures of *N,N′*-dicyanorhodamine (**6a**), *N,N′*-dicyanocarborhodamine (**6c**) and their structural analogues (fluorescein, TMR, carbofluorescein, **S1** [S1]).

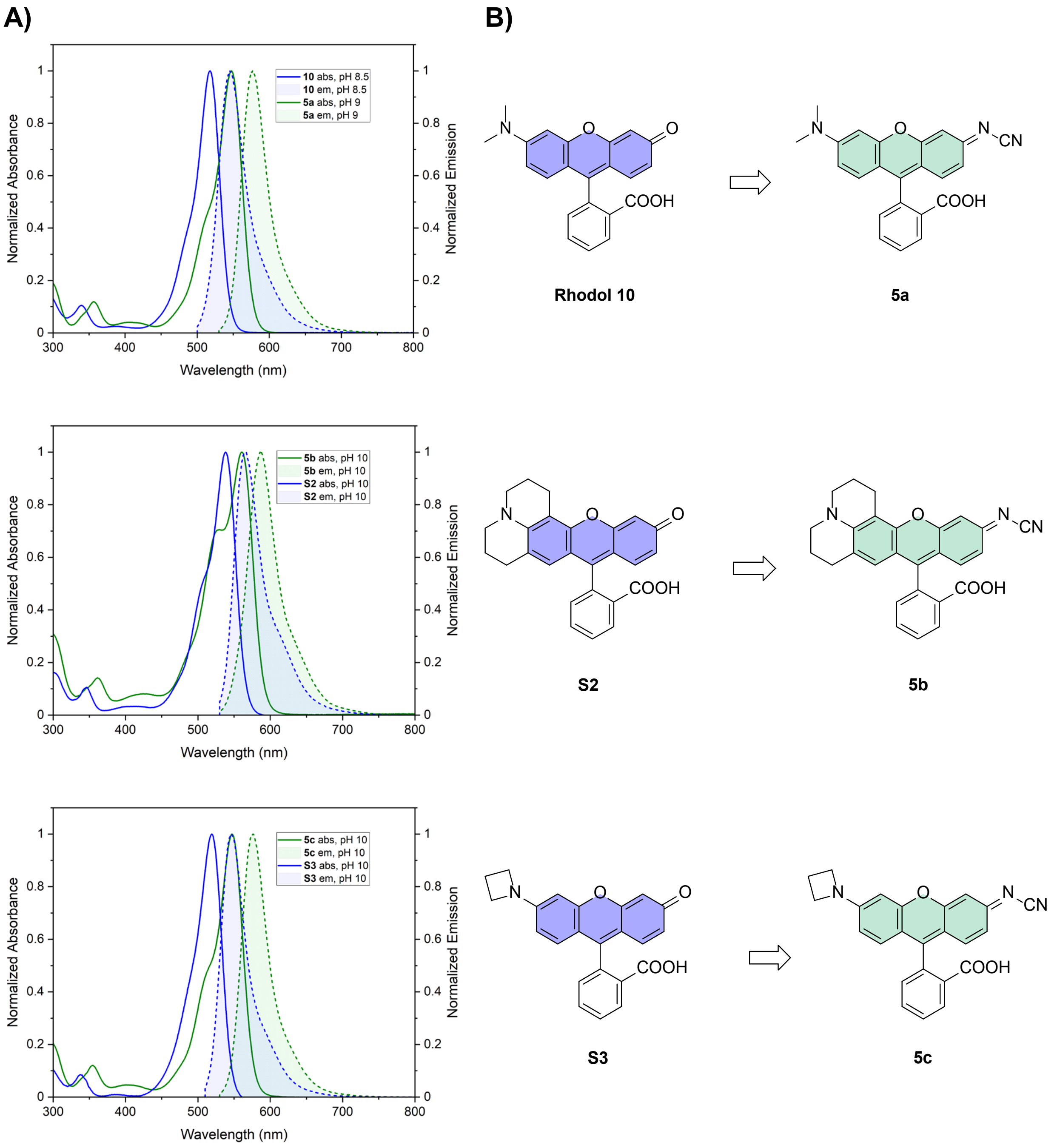

Figure S2. Absorption and emission spectra of *N*-cyanorhodamines compared to their rhodol analogues. A) Normalized absorption and emission spectra in 0.1M phosphate buffer (pH 8.5, 9 *or* 10); B) chemical structures of *N*-cyanorhodamines (**5a-c**) and rhodols (**10**, **S2** [S2], **S3** [S1]).

6a:

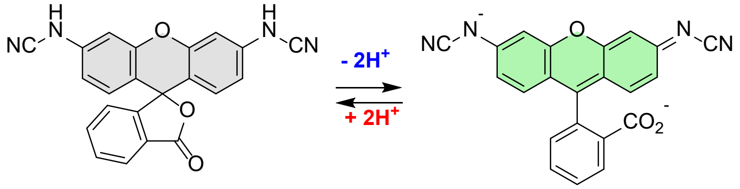

absorption

fluorescence emission

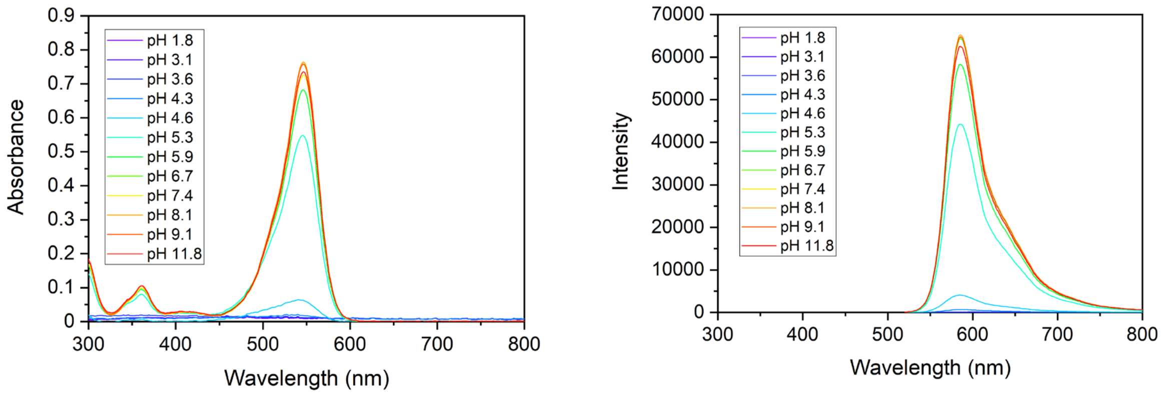

Figure S3. Absorption and emission spectra of *N,N'*-dicyanorhodamine (6a) in 0.1M sodium phosphate buffer (pH 2-12).

absorption

fluorescence emission

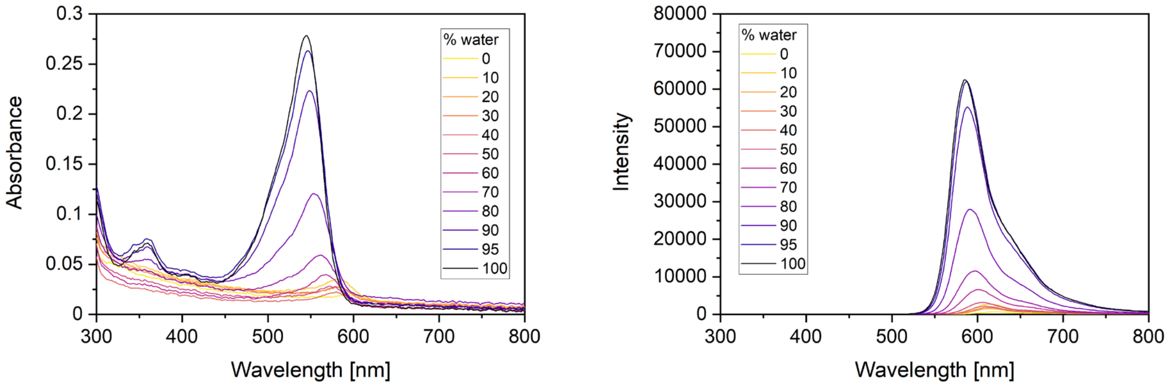

Figure S4. Absorption and emission spectra of *N,N'*-dicyanorhodamine (6a) in aqueous dioxane solutions with varying water content (0-100%).

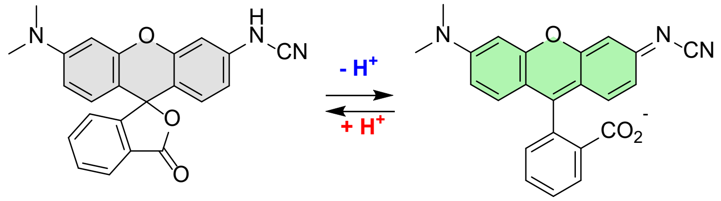

absorption

fluorescence emission

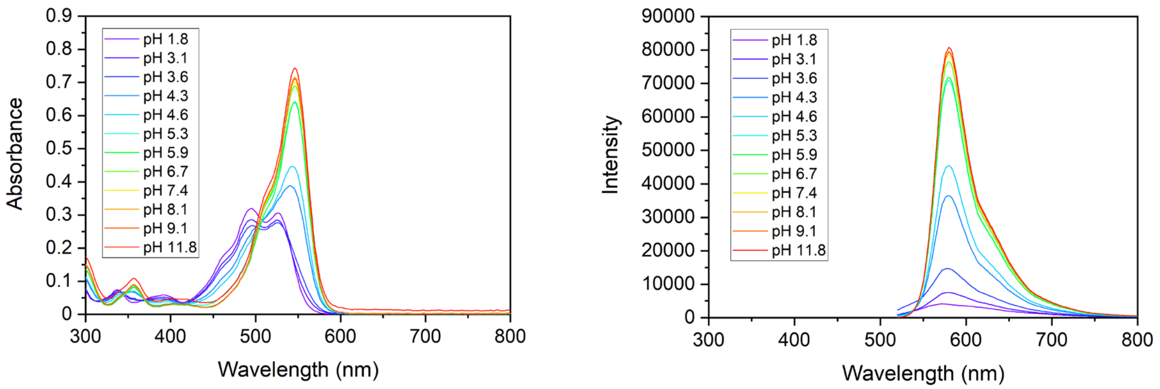

Figure S5. Absorption and emission spectra of *N*-cyanorhodamine 5a in 0.1M sodium phosphate buffer (pH 2-12).

absorption

fluorescence emission

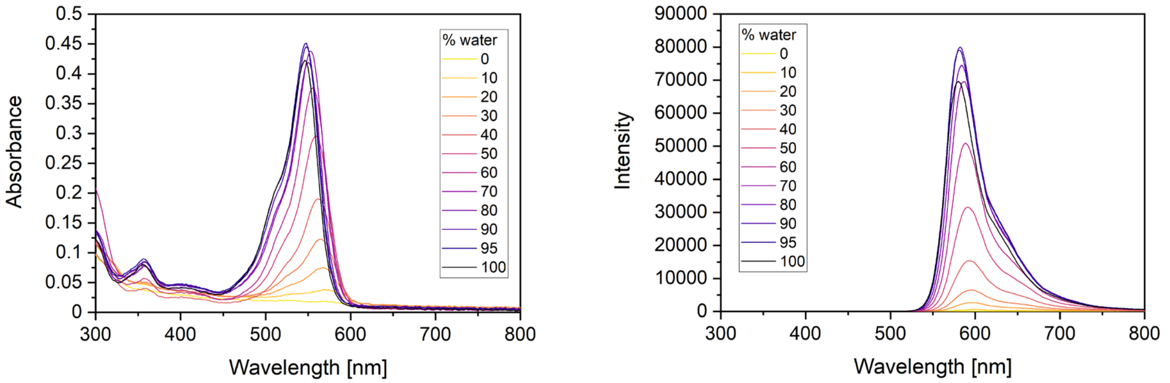

Figure S6. Absorption and emission spectra of *N*-cyanorhodamine 5a in aqueous dioxane solutions with varying water content (0-100%).

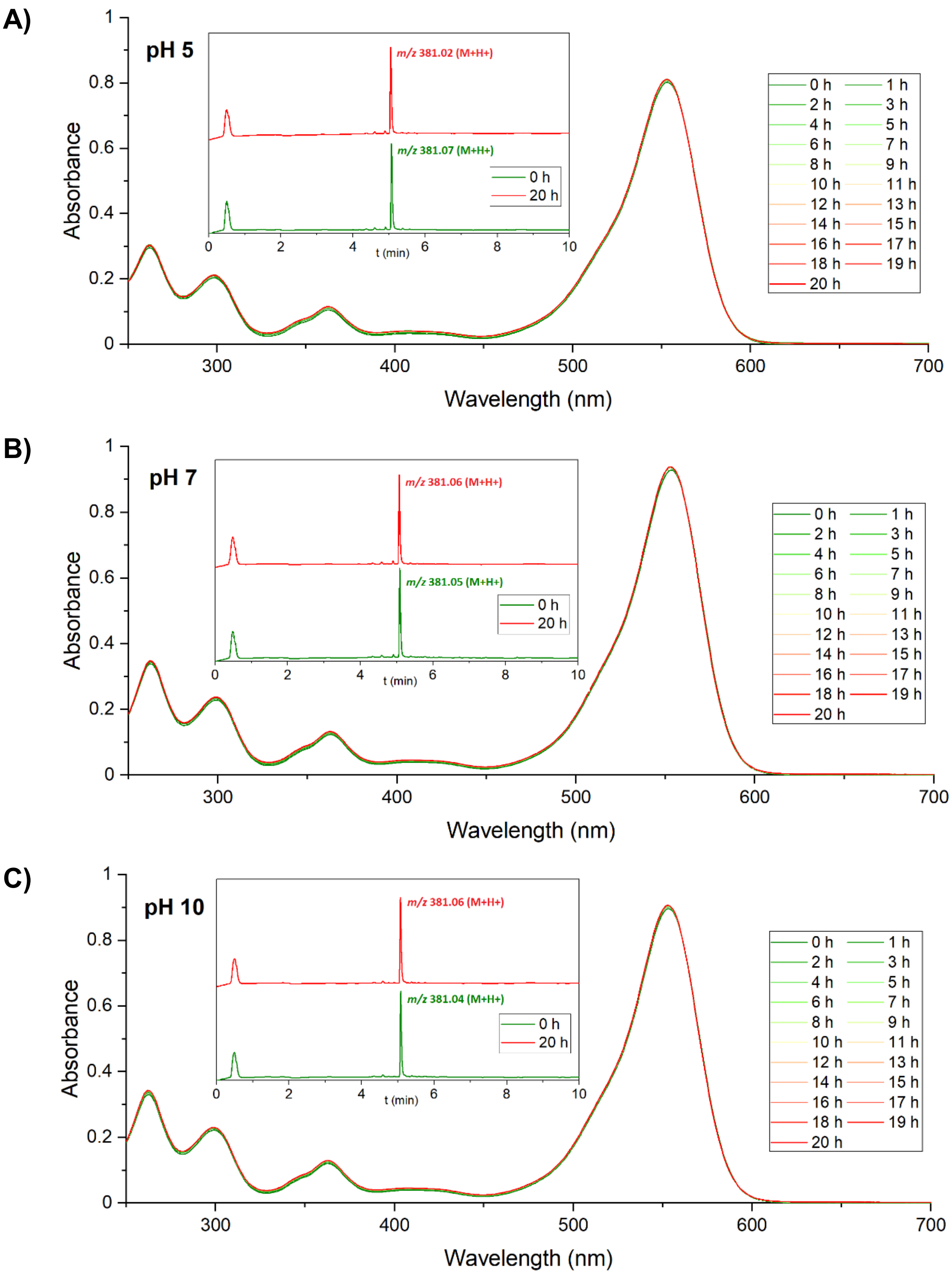

Figure S7. Absorption spectra of *N,N'*-dicyanorhodamine (6a) solutions in air-saturated 0.1 M phosphate buffer (+20% v/v DMSO) at pH 5 (A), 7 (B) and 10 (C) recorded over 20 h. The insets show the corresponding HPLC traces (absorption at 254 nm) at 0 h and 20 h with ESI-MS data for the molecular ion. Minor absorbance drift is likely due to slow evaporation of the solvent.

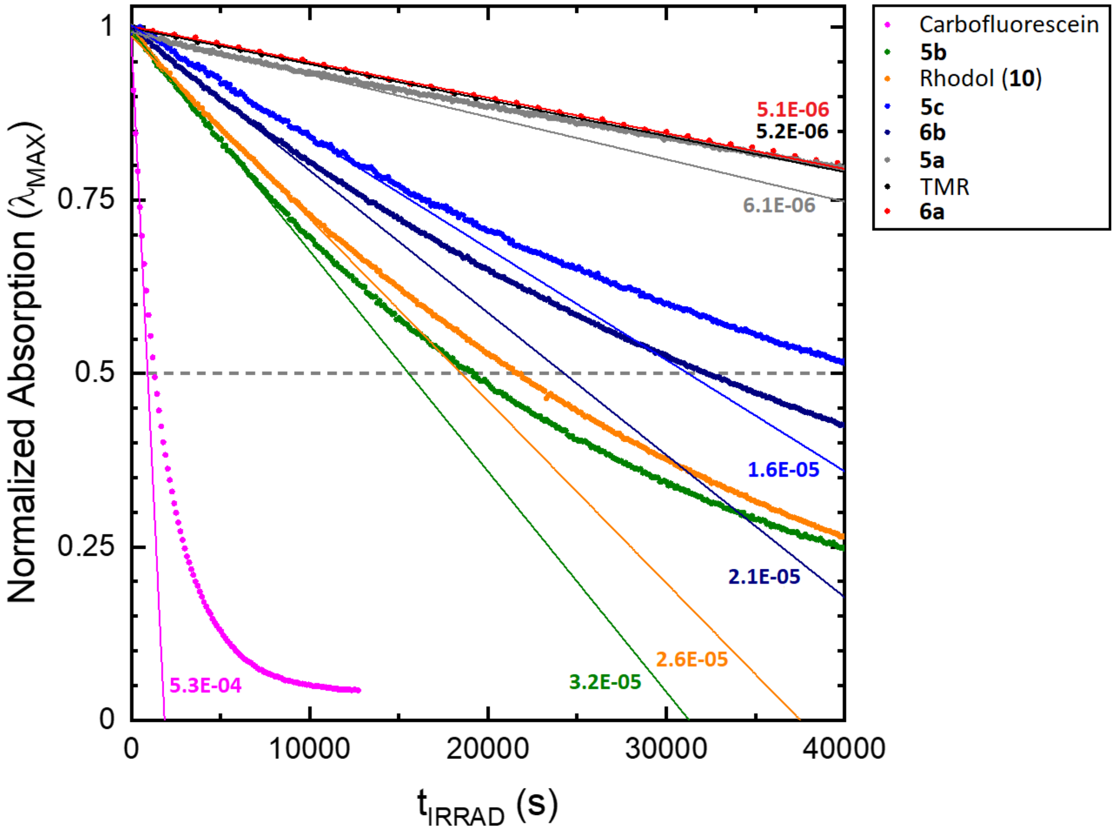

compound	ε_{530}, ×10^{-3} ($M^{-1}cm^{-1}$)	-slope, normalized (s^{-1})	ε_{530}×Φ_{BLEACH} ($M^{-1}cm^{-1}$)	Φ_{BLEACH}, ×10^{-6}
Carbofluorescein	52	5.3E-04	1.8	35
**5b**	32	3.2E-05	0.82	2.5
N,N-Dimethylrhodol (**10**)	41	2.6E-05	0.77	1.9
**5c**	36	1.6E-05	0.43	1.2
**6b**	58	2.1E-05	0.58	0.99
**5a**	28	6.1E-06	0.18	0.63
TMR	48	5.2E-06	0.14	0.3
**6a**	93	5.1E-06	0.15	0.16

Figure S8. Initial photobleaching rates and photobleaching quantum yields of *N*-cyanorhodamines (5a-c), *N,N'*-dicyanorhodamines (6a,b) and reference dyes in borate buffer (pH 9.9). ε_{MAX} – extinction coefficient at absorption λ_{max}, ε_{530} – extinction coefficient at the excitation wavelength (530 nm), Φ_{BLEACH} – photobleaching quantum yield. The dyes are listed in the order of increasing photostability (from high to low Φ_{BLEACH}).

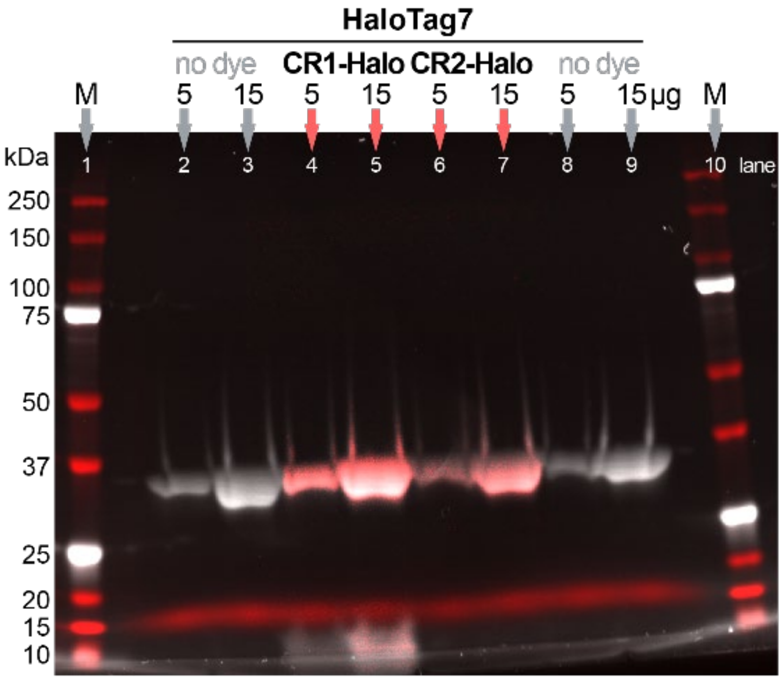

Figure S9. SDS PAGE of HaloTag7 labeled with CR1-Halo and CR2-Halo. The unlabeled HaloTag7 was added to lanes 2 and 8 at low amounts (5 µg) and to lanes 3 and 9 at high amounts (15 µg). **CR1-Halo**-labeled/**CR2-Halo**-labeled HaloTag7 protein was added to lanes 4/6 at low amounts (5 µg) and to lanes 5/7 at high amounts (15 µg). The marker (M) was added to lanes 1 and 10. Protein content was visualized via trihalo-enhanced tryptophan fluorescence (grey). **CR1-Halo** and **CR2-Halo** were detected via their own fluorescence (red). The observed protein bands match the expected size of HaloTag7 (35 kDa) and confirm specific labeling of HaloTag7 with **CR1-Halo** and **CR2-Halo**.

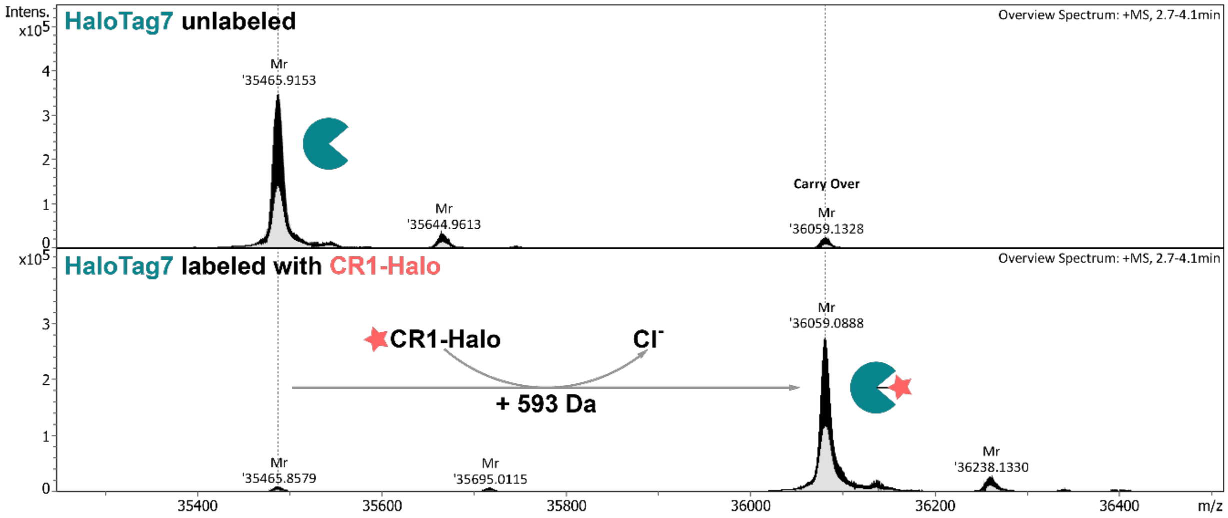

Figure S10. Mass spectrometry of HaloTag7 labeled with CR1-Halo. Comparison of the electrospray ionization mass spectra of unlabeled HaloTag7 (top) and HaloTag7 labeled with **CR1-Halo** (bottom) confirm the expected mass increase of 593 Da upon covalent binding of the dye and release of chloride. The molecular masses are indicated.

CR1-Halo (5 nM)

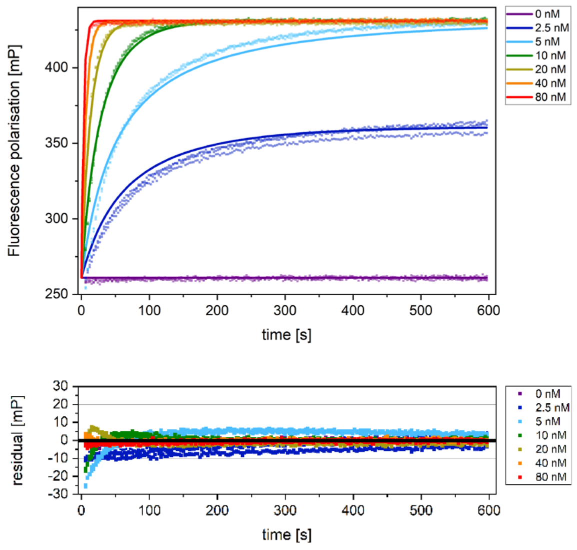

CR2-Halo (5 nM)

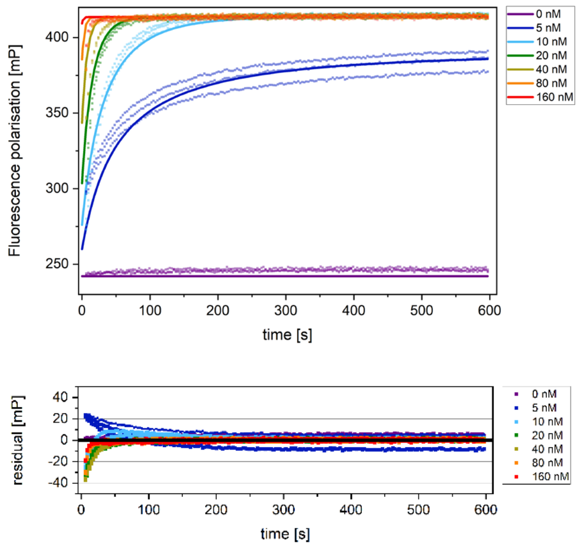

CR3-Halo (5 nM)

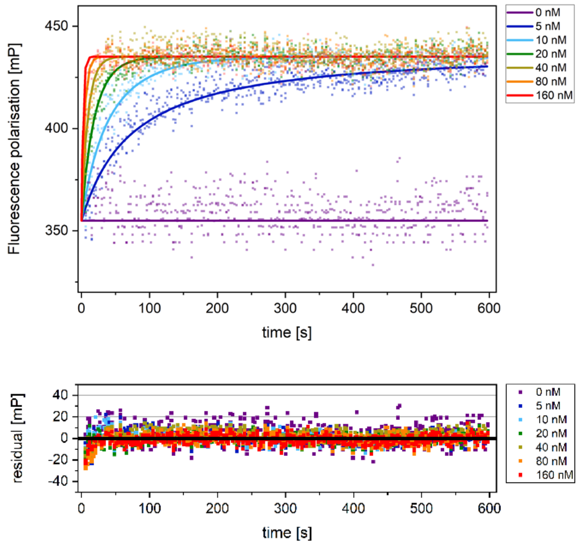

Fluorescein-Halo (5 nM)

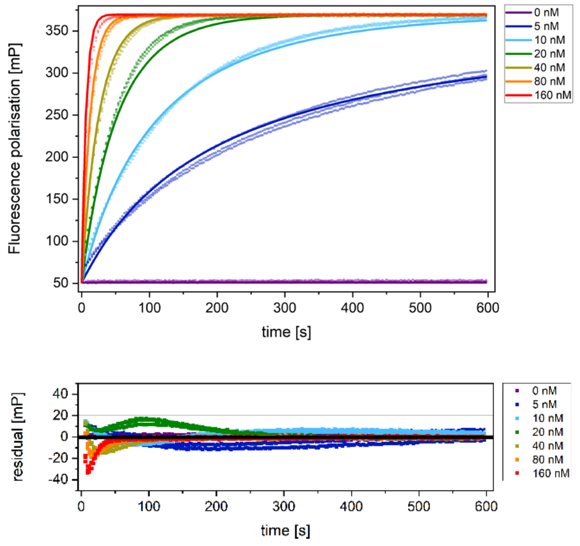

(continued)

TMR-Halo (1 nM)

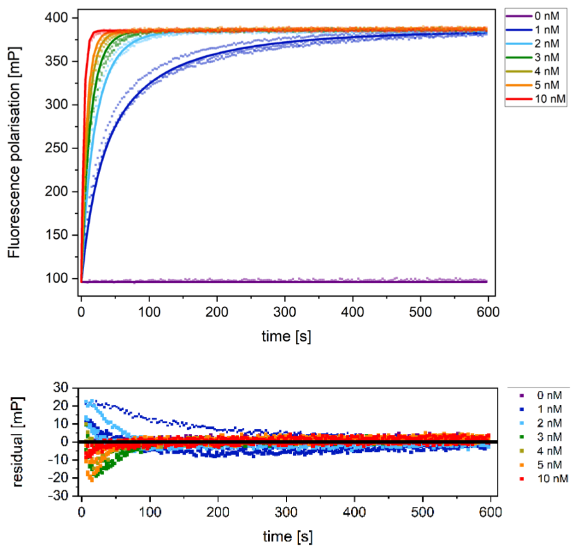

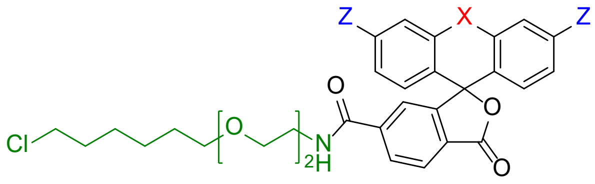

CR1-Halo: X = O, Z = NHCN
CR2-Halo: X = CMe_2, Z = NHCN
CR3-Halo: X = $SiMe_2$, Z = NHCN
Fluorescein-Halo: X = O, Z = OH
TMR-Halo: X = O, Z = NMe_2

Figure S11. Labeling kinetics of HaloTag7 with fluorescent ω-chloroalkane ligands. Top panels: Full fluorescence polarization traces (dots) and fitted values (lines) based on an irreversible second-order reaction model; bottom panels: residuals from the fits. All experiments were performed at 25 °C at a fixed fluorescent substrate concentration (5 nM, except for **TMR-Halo**: 1 nM) with varying HaloTag7 protein concentrations (as indicated).

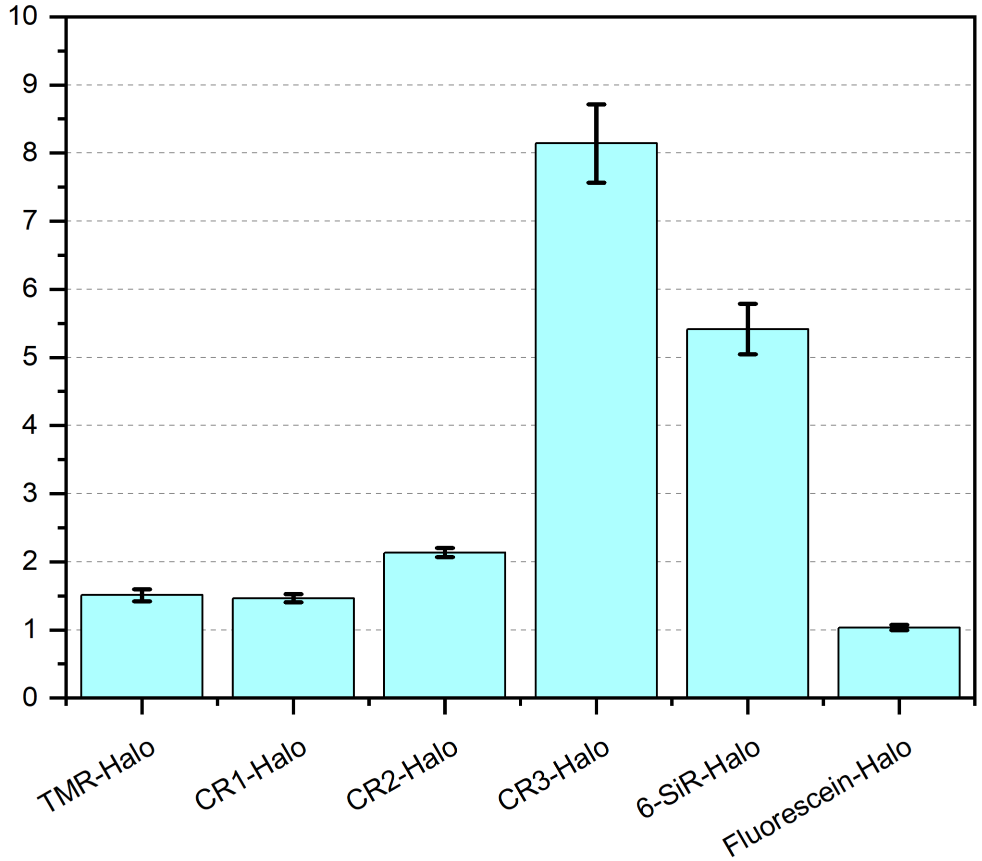

Figure S12. Fluorescence intensity changes upon binding to HaloTag7 protein. The bar length shows *x*-fold increase in fluorescence intensity upon reaction with excess HaloTag7 for 2 h at 37 °C (ratio protein:ligand = 2:1).

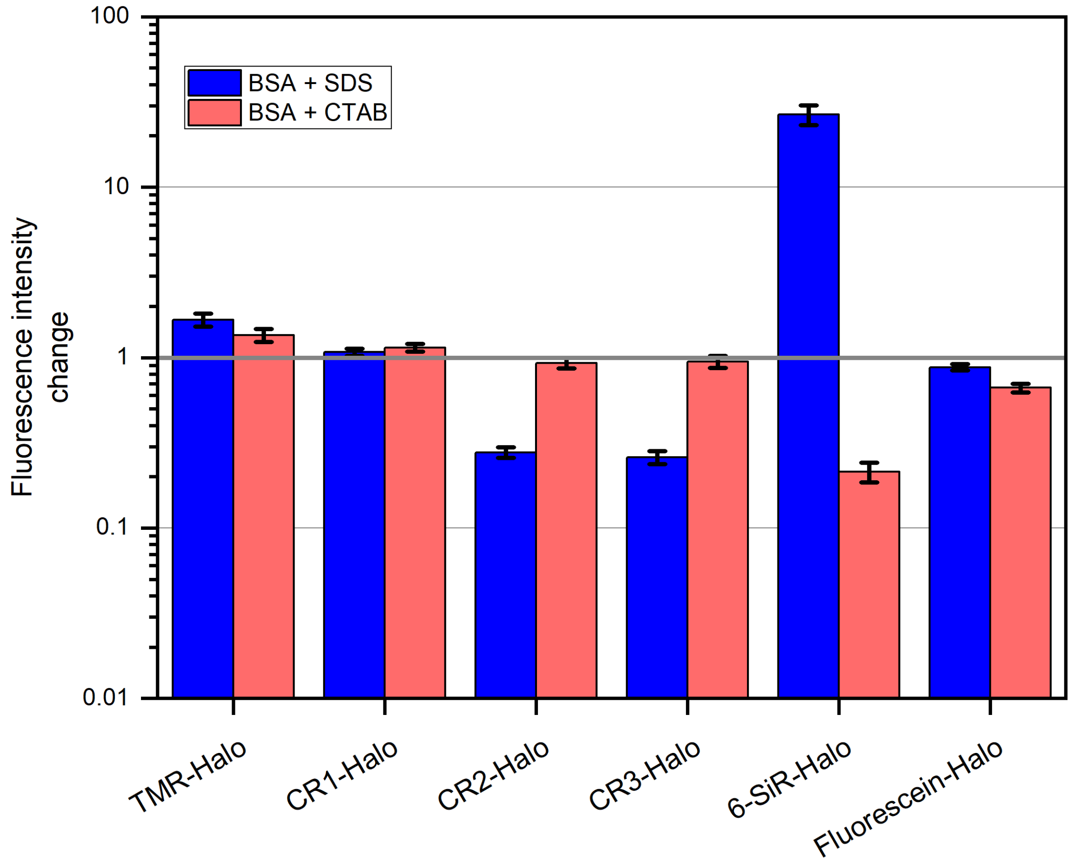

Figure S13. Fluorescence intensity changes upon addition of surfactants (SDS or CTAB) to the solutions of HaloTag ligands containing 0.1 mg/mL BSA. Blue bars: 0.1 mg/mL bovine serum albumin (BSA) in PBS (pH 7.4) + 0.5% sodium dodecyl sulfate (SDS); red bars: 0.1 mg/mL BSA in PBS (pH 7.4) + 0.5% cetyltrimethylammonium bromide (CTAB). The bar length shows the change (*x*-fold) in fluorescence intensity upon addition of a surfactant to the solution containing 0.1 mg/mL BSA in PBS only.

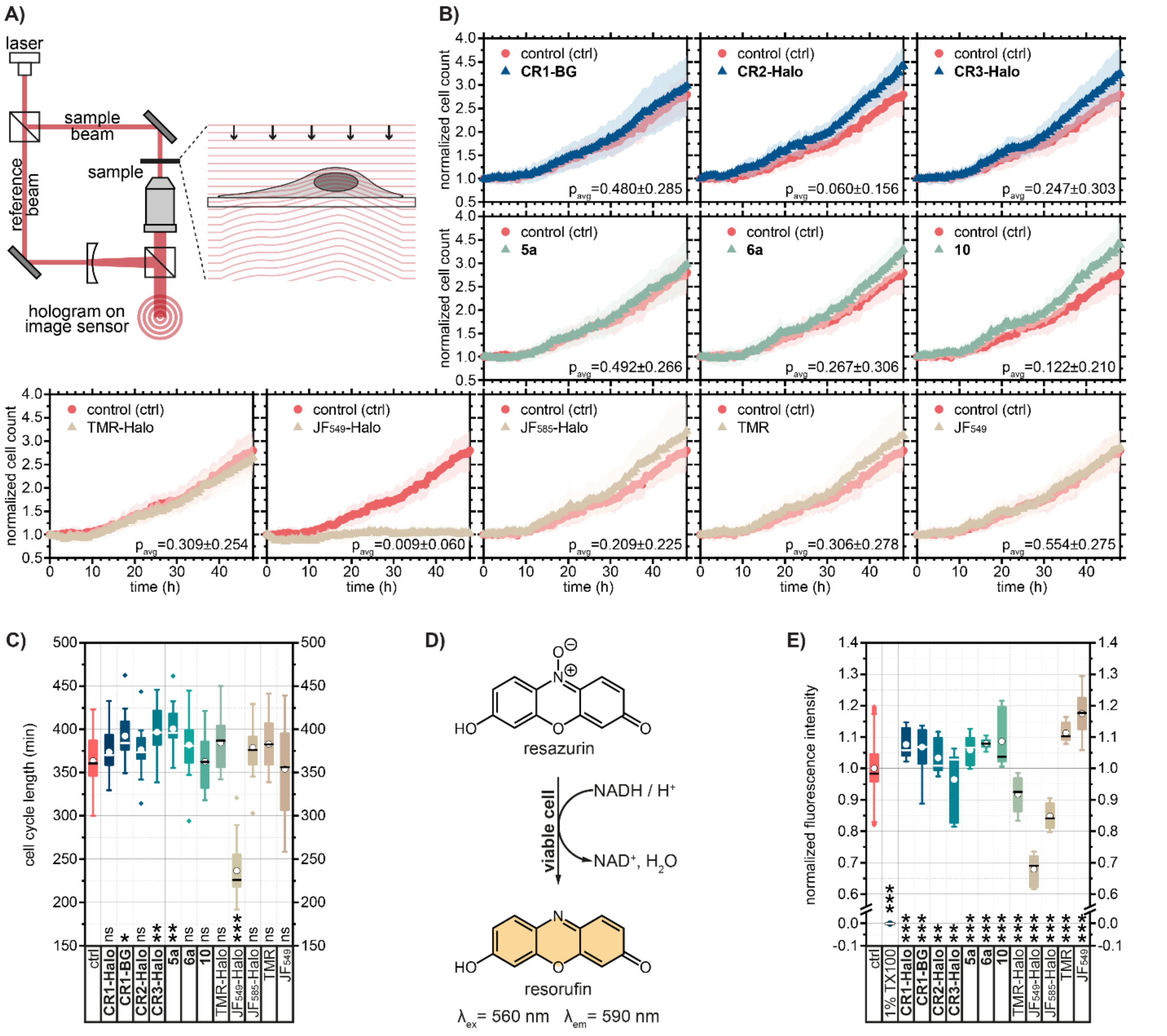

Figure S14. Assessment of cytotoxicity. (A) Schematic depiction of digital holographic microscopy. A cell delays the phase of the sample beam due to an increased refractive index as compared to its surrounding medium. Its phase image is computed from the hologram created by interference of the phase-shifted sample beam and the reference beam. (B) Normalized U-2 OS cell count and (C) U-2 OS cell cycle length as followed by holographic time-lapse imaging cytometry over 48 h in presence of 5 µM compound. (D) The CellTiter-Blue cell viability assay is based on the ability of viable cells to reduce the non-fluorescent redox indicator resazurin to the fluorescent dye resorufin. As non-viable cells rapidly lose this metabolic capacity, the fluorescent signal is directly proportional to the number of living cells. (E) CellTiter-Blue cell viability assay with 1% Triton X-100 (TX100) as positive control (U-2 OS cells, over 24 h, all compounds at 5 µM). Statistical significance: ns – no significant difference to control, *p<0.05, **p<0.005, ***p<0.0005; detailed information is provided in Table S3 and S4.

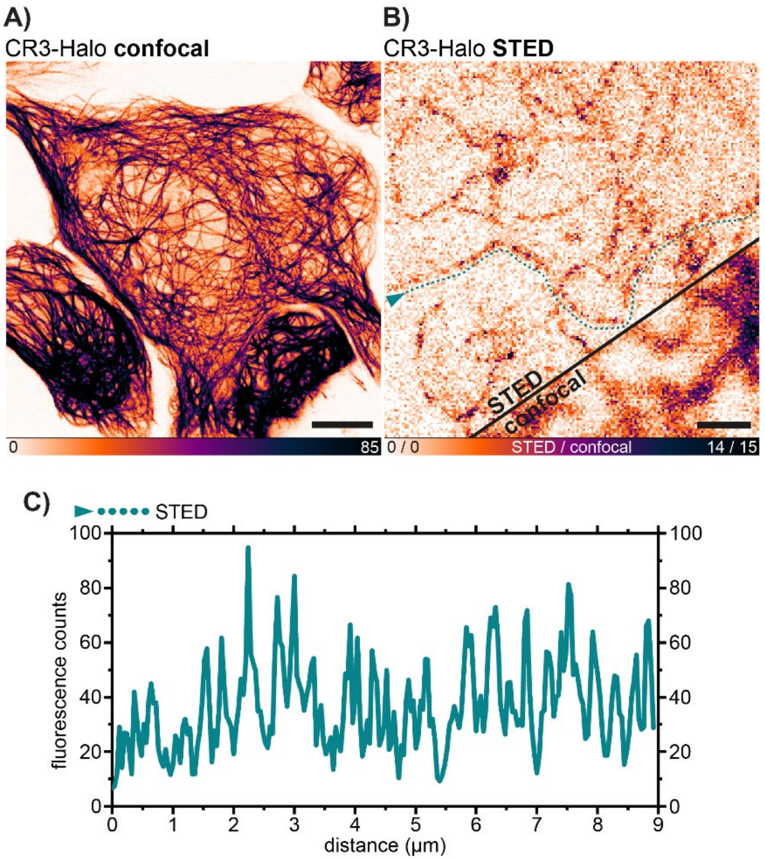

Figure S15. CR3-Halo confocal and STED imaging. (A) Exemplary confocal overview image and (B) comparison of a STED and confocal zoom-in of the vimentin network in U-2 OS Vim-Halo cells labeled with 5 µM **CR3-Halo**. (C) Line profile along the vimentin filament highlighted in (B) to illustrate the spotty **CR3-Halo** STED signal due to many molecules residing in the non-fluorescent form under live cell imaging conditions.

Supplementary Tables

Table S1. Ullmann amination of 3',6'-diiodofluoran (4a) with cyanamide: screening of the reaction conditions.

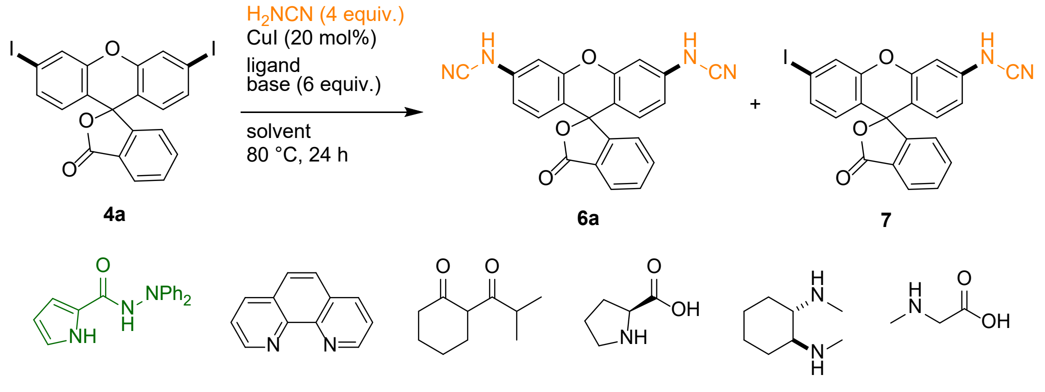

entry	ligand	base	solvent	HPLC yield
1	–	Cs_2CO_3	DMSO / DEG (10:1)	22%
2	**L1** (20 mol%)	Cs_2CO_3	DMSO	70%
3	**L1** (20 mol%)	K_3PO_4	EGME	81%
4	**L1** (30 mol%)	K_3PO_4	DEG	53%
5	**L2** (50 mol%)	K_3PO_4	DMSO	9%[a]
6	**L2** (40 mol%)	Cs_2CO_3	DMSO	20%[a,b]
7	**L2** (50 mol%)	Cs_2CO_3	DMSO	28%
8	**L3** (50 mol%)	Cs_2CO_3	DMSO	49%
9	**L4** (50 mol%)	Cs_2CO_3	DMSO	64%[c]
10	**L4** (50 mol%)	Cs_2CO_3	DMSO	49%[c,d]
11	**L5** (40 mol%)	Cs_2CO_3	DMSO	0%
12	**L6** (50 mol%)	Cs_2CO_3	DMSO	14%

Reaction conditions: **4a** (0.1 mmol), H_2NCN (0.4 mmol, 4 equiv.), CuI (0.02 mmol, 20 mol%), ligand, base (6 equiv.) and 4,4'-di(*tert*-butyl)biphenyl as an internal standard (0.5 equiv.) were stirred in 1.0 mL of a degassed solvent (0.1 M) at 80 °C; reaction time 24 h. [a] Multiple side products formed; [b] with 10 equiv. H_2NCN; [c] *N*-arylation of the ligand was observed; [d] with 50 mol% CuI. DEG = diethylene glycol, EGME = ethylene glycol monomethyl ether.

Table S2. Imaging parameters used for the acquisition of live cell STED and confocal microscopy data.

Fig.	dye	excitation (wavelength, power)	STED (wavelength, power)	delay [ns]	gate [ns]	detection window [nm]	pixel size [nm]	dwell time [µs]	line repetition	multiplexing
**6A**	**6a**	561nm, 62µW	---	---	---	570-640	80	10	1	frame
**6A**	TMR	561nm, 62µW	---	---	---	570-640	80	10	1	frame
**6A**	Hoechst	405nm, 1µW	---	---	---	420-475	80	10	2	frame
**6B**	**CR1-Halo**	561nm, 62µW	---	---	---	570-640	80	10	1	frame
**6B**	TMR-Halo	561nm, 62µW	---	---	---	570-640	80	10	1	frame
**6B**	Hoechst	405nm, 1µW	---	---	---	420-475	80	10	2	frame
**6C,D**	TMR-Halo	561nm, 62µW	---	---	---	570-640	80	10	1	frame
**6C,D**	ER-Tracker	405nm, 50µW	---	---	---	420-475	80	10	2	frame
**7A**	**CR1-Halo**	561nm, 7µW	---	---	---	575-630	80	5	4	pixel
**7A**	SiR-Hoechst	640nm, 13µW	---	---	---	655-757	80	5	4	pixel
**7A**	LIVE 510	485nm, 33µW	---	---	---	500-545	80	5	4	frame
**7A**	ER-Tracker	405nm, 92µW	---	---	---	410-700	80	5	4	frame
**7B**	**CR1-Halo**	561nm, 11µW	775nm, 368mW	0.75	8	570-630	25	3	40	line
**7B**	**CR1-Halo**	561nm, 18µW	---	---	---	570-630	25	5	4	pixel
**7C**	SiR-Hoechst	640nm, 9µW	775nm, 220mW	0.75	8	650-757	25	3	40	line
**7C**	SiR-Hoechst	640nm, 3µW	---	---	---	650-757	25	5	4	pixel
**7D**	**CR2-Halo**	640nm, 37µW	775nm, 146mW	0.75	8	650-757	25	20	4	line
**7D**	LIVE 550	561nm, 17µW	775nm, 368mW	0.75	8	575-645	25	20	4	line

Fig.	dye	excitation (wavelength, power)	STED (wavelength, power)	delay [ns]	gate [ns]	detection window [nm]	pixel size [nm]	dwell time [µs]	line repetition	multiplexing
**7D**	Hoechst	405nm, 5µW	---	---	---	420-475	25	10	2	frame
**7E**	**CR2-Halo**	640nm, 37µW	775nm, 146mW	0.75	8	650-757	25	20	4	line
**7E**	**CR2-Halo**	640nm, 33µW	---	---	---	650-757	25	10	1	pixel
**7F**	LIVE 550	561nm, 17µW	775nm, 368mW	0.75	8	575-645	25	20	4	line
**7F**	LIVE 550	561nm, 15µW	---	---	---	575-645	25	10	1	pixel
**8A**	**CR1-BG**	561nm, 7µW	---	---	---	575-630	80	10	1	frame
**8A**	GeR-tubulin	640nm, 6µW	---	---	---	650-750	80	10	1	frame
**8A**	Mito-Tracker	485nm, 6µW	---	---	---	500-550	80	10	1	frame
**8A**	Hoechst	405nm, 1µW	---	---	---	420-475	80	10	1	frame
**8B**	**CR1-BG**	561nm, 23µW	775nm, 750mW	1	10	570-630	25	3	5	frame
**8B**	**CR1-BG**	561nm, 7µW	---	---	---	570-630	25	3	3	frame
**8C**	GeR-tubulin	640nm, 6µW	775nm, 146mW	0.85	10	650-757	25	3	20	line
**8C**	GeR-tubulin	640nm, 6µW	---	---	---	650-757	25	3	3	line
**S12A**	**CR3-Halo**	640nm, 33µW	---	---	---	650-757	80	10	1	---
**S12B**	**CR3-Halo**	640nm, 136µW	775nm, 73mW	0.75	8	650-757	40	10	8	line
**S12B**	**CR3-Halo**	640nm, 67µW	---	---	---	650-763	40	10	1	line

Laser powers are measured at the back focal aperture; delay – timing delay between excitation and STED laser pulses, gate – time of gated detection, multiplexing – pixel, line or frame multiplexing between different channels. Dye name abbreviations: Hoechst – Hoechst 33342, TMR-Halo – HaloTag TMR Ligand, ER-Tracker – ER-Tracker Blue-White DPX, LIVE 510 – abberior LIVE 510 tubulin, Mito-Tracker – MitoTracker Green FM, LIVE 550 – abberior LIVE 550 tubulin.

Table S3. Statistical information on CellTiter-Blue cell viability assay.

Fig.	condition	concentration	N_{exp}	normalized fluorescence intensity		p-value
				mean	SD	
5A	ctrl	---	630	1.000	0.091	---
	**5a**	1 µM	63	1.081	0.039	8×10^{-12}
	**5a**	5 µM	63	1.058	0.040	6×10^{-7}
	**5a**	10 µM	63	1.091	0.017	1×10^{-14}
	**5a**	33 µM	63	1.066	0.056	3×10^{-8}
	**5a**	100 µM	63	0.890	0.023	2×10^{-20}
	**5a**	333 µM	63	0.150	0.019	$<10^{-308}$
	**6a**	1 µM	63	0.992	0.077	5×10^{-1}
	**6a**	5 µM	63	1.079	0.014	2×10^{-11}
	**6a**	10 µM	63	1.008	0.048	5×10^{-1}
	**6a**	33 µM	63	1.045	0.102	2×10^{-4}
	**6a**	100 µM	63	0.932	0.048	8×10^{-9}
	**6a**	333 µM	63	0.095	0.013	$<10^{-308}$
	TMR	1 µM	63	1.048	0.052	5×10^{-5}
	TMR	5 µM	63	1.113	0.028	4×10^{-21}
	TMR	10 µM	63	1.149	0.030	4×10^{-34}
	TMR	33 µM	63	1.143	0.048	3×10^{-31}
	TMR	100 µM	63	0.830	0.025	6×10^{-43}
	TMR	333 µM	63	0.119	0.024	$<10^{-308}$
	DMSO	0.02%	63	0.995	0.039	6×10^{-1}
	DMSO	0.2%	63	1.004	0.057	8×10^{-1}
	DMSO	0.66%	63	1.075	0.022	2×10^{-10}
	DMSO	2.00%	63	0.816	0.021	4×10^{-49}
	DMSO	6.60%	63	0.087	0.005	$<1\times10^{-308}$
	DMSO	20.0%	63	0.000	0.001	$<1\times10^{-308}$
S11E	ctrl	---	504	1.000	0.084	---
	TX100	1.00%	63	0.000	0.001	$<1\times10^{-308}$
	**CR1-Halo**	5 µM	63	1.076	0.042	7×10^{-12}
	**CR1-BG**	5 µM	63	1.068	0.051	1×10^{-9}
	**CR2-Halo**	5 µM	63	1.033	0.050	2×10^{-3}
	**CR3-Halo**	5 µM	63	0.964	0.100	2×10^{-3}
	**5a**	5 µM	63	1.058	0.040	9×10^{-8}
	**6a**	5 µM	63	1.079	0.014	5×10^{-13}
	**10**	5 µM	63	1.086	0.081	1×10^{-13}
	TMR-Halo	5 µM	63	1.030	0.061	2×10^{-13}
	JF_{549}-Halo	5 µM	63	0.984	0.031	3×10^{-117}
	JF_{585}-Halo	5 µM	63	0.918	0.050	7×10^{-39}
	TMR	5 µM	63	0.679	0.044	1×10^{-23}
	JF_{549}	5 µM	63	0.848	0.037	8×10^{-48}

N_{exp} – number of experiments, SD – standard deviation, ctrl – control, TX100 – Triton X-100; p-values state significance of difference to control.

Table S4. Statistical information on holographic time-lapse imaging.

Fig.	condition	concentration	N_{exp}	N_{cells}	measured parameter		p-value
					mean	SD	
5B	ctrl	---	19	22713	0.359	0.018	---
	**CR1-Halo**	5 µM	15	18196	0.365	0.02	4×10^{-1}
	**CR1-BG**	5 µM	18	23642	0.373	0.01	1×10^{-2}
	**CR2-Halo**	5 µM	19	23117	0.368	0.014	1×10^{-1}
	**CR3-Halo**	5 µM	19	23790	0.373	0.009	8×10^{-3}
	**5a**	5 µM	18	23882	0.372	0.01	1×10^{-2}
	**6a**	5 µM	18	20932	0.371	0.013	3×10^{-2}
	**10**	5 µM	16	17873	0.365	0.029	5×10^{-1}
	TMR-Halo	5 µM	20	23532	0.369	0.01	5×10^{-2}
	JF_{549}-Halo	5 µM	18	11254	0.305	0.046	5×10^{-5}
	JF_{585}-Halo	5 µM	19	24520	0.373	0.007	5×10^{-3}
	TMR	5 µM	17	21461	0.372	0.012	2×10^{-2}
	JF_{549}	5 µM	19	20955	0.367	0.013	1×10^{-1}
5C	ctrl	---	19	22713	2.80	0.43	---
	**CR1-Halo**	5 µM	15	18196	3.34	0.62	0.297±0.289
S11B	ctrl	---	19	22713	2.80	0.43	---
	**CR1-BG**	5 µM	18	23642	2.99	0.65	0.480±0.285
	**CR2-Halo**	5 µM	19	23117	3.42	0.37	0.060±0.156
	**CR3-Halo**	5 µM	19	23790	3.26	0.59	0.247±0.303
	**5a**	5 µM	18	23882	2.97	0.52	0.492±0.266
	**6a**	5 µM	18	20932	3.30	0.44	0.267±0.306
	**10**	5 µM	16	17873	3.40	0.46	0.122±0.210
	TMR-Halo	5 µM	20	23532	2.65	0.45	0.309±0.254
	JF_{549}-Halo	5 µM	18	11254	1.02	0.17	0.009±0.060
	JF_{585}-Halo	5 µM	19	24520	3.22	0.75	0.209±0.225
	TMR	5 µM	17	21461	3.13	0.67	0.306±0.278
	JF_{549}	5 µM	19	20955	2.86	0.38	0.554±0.275
S11C	ctrl	---	19	22713	364	32	---
	**CR1-Halo**	5 µM	15	18196	374	26	4×10^{-1}
	**CR1-BG**	5 µM	18	23642	392	26	6×10^{-3}
	**CR2-Halo**	5 µM	19	23117	376	26	2×10^{-1}
	**CR3-Halo**	5 µM	19	23790	397	28	2×10^{-3}
	**5a**	5 µM	18	23882	401	26	7×10^{-4}
	**6a**	5 µM	18	20932	382	36	1×10^{-1}
	**10**	5 µM	16	17873	363	34	9×10^{-1}
	TMR-Halo	5 µM	20	23532	384	30	5×10^{-2}
	JF_{549}-Halo	5 µM	18	11254	237	32	1×10^{-13}
	JF_{585}-Halo	5 µM	19	24520	379	30	1×10^{-1}
	TMR	5 µM	17	21461	383	27	7×10^{-2}
	JF_{549}	5 µM	19	20955	354	48	5×10^{-1}

N_{exp} – number of experiments, N_{cells} – number of analyzed cells, SD – standard deviation, ctrl – control, TX100 – Triton X-100; measured parameter: 5B – cell division frequency in divisions per cell, 5C/S11B: increase of cell count after 48 h in fold change, S11: cell cycle length in minutes; p-values state significance of difference to control; for 5C/S11B, p-value averages of all time points are given.

Supplementary Methods

General experimental information

Thin layer chromatography

Analytical TLC (normal phase) was performed on Merck Millipore ready-to-use aluminum sheets coated with silica gel 60 (F_{254}) (Cat. No. 1.05554.0001). Analytical TLC on reversed phase (RP-C_{18}) was performed on Merck Millipore ready-to-use aluminum sheets coated with RP-18 60 (F_{254}s) (Cat. No. 1.05560.0001). Compounds were detected by exposing TLC plates to UV-light (254 or 366 nm) or by heating with vanillin stain (6 g vanillin and 1.5 mL conc. H_2SO_4 in 100 mL ethanol), PMA stain (10 g of phosphomolybdic acid hydrate in 100 mL ethanol), $KMnO_4$ stain (600 mg of $KMnO_4$, 4 g of K_2CO_3, 1.4 mL of 1 N NaOH in 80 mL water), 0.1 N NaOH (on mild heating, hydrolyzes fluorescein triflates but not iodofluoranes), 1 N NaOH or 1 N HCl as indicated.

Preparative flash column chromatography

Automated separations on normal phase were performed with an Isolera Spektra One system (Biotage AG, Sweden) using commercially available cartridges of suitable size (RediSep Rf series from Teledyne ISCO, Puriflash Silica HP 30µm series from Interchim) and solvent gradient indicated.

High-Performance Liquid Chromatography (HPLC) and Mass Spectrometry (MS)

Analytical liquid chromatography-mass spectrometry was performed on an LC-MS system (Shimadzu): 2x LC-20AD HPLC pumps with DGU-20A3R solvent degassing unit, SIL-20ACHT autosampler, CTO-20AC column oven, SPD-M30A diode array detector and CBM-20A communication bus module, integrated with CAMAG TLC-MS interface 2, FCV-20AH_2 diverter valve and LCMS-2020 spectrometer with electrospray ionization (ESI, 100 – 1500 m/z). Analytical column: ThermoScientific Hypersil Gold 50×2.1 mm 1.9µm, standard conditions: sample volume 1-2 µL, solvent flow rate 0.5 mL/min, column temperature 30 °C. General method: isocratic 95:5 A:B over 2 min, then gradient 95:5 to

0:100 A:B over 5 min, then isocratic 0:100 A:B over 2 min; solvent A – water + 0.1% (v/v) HCO_2H, solvent B – acetonitrile + 0.1% (v/v) HCO_2H.

High resolution mass spectra (HRMS) were obtained on a maXis II ETD (Bruker) with electrospray ionization (ESI) at the Mass Spectrometry Core facility of the Max-Planck Institute for Medical Research (Heidelberg, Germany).

Preparative high-performance liquid chromatography was performed on a Büchi Reveleris Prep system using the suitable preparative columns and conditions as indicated for individual preparations. Method scouting was performed on a HPLC system (Shimadzu): 2x LC-20AD HPLC pumps with DGU-20A3R solvent degassing unit, CTO-20AC column oven equipped with a manual injector with a 20 µL sample loop, SPD-M20A diode array detector, RF-20A fluorescence detector and CBM-20A communication bus module; or on a Dionex Ultimate 3000 UPLC system: LPG-3400SD pump, WPS-3000SL autosampler, TCC-3000SD column compartment with 2× 7-port 6-position valves and DAD-3000RS diode array detector. The test runs were performed on analytical columns with matching phases (HPLC: Interchim 250×4.6 mm 5 µm PhC4, solvent flow rate 1.2 mL/min; UPLC: Interchim PhC4 75×2.1 mm 2.2 µm, ThermoScientific Hypersil Gold 100×2.1 mm 1.9 µm, solvent flow rate 0.5 mL/min).

NMR spectra

NMR spectra were recorded at 25 °C with a Bruker Ascend 400 spectrometer at 400.15 MHz (^{1}H), 376.52 MHz (^{19}F) and 100.62 MHz (^{13}C) and are reported in ppm. All ^{1}H spectra are referenced to tetramethylsilane as an internal standard (0.03% v/v; δ = 0.00 ppm) or the residual protons of DMSO-d_5 (2.50 ppm) for DMSO-d_6. ^{13}C spectra are referenced to tetramethylsilane (δ = 0 ppm) using the signals of tetramethylsilane as an internal standard (0.03% v/v) or the solvent: $CDCl_3$ (77.16 ppm), CD_3OD (49.00 ppm), DMSO-d_6 (39.52 ppm) or pyridine-d_5 (150.35 ppm, C-2,6). Multiplicities of the signals are described as follows: s = singlet, d = doublet, t = triplet, q = quartet, m = multiplet or overlap of non-equivalent resonances; br = broad signal. Coupling constants $^{n}J_{X\text{-}Y}$ are given in Hz, where n is the number of bonds between the coupled nuclei X and Y ($J_{H\text{-}H}$ are always listed as

J without indices). For the ^{13}C chemical shifts obtained by indirect detection from HSQC experiments (minimum resolution in F1: t1≥192), only H-coupled C-nuclei are resolved.

Optical spectroscopy

Absorption spectra were recorded with a Varian Cary 5000 UV-Vi-NIRs double-beam spectrophotometer (Agilent Technologies, USA). The emission spectra were recorded with a Varian Cary Eclipse fluorescence spectrophotometer (Agilent Technologies). The absorption and emission spectra were recorded in quartz cells (optical path length 1 cm). Fluorescence quantum yields (absolute method determinations) were obtained with a Quantaurus-QY absolute PL quantum yield spectrometer (model C11347-11, Hamamatsu) according to the manufacturer's instructions. All measurements were performed in air-saturated solvents at ambient temperature. Fluorescence lifetimes were measured with a FluoTime 300 fluorescence lifetime spectrometer FluoTime 300 (PicoQuant) using a picosecond pulsed diode laser LDH-P-C-470, LDH-D-TA-560 or LDH-P-C-640B (PicoQuant) and the manufacturer's EasyTau 2 fitting/analysis software, in air-saturated solvents thermostated at 25 °C.

Series of absorption and fluorescence emission spectra were recorded in triplicate with a CLARIOstar Plus microplate reader (BMG LABTECH GmbH, Germany) in 96-well microplates (200 µL/well): non-binding polystyrene F-bottom, µClear (Greiner Bio-One GmbH, Ref. 655906) for aqueous solutions, polypropylene F-bottom (Greiner Bio-One GmbH, Ref. 655201) for dioxane-water mixtures. The spectra were recorded at 25 °C in air-saturated solvents and are background corrected.

For determination of pK_a values, 10 µM solutions of dyes in 0.1M sodium phosphate buffers were transferred to a 96-well plate (200 µL per well), and absorption and emission spectra were recorded in triplicates. pK_a values were determined by plotting the pH *vs.* the normalized absorbance at a wavelength λ_{max} (corresponding to the dye absorption maximum in the visible range) and the data were fitted to equation (1).

$$abs_{norm} = \frac{1}{1 + 10^{\mathrm{n(pH-pK_a)}}} \qquad (1)$$

where abs_{norm} is the normalized absorbance at λ_{max}, n – Hill coefficient (a fitted parameter).

For determination of $D_{0.5}$ values, 10 µM solutions of dyes in aqueous dioxane (0%, 10%, 20%, 30%, 40%, 50%, 60%, 70%, 80%, 90%, 95% and 100% water content) were transferred to a 96-well plate (200 µL per well), and absorption and emission spectra were recorded in triplicates. $D_{0.5}$ values were determined by plotting the water content (% v/v) *vs.* the normalized absorbance at a wavelength λ_{max} (corresponding to the dye absorption maximum in the visible range) and the data were fitted to equation (2).

$$abs_{norm} = \frac{1}{1 + 10^{\mathrm{n}(\mathrm{D}-D_{0.5})}} \qquad (2)$$

where abs_{norm} is the normalized absorbance at λ_{max}, n – Hill coefficient (a fitted parameter), D – dielectric constant of the corresponding dioxane-water mixture [S3].

Chemometric analysis of the photobleaching reaction kinetics

Solutions of the dyes in air-saturated borate buffer (100 mM, pH 9.9) were irradiated in a previously described [S4] home-built setup with a 530 nm LED source (ML530L3, Thorlabs Inc.). During the irradiation, samples were maintained at 20 °C and continuously stirred with a Peltier-based temperature-controlled cuvette holder (Luma 40, Quantum Northwest, Inc.). The absorption and emission of irradiated solutions was monitored at desired irradiation intervals with a fiber-based spectrometer (Flame-S-UV-Vis-ES, Ocean Insight). For absorption measurements, a deuterium and tungsten halogen source was used for illumination (DH-2000-BAL, Ocean Insight), and for fluorescence excitation was performed in a 90° configuration with a short pulse (<10 ms) of the same LED. Data collection and analysis was performed with custom-made routines in Matlab R2020a (MathWorks).

The photobleaching quantum yield (Φ_{BLEACH}, see Figure S7) was calculated from the following expression (equation (3) [S5]):

$$-\frac{\Delta[dye]}{\Delta t} \approx -\frac{d[dye]}{dt} = \Phi_{BLEACH} \times I_0 \times \varepsilon_{530} \times F \times [dye]$$

$$-\frac{\Delta Abs_{MAX}/\varepsilon_{530}}{\Delta t} \approx \Phi_{BLEACH} \times I_0 \times \varepsilon_{530} \times F \times (Abs_{MAX}/\varepsilon_{530})_0$$

$$-\frac{\Delta Abs_{MAX}/(Abs_{MAX})_0}{\Delta t} = -slope \approx \Phi_{BLEACH} \times I_0 \times \varepsilon_{530} \times F \quad (3)$$

where *F* is the photokinetic factor at 530 nm and I_0 is the volumetric photon flux of irradiation light (previously measured with a chemical actinometer) in $mol_{photons}\cdot L^{-1}\cdot s^{-1}$.

HaloTag7 labeling specificity

To confirm labeling specificity of the *N,N'*-dicyanorhodamine HaloTag ligands, electrospray ionization mass spectrometry (ESI-MS) and sodium dodecyl sulfate polyacrylamide gel electrophoresis (SDS PAGE) were conducted with purified HaloTag7 protein. After incubating HaloTag7 (634 µM, 22.48 mg/mL) with **CR1-Halo** or **CR2-Halo** (1.1 equiv, 697 µM) in PBS pH 7.4 + 0.1% CHAPS at rt for 1 h, unlabeled and labeled protein was diluted to ~1 mg/mL in 13 mM ammonium acetate for ESI-MS, and to ~1 mg/mL and ~3 mg/mL in tris-glycine-SDS buffer (25 mM tris, 190 mM glycine, 0.1% SDS) for SDS PAGE. ESI-MS data were recorded on a maXis II ETD spectrometer (Bruker) at the Mass Spectrometry Core facility of the Max-Planck Institute for Medical Research (Heidelberg, Germany).

For SDS PAGE, samples were further diluted 1:4 in 4x Laemmli Sample Buffer (1610747, Bio-Rad) with 10% 2-mercaptoethanol, boiled at 95°C for 5 min and loaded (20 µL/well) onto a 10% Mini-PROTEAN TGX Stain-Free Precast Gel (4568035, Bio-Rad) in a Mini-PROTEAN Tetra Vertical Electrophoresis Cell (Bio-Rad). The Precision Plus Protein Dual Color Standards (1610374, Bio-Rad) was used as reference (3 µL/well). The gel was run for 5 min at 50 V and for another 45 min at 150 V. Fluorescence was measured with a UVP ChemStudio PLUS imager (Analytik Jena). **CR1-Halo** and **CR2-Halo** were excited at 510 nm with fluorescence detection at 605/50 nm. The HaloTag7 protein was visualized via trihalo compounds after a short UV photoactivation via UV excitation with fluorescence detection at 535/45 nm.

HaloTag7 labeling kinetics

Kinetic measurements by means of fluorescence polarization (FP) were performed on a CLARIOstar Plus microplate reader as described in [S6]. More specifically, a series of HaloTag7 protein solutions (0-320 nM) in PBS pH 7.4 + 0.1% CHAPS were prepared in standard polypropylene 1.5 mL Eppendorf tubes, pre-blocked with 1% BSA in PBS pH 7.4 for 1 h. A polystyrene black flat-bottom non-binding 96 well plate (Greiner Bio-One, part Nr. 655900) was loaded with a series of HaloTag7 protein solutions (100 µL per well), the injector was loaded with a 10 nM solution of the corresponding fluorescent ligand (2 nM in the case of TMR-Halo) in 0.1% CHAPS – PBS pH 7.4. Mixing was performed in a well-by-well readout mode (fast kinetics) by injecting (at 300 µL/s) 100 µL of the ligand solution and double orbital shaking for 3 s. Fluorescence polarization readout was started 6 s from the injection point at 3 s intervals for the total time of 10 min. The optic settings were set as per the following table:

Ligand	TMR-Halo	CR1-Halo	CR2-Halo	CR3-Halo	Fluorescein-Halo
Excitation filter	540-20	540-20	590-50	590-50	482-16
Dichroic mirror	LP 566	LP 566	LP 639	LP 639	LP 504
Emission filter	590-20	590-20	675-50	675-50	530-40
Gain	1500	1400	1200	1000	1000

Experiments were run in triplicates at 25 °C, and the resulting kinetic data were fitted to a simplified kinetic model (equation (4): apparent second-order reaction, irreversible binding) using DynaFit software (http://www.biokin.com/dynafit/) as described in [S7]:

$$P + S \xrightarrow{k_{app}} PS \qquad (4)$$

where P is the HaloTag protein, S is the fluorescent substrate and PS is the protein-substrate conjugate.

Fluorescence intensity changes of substrates in the presence of a HaloTag7 protein

Fluorescence intensity changes in the presence of HaloTag7 protein were determined using a modified procedure from [S8]. In 1.5 mL Eppendorf tubes, 10 µL of 25 µM stock solution of a HaloTag ligand in DMSO was added to 490 µL of:

1) 10 % FBS (HI, lot: 08F0597K) in DMEM (Gibco, 4.5 g/L D-glucose, L-glutamine, 25 mM HEPES, no sodium pyruvate) *or*
2) 1 µM HaloTag7 in 10 % FBS in DMEM (as above).

The resulting mixtures were incubated at 37 °C for 2 h and transferred (100 µL per well) to a GREINER 96 F-BOTTOM (black bottom, non-binding) microplate. Fluorescence intensities were measured in triplicates with a CLARIOstar Plus microplate reader (every 10 min over 1 h). Excitation and emission wavelengths were chosen as in the table below (excitation and emission bandwidths: 10 nm, gain: 1000):

**Ligand**	**TMR-Halo**	**CR1-Halo**	**CR2-Halo**	**CR3-Halo**	**6-SiR-Halo**	**Fluorescein-Halo**
Excitation wavelength [nm]	540	540	610	640	640	490
Emission wavelength [nm]	580	580	650	680	680	530

For data processing, the first 3 stable readings per sample (9 values per label) were averaged and the results were presented as fluorescence intensity ratios for solutions containing HaloTag protein/solutions containing FBS in DMEM only.

Fluorescence intensity changes of substrates in the presence of surfactants

Fluorescence intensity changes in the presence of surfactants were determined using a modified procedure from [S8]. In 1.5 mL Eppendorf tubes, 10 µL of 25 µM stock solution of a HaloTag ligand in DMSO was added to 490 µL of:

1) 0.1 mg/mL BSA in PBS (pH 7.4),
2) 0.1 mg/mL BSA in PBS (pH 7.4) containing 0.5% SDS (sodium dodecyl sulfate) *or*
3) 0.1 mg/mL BSA in PBS (pH 7.4) containing 0.5% CTAB (cetyltrimethylammonium bromide).

The resulting mixtures were incubates at 37 °C for 2 h and transferred (100 µL per well) to a GREINER 96 F-BOTTOM (black bottom, non-binding) microplate. Fluorescence intensities were measured in triplicates with a CLARIOstar Plus microplate reader by

recording the full emission spectra with the excitation wavelengths (bandwidth: 10 nm) set as in the following table:

Ligand	TMR-Halo	CR1-Halo	CR2-Halo	CR3-Halo	6-SiR-Halo	Fluorescein-Halo
Excitation wavelength [nm]	500	500	580	600	600	450

(due to a non-negligible bathochromic shift of emission of *N*-cyanorhodamine dyes in the presence of CTAB). The data were acquired in triplicates and the results were presented as fluorescence intensity ratios (at the corresponding emission maxima λ_{max}) for SDS (sample with BSA+SDS/sample with BSA only) and CTAB (sample with BSA+CTAB/sample with BSA only).

Cell culture

The human bone osteosarcoma epithelial cell line U-2 OS (ATCC HTB-96) and its genetically engineered variant U-2 OS-Vim-Halo were cultivated in Gibco Dulbecco's Modified Eagle Medium (DMEM) high glucose/GlutaMAX™/pyruvate (31966, ThermoFisher) supplemented with 1% (v/v) penicillin/streptomycin (15140122, Gibco) and 10% (v/v) fetal bovine serum (10500064, ThermoFisher). The genetically engineered human bone osteosarcoma epithelial cell line U-2 OS-CRISPR-NUP96-Halo clone #252 (300448, CLS GmbH) [S9] was cultivated in McCoy's 5a (modified) Medium containing L-glutamine and sodium pyruvate (26600023, Gibco) supplemented with 1% (v/v) penicillin/streptomycin (15140122, Gibco) and 10% (v/v) fetal bovine serum (10500064, ThermoFisher). Cells were grown at 37°C in humidified air with 5% CO_2 and were harvested using TrypLE Express (12604013, Gibco; <20 passages between thawing and experimental use). Cell line authentication and mycoplasma testing was regularly performed. For overexpression of PMLIII, U-2 OS cells were transiently transfected with the N-terminal fusion construct pSNAP-PMLIII (based on a previous plasmid construct [S10]) using the jetPRIME Transfection Reagent (101000027, Polyplus) at 70-90 % confluency according to the manufacturer's protocol. At 24 h post transfection, the cells were subjected to live cell staining.

Holographic time-lapse imaging

Potential cytotoxicity of the dye cores and the HaloTag/SNAP-tag ligands of the *N*-cyanorhodamines, TMR and spectrally similar Janelia Fluor (JF) dyes was assessed by monitoring U-2 OS cell proliferation via holographic time-lapse imaging and by analyzing the frequency of cell division and the cell cycle length. U-2 OS cells were seeded in lumox 96-multiwell plates (94.6120.096, Sarstedt; 100 µL/well, 10^4 cells/mL) 24 h prior to the experiment and kept at 37 °C in humidified air with 5% CO_2. Stock solutions of the the *N*-cyanorhodamine fluorophores and the derived HaloTag/SNAP-tag ligands as well as spectrally similar rhodamine dyes were prepared in DMSO (5 mM). Imaging was performed with a HoloMonitor® M4 cytometer (PHI AB) for a total period of 48 h with 30 min between image captures at 37 °C in humidified air with 5% CO_2. Cell proliferation was followed in absence and in presence of 5 µM dilutions of **CR1-Halo**, **CR1-BG**, **CR2-Halo**, **CR3-Halo**, **5a**, **6a**, *N,N*-dimethylrhodol (**10**), HaloTag TMR Ligand (G8252, Promega), JF_{549} HaloTag Ligand (GA1110, Promega), JF_{585} HaloTag Ligand (CS315105, Promega), TMR and JF_{549} in DMEM. Data analysis comprised cell segmentation, tracking of cells, identification of dividing cells, and cell counting with the AppSuite 3.5 (PHI AB) software.

Cell viability assay

Potential cytotoxicity of the compounds was additionally investigated by evaluating the metabolic activity of U-2 OS cells after treatment. Cells were seeded in lumox 96-multiwell plates (94.6120.096, Sarstedt; 100 µL/well, 5×10^4 cells/mL) and kept at 37 °C in humidified air with 5% CO_2. The next day, the cells were treated with 1/5/10/33/100/333 µM dilutions of **CR1-Halo**, **CR1-BG**, **CR2-Halo**, **CR3-Halo**, **5a**, **6a**, *N,N*-dimethylrhodol (**10**), HaloTag TMR Ligand (G8252, Promega), JF_{549} HaloTag Ligand (GA1110, Promega), JF_{585} HaloTag Ligand (CS315105, Promega), TMR and JF_{549} in DMEM. For the vehicle control, the cells were treated with 0.02/0.10/0.20/0.66/2.00/6.60/20.00% DMSO in DMEM to account for the 5 mM dye stocks in DMSO. After 24 h of incubation, cells were washed three times with DMEM. Next, 20 µL/well of CellTiter-Blue Reagent (G8080, Promega) was added to 100 µL/well of DMEM and the cells were incubated for

4 h at 37 °C in humidified air with 5% CO_2. To stop the formation of resorufin and stabilize the assay, 50 µL of 3% SDS (sodium dodecyl sulfate) was added. Fluorescence intensity was measured at 590 nm with excitation at 560 nm using a CLARIOstar Plus microplate reader (BMG Labtech). To correct for cell culture medium and compound background, the assay was likewise carried out without cells. The corrected fluorescence intensity is directly proportional to the number of viable cells.

Live cell STED and confocal imaging

For live cell STED and confocal imaging, 10^5 cells/well were seeded in 4-well chambered coverslips (Nunc Lab-Tek II chambered coverglasses, 154526, ThermoFisher) and kept for 24 h at 37 °C and 5% CO_2. Stock solutions of the dye cores and the HaloTag/SNAP-tag ligands of the *N*-cyanorhodamines, TMR and spectrally similar JF dyes were prepared in DMSO (5 mM) and further diluted in DMEM to a final working concentration of 1-5 µM. ER-Tracker Blue-White DPX (E12353, Invitrogen), MitoTracker Green FM (M7514, Invitrogen), abberior LIVE 510 tubulin (LV510-0141-50UG, Abberior), abberior LIVE 550 tubulin (LV550-0141-50UG, Abberior), SiR-Hoechst [S11] and GeR-tubulin [S12] were used at final concentrations of 8 µM, 250 nM, 2 µM, 250 nM, 500 nM, 200 nM and 1 µM in DMEM, respectively. SiR-Hoechst was co-incubated with 10 µM verapamil. The cells were stained for 6 h (overnight for off-target affinity experiments) at 37 °C in humidified air with 5% CO_2. Hoechst 33342 (H1399, Invitrogen) was added to the staining solution for the last 10 min of incubation. After washing with DMEM for 1 h, living cells were imaged in Gibco FluoroBrite DMEM (A1896701, ThermoFisher) supplemented with 10% (v/v) fetal bovine serum (10500064, ThermoFisher) and 4 mM Gibco GlutaMAX (35050061, ThermoFisher) on an Abberior expert line microscope (Abberior Instruments) equipped with a 405 nm excitation cw laser, 485 nm, 561 nm and 640 nm excitation pulsed (40 MHz) lasers, a 775 nm pulsed (40 MHz) STED laser, and spectral detection. STED and confocal images were acquired with a 100x/1.4 NA magnification oil immersion lens and with the pinhole set to 100 µm (1 Airy unit). The z-focus drift compensation unit of the microscope minimized axial drift. The imaging parameters for all live cell STED and confocal microscopy data presented in this work are summarized in Table S2. For colocalization analysis between HaloTag TMR Ligand (G8252, Promega) and ER-Tracker Blue-White DPX (E12353, Invitrogen) in the off-target affinity experiments, the Pearson correlation coefficient was calculated with the ImageJ plugin JACoP [S13] and the fluorogram was plotted with the ImageJ plugin Colocalization Finder [S14]. All line profiles on confocal and STED data were drawn on raw data and averaged over 5 pixels. If applicable, confocal line profiles were fitted with a Gaussian function and STED line profiles were fitted with a Lorentzian function. All live cell STED and confocal microscopy data shown are raw data.

Statistical Aspects

Experiments were performed at least in triplicate. Quantitative data are expressed as mean ± standard deviation (SD) and the number of replicates is denoted as *N*. Box plots indicate the interquartile range (box), the outer-most data points falling within 1.5× interquartile range (whiskers), the median (center line) and the mean (circle) of the measurements for replicate experiments. If applicable, means were compared using the Student's *t*-test. P-values < 0.05 were considered statistically significant.

Synthesis and properties of new compounds

Preparation of the starting materials

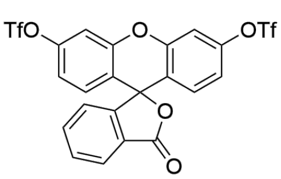

2

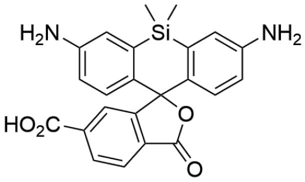

8c *(600SiR)*

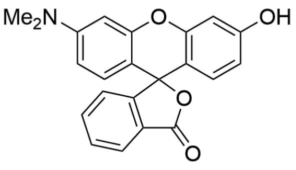

10

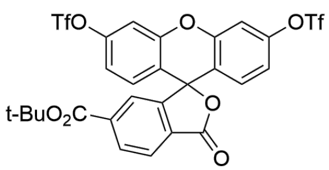

S4

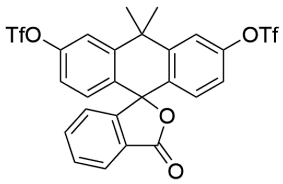

S5

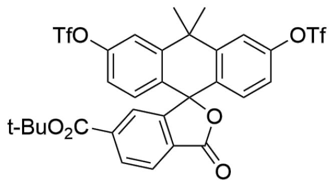

S6

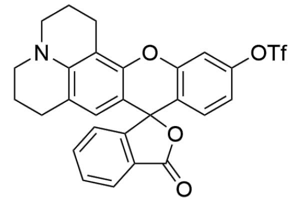

S7

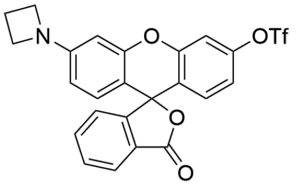

S8

Fluorescein ditriflate (**2**) [S15], *600SiR* dye (**8c**) [S16], *N*,*N*-dimethylrhodol (**10**) [S17], 6-(*tert*-butoxycarbonyl)fluorescein ditriflate (**S4**) [S1], carbofluorescein ditriflate (**S5**) [S18], 6-(*tert*-butoxycarbonyl)carbofluorescein ditriflate (**S6**) [S8], rhodol triflates **S7** [S2] and **S8** [S1] were prepared according to the literature procedures.

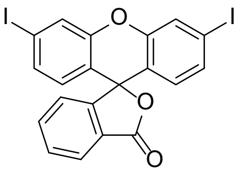

S9

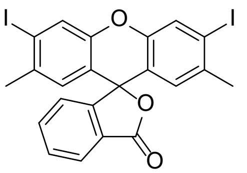

S10

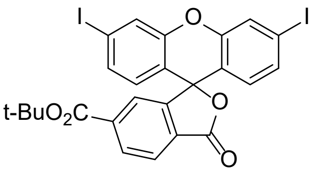

S11

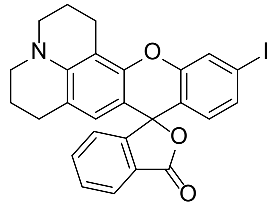

S12

Aryl iodides **S9-S12** were prepared from the corresponding aryl triflates as previously reported in [S19].

***N*,*N*-Dimethylrhodol triflate (1)**

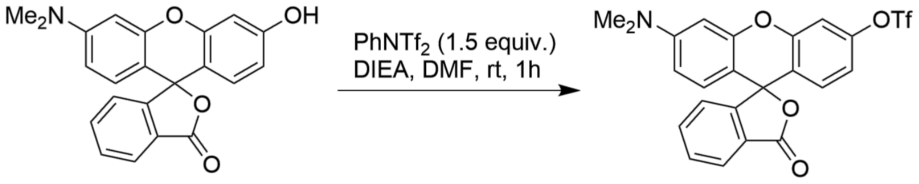

10 **1**

Following the procedure from [S2], rhodol **10** (1.31 g, 3.65 mmol) and *N*-phenyl-bis(trifluoromethanesulfonimide) (1.93 g, 5.41 mmol, 1.5 equiv) were dissolved in DMF (15 mL) under argon atmosphere. DIEA (*N*,*N*-diisopropylethylamine; 2.80 mL, 16.1 mmol, 4.4 equiv) was added, and the reaction mixture stirred at rt for 1 h. The reaction mixture was diluted with water (50 mL), extracted with EtOAc (3x 25 mL), the combined organic layers were washed with water (3x 10 mL) and brine (25 mL) and dried over anhydrous Na_2SO_4. The product was isolated by flash chromatography on silica (80 g Redisep Rf, gradient 0% to 90% EtOAc/hexane) to give **1** as pink powder (1.28 g, 71%) containing 8 mol% hexane.

^{1}H NMR (400 MHz, $CDCl_3$): δ 8.07 – 8.00 (m, 1H), 7.73 – 7.60 (m, 2H), 7.22 (d, *J* = 2.3 Hz, 1H), 7.18 (d, *J* = 7.5 Hz, 1H), 6.92 (dd, *J* = 8.8, 2.4 Hz, 1H), 6.87 (d, *J* = 8.7 Hz, 1H), 6.62 (d, *J* = 8.8 Hz, 1H), 6.52 (d, *J* = 2.5 Hz, 1H), 6.46 (dd, *J* = 8.9, 2.6 Hz, 1H), 3.00 (s, 6H).

^{19}F NMR (376 MHz, $CDCl_3$): δ -72.67.

^{13}C NMR (101 MHz, $CDCl_3$): δ 169.3, 152.8, 152.6, 152.3, 152.2, 150.0, 135.3, 130.2, 130.1, 128.8, 126.9, 125.3, 124.1, 120.3, 118.8 (q, $^1J_{C\text{-}F}$ = 320.9 Hz), 116.4, 110.5, 109.7, 105.7, 98.7, 82.7, 40.4.

HR-MS (ESI) m/z: $[M+H]^+$ 492.0719 (found), 492.0723 (calcd. for $C_{23}H_{16}F_3NO_6S$).

3'-(Dimethylamino)-6'-iodofluoran (3a)

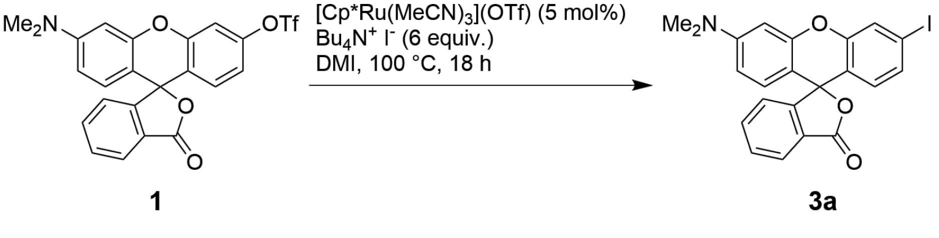

Following the procedure from [S19], a 25 mL oven-dried flask was charged with **1** (194 mg, 0.40 mmol), tetrabutylammonium iodide (882 mg, 2.40 mmol, 6 equiv.), and [Cp*Ru(MeCN)$_3$](OTf) catalyst (10 mg, 0.02 mmol, 5 mol%). The vessel was evacuated and backfilled with argon (3x). Anhydrous 1,3-dimethyl-2-imidazolidinone (3.0 mL) was

then injected, and the resulting mixture was stirred at 100 °C overnight (18 h). The mixture was then poured into water (30 mL) and extracted with hexane/EtOAc (1:4, 3x 25 mL). The organic layers were washed with water/brine (2x 30 mL, 1:1) and brine (30 mL), dried with anhydrous Na_2SO_4, filtered, and evaporated. The product was isolated by flash chromatography on silica (25g Puriflash, gradient 20% to 50% EtOAc/hexane) and freeze-dried from dioxane to give **3a** as pinkish powder (177 mg, 88%) containing 27 mol% dioxane.

^{1}H NMR (400 MHz, $CDCl_3$) δ 8.01 (ddd, *J* = 7.3, 1.4, 0.8 Hz, 1H), 7.70 – 7.56 (m, 3H), 7.31 (dd, *J* = 8.3, 1.7 Hz, 1H), 7.18 – 7.12 (m, 1H), 6.60 (d, *J* = 8.8 Hz, 1H), 6.53 – 6.45 (m, 2H), 6.42 (dd, *J* = 8.9, 2.6 Hz, 1H), 2.99 (s, 6H).
^{13}C NMR (101 MHz, $CDCl_3$) δ 169.6, 153.1, 152.3, 152.1, 152.1, 135.2, 132.5, 129.9, 129.6, 128.8, 126.9, 126.3, 125.2, 124.0, 119.4, 109.3, 105.7, 98.5, 95.2, 83.3, 40.4.
HR-MS (ESI) m/z: $[M+H]^+$ 470.0240 (found), 470.0248 (calcd. for $C_{22}H_{16}INO_3$).

6-Carboxyrhodamine 110 (8a)

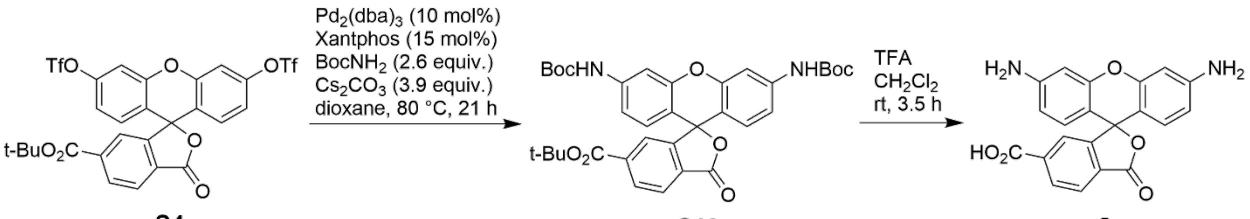

A 25 mL round-bottom flask charged with Cs_2CO_3 (344 mg, 1.06 mmol, 3.9 equiv.) and a stirring bar was dried under vacuum. After cooling down, the flask was backfilled with argon, charged with 6-(*tert*-butoxycarbonyl)fluorescein ditriflate (**S4**) (214 mg, 0.27 mmol), *tert*-butyl carbamate (83 mg, 0.71 mmol, 2.6 equiv.), $Pd_2(dba)_3$ (27.5 mg, 0.03 mmol, 10 mol%) and Xantphos (26 mg, 0.045 mmol, 15 mol%), evacuated and backfilled with argon (3x). After addition of anhydrous dioxane (5.0 mL), the resulting mixture was stirred at 80 °C overnight (21 h). The reaction mixture cooled down, poured into water (20 mL) and extracted with EtOAc (3x 10 mL), the combined organic layers were washed with brine (20 mL) and dried over anhydrous Na_2SO_4. The product was isolated by flash

column chromatography on silica (25 g Puriflash, gradient 0% to 40% EtOAc/hexane) to afford crude Boc-amide **S13** (139 mg, 73% yield), containing 28 mol% $BocNH_2$.

^{1}H NMR (400 MHz, $CDCl_3$): δ 8.22 (dd, *J* = 8.0, 1.3 Hz, 1H), 8.04 (dd, *J* = 8.0, 0.8 Hz, 1H), 7.66 (dd, *J* = 1.4, 0.8 Hz, 1H), 7.47 (d, *J* = 2.2 Hz, 2H), 6.92 (dd, *J* = 8.6, 2.2 Hz, 2H), 6.66 (d, *J* = 8.6 Hz, 2H), 6.60 (s, 2H), 1.54 (s, 9H), 1.52 (s, 18H).

To a solution of crude **S13** (111 mg) in CH_2Cl_2 (5.0 mL), trifluoroacetic acid (1.0 mL) was added. The resulting mixture was stirred at rt for 3.5 h. The mixture was diluted with toluene (5 mL) and evaporated to dryness from toluene (3x) and from CH_2Cl_2 (3x), followed by freeze-drying from dioxane to afford 6-carboxyrhodamine 110 (**8a**) as red powder (TFA salt; 97.5 mg, 71 % yield over 2 steps), containing 46 mol% dioxane.

^{1}H NMR (400 MHz, DMSO-d_6 + 1% TFA-d): δ 8.32 (dd, *J* = 8.2, 0.7 Hz, 1H), 8.28 (dd, *J* = 8.2, 1.6 Hz, 1H), 7.90 (dd, *J* = 1.6, 0.7 Hz, 1H), 7.02 (d, *J* = 9.1 Hz, 2H), 6.83 (dd, *J* = 9.1, 2.1 Hz, 2H), 6.79 (d, *J* = 2.1 Hz, 2H).
^{13}C NMR (101 MHz, DMSO-d_6 + 1% TFA-d): δ 166.0, 165.7, 159.3, 158.4, 157.5, 134.5, 134.2, 133.6, 131.8, 131.4, 130.8, 130.8, 116.9, 113.1, 97.0.
HR-MS (ESI) m/z: $[M+H]^+$ 375.0974 (found), 375.0975 (calcd. for $C_{21}H_{14}N_2O_5$).

6-Carboxycarborhodamine 8b

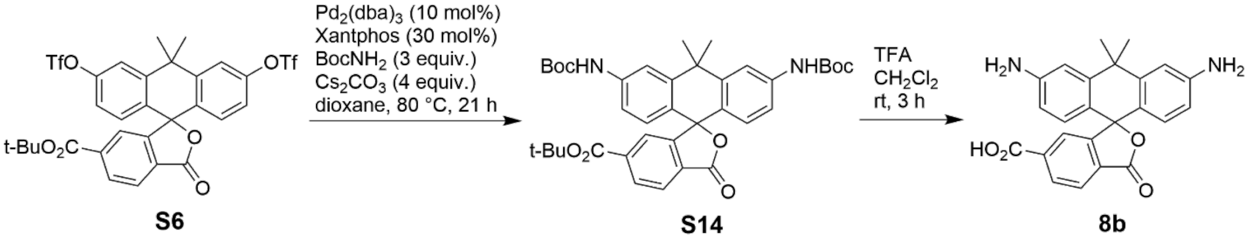

A 25 mL round-bottom flask charged with Cs_2CO_3 (259 mg, 0.78 mmol, 4.0 equiv.) and a stirring bar was dried under vacuum. After cooling down, the flask was backfilled with argon, charged with 6-(*tert*-butoxycarbonyl)carbofluorescein ditriflate (**S6**) (143 mg, 0.20 mmol), *tert*-butyl carbamate (70 mg, 0.60 mmol, 3.0 equiv.), $Pd_2(dba)_3$ (18 mg, 0.02 mmol, 10 mol%) and Xantphos (35 mg, 0.061 mmol, 30 mol%), evacuated and backfilled

with argon (3x). After addition of anhydrous dioxane (2.0 mL), the resulting mixture was stirred at 80 °C overnight (21 h). The reaction mixture was cooled down, poured into sat. aq. NH_4Cl (10 mL), diluted with water (10 mL), and extracted with EtOAc (3x 20 mL). The combined organic layers were washed with brine (30 mL) and dried over anhydrous Na_2SO_4. The product was isolated by flash column chromatography on silica (25 g Puriflash, gradient 0% to 50% EtOAc/hexane), followed by freeze-drying from dioxane to give crude Boc-amide **S14** (127 mg, 86% yield, containing 10 mol% dioxane and 36 mol% $BocNH_2$).

^{1}H NMR (400 MHz, $CDCl_3$): δ 8.16 (dd, *J* = 8.0, 1.3 Hz, 1H), 8.03 (dd, *J* = 8.0, 0.8 Hz, 1H), 7.83 – 7.70 (m, 2H), 7.54 – 7.49 (m, 1H), 7.03 (dd, *J* = 8.6, 2.3 Hz, 2H), 6.67 (d, *J* = 8.6 Hz, 2H), 6.63 (s, 2H), 1.87 (s, 3H), 1.76 (s, 3H), 1.52 (s, 27H).
^{13}C NMR (101 MHz, $CDCl_3$): δ 170.0, 164.2, 155.6, 152.7, 146.3, 139.4, 138.2, 130.5, 129.2, 128.6, 125.3, 125.2, 124.7, 117.6, 116.2, 86.4, 82.6, 81.0, 38.5, 34.8, 33.7, 28.5, 28.1.
HR-MS (ESI) m/z: $[M+H]^+$ 657.3169 (found), 657.3170 (calcd. for $C_{38}H_{44}N_2O_8$).

To a solution of crude **S14** from the previous step (116 mg) in CH_2Cl_2 (2.0 mL), TFA (0.4 mL) was added. The resulting mixture was stirred at rt for 3 h. The mixture was diluted with toluene (3 mL) and evaporated to dryness from toluene (3x) and from CH_2Cl_2 (3x), followed by freeze-drying from dioxane to afford 6-carboxycarborhodamine **8b** as violet powder (TFA salt; 124 mg, 88% yield), which was used without additional purification.

For photophysical characterization, a portion of **8b** (22.3 mg) was further purified by preparative HPLC (Interchim PhC4 250x21.2 mm 5 µm, gradient 10/90 to 50/50 A:B, A = MeCN + 0.1 % formic acid, B = H_2O + 0.1 % formic acid) followed by freeze-drying from dioxane to afford pure **8b** (13.7 mg, containing 64 mol% dioxane) as violet powder.

^{1}H NMR (400 MHz, CD_3OD): δ 8.35 (dd, *J* = 8.2, 0.7 Hz, 1H), 8.32 (dd, *J* = 8.2, 1.5 Hz, 1H), 7.87 (dd, *J* = 1.6, 0.7 Hz, 1H), 7.17 (d, *J* = 2.2 Hz, 2H), 6.92 (d, *J* = 9.0 Hz, 2H), 6.61 (dd, *J* = 9.0, 2.2 Hz, 2H), 1.80 (s, 3H), 1.69 (s, 3H).
^{13}C NMR (101 MHz, CD_3OD): δ 167.9, 167.6, 159.8, 159.0, 139.4, 138.8, 136.0, 135.5, 132.4, 132.1, 131.6, 122.1, 119.0, 116.1, 114.0, 42.4, 35.1, 31.8.

HR-MS (ESI) m/z: $[M+H]^+$ 401.1494 (found), 401.1496 (calcd. for $C_{24}H_{20}N_2O_4$).

Compound S15

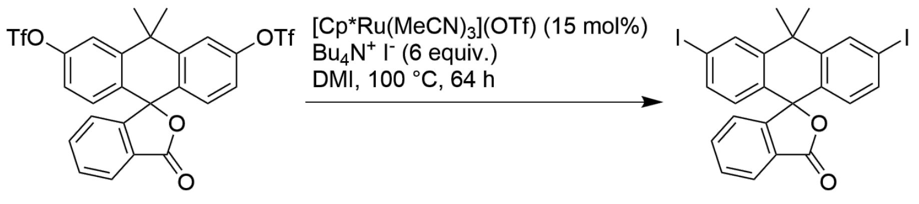

Following the procedure from [S19], a 10 mL oven-dried flask was charged with **S5** (300 mg, 0.48 mmol), tetrabutylammonium iodide (1.07 g, 2.89 mmol, 6 equiv.), and $[Cp^*Ru(MeCN)_3](OTf)$ catalyst (25 mg, 0.048 mmol, 10 mol%), evacuated and backfilled with argon (3x). Anhydrous 1,3-dimethyl-2-imidazolidinone (2.0 mL) was then injected, and the resulting mixture was stirred at 100 °C for 44 h. An LC-MS sample showed mono-iodide still present in the mixture, so an additional portion of $[Cp^*Ru(MeCN)_3](OTf)$ catalyst (12 mg, 0.024 mmol, 5 mol%) was added, the mixture was degassed and stirred at 100 °C for further 20 h. The mixture was then poured into water (200 mL) and extracted with chloroform/hexane (3:1, 3x 40 mL). The combined organic layers were washed with water (2x 200 mL), and brine (100 mL) and dried over anhydrous Na_2SO_4, filtered and evaporated on silica. The product was isolated by flash chromatography on silica (25g Puriflash, gradient 0% to 30% EtOAc/hexane) and freeze-dried from dioxane to give **S15** as white solid (250 mg, 90%) containing 29 mol% dioxane.

^{1}H NMR (400 MHz, $CDCl_3$): δ 8.05 – 8.00 (m, 1H), 7.97 (d, J = 1.7 Hz, 2H), 7.64 – 7.56 (m, 2H), 7.46 (dd, J = 8.4, 1.7 Hz, 2H), 6.99 – 6.94 (m, 1H), 6.50 (d, J = 8.4 Hz, 2H), 1.85 (s, 3H), 1.76 (s, 3H).
^{13}C NMR (101 MHz, $CDCl_3$): δ 170.2, 154.8, 146.6, 136.3, 136.0, 135.3, 130.9, 129.8, 129.6, 125.8, 125.7, 123.5, 96.0, 85.0, 38.0, 34.9, 33.4.
HR-MS (ESI) m/z: $[M+H]^+$ 578.9298 (found), 578.9312 (calcd. for $C_{23}H_{16}I_2O_2$).

Compound S16

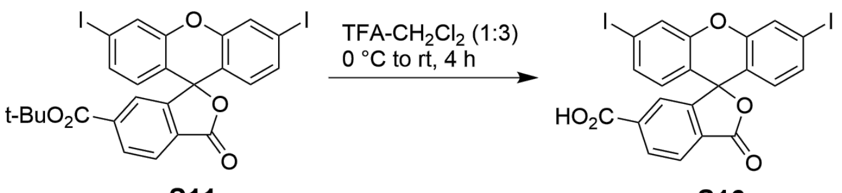

To a solution of ester **S11** (33 mg, 0.051 mmol) in CH_2Cl_2 (0.75 mL) cooled to 0 °C, TFA (0.25 mL) was added slowly. The resulting mixture was stirred at 0 °C for 10 min, allowed to warm up and stirred at rt for 4 h. The mixture was diluted with toluene (3 mL) and evaporated to dryness with toluene (3x) and with CH_2Cl_2 (1x). The residue was freeze-dried from dioxane yield the carboxylic acid **S16** as off-white powder (quant. yield, 32 mg) containing 22 mol% dioxane, which was used without further purification.

^{1}H NMR (400 MHz, DMSO-d$_6$): δ 13.6 (br.s, 1H, CO_2H), 8.25 (dd, *J* = 8.0, 1.4 Hz, 1H), 8.16 (dd, *J* = 7.9, 0.8 Hz, 1H), 7.83 – 7.81 (m, 3H), 7.48 (dd, *J* = 8.3, 1.7 Hz, 2H), 6.67 (d, *J* = 8.3 Hz, 2H).
^{13}C NMR (101 MHz, DMSO-d$_6$): δ 167.6, 165.9, 152.2, 150.4, 137.8, 133.3, 131.4, 129.8, 128.6, 125.7, 125.6, 124.7, 117.8, 97.2, 81.0.
HR-MS (ESI) m/z: $[M+H]^+$ 596.8685 (found), 596.8690 (calcd. for $C_{21}H_{10}I_2O_5$).

Preparation of *N*-cyanorhodamines by Buchwald-Hartwig amination

Compound 5a

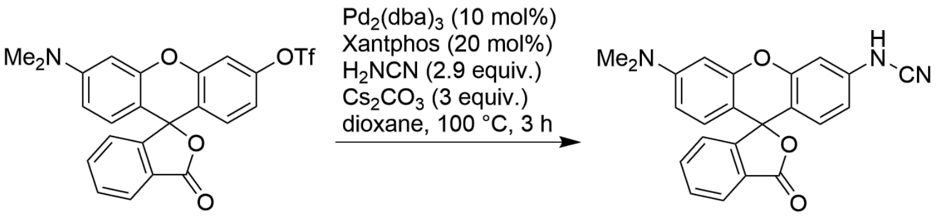

A 10 mL reaction tube (Biotage 2-5 mL microwave vial was used) charged with Cs_2CO_3 (97 mg, 0.30 mmol, 3.0 equiv.) and a stirring bar was dried under vacuum. After cooling

down, the tube was backfilled with argon, charged with cyanamide (12 mg, 0.29 mmol, 2.9 equiv.), $Pd_2(dba)_3$ (9.2 mg, 0.01 mmol, 10 mol%) and Xantphos (20.0 mol%, 0.020 mmol, 11.5 mg) evacuated and backfilled with argon (3x). A solution of **1** (49 mg, 0.1 mmol) in anhydrous, degassed dioxane (1.0 mL) was then injected, and the reaction mixture was stirred at 100 °C for 3 h. The product was isolated by flash column chromatography on silica (12 g Puriflash, gradient 0% to 100% A:B, A = 20% EtOH/CH_2Cl_2 + 0.1% NEt_3, B = CH_2Cl_2 + 0.1% NEt_3) followed by freeze-drying from dioxane to give *N*-cyanorhodamine **5a** as violet powder (22.6 mg, 50% yield), containing 43 mol% NEt_3.

^{1}H NMR (400 MHz, pyridine-d$_5$): δ 8.31 (d, *J* = 7.6 Hz, 1H), 7.74 (td, *J* = 7.5, 1.3 Hz, 1H), 7.67 (td, *J* = 7.5, 1.1 Hz, 1H), 7.39 (dt, *J* = 7.6, 1.0 Hz, 1H), 6.98 – 6.84 (m, 3H), 6.63 (d, *J* = 2.6 Hz, 1H), 6.50 (dd, *J* = 9.0, 2.6 Hz, 1H), 2.86 (s, 6H).
^{13}C NMR (101 MHz, pyridine-d$_5$) δ 169.9, 135.2, 130.8, 130.6, 129.8, 115.3, 110.8, 103.8, 99.0, 40.4 (some quarternary C's were not detected).
HR-MS (ESI) m/z: $[M+H]^+$ 384.1341 (found), 384.1343 (calcd. for $C_{23}H_{17}N_3O_3$).

Compound 5b

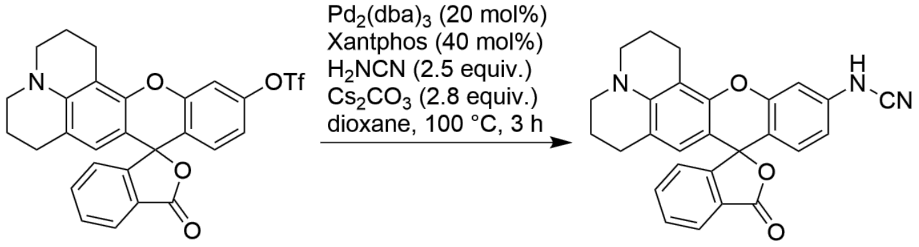

S7 5b

A 10 mL reaction tube was charged with Cs_2CO_3 (47 mg, 0.15 mmol, 2.8 equiv.) and a stirring bar was dried under vacuum. After cooling down, the tube was backfilled with argon, charged with cyanamide (5.4 mg, 0.13 mmol, 2.5 equiv.), $Pd_2(dba)_3$ (9.2 mg, 0.01 mmol, 20 mol%), Xantphos (11.2 mg, 0.02 mmol, 40 mol%) and **S7** (28 mg, 0.05 mmol), evacuated and backfilled with argon (3x). Anhydrous, degassed dioxane (0.5 mL) was then injected, and the reaction mixture was stirred at 100 °C for 3 h. The product was

isolated by flash column chromatography on silica (12 g Puriflash, gradient 0% to 100% A:B, A = 20% EtOH/CH_2Cl_2 + 0.5% acetic acid, B = CH_2Cl_2 + 0.5% acetic acid) and re-purified by preparative HPLC (Interchim PhC4 250x21.2 mm 5 µm, gradient 35/65 to 65/35 A:B, A = MeCN + 0.1% formic acid, B = H_2O + 0.1% formic acid) followed by freeze-drying from dioxane to afford cyanamide **5b** as violet powder (10.2 mg, 45% yield).

^{1}H NMR (400 MHz, pyridine-d_5): δ 8.53 (d, *J* = 8.0 Hz, 1H), 7.80 – 7.65 (m, 2H), 7.50 (dd, *J* = 7.3, 1.4 Hz, 1H), 7.39 (s, 1H), 7.11 – 7.01 (m, 2H), 6.77 (s, 1H), 3.19 – 2.92 (m, 4H), 2.81 – 2.56 (m, 2H), 2.41 – 2.25 (m, 2H), 1.85 – 1.69 (m, 2H), 1.67 – 1.43 (m, 2H).
^{13}C NMR (101 MHz, pyridine-d_5): δ 169.1, 133.5, 130.8, 130.3, 126.4, 115.4, 106.2, 103.5, 50.7, 50.24, 27.9, 21.5, 20.7, 20.6 (some quarternary C's were not detected).
HR-MS (ESI) m/z: $[M+H]^+$ 436.1656 (found), 436.1656 (calcd. for $C_{27}H_{21}N_3O_3$).

Compound 5c

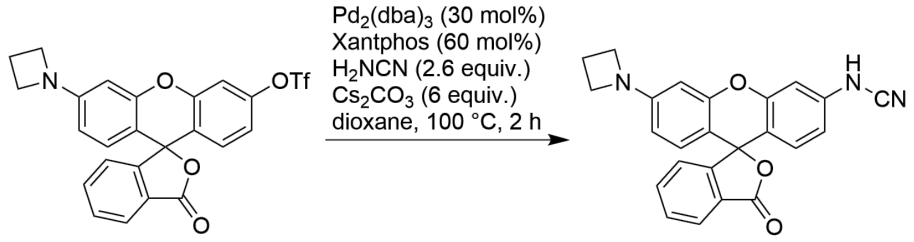

S8 **5c**

A 10 mL reaction tube was charged with Cs_2CO_3 (47 mg, 0.14 mmol, 6 equiv.) and a stirring bar was dried under vacuum. After cooling down, the tube was backfilled with argon, charged with cyanamide (2.5 mg, 0.058 mmol, 2.6 equiv.), $Pd_2(dba)_3$ (6.4 mg, 0.007 mmol, 30 mol%), Xantphos (8.2 mg, 0.014 mmol, 60 mol%) and **S8** (11 mg, 0.023 mmol), evacuated and backfilled with argon (3x). Anhydrous, degassed dioxane (0.5 mL) was then injected, and the reaction mixture was stirred at 100 °C for 2 h. The product was isolated by flash column chromatography on silica (12 g Puriflash, gradient 0% to 100% A:B, A = 20% EtOH/CH_2Cl_2 + 0.5% acetic acid, B = CH_2Cl_2 + 0.5% acetic acid) and re-purified by preparative HPLC (Interchim PhC4 250x21.2 mm 5 µm, gradient 35/65 to

65/35 A:B, A = MeCN + 0.1% formic acid, B = H_2O + 0.1% formic acid) followed by freeze-drying from dioxane to afford cyanamide **5c** as violet powder (3.2 mg, 35% yield).

^{1}H NMR (400 MHz, pyridine-d$_5$): δ 8.32 (d, *J* = 7.6 Hz, 1H), 7.71 (td, *J* = 7.5, 1.3 Hz, 1H), 7.65 (td, *J* = 7.5, 1.2 Hz, 1H), 7.36 (d, *J* = 7.7 Hz, 1H), 6.95 – 6.86 (m, 3H), 6.31 (d, *J* = 2.3 Hz, 1H), 6.18 (dd, *J* = 8.7, 2.3 Hz, 1H), 3.77 (t, *J* = 7.3 Hz, 4H), 2.10 (p, *J* = 7.3 Hz, 2H).
^{13}C NMR (101 MHz, pyridine-d$_5$): δ 135.8, 134.4, 130.0, 129.9, 129.5, 126.6, 123.8, 109.0, 103.3, 97.1, 51.8, 16.4 (indirect detection from a gHSQC experiment, only H-coupled carbons are resolved).
HR-MS (ESI) m/z: $[M+H]^+$ 396.1340 (found), 396.1343 (calc. for $C_{24}H_{17}N_3O_3$).

Preparation of *N*-cyanorhodamines by Ullmann coupling

Compound 5a

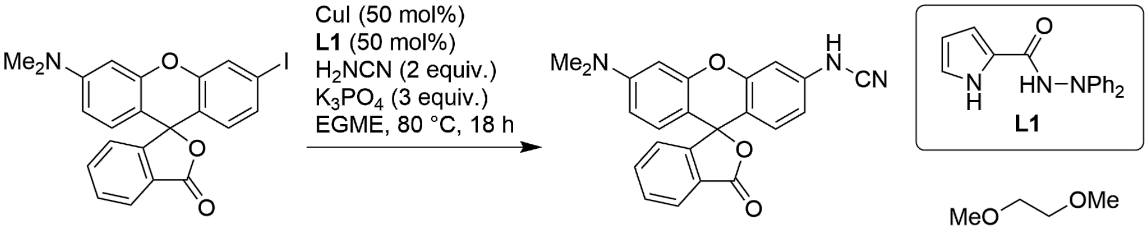

3a **5a**

A 10 mL reaction tube was charged with K_3PO_4 (63 mg, 0.30 mmol, 3 equiv.) and a stirring bar was dried under vacuum. After cooling down, the tube was backfilled with argon, charged with iodide **3a** (48 mg, 0.10 mmol), cyanamide (9 mg, 0.21 mmol, 2 equiv.), copper(I) iodide (9.4 mg, 0.05 mmol, 50 mol%) and ligand **L1** (13 mg, 0.05 mmol, 50 mol%; prepared according to the literature procedure [S19]), and evacuated and backfilled with argon (3x). Ethylene glycol monomethyl ether (EGME; 1.0 mL) was added, and the reaction mixture was stirred at 80 °C overnight (18 h). The mixture was poured into water (10 mL), acidified with acetic acid to pH 4, and extracted with EtOAc (2x 20 mL). The combined organic layers were washed with brine (20 mL), dried over anhydrous Na_2SO_4, and the product was isolated by flash column chromatography on silica (12 g Puriflash, gradient 20% to 80% A:B, A = EtOAc + 0.5% acetic acid, B = hexane + 0.5% acetic acid).

Further purification by preparative HPLC (Interchim PhC4 250x21.2 mm 5 µm, gradient 30/70 to 60/40 A:B, A = MeCN + 0.1 % formic acid, B = H_2O + 0.1 % formic acid), followed by freeze-drying from dioxane, afforded cyanamide **5a** as dark-red powder (22.3 mg, 57% yield).

The analytical data of a sample matches the compound **5a** prepared by Buchwald-Hartwig amination.

Compound 5b

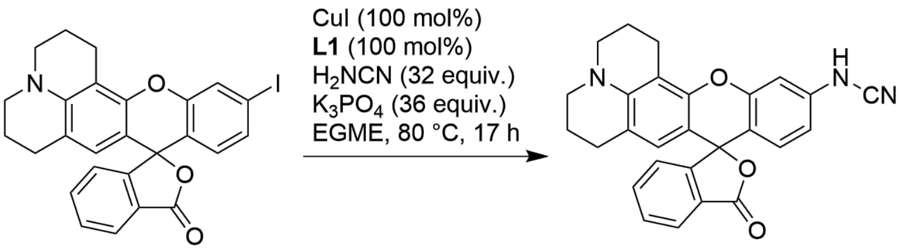

A 10 mL reaction tube was charged with K_3PO_4 (137 mg, 0.64 mmol, 36 equiv.) and a stirring bar was dried under vacuum. After cooling down, the tube was backfilled with argon, charged with iodide **S12** (9.3 mg, 0.018 mmol), cyanamide (24 mg, 0.57 mmol, 32 equiv.), copper(I) iodide (3.2 mg, 0.017 mmol, 100 mol%) and ligand **L1** (3.8 mg, 0.017 mmol, 100 mol%), and evacuated and backfilled with argon (3x). Ethylene glycol monomethyl ether (EGME; 1.0 mL) was added, and the reaction mixture was stirred at 80 °C overnight (17 h). The mixture was diluted with CH_2Cl_2, acetic acid was added to adjust pH to 4, evaporated on Celite, and the product was isolated by flash column chromatography on silica (12 g Puriflash, gradient 0% to 100% A:B, A = 20% EtOH/CH_2Cl_2 + 0.5% acetic acid, B = CH_2Cl_2 + 0.5% acetic acid). Further purification by preparative HPLC (Interchim PhC4 250x21.2 mm 5 µm, gradient 35/65 to 65/35 A:B, A = MeCN + 0.1% formic acid, B = H_2O + 0.1% formic acid), followed by freeze-drying from dioxane, afforded cyanamide **5b** as violet powder (4.0 mg, 51% yield), containing 11 mol% dioxane.

The analytical data of a sample matches the compound **5b** prepared by Buchwald-Hartwig amination.

Compound 6a

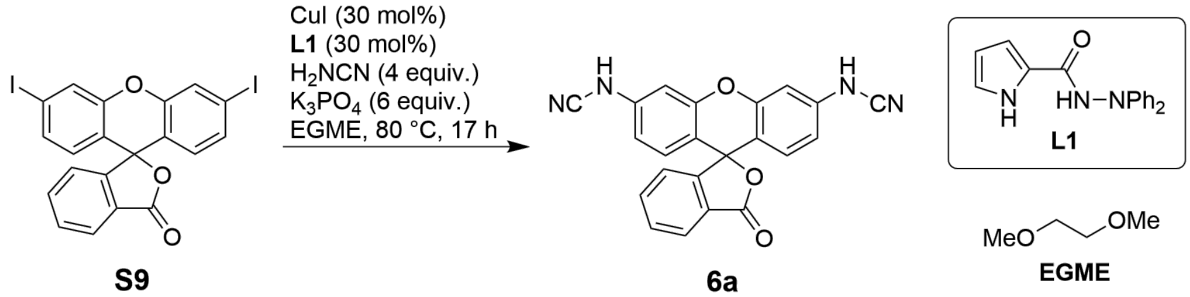

A 10 mL reaction tube was charged with K_3PO_4 (131 mg, 0.62 mmol, 6 equiv.) and a stirring bar was dried under vacuum. After cooling down, the tube was backfilled with argon, charged with iodide **S9** (55 mg, 0.10 mmol), cyanamide (17 mg, 0.40 mmol, 4 equiv.), copper(I) iodide (5.2 mg, 0.03 mmol, 30 mol%) and ligand **L1** (7.2 mg, 0.03 mmol, 30 mol%), and evacuated and backfilled with argon (3x). Ethylene glycol monomethyl ether (EGME; 1.0 mL) was added, and the reaction mixture was stirred at 80 °C overnight (17 h). The mixture was poured into water (20 mL), acidified with acetic acid to pH 4, and extracted with EtOAc (5x 10 mL). The combined organic layers were washed with brine (20 mL), dried over anhydrous Na_2SO_4, and the product was isolated by flash column chromatography on silica (12 g Puriflash, gradient 20% to 80% A:B, A = EtOAc + 0.5% acetic acid, B = hexane + 0.5% acetic acid). Further purification by preparative HPLC (Interchim PhC4 250x21.2 mm 5 µm, gradient 30/70 to 60/40 A:B, A = MeCN + 0.1 % formic acid, B = H_2O + 0.1 % formic acid), followed by freeze-drying from dioxane, afforded cyanamide **6a** as pink powder (19.0 mg, 49% yield).

^{1}H NMR (400 MHz, DMSO-d$_6$): δ 10.66 (br s, 2H), 8.03 (dt, *J* = 7.5, 1.0 Hz, 1H), 7.81 (td, *J* = 7.5, 1.2 Hz, 1H), 7.74 (td, *J* = 7.5, 1.1 Hz, 1H), 7.30 (dt, *J* = 7.7, 1.0 Hz, 1H), 6.89 (d, *J* = 2.2 Hz, 2H), 6.80 (d, *J* = 8.6 Hz, 2H), 6.76 (dd, *J* = 8.6, 2.2 Hz, 2H).
^{13}C NMR (101 MHz, DMSO-d$_6$): δ 168.6, 152.3, 151.3, 141.4, 135.9, 130.4, 130.0, 125.6, 124.9, 124.0, 112.9, 111.9, 111.2, 102.4, 81.5.
HR-MS (ESI) m/z: $[M+H]^+$ 381.0980 (found), 381.0982 (calcd. for $C_{22}H_{12}N_4O_3$).

Compound 6b

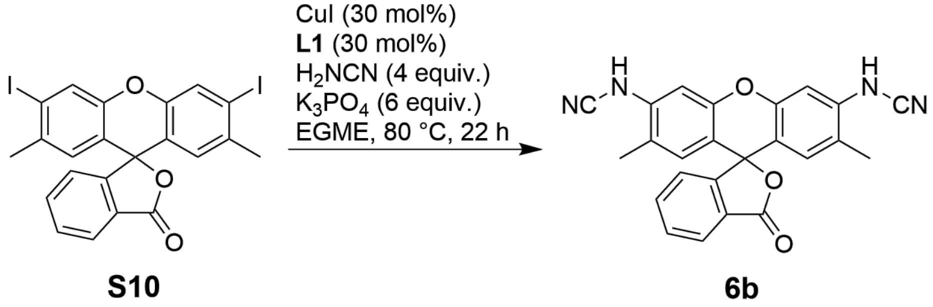

A 10 mL reaction tube was charged with K_3PO_4 (132 mg, 0.62 mmol, 6 equiv.) and a stirring bar was dried under vacuum. After cooling down, the tube was backfilled with argon, charged with iodide **S10** (59 mg, 0.10 mmol), cyanamide (18 mg, 0.40 mmol, 4 equiv.), copper(I) iodide (5 mg, 0.03 mmol, 30 mol%) and ligand **L1** (7 mg, 0.03 mmol, 30 mol%), and evacuated and backfilled with argon (3x). Ethylene glycol monomethyl ether (EGME; 1.0 mL) was added, and the reaction mixture was stirred at 80 °C overnight (22 h). The mixture was poured into water (20 mL), acidified with acetic acid to pH 3, and extracted with EtOAc (3x 10 mL). The combined organic layers were washed with brine (10 mL), dried over anhydrous Na_2SO_4, and the product was isolated by flash column chromatography on silica (12 g Puriflash, gradient 20% to 100% A:B, A = EtOAc + 0.5% acetic acid, B = hexane + 0.5% acetic acid). Further purification by preparative HPLC (Interchim PhC4 250x21.2 mm 5 µm, gradient 40/60 to 80/20 A:B, A = MeCN + 0.1 % formic acid, B = H_2O + 0.1 % formic acid), followed by freeze-drying from dioxane, afforded cyanamide **6b** as pink powder (21.9 mg, 49% yield).

^{1}H NMR (400 MHz, DMSO-d_6): δ 9.75 (s, 2H), 8.02 (dt, *J* = 7.4, 1.1 Hz, 1H), 7.82 – 7.67 (m, 2H), 7.29 – 7.20 (m, 1H), 6.94 (s, 2H), 6.60 (s, 2H), 2.03 (s, 6H).
^{13}C NMR (101 MHz, DMSO-d_6): δ 168.7, 152.6, 149.5, 139.5, 135.8, 130.3, 129.9, 125.5, 125.0, 123.9, 121.5, 112.7, 111.8, 102.4, 81.4, 16.5.
HR-MS (ESI) m/z: $[M+H]^+$ 409.1293 (found), 409.1295 (calcd. for $C_{24}H_{16}N_4O_3$).

Compound 6c

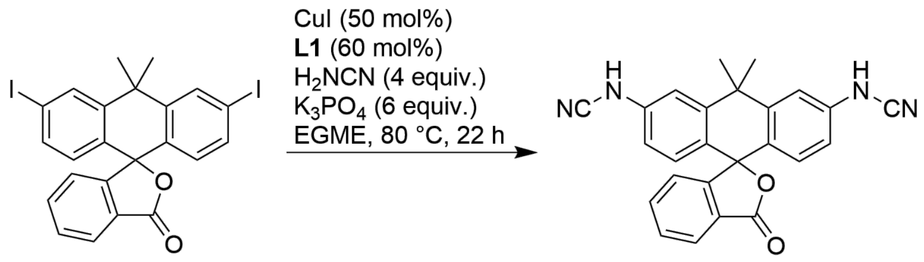

S15 **6c**

A 10 mL reaction tube was charged with K_3PO_4 (65 mg, 0.30 mmol, 6 equiv.) and a stirring bar was dried under vacuum. After cooling down, the tube was backfilled with argon, charged with iodide **S15** (31 mg, 0.054 mmol), cyanamide (10 mg, 0.24 mmol, 4.4 equiv.), copper(I) iodide (5.4 mg, 0.028 mmol, 50 mol%) and ligand **L1** (7.6 mg, 0.034 mmol, 60 mol%), and evacuated and backfilled with argon (3x). Ethylene glycol monomethyl ether (EGME; 1.0 mL) was added, and the reaction mixture was stirred at 80 °C overnight (22 h). The mixture was diluted with EtOAc, acetic acid was added to adjust pH to 4, the mixture was evaporated on Celite and the product was isolated by flash column chromatography on silica (12 g Puriflash, gradient 20% to 80% A:B, A = EtOAc + 0.5% acetic acid, B = hexane + 0.5% acetic acid). Further purification by preparative HPLC (Interchim PhC4 250x21.2 mm 5 µm, gradient 35/65 to 65/35 A:B, A = MeCN + 0.1 % formic acid, B = H_2O + 0.1 % formic acid), followed by freeze-drying from dioxane, afforded cyanamide **6c** as light-pink powder (18.9 mg, 81% yield), containing 25 mol% dioxane.

^{1}H NMR (400 MHz, DMSO-d_6): δ 10.38 (br s, 2H), 8.06 – 7.99 (m, 1H), 7.71 (dtd, *J* = 20.6, 7.3, 1.2 Hz, 2H), 7.24 (d, *J* = 2.4 Hz, 2H), 7.11 – 7.06 (m, 1H), 6.85 (dd, *J* = 8.6, 2.4 Hz, 2H), 6.71 (d, *J* = 8.6 Hz, 2H), 1.79 (s, 3H), 1.68 (s, 3H).
^{13}C NMR (101 MHz, DMSO-d_6): δ 169.6, 154.6, 146.0, 139.7, 135.7, 129.9, 129.4, 125.2, 125.1, 125.0, 123.6, 114.6, 112.3, 111.7, 84.9, 37.8, 34.3, 33.0.
HR-MS (ESI) m/z: $[M+H]^+$ 407.1503 (found), 407.1503 (calcd. for $C_{25}H_{18}N_4O_2$).

Compound 7

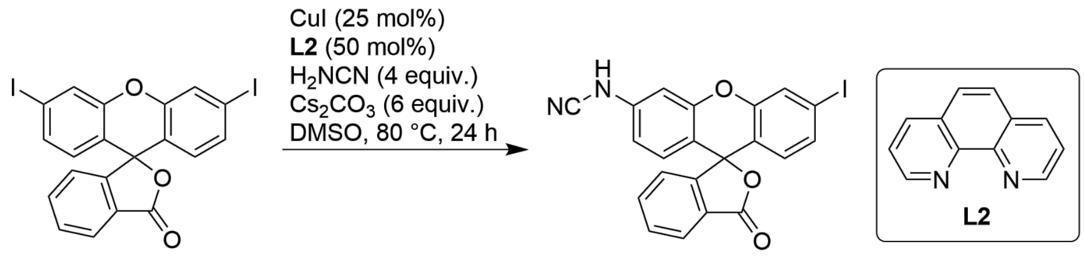

A 10 mL reaction tube was charged with Cs_2CO_3 (196 mg, 0.60 mmol, 6 equiv.) and a stirring bar was dried under vacuum. After cooling down, the tube was backfilled with argon, charged with iodide **S9** (55 mg, 0.10 mmol), cyanamide (18 mg, 0.42 mmol, 4 equiv.), copper(I) iodide (4.6 mg, 0.025 mmol, 25 mol%) and ligand **L2** (1,10-phenanthroline; 9.8 mg, 0.055 mmol, 50 mol%), and evacuated and backfilled with argon (3x). DMSO (1.0 mL) was added, and the reaction mixture was stirred at 80 °C for 24 h. The reaction mixture was diluted with ethanol, acetic acid was added to adjust pH to 4, and the mixture was evaporated to dryness. The product was isolated by flash column chromatography on silica (12 g Puriflash, gradient 0% to 100% A:B, A = EtOAc + 0.5% acetic acid, B = hexane + 0.5% acetic acid) to give cyanamide **7** as pink powder (16.3 mg, 34% yield), containing 18 mol% dioxane.

^{1}H NMR (400 MHz, DMSO-d_6): δ 8.04 (dt, *J* = 7.5, 1.1 Hz, 1H), 7.84 (d, *J* = 1.7 Hz, 1H), 7.81 (td, *J* = 7.5, 1.3 Hz, 1H), 7.74 (td, *J* = 7.4, 1.1 Hz, 1H), 7.48 (dd, *J* = 8.3, 1.7 Hz, 1H), 7.33 (dt, *J* = 7.7, 1.0 Hz, 1H), 6.86 (d, *J* = 2.3 Hz, 1H), 6.82 (d, *J* = 8.6 Hz, 1H), 6.77 (dd, *J* = 8.6, 2.2 Hz, 1H), 6.59 (d, *J* = 8.3 Hz, 1H).
^{13}C NMR (101 MHz, DMSO): δ 168.5, 152.2, 151.0, 150.7, 141.7, 136.0, 133.2, 130.5, 130.0, 129.6, 125.6, 125.4, 125.0, 124.0, 118.6, 112.4, 112.1, 111.3, 102.4, 96.8, 81.1.
HR-MS (ESI) m/z: $[M+H]^+$ 466.9883 (found), 466.9887 (calcd. for $C_{21}H_{11}IN_2O_3$).

Compound CR1

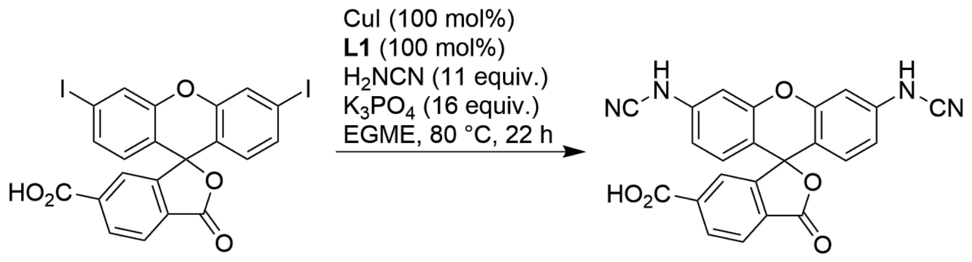

S16 **CR1**

A 10 mL reaction tube was charged with K_3PO_4 (67 mg, 0.32 mmol, 16 equiv.) and a stirring bar was dried under vacuum. After cooling down, the tube was backfilled with argon, charged with iodide **S16** (10.3 mg, 0.02 mmol), cyanamide (9.1 mg, 0.22 mmol, 11 equiv.), copper(I) iodide (3.6 mg, 0.02 mmol, 100 mol%) and ligand **L1** (4.5 mg, 0.02 mmol, 100 mol%), and evacuated and backfilled with argon (3x). Ethylene glycol monomethyl ether (EGME; 1.0 mL) was added, and the reaction mixture was stirred at 80 °C overnight (22 h). The mixture was diluted with EtOAc, acetic acid was added to adjust pH to 4, the mixture was evaporated on Celite and the product was isolated by flash column chromatography on silica (12 g Puriflash, gradient 0% to 100% A:B, A = 20 % EtOH/CH_2Cl_2 + 0.5 % formic acid, B = CH_2Cl_2+ 0.5 % formic acid). Further purification by preparative HPLC (Interchim PhC4 250x21.2 mm 5 µm, gradient 30/70 to 60/40 A:B, A = MeCN + 0.1 % formic acid, B = H_2O + 0.1 % formic acid; loaded in DMSO solution), followed by freeze-drying from dioxane, afforded cyanamide **CR1** as light-pink powder (5.5 mg, 72% yield), containing 22 mol% DMSO.

^{1}H NMR (400 MHz, DMSO-d$_6$): δ 8.24 (dd, *J* = 8.0, 1.4 Hz, 1H), 8.15 (d, *J* = 8.0 Hz, 1H), 7.73 (s, 1H), 6.89 (d, *J* = 2.2 Hz, 2H), 6.85 (d, *J* = 8.6 Hz, 2H), 6.75 (dd, *J* = 8.6, 2.4 Hz, 2H).
^{13}C NMR (101 MHz, DMSO-d$_6$): δ 167.3, 166.0, 153.1, 151.4, 141.5, 137.3, 131.2, 130.1, 128.8, 125.5, 124.6, 112.3, 111.9, 111.2, 102.5, 81.9.
HR-MS (ESI) m/z: $[M+H]^+$ 425.0875 (found), 425.0880 (calcd. for $C_{23}H_{12}N_4O_5$).

Preparation of *N*-cyanorhodamines via base-induced degradation of 1-aryltetrazoles

Compound 9a

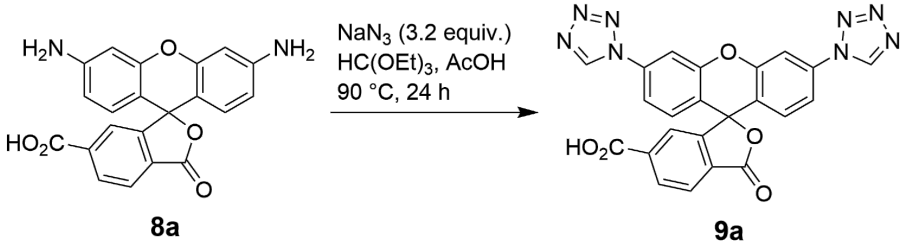

A mixture of **8a** (TFA salt; 40 mg, 0.082 mmol) and sodium azide (17 mg, 0.26 mmol, 3.2 equiv.) in acetic acid (0.8 mL) and triethyl orthoformate (0.20 mL) was stirred at 90 °C for 24 h. The product was isolated by preparative HPLC (Interchim PhC4 250x21.2 mm 5 µm, gradient 35/65 to 65/35 A:B, A = MeCN + 0.1 % formic acid, B = H_2O + 0.1 % formic acid) followed by freeze-drying from dioxane, giving bis-tetrazole **9a** as pink powder (15.6 mg, 45% yield).

^{1}H NMR (400 MHz, DMSO-d_6 + 1% TFA-d): δ 10.2 (s, 2H), 8.30 (dd, *J* = 8.0, 1.3 Hz, 1H), 8.23 (dd, *J* = 8.0, 0.8 Hz, 1H), 8.13 (d, *J* = 2.2 Hz, 2H), 7.93 (dd, *J* = 1.3, 0.8 Hz, 1H), 7.75 (dd, *J* = 8.6, 2.2 Hz, 2H), 7.30 (d, *J* = 8.6 Hz, 2H).
^{13}C NMR (101 MHz, DMSO-d_6 + 1% TFA-d): δ 167.6, 165.9, 152.4, 150.8, 142.7, 137.8, 135.6, 131.6, 130.5, 128.6, 126.0, 124.8, 119.0, 117.2, 109.7, 80.4.
HR-MS (ESI) m/z: $[M+H]^+$ 481.1003 (found), 481.1003 (calcd. for $C_{23}H_{12}N_8O_5$).

Compound 9b

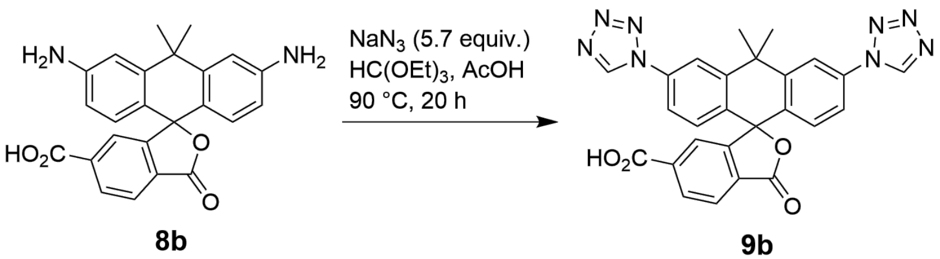

A mixture of **8b** (26 mg, 0.066 mmol) and sodium azide (24 mg, 0.37 mmol, 5.7 equiv.) in acetic acid (1 mL) and triethyl orthoformate (0.15 mL) was stirred at 90 °C for 20 h. The product was isolated by preparative HPLC (Interchim PhC4 250x21.2 mm 5 µm, gradient 35/65 to 65/35 A:B, A = MeCN + 0.1 % formic acid, B = H_2O + 0.1 % formic acid) followed by freeze-drying from dioxane, giving bis-tetrazole **9b** as off-white powder (19.9 mg, 59% yield).

^{1}H NMR (400 MHz, DMSO-d$_6$): δ 13.7 (br.s, 1H, CO_2H), 10.22 (s, 2H), 8.44 (d, *J* = 2.3 Hz, 2H), 8.24 (s, 1H), 7.80 (dd, *J* = 8.7, 2.2 Hz, 2H), 7.54 – 7.52 (m, 1H), 7.16 (d, *J* = 8.7 Hz, 2H), 2.04 (s, 3H), 1.93 (s, 3H).
^{13}C NMR (101 MHz, DMSO-d$_6$): δ 168.6, 165.8, 154.5, 146.1, 142.6, 137.8, 134.8, 131.1, 131.0, 129.6, 127.8, 126.3, 123.6, 120.4, 120.0, 83.8, 38.6, 33.7.
HR-MS (ESI) m/z: $[M+H]^+$ 507.1522 (found), 507.1524 (calcd. for $C_{26}H_{18}N_8O_4$).

Compound 9c

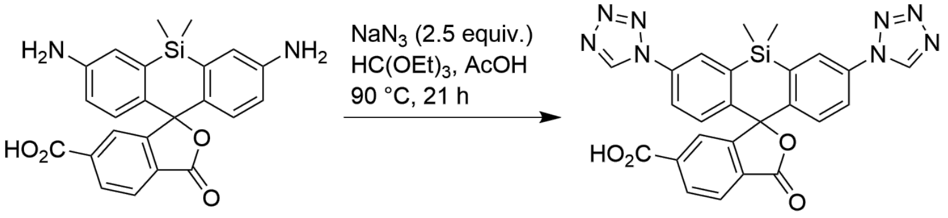

8c 9c

A mixture of **8c** (34 mg, 0.066 mmol) and sodium azide (13 mg, 0.20 mmol, 2.5 equiv.) in acetic acid (0.8 mL) and triethyl orthoformate (0.2 mL) was stirred at 90 °C for 21 h. The product was isolated by preparative HPLC (Interchim PhC4 250x21.2 mm 5 µm, gradient 40/60 to 70/30 A:B, A = MeCN + 0.1 % formic acid, B = H_2O + 0.1 % formic acid) followed by freeze-drying from dioxane, giving bis-tetrazole **9c** as light-pink powder (10.6 mg, 25% yield).

^{1}H NMR (400 MHz, DMSO-d$_6$): δ 13.7 (br.s, 1H, CO_2H), 10.16 (s, 2H), 8.46 (d, *J* = 2.5 Hz, 2H), 8.18 (s, 1H), 8.17 (s, 1H), 7.96 (dd, *J* = 8.7, 2.5 Hz, 2H), 7.82 (t, *J* = 1.0 Hz, 1H), 7.46 (d, *J* = 8.8 Hz, 2H), 0.88 (s, 3H), 0.75 (s, 3H).

^{13}C NMR (101 MHz, DMSO-d_6): δ 168.8, 165.7, 154.0, 144.2, 142.5, 137.6, 136.2, 133.6, 130.9, 127.9, 127.0, 126.8, 126.3, 123.7, 123.5, 88.0, -0.6, -0.7.
HR-MS (ESI) m/z: $[M+H]^+$ 523.1292 (found), 523.1293 (calcd. for $C_{25}H_{18}N_8O_4Si$).

Compound CR1

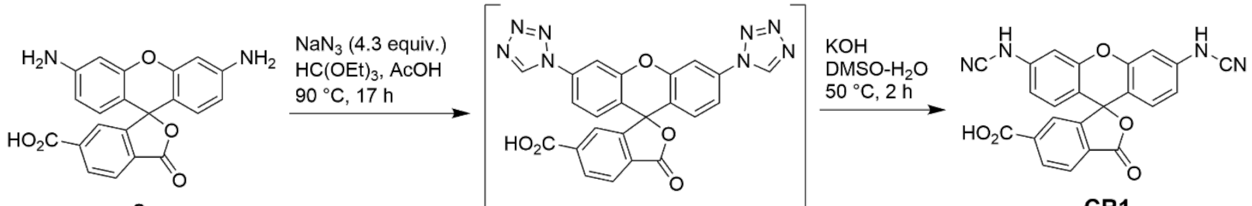

Compound CR1 was prepared from **8a** in the following one-pot procedure: a mixture of **8a** (TFA salt; 29 mg, 0.051 mmol) and sodium azide (14 mg, 0.22 mmol, 4.3 equiv.) in acetic acid (0.8 mL) and triethyl orthoformate (0.20 mL) was stirred at 90 °C overnight (17 h). The solvents were removed by evaporation and crude residue was resuspended in DMSO (0.8 mL). KOH (1.6 mL of 1M solution in water) was gradually added, and the resulting mixture was stirred at rt for 3h and at 50 °C for 2 h, until HPLC control showed complete conversion of bis-tetrazole (**9a**). The reaction mixture was diluted with water (10 mL), acidified with acetic acid to pH 3 and extracted with EtOAc (3x 10 mL). The combined organic layers were evaporated to dryness, and the product was isolated by preparative HPLC (Interchim PhC4 250x21.2 mm 5 µm, gradient 30/70 to 60/40 A:B, A = MeCN + 0.1 % formic acid, B = H_2O + 0.1 % formic acid) followed by freeze-drying from dioxane, giving **CR1** dye as pink powder (14.9 mg, 67% yield) containing 12 mol% dioxane.

The analytical data of a sample matches the compound **CR1** prepared by Ullmann coupling.

Compound CR2

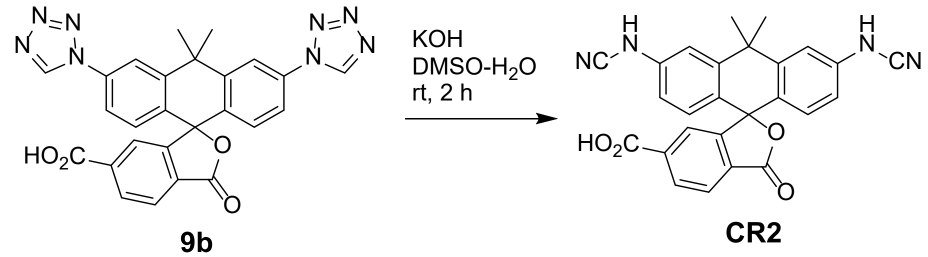

9b **CR2**

KOH (0.1 mL of 1M solution in water) was added to the suspension of **9b** (15 mg, 0.03 mmol) in DMSO (0.4 mL), and the reaction mixture was stirred at rt for 2 h. The reaction mixture was diluted with water (10 mL), acidified with 1M HCl to pH 1 and extracted with CH_2Cl_2 (4x 10 mL). The combined organic layers were evaporated, and the product was isolated by preparative HPLC (Interchim PhC4 250x21.2 mm 5 µm, gradient 30/70 to 60/40 A:B, A = MeCN + 0.1 % formic acid, B = H_2O + 0.1 % formic acid) followed by freeze-drying from dioxane, giving the dye **CR2** as light-pink powder (12.3 mg, 84% yield) containing 32 mol% dioxane.

^{1}H NMR (400 MHz, DMSO-d$_6$): δ 10.49 (br s, 2H), 8.20 (d, *J* = 8.0 Hz, 1H), 8.14 (d, *J* = 7.9 Hz, 1H), 7.46 (s, 1H), 7.25 (d, *J* = 2.4 Hz, 2H), 6.85 (dd, *J* = 8.5, 2.3 Hz, 2H), 6.75 (d, *J* = 8.6 Hz, 2H), 1.79 (s, 3H), 1.68 (s, 3H).
^{13}C NMR (101 MHz, DMSO-d$_6$): δ 168.8, 166.0, 154.7, 146.1, 134.0, 137.7, 130.8, 129.6, 128.5, 125.8, 124.6, 123.8, 114.8, 112.5, 111.7, 85.2, 37.9, 34.4, 33.2.
HR-MS (ESI) m/z: $[M+H]^+$ 451.1401 (found), 451.1401 (calcd. for $C_{26}H_{18}N_4O_4$).

Compound CR3

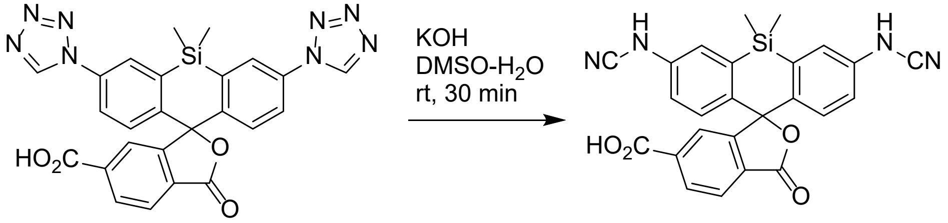

9c **CR3**

KOH (0.1 mL of 1M solution in water) was added to the suspension of **9c** (4.5 mg, 0.009 mmol) in DMSO (0.4 mL), and the reaction mixture was stirred at rt for 30 min. The

reaction mixture was diluted with water (10 mL), with acetic acid to pH 3 and extracted with EtOAc (3x 10 mL). The combined organic layers were evaporated to dryness, and the product was isolated by preparative HPLC (Hypersil Gold C18 250x21.2 mm 5 µm, gradient 30/70 to 60/40 A:B, A = MeCN + 0.1 % formic acid, B = H_2O + 0.1 % formic acid) followed by freeze-drying from dioxane, giving the dye **CR3** as off-white powder (2.1 mg, 50% yield) containing 11 mol% dioxane.

^{1}H NMR (400 MHz, DMSO-d_6): δ 8.14 (dd, *J* = 8.0, 1.3 Hz, 1H), 8.06 (d, *J* = 7.9 Hz, 1H), 7.73 (t, *J* = 1.0 Hz, 1H), 7.30 (d, *J* = 2.5 Hz, 2H), 6.99 (d, *J* = 8.7 Hz, 2H), 6.94 (dd, *J* = 8.7, 2.6 Hz, 2H), 0.67 (s, 3H), 0.57 (s, 3H).
^{13}C NMR (101 MHz, DMSO-d_6): δ 130.4, 128.1, 126.5, 124.2, 119.9, 117.3, -0.6, -1.2 (indirect detection from a gHSQC experiment, only H-coupled carbons are resolved).
HR-MS (ESI) m/z: $[M+H]^+$ 467.1171 (found), 467.1170 (calcd. for $C_{25}H_{18}N_4O_4Si$).

Preparation of self-labelling tag ligands from the dyes CR1-CR3

CR1-Halo

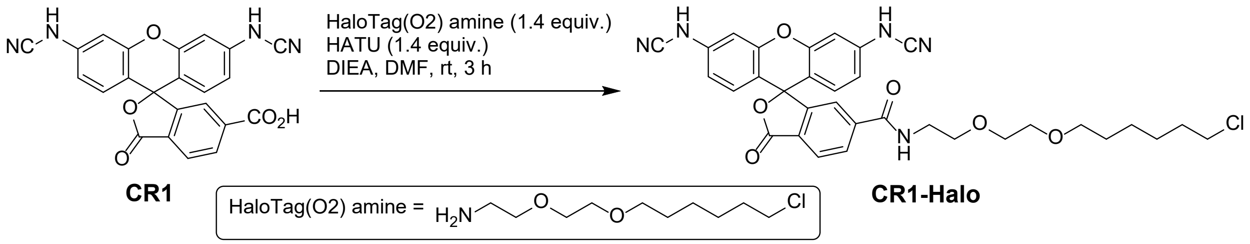

To the mixture of **CR1** (3.8 mg, 0.009 mmol) in DMF (40 µL) and DIEA (*N*,*N*-diisopropylethylamine; 40 µL), the solutions of HATU (1-[bis(dimethylamino)methylene]-1*H*-1,2,3-triazolo[4,5-*b*]pyridinium 3-oxid hexafluorophosphate; 30 µL of 9.2 mg/100 µL stock solution in DMF; 0.012 mmol, 1.4 equiv.) and HaloTag(O2) amine (30 µL of 15.7 mg/100 µL stock solution in DMF; 0.012 mmol, 1.4 equiv.) were added. The reaction mixture was stirred at rt for 3 h. The solvents were removed *in vacuo* and the product was isolated by preparative HPLC (Interchim PhC4 250x21.2 mm 5 µm, gradient 45/55 to 75/25 A:B, A = MeCN + 0.1 % formic acid, B = H_2O + 0.1 % formic acid), followed by freeze-drying from dioxane, to yield **CR1-Halo** HaloTag label as violet powder (2.1 mg, 37% yield).

^{1}H NMR (400 MHz, DMSO-d$_6$): δ 10.69 (s, 2H), 8.73 (t, *J* = 5.5 Hz, 1H), 8.18 (dd, *J* = 8.0, 1.4 Hz, 1H), 8.12 (d, *J* = 8.0 Hz, 1H), 7.69 (s, 1H), 6.90 (d, *J* = 2.3 Hz, 2H), 6.85 (d, *J* = 8.6 Hz, 2H), 6.76 (dd, *J* = 8.6, 2.3 Hz, 2H), 3.59 (t, *J* = 6.6 Hz, 2H), 3.50 – 3.43 (m, 4H), 3.44 – 3.37 (m, 2H), 3.38 – 3.29 (m, 2H), 3.31 – 3.25 (m, 2H), 1.72 – 1.60 (m, 2H), 1.45 – 1.35 (m, 2H), 1.36 – 1.28 (m, 2H), 1.28 – 1.18 (m, 4H).
^{13}C NMR (101 MHz, DMSO-d$_6$): δ 130.2, 129.8, 125.3, 122.4, 112.1, 102.4, 70.1, 69.3, 69.2, 45.1, 39.3, 31.9, 28.9, 28.8, 26.2, 24.8 (indirect detection from a gHSQC experiment, only H-coupled carbons are resolved).
HR-MS (ESI) m/z: $[M+H]^+$ 630.2113 (found), 630.2114 (calcd. for $C_{33}H_{32}ClN_5O_6$).

CR1-BG

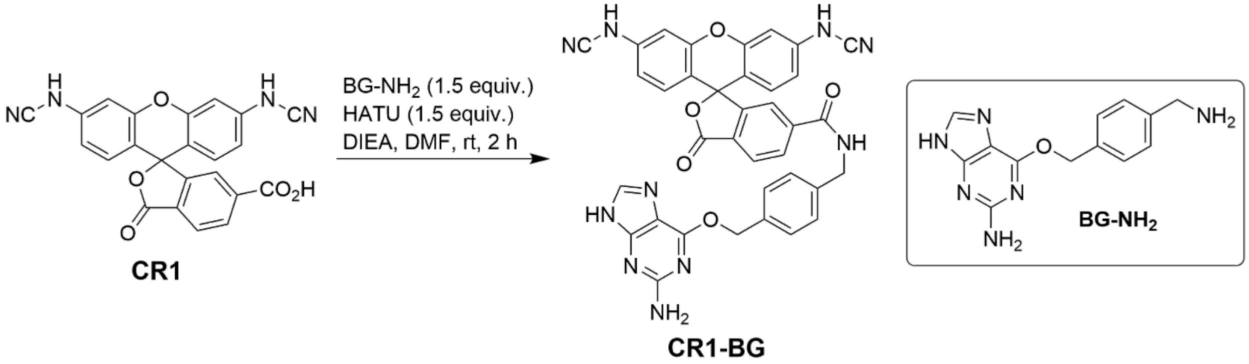

To the mixture of **CR1** (5.0 mg, 0.012 mmol) and BG-NH$_2$ (6-(4-(aminomethyl)benzyloxy)-7*H*-purin-2-amine; 4.8 mg, 0.018 mmol, 1.5 equiv.) in DMF (70 µL) and DIPEA (40 µL), a solution of HATU (30 µL of 22.6 mg/100 µL stock solution in DMF; 0.018 mmol, 1.5 equiv.) was added. The reaction mixture was stirred at rt for 2 h. The solvents were removed *in vacuo* and the product was isolated by preparative HPLC (Interchim PhC4 250x21.2 mm 5 µm, gradient 20/80 to 60/40 A:B, A = MeCN + 0.1 % formic acid, B = H_2O + 0.1 % formic acid), followed by freeze-drying from dioxane, to yield **CR1-BG** SNAP-tag label as violet powder (1.7 mg, 22% yield).

^{1}H NMR (400 MHz, DMSO-d$_6$): δ 12.40 (br s, 1H), 10.68 (br s, 2H), 9.21 (t, *J* = 5.8 Hz, 1H), 8.22 (dd, *J* = 8.0, 1.4 Hz, 1H), 8.12 (d, *J* = 8.0 Hz, 1H), 7.79 (br s, 1H), 7.73 (s, 1H), 7.43 (d, *J* = 7.8 Hz, 2H), 7.28 (d, *J* = 7.8 Hz, 2H), 6.93 – 6.81 (m, 3H), 6.75 (dd, *J* = 8.6, 2.3 Hz, 2H), 6.25 (br s, 2H), 5.42 (s, 2H), 4.42 (d, *J* = 5.8 Hz, 2H).

^{13}C NMR (101 MHz, DMSO-d_6): δ 130.2, 129.8, 128.6, 127.7, 125.2, 122.4, 112.0, 102.4, 66.5, 42.6 (indirect detection from a gHSQC experiment, only H-coupled carbons are resolved).
HR-MS (ESI) m/z: $[M+H]^+$ 677.2002 (found), 677.2004 (calcd. for $C_{36}H_{24}N_{10}O_5$).

9b-Halo

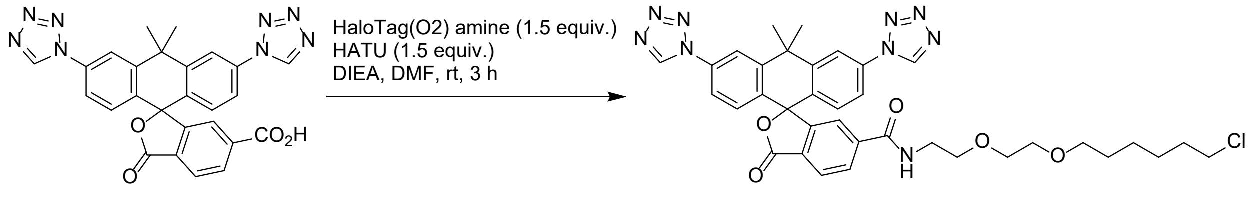

9b 9b-Halo

To the mixture of **9b** (5.5 mg, 0.011 mmol) in DMF (40 µL) and DIEA (40 µL), the solutions of HATU (30 µL of 20.8 mg/100 µL stock solution in DMF; 0.016 mmol, 1.5 equiv.) and HaloTag(O2) amine (30 µL of 12.4 mg/100 µL stock solution in DMF; 0.017 mmol, 1.5 equiv.) were added. The reaction mixture was stirred at rt for 3 h. The solvents were removed *in vacuo* and the product was isolated by preparative HPLC (Interchim PhC4 250x21.2 mm 5 µm, gradient 45/55 to 75/25 A:B, A = MeCN + 0.1 % formic acid, B = H_2O + 0.1 % formic acid) followed by freeze-drying from dioxane, giving **9b-Halo** as off-white powder (5.8 mg, 78% yield).

^1H NMR (400 MHz, DMSO-d_6): δ 10.24 (s, 2H), 8.82 (t, *J* = 5.6 Hz, 1H), 8.45 (d, *J* = 2.3 Hz, 2H), 8.22 (dd, *J* = 8.1, 0.7 Hz, 1H), 8.17 (dd, *J* = 8.1, 1.3 Hz, 1H), 7.80 (dd, *J* = 8.7, 2.2 Hz, 2H), 7.49 (t, *J* = 1.0 Hz, 1H), 7.13 (d, *J* = 8.7 Hz, 2H), 3.57 (t, *J* = 6.6 Hz, 2H), 3.46 – 3.41 (m, 4H), 3.40 – 3.36 (m, 2H), 3.34 – 3.27 (m, 2H), 3.26 (t, *J* = 6.5 Hz, 2H), 2.05 (s, 3H), 1.93 (s, 3H), 1.69 – 1.58 (m, 2H), 1.41 – 1.32 (m, 2H), 1.32 – 1.25 (m, 2H), 1.25 – 1.16 (m, 2H).
^{13}C NMR (101 MHz, DMSO-d_6): δ 168.8, 164.6, 154.4, 146.2, 142.6, 141.2, 134.8, 131.1, 129.8, 129.6, 126.7, 125.9, 121.8, 120.4, 120.0, 83.7, 70.1, 69.5, 69.4, 68.6, 45.4, 38.6, 33.8, 33.6, 32.0, 29.0, 26.1, 24.9.
HR-MS (ESI) m/z: $[M+H]^+$ 712.2750 (found), 712.2757 (calcd. for $C_{36}H_{38}ClN_9O_5$).

CR2-Halo

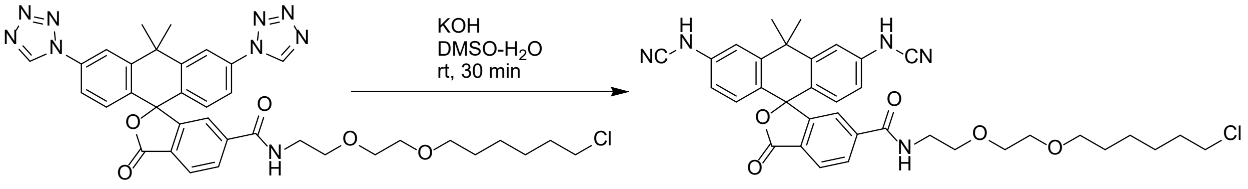

9b-Halo CR2-Halo

KOH (0.2 mL of 1M solution in water) was added to the solution of **9b-Halo** (5.8 mg, 0.008 mmol) in DMSO (0.8 mL), and the reaction mixture was stirred at rt for 30 min. The reaction mixture was diluted with water (10 mL), with acetic acid to pH 3 and extracted with EtOAc (4x 10 mL). The combined organic layers were evaporated to dryness, and the product was isolated by preparative HPLC (Interchim PhC4 250x21.2 mm 5 µm, gradient 45/55 to 75/25 A:B, A = MeCN + 0.1 % formic acid, B = H_2O + 0.1 % formic acid), followed by freeze-drying from dioxane, to yield **CR2-Halo** HaloTag label as light-pink powder (4.8 mg, 90% yield).

^{1}H NMR (400 MHz, DMSO-d$_6$): δ 10.45 (br s, 2H), 8.79 (t, *J* = 5.6 Hz, 1H), 8.14 (dd, *J* = 8.1, 1.4 Hz, 1H), 8.11 (d, *J* = 8.1 Hz, 1H), 7.46 (s, 1H), 7.25 (d, *J* = 2.4 Hz, 2H), 6.83 (dd, *J* = 8.6, 2.4 Hz, 2H), 6.71 (d, *J* = 8.6 Hz, 2H), 3.58 (t, *J* = 6.6 Hz, 2H), 3.51 – 3.42 (m, 4H), 3.43 – 3.36 (m, 2H), 3.28 (t, *J* = 6.5 Hz, 2H), 1.81 (s, 3H), 1.71 – 1.59 (m, 5H), 1.45 – 1.35 (m, 2H), 1.38 – 1.26 (m, 2H), 1.28 – 1.18 (m, 2H).
^{13}C NMR (101 MHz, DMSO-d$_6$): δ 129.7, 129.3, 125.4, 122.0, 114.8, 112.5, 70.1, 69.4, 69.2, 45.3, 39.3, 34.5, 32.7, 32.0, 28.9, 26.0, 25.2 (indirect detection from a gHSQC experiment, only H-coupled carbons are resolved).
HR-MS (ESI) m/z: $[M+H]^+$ 656.2630 (found), 656.2634 (calcd. for $C_{36}H_{38}ClN_5O_5$).

9c-Halo

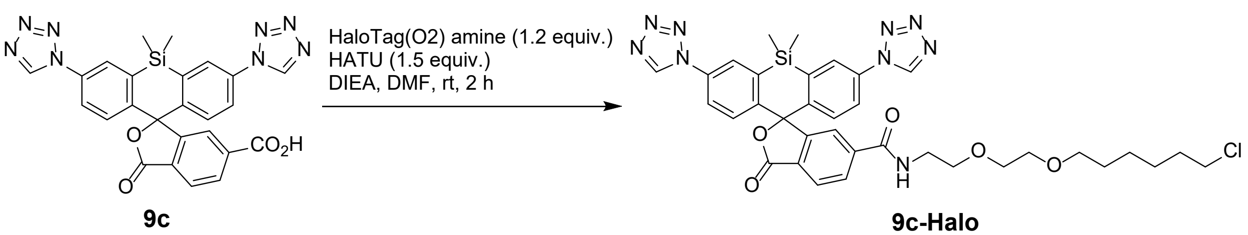

9c 9c-Halo

To the mixture of **9c** (7.6 mg, 0.014 mmol) in DMF (40 µL) and DIEA (40 µL), the solutions of HATU (30 µL of 26.6 mg/100 µL stock solution in DMF; 0.021 mmol, 1.5 equiv.) and HaloTag(O2) amine (30 µL of 13.0 mg/100 µL stock solution in DMF; 0.017 mmol, 1.2 equiv.) were added. The reaction mixture was stirred at rt for 2 h. The solvents were removed *in vacuo* and the product was isolated by preparative HPLC (Interchim PhC4 250x21.2 mm 5 µm, gradient 50/50 to 80/20 A:B, A = MeCN + 0.1 % formic acid, B = H_2O + 0.1 % formic acid) followed by freeze-drying from dioxane, giving **9c-Halo** as off-white powder (3.5 mg, 33% yield).

^{1}H NMR (400 MHz, $CDCl_3$): δ 9.05 (s, 2H), 8.12 – 8.05 (m, 3H), 7.91 (dd, *J* = 8.0, 1.3 Hz, 1H), 7.87 (dd, *J* = 1.3, 0.7 Hz, 1H), 7.65 (dd, *J* = 8.7, 2.4 Hz, 2H), 7.44 (d, *J* = 8.7 Hz, 2H), 6.97 – 6.89 (m, 1H), 3.67 – 3.61 (m, 6H), 3.57 (ddd, *J* = 7.0, 3.2, 1.4 Hz, 2H), 3.50 (t, *J* = 6.6 Hz, 2H), 3.43 (t, *J* = 6.7 Hz, 2H), 1.76 – 1.65 (m, 2H), 1.53 (p, *J* = 6.9 Hz, 2H), 1.45 – 1.35 (m, 2H), 1.35 – 1.21 (m, 2H), 0.91 (s, 3H), 0.77 (s, 3H).
^{13}C NMR (101 MHz, $CDCl_3$): δ 169.0, 165.5, 153.7, 145.5, 141.0, 140.6, 137.8, 133.8, 128.4, 128.0, 127.1, 126.8, 126. 8, 123.7, 122.9, 88. 7, 71.4, 70.3, 70.1, 69.5, 45.2, 40.2, 32.5, 29.5, 26.7, 25.5, 0.2, -0.6.
HR-MS (ESI) m/z: $[M+H]^+$ 728.2523 (found), 728.2526 (calcd. for $C_{35}H_{38}ClN_9O_5Si$).

CR3-Halo

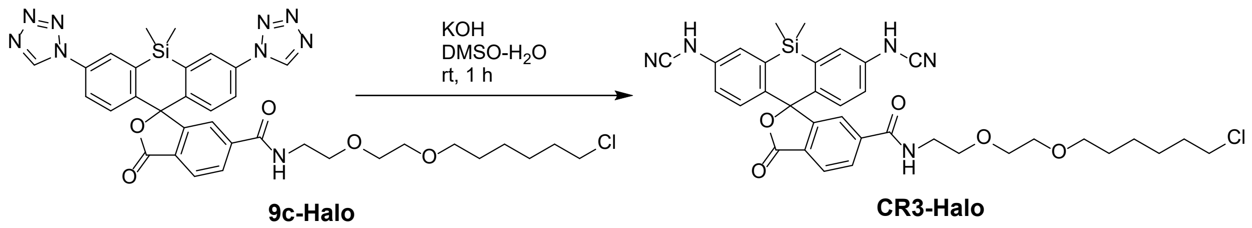

KOH (0.1 mL of 1M solution in water) was added to the solution of **9c-Halo** (3.5 mg, 0.008 mmol) in DMSO (0.4 mL), and the reaction mixture was stirred at rt for 1 h. The reaction mixture was diluted with water (10 mL), with acetic acid to pH 3 and extracted with EtOAc (3x 10 mL). The combined organic layers were evaporated to dryness, and the product was isolated by preparative HPLC (Interchim PhC4 250x21.2 mm 5 µm, gradient 45/55 to 75/25 A:B, A = MeCN + 0.1 % formic acid, B = H_2O + 0.1 % formic acid), followed by

freeze-drying from dioxane, to yield **CR3-Halo** HaloTag label as light-pink powder (1.9 mg, 55% yield).

^{1}H NMR (400 MHz, DMSO-d_6): δ 10.41 (s, 2H), 8.81 (t, *J* = 5.6 Hz, 1H), 8.11 (dd, *J* = 8.0, 1.3 Hz, 1H), 8.08 (dd, *J* = 8.0, 0.8 Hz, 1H), 7.72 (t, *J* = 1.1 Hz, 1H), 7.31 (t, *J* = 1.6 Hz, 2H), 6.97 – 6.94 (m, 4H), 3.57 (t, *J* = 6.6 Hz, 2H), 3.53 – 3.47 (m, 4H), 3.45 – 3.41 (m, 2H), 3.40 – 3.36 (m, 2H), 3.31 – 3.27 (m, 2H), 1.69 – 1.60 (m, 2H), 1.44 – 1.34 (m, 2H), 1.34 – 1.27 (m, 2H), 1.26 – 1.19 (m, 2H), 0.68 (s, 3H), 0.57 (s, 3H).
^{13}C NMR (101 MHz, DMSO-d_6): δ 128.8, 128.4, 126.1, 122.8, 119.7, 117.0, 70.0, 69.5, 69.3, 45.3, 39.4, 31.9, 28.8, 26.0, 24.7, -0.5, -1.7 (indirect detection from a gHSQC experiment, only H-coupled carbons are resolved).
HR-MS (ESI) m/z: $[M+H]^+$ 672.2407 (found), 672.2404 (calcd. for $C_{35}H_{38}ClN_5O_5Si$).

Supplementary References

[S1] Grimm, J.B.; English, B.P.; Chen, J.; Slaughter, J.P.; Zhang, Z.; Revyakin, A.; Patel, R.; Macklin, J.J.; Normanno, D.; Singer, R.H.; Lionnet, T.; Lavis, L.D. *Nat. Methods* **2015**, *12*, 244–250.

[S2] Dickinson, B.C.; Huynh, C.; Chang, C.J. *J. Am. Chem. Soc.* **2010**, *132*, 5906–5915.

[S3] Critchfield, F.E.; Gibson Jr., J.A.; Hall, J.L. *J. Am. Chem. Soc.*, **1953**, *75(8)*, 1991–1992.

[S4] Uno, K.; Bossi, M.L.; Konen, T.; Belov, V.N.; Irie, M.; Hell, S.W. *Adv. Opt. Mater.* **2019**, *7*, 1801746.

[S5] Deniel, M.H.; Lavabre, D.; Micheau, J.C. in *Organic Photochromic and Thermochromic Compounds* (eds. John C. Crano & Robert J. Guglielmetti), Ch. 4, 167-209 (Springer US, **2002**).

[S6] Kaushik, S.; Prokop, Z.; Damborsky, J.; Chaloupkova, R. *FEBS J.* **2017**, *284(1)*, 134–148.

[S7] Wilhelm, J; Kühn, S.; Tarnawski, M.; Gotthard, G.; Tünnermann, J.; Tänzer, T.; Karpenko, J.; Mertes, N.; Xue, L.; Uhrig, U.; Reinstein, J.; Hublot, J.; Johnsson, K. *Biochemistry*, **2021**, *60*, 2560–2575.

[S8] Butkevich, A.N.; Mitronova, G.Y.; Sidenstein, S.C.; Klocke, J.L.; Kamin, D.; Meineke, D.N.H.; D'Este, E.; Kraemer, P.-T.; Danzl, J.G.; Belov, V.N.; Hell, S.W. *Angew. Chem. Int. Ed.*, **2016**, *55*, 3290–3294.

[S9] Thevathasan, J.V.; Kahnwald, M.; Cieslinski, K.; Hoess, P.; Peneti, S.K.; Reitberger, M.; Heid, D.; Kasuba, K.C.; Hoerner, S.J.; Li, Y.M.; Wu, Y.L.; Mund, M.; Matti, U.; Pereira, P.M.; Henriques, R.; Nijmeijer, B.; Kueblbeck, M.; Jimenez Sabinina, V.; Ellenberg, J.; Ries. J. *Nat. Methods*, **2019**, *16*, 1045–1053.

[S10] Jegou, T.; Chung, I.; Heuvelman, G.; Wachsmuth, M.; Gorisch, S.M.; K.M. Greulich-Bode; Boukamp, P.; Lichter, P.; Rippe, K. *Mol. Biol. Cell*, **2009**, *20*, 2070–2082.

[S11] Lukinavičius, G.; Blaukopf, C.; Pershagen, E.; Schena, A.; Reymond, L.; Derivery, E.; Gonzalez-Gaitan, M.; D'Este, E.; Hell, S.W.; Gerlich, D.W.; Johnsson, K. *Nat. Commun.* **2015**, *6*, 8497.

[S12] Butkevich, A.N.; Belov, V.N.; Kolmakov, K.; Sokolov, V.V.; Shojaei, H.; Sidenstein, S.C.; Kamin, D.; Matthias, J.; Vlijm, R.; Engelhardt, J.; Hell, S.W. *Chem. Eur. J.* **2017**, *23*, 12114–12119.

[S13] Bolte, S.; Cordelières, F.P. *J. Microsc.* **2006**, *224*, 213–232.

[S14] Schneider, C.A.; Rasband, W.S.; Eliceiri, K.W. *Nat. Methods*, **2012**, *9*, 671–675.

[S15] Grimm, J.B.; Lavis, L.D. *Org. Lett.* **2011**, *13*, 6354–6357.

[S16] Butkevich, A.N.; Weber, M.; Cereceda Delgado, A.R.; Ostersehlt, L.M.; D'Este, E.; Hell, S.W. *J. Am. Chem. Soc.* **2021**, *143*, 18388–18393.

[S17] Peng, T.; Yang, D. *Org. Lett.* **2010**, *12(3)*, 496–499.

[S18] Grimm, J.B.; Sung, A.J.; Legant, W.R.; Hulamm, P.; Matlosz, S.M.; Betzig, E.; Lavis, L.D. *ACS Chem. Biol.* **2013**, *8(6)*, 1303–1310.

[S19] Butkevich, A.N.; Bossi, M.L.; Lukinavičius, G.; Hell, S.W. *J. Am. Chem. Soc.* **2019**, *141(2)*, 981–989.

NMR spectra

S13

1H (400.15 MHz, CDCl3)

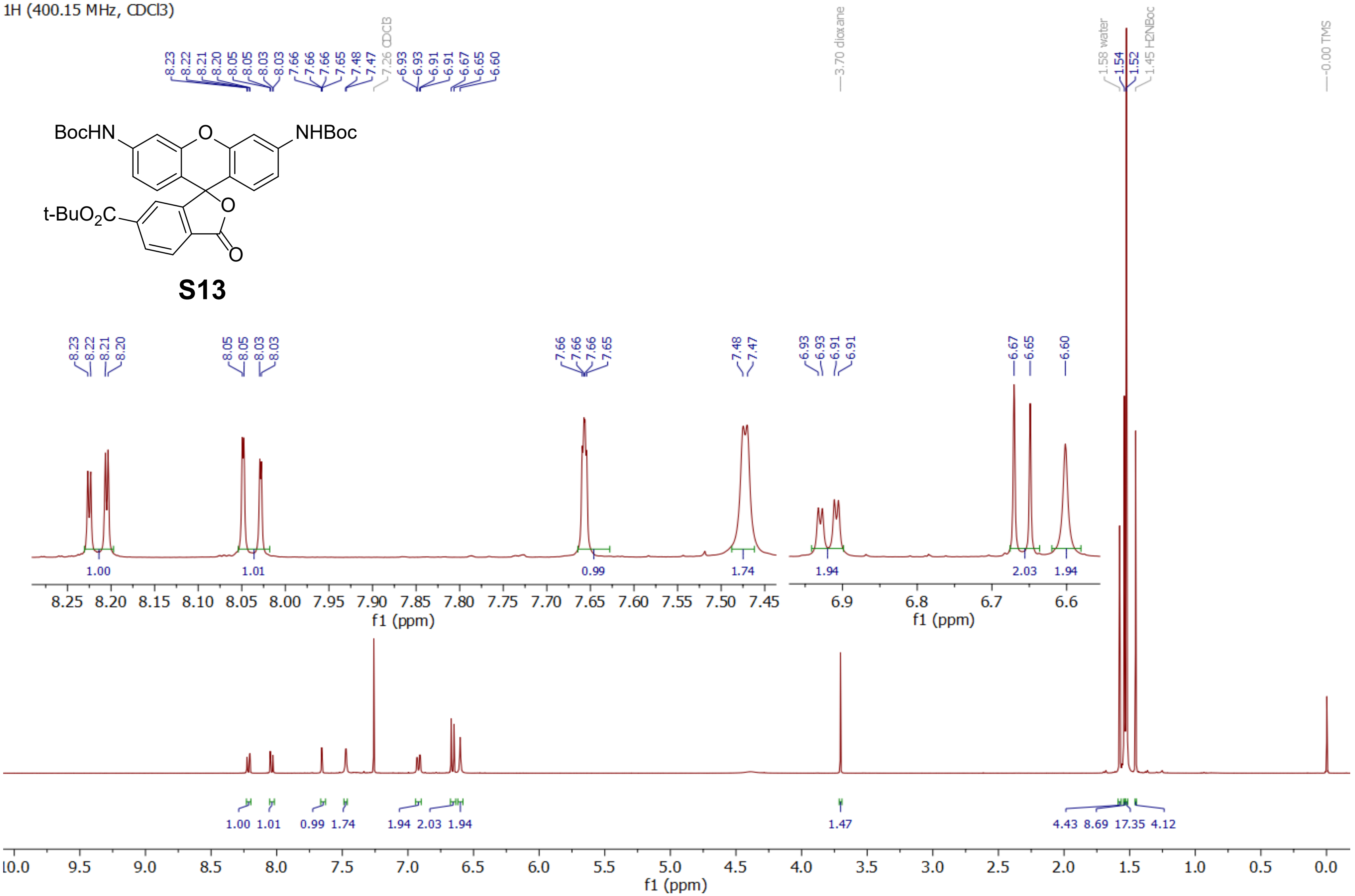

S14

1H (400.15 MHz, CDCl3)

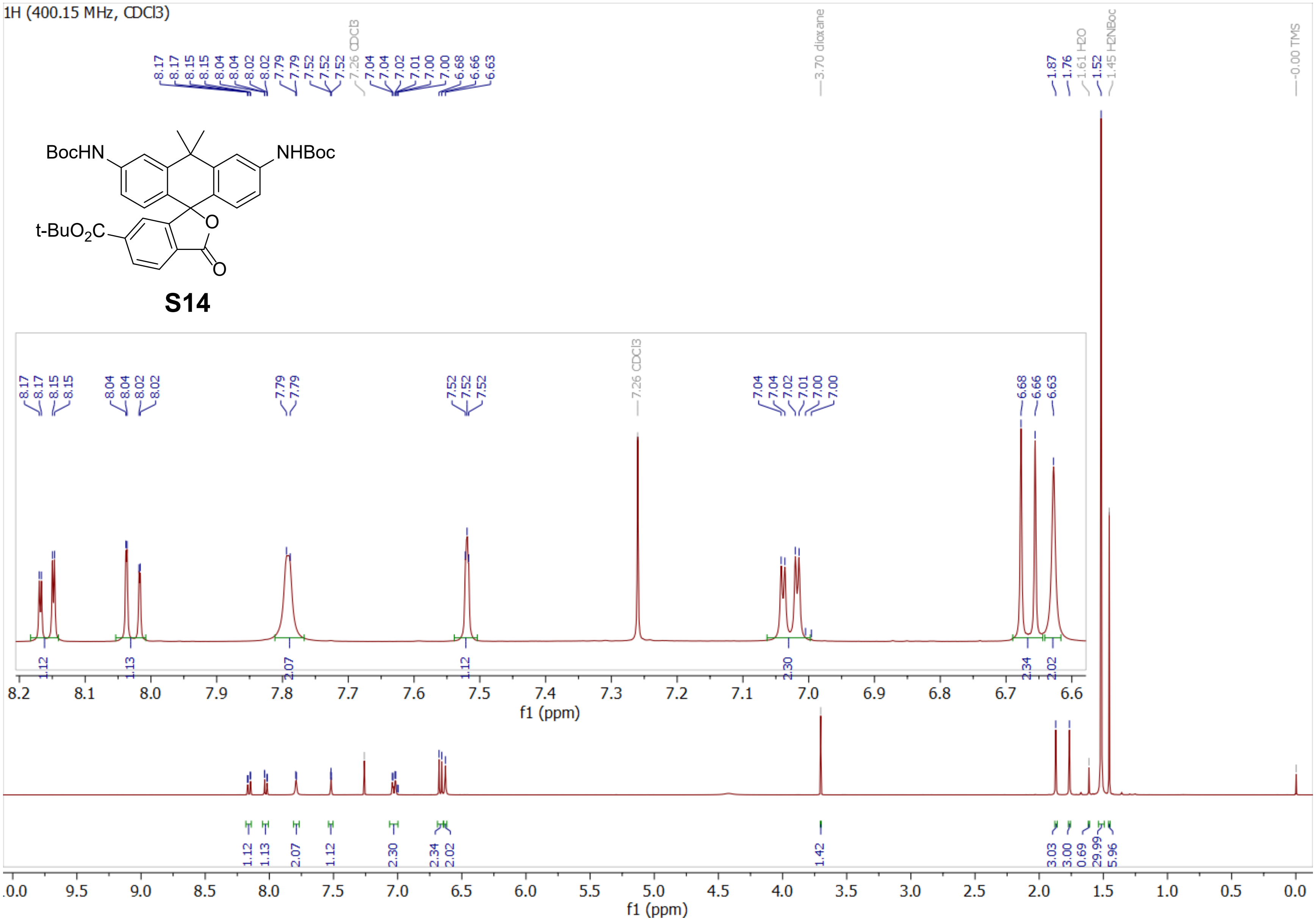

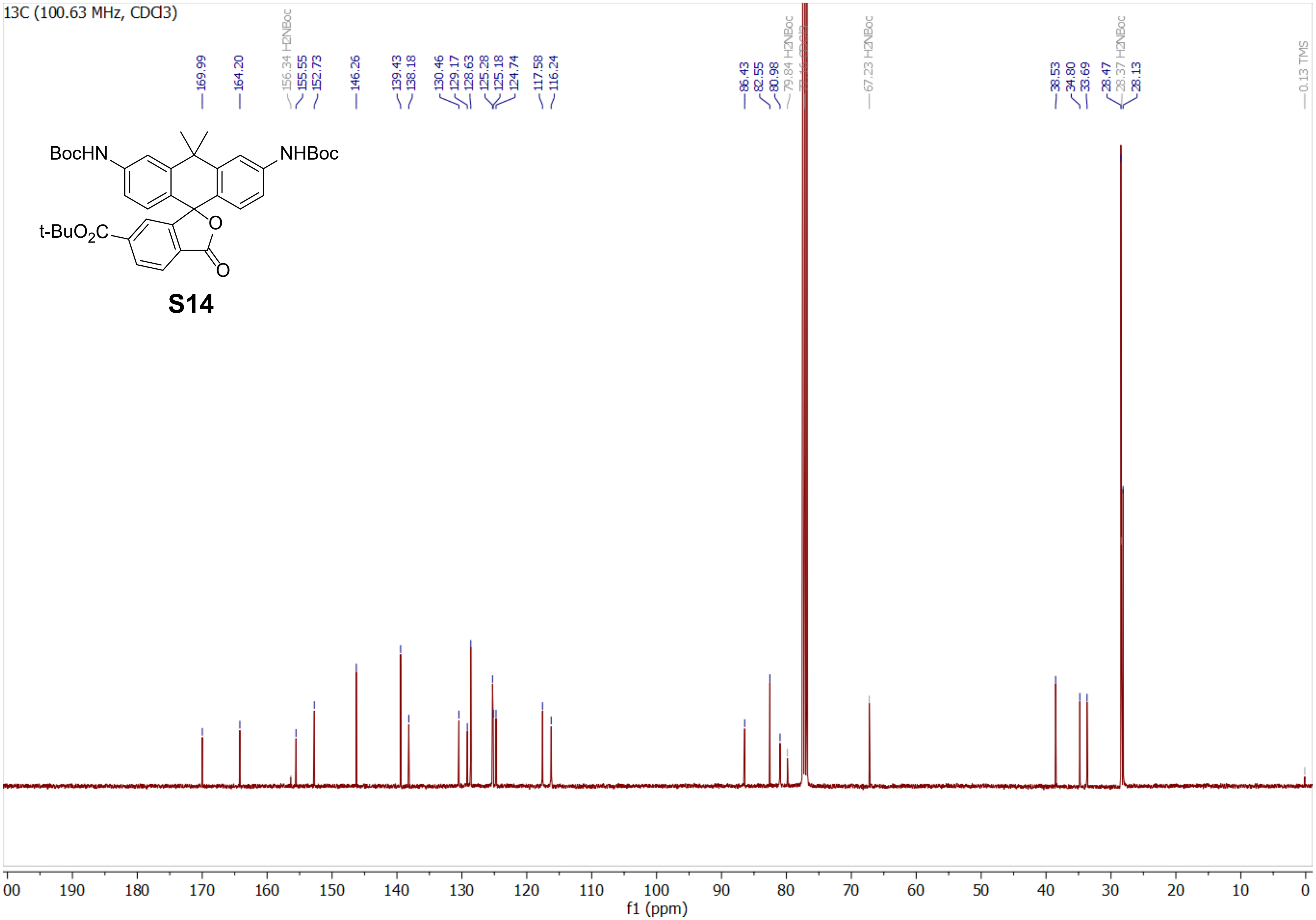

13C (100.63 MHz, CDCl3)
169.99
164.20
156.34 H2NBoc
155.55
152.73
146.26
139.43
138.18
130.46
129.17
128.63
125.28
125.18
124.74
117.58
116.24
86.43
82.55
80.98
79.84 H2NBoc
67.23 H2NBoc
38.53
34.80
33.69
28.47
28.37 H2NBoc
28.13
0.13 TMS
BocHN
NHBoc
t-BuO2C
O
O
S14
f1 (ppm)

S15

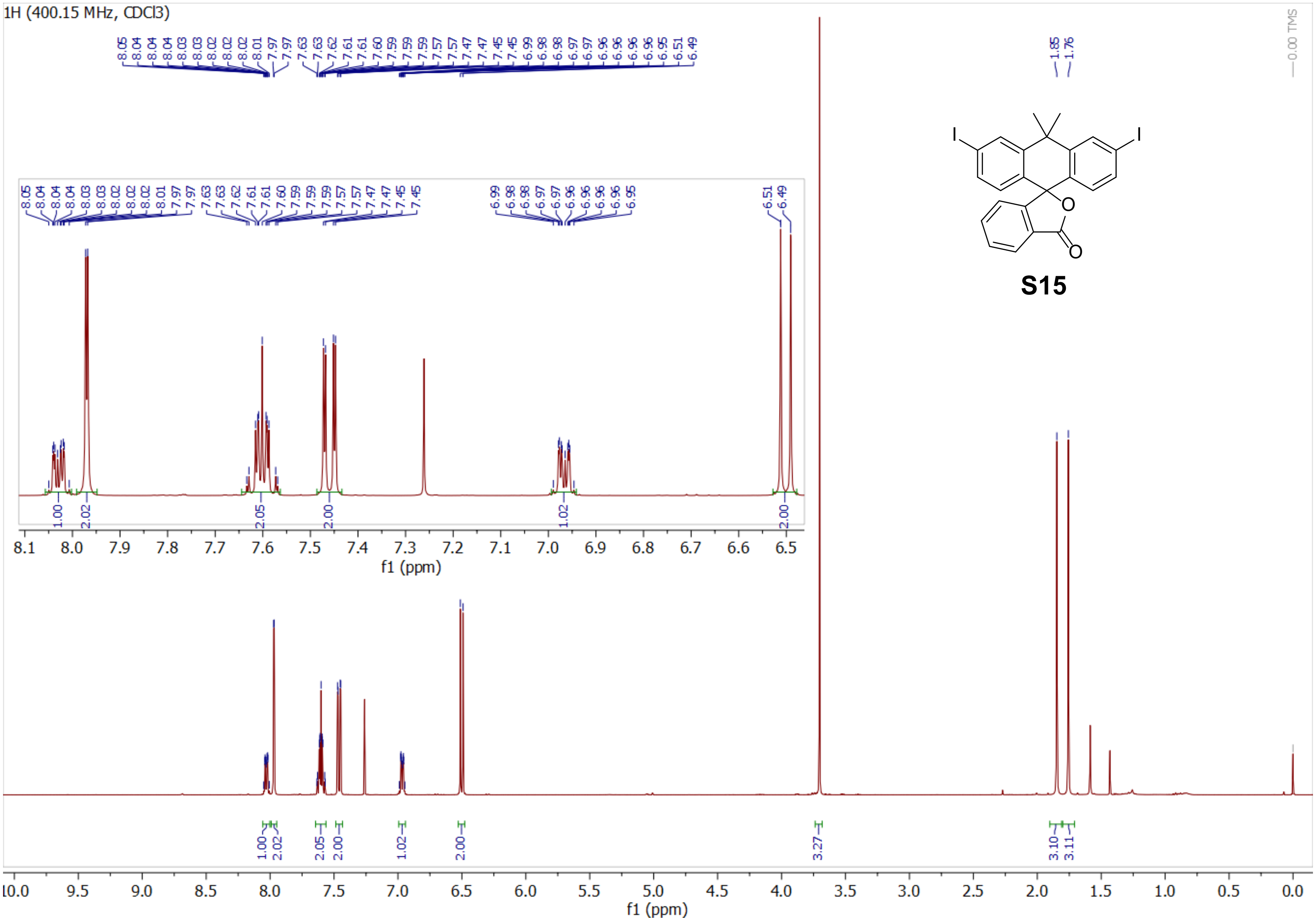

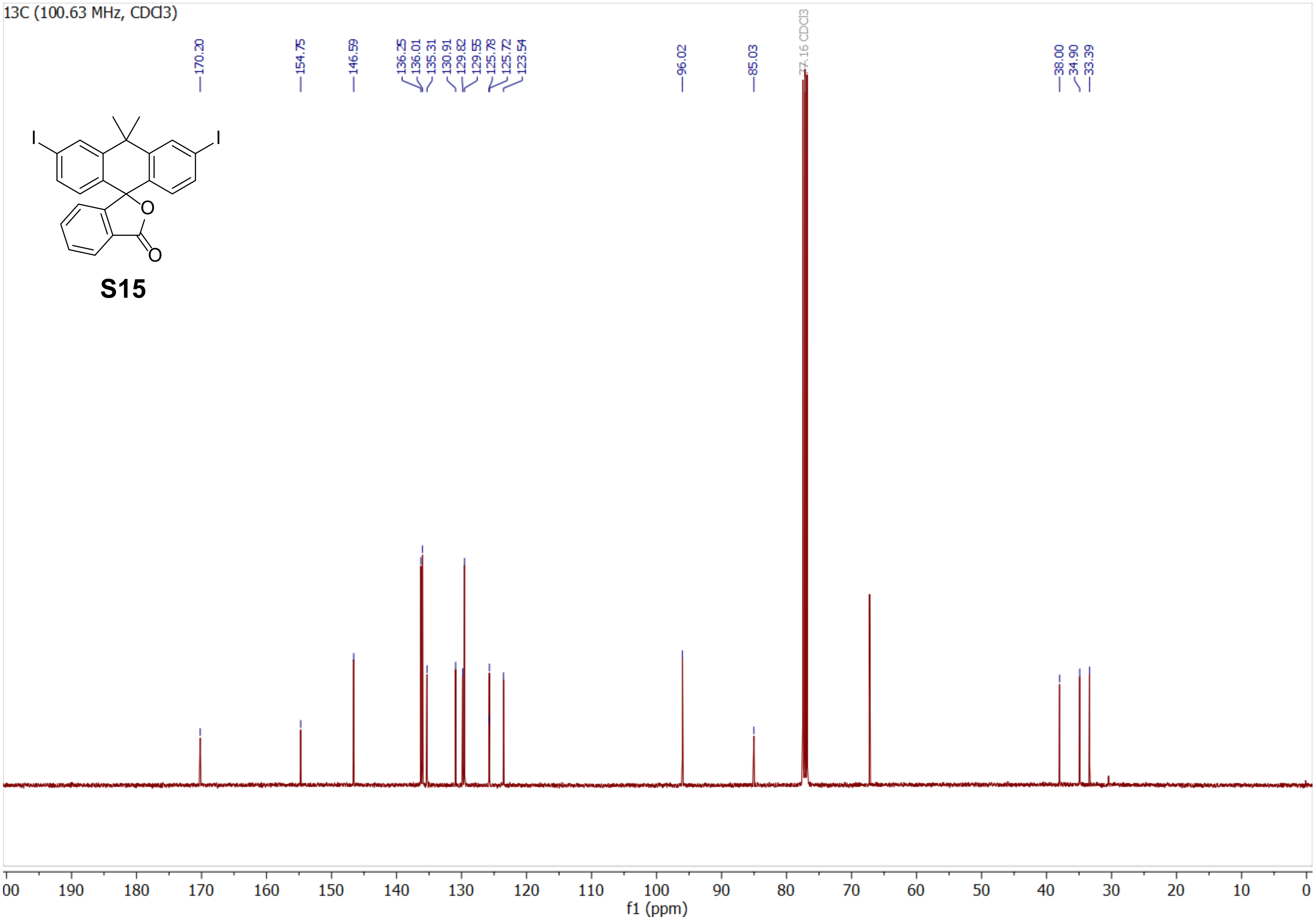
13C (100.63 MHz, CDCl3)
170.20
154.75
146.59
136.25
136.01
135.31
130.91
129.82
129.55
125.78
125.72
123.54
96.02
85.03
77.16 CDCl3
38.00
34.90
33.39
I
I
O
O
S15
00
190
180
170
160
150
140
130
120
110
100
90
80
70
60
50
40
30
20
10
0
f1 (ppm)

S16

1H (400.15 MHz, DMSO)

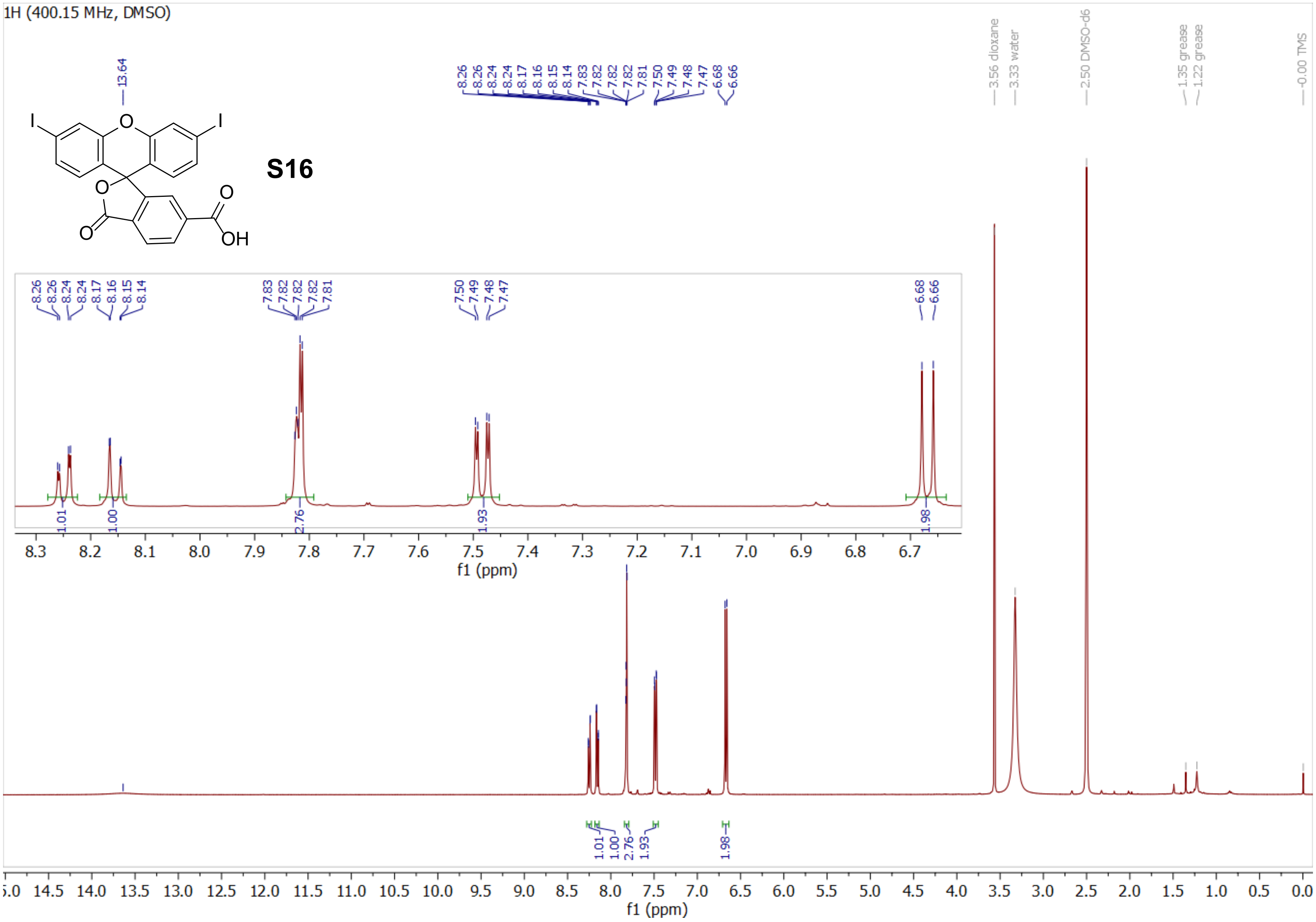

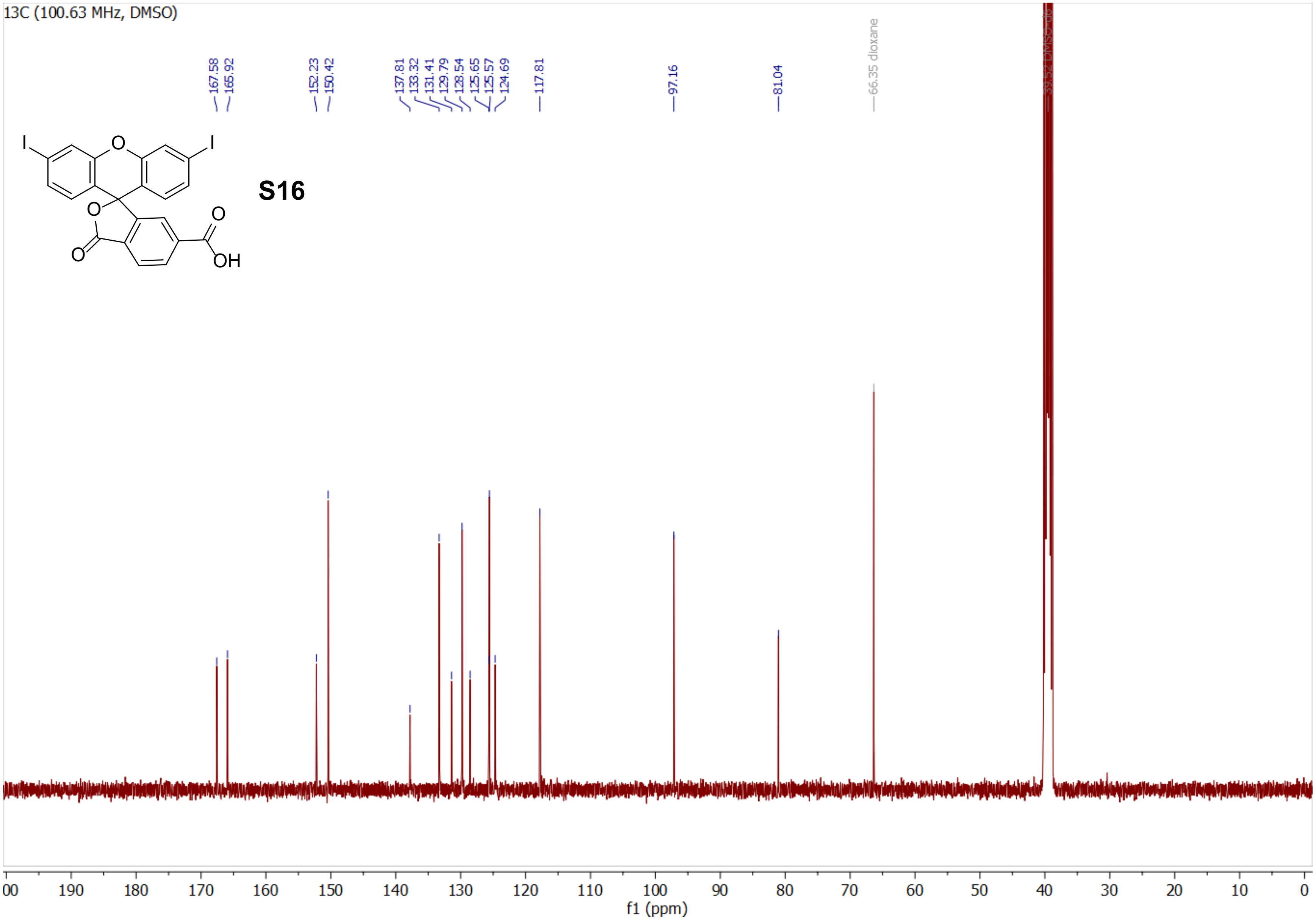
13C (100.63 MHz, DMSO)
167.58
165.92
152.23
150.42
137.81
133.32
131.41
129.79
128.54
125.65
125.57
124.69
117.81
97.16
81.04
66.35 dioxane
39.52 DMSO-d6
I
O
I
O
O
O
OH
S16
00
190
180
170
160
150
140
130
120
110
100
90
80
70
60
50
40
30
20
10
0
f1 (ppm)

1a

1H (400.15 MHz, CDCl3)

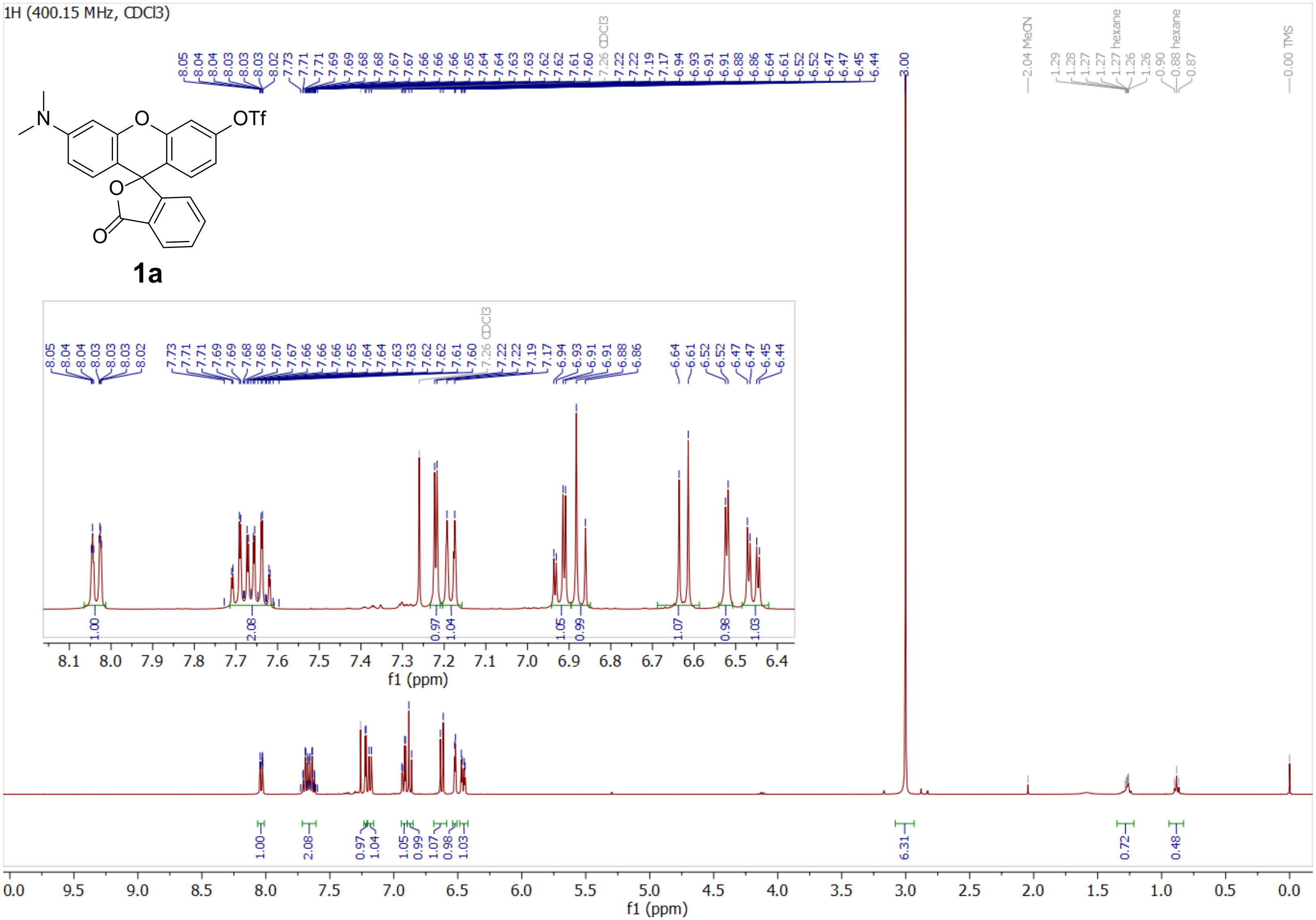

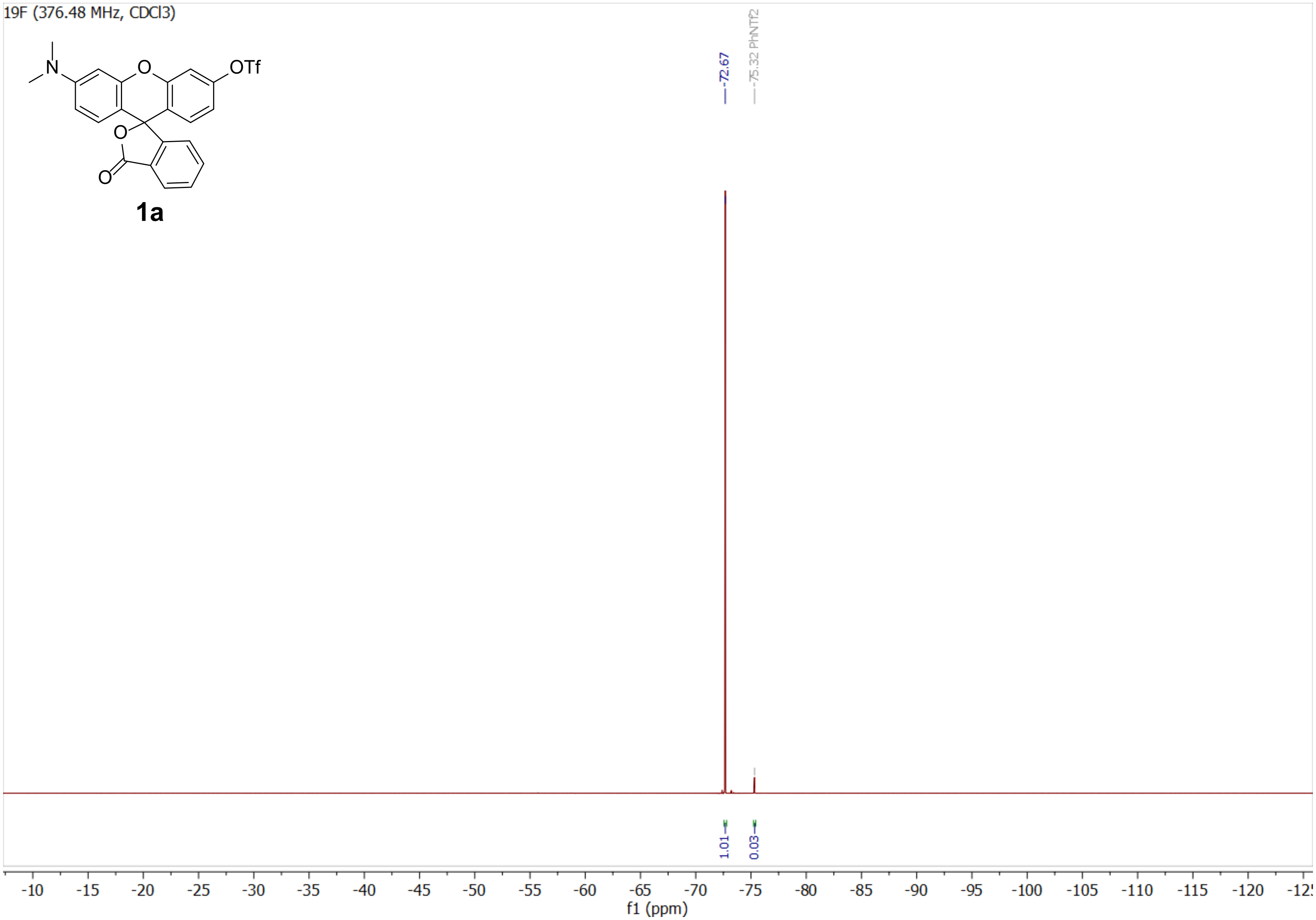
19F (376.48 MHz, CDCl3)
OTf
1a
-72.67
-75.32 PhNTf2
1.01
0.03
-10 -15 -20 -25 -30 -35 -40 -45 -50 -55 -60 -65 -70 -75 -80 -85 -90 -95 -100 -105 -110 -115 -120
f1 (ppm)

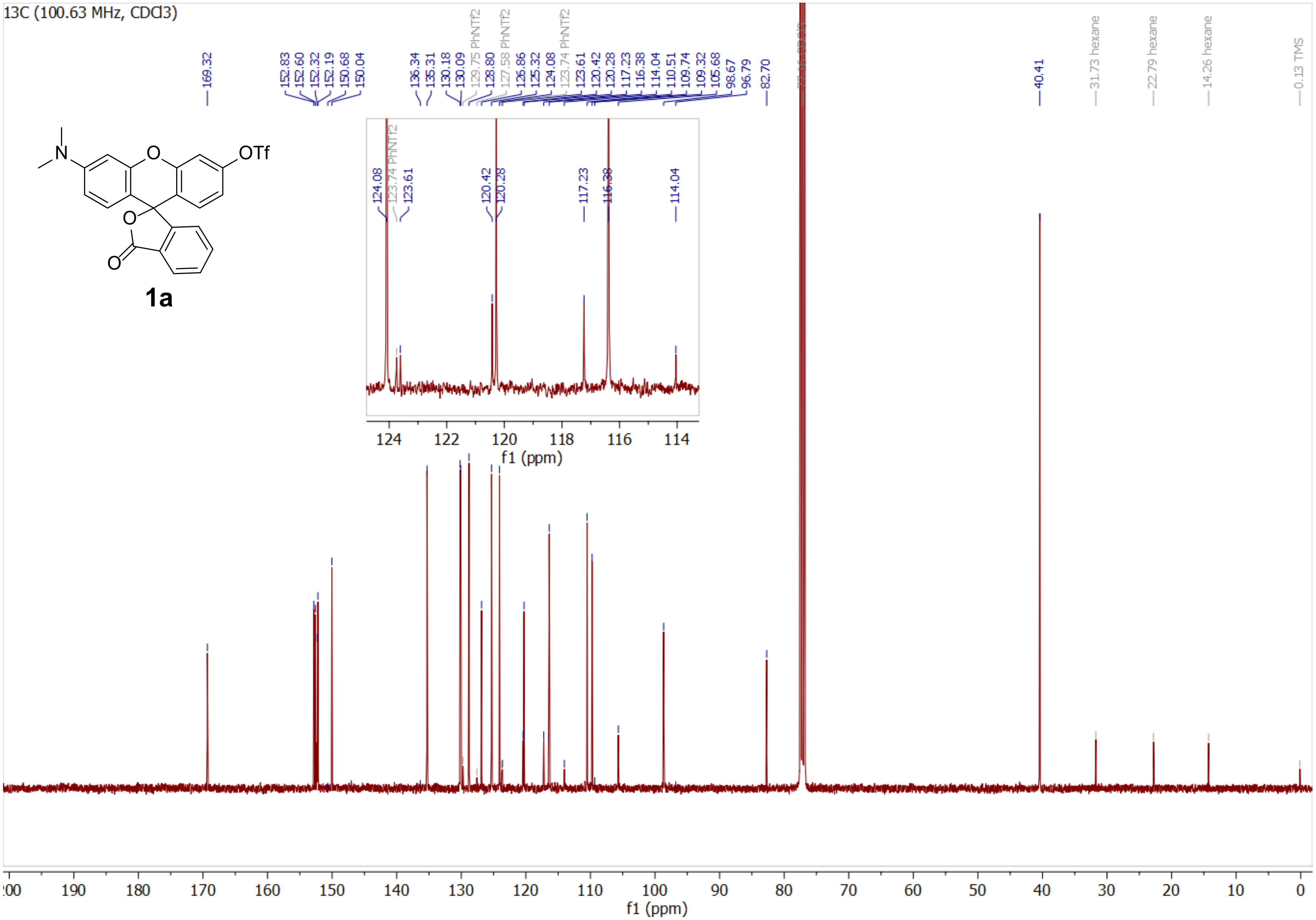
1a
13C (100.63 MHz, CDCl3)
169.32
152.83
152.60
152.32
152.19
150.68
150.04
136.34
135.31
130.18
130.09
129.75 PhNTf2
128.80
127.58 PhNTf2
126.86
125.32
124.08
123.74 PhNTf2
123.61
120.42
120.28
117.23
116.38
114.04
110.51
109.74
109.32
105.68
98.67
96.79
82.70
40.41
31.73 hexane
22.79 hexane
14.26 hexane
0.13 TMS
f1 (ppm)

3a

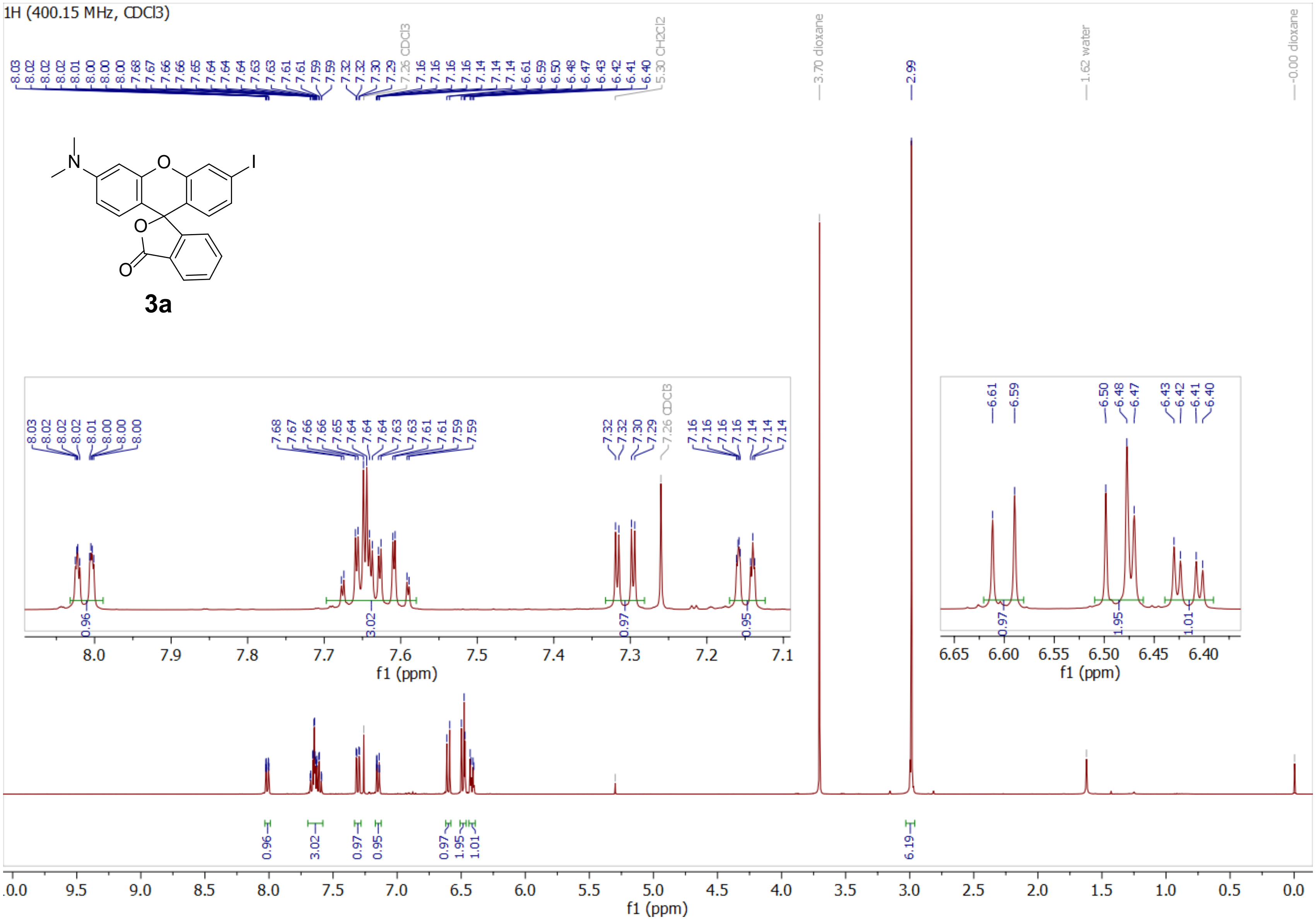

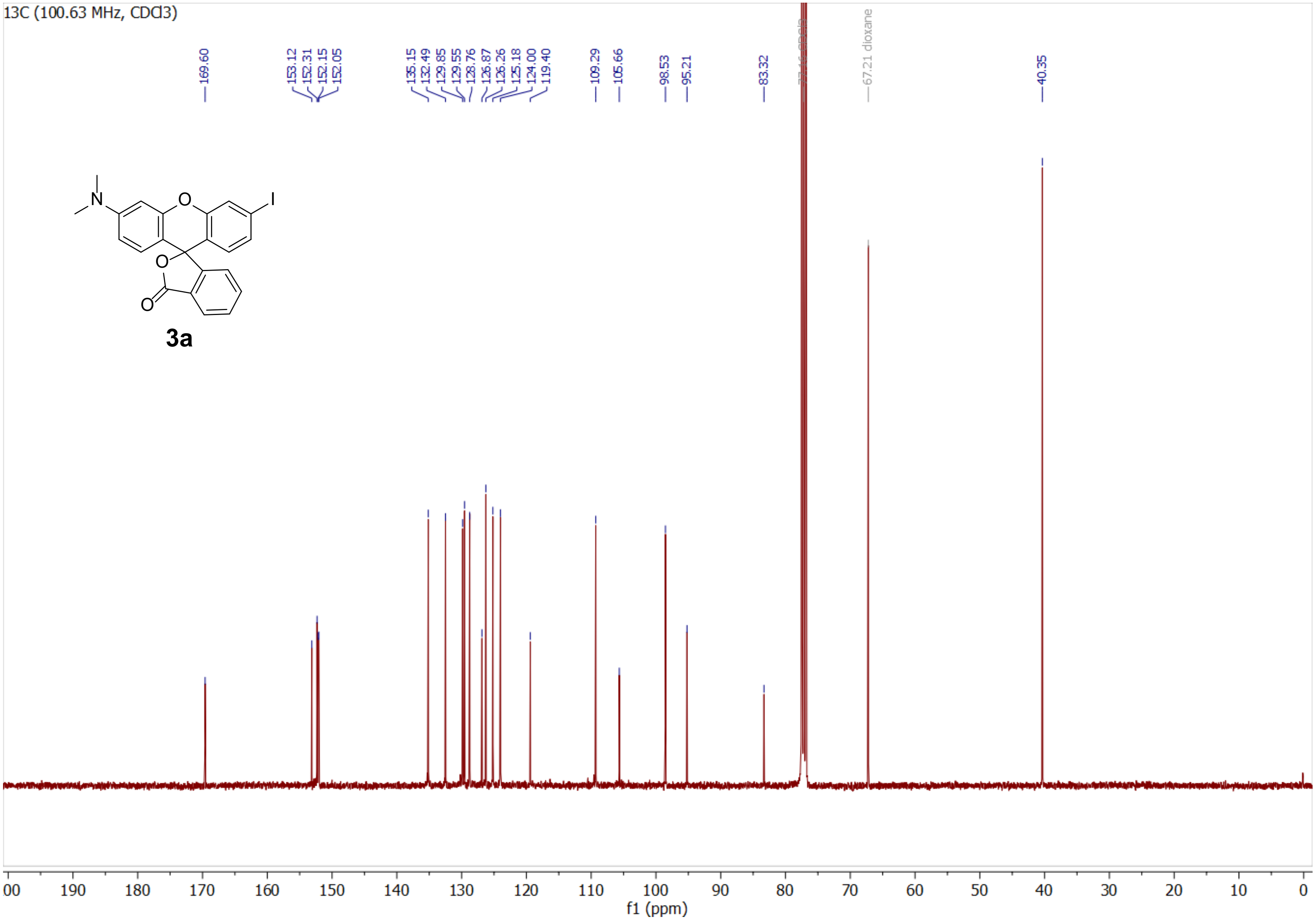
13C (100.63 MHz, CDCl3)
169.60
153.12
152.31
152.15
152.05
135.15
132.49
129.85
129.55
128.76
126.87
126.26
125.18
124.00
119.40
109.29
105.66
98.53
95.21
83.32
67.21 dioxane
40.35
3a
f1 (ppm)

5a

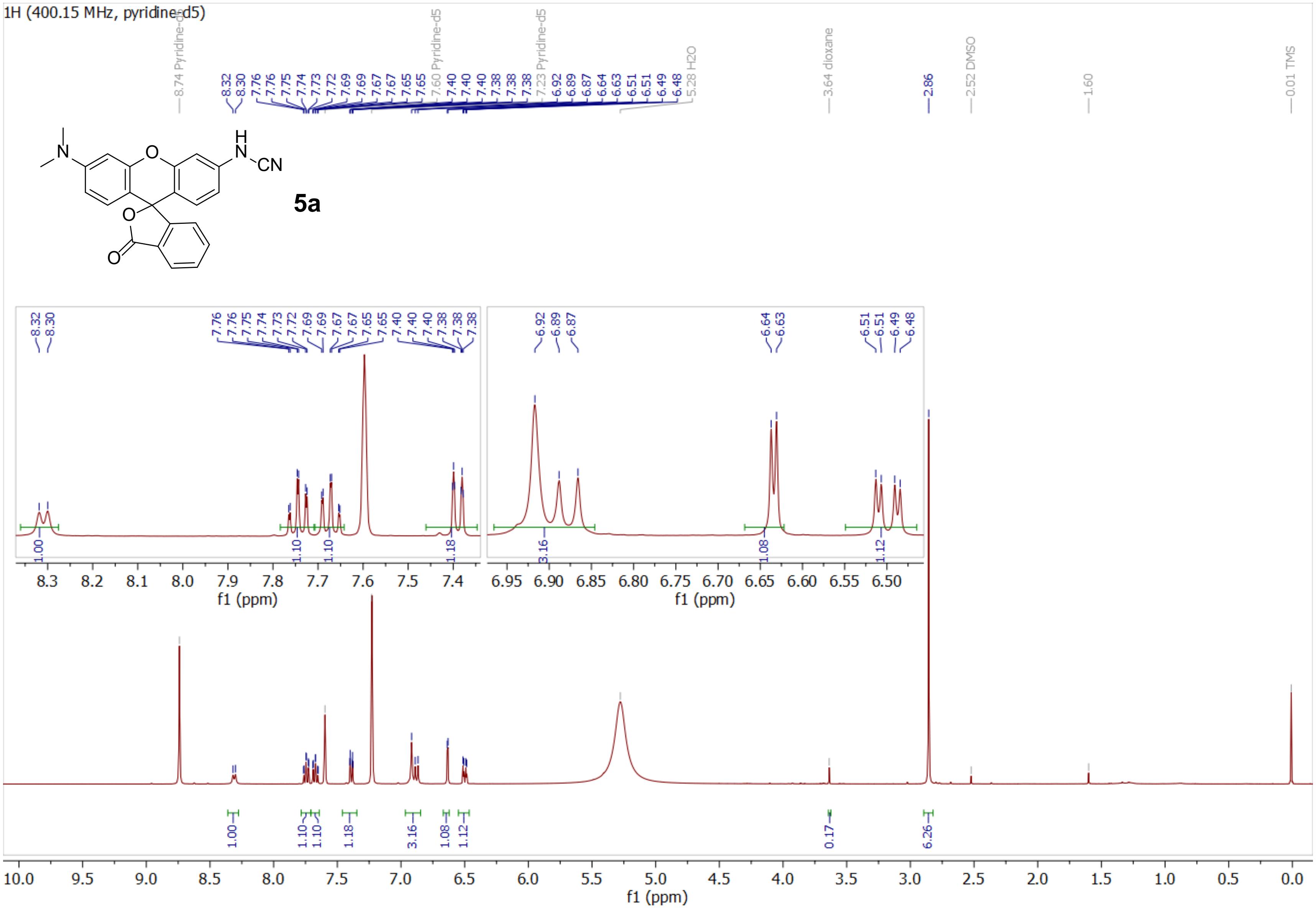

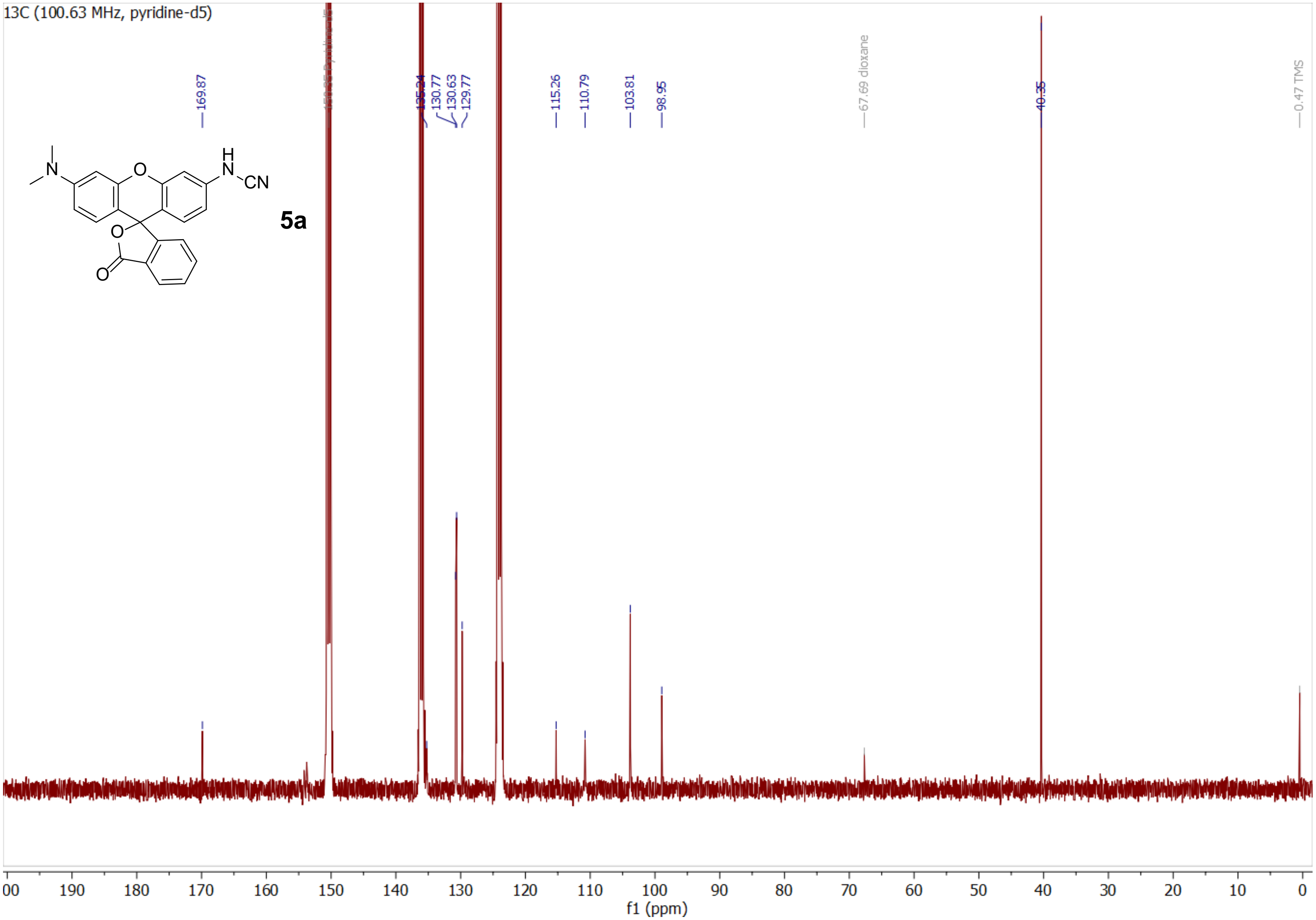

13C (100.63 MHz, pyridine-d5)
169.87
135.24
130.77
130.63
129.77
115.26
110.79
103.81
98.95
67.69 dioxane
40.35
0.47 TMS
N
O
H
N
CN
O
O
f1 (ppm)

5a

5b

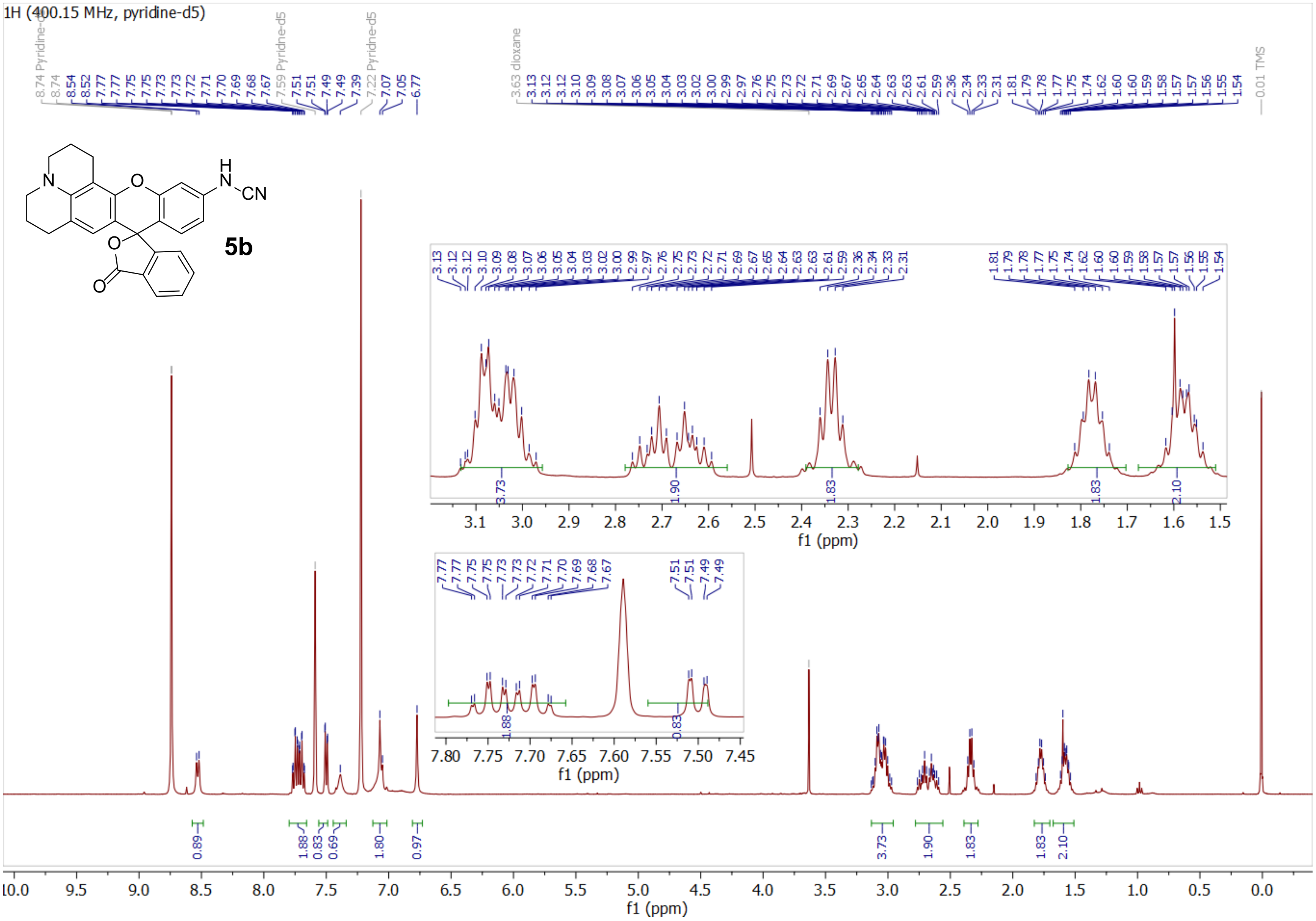

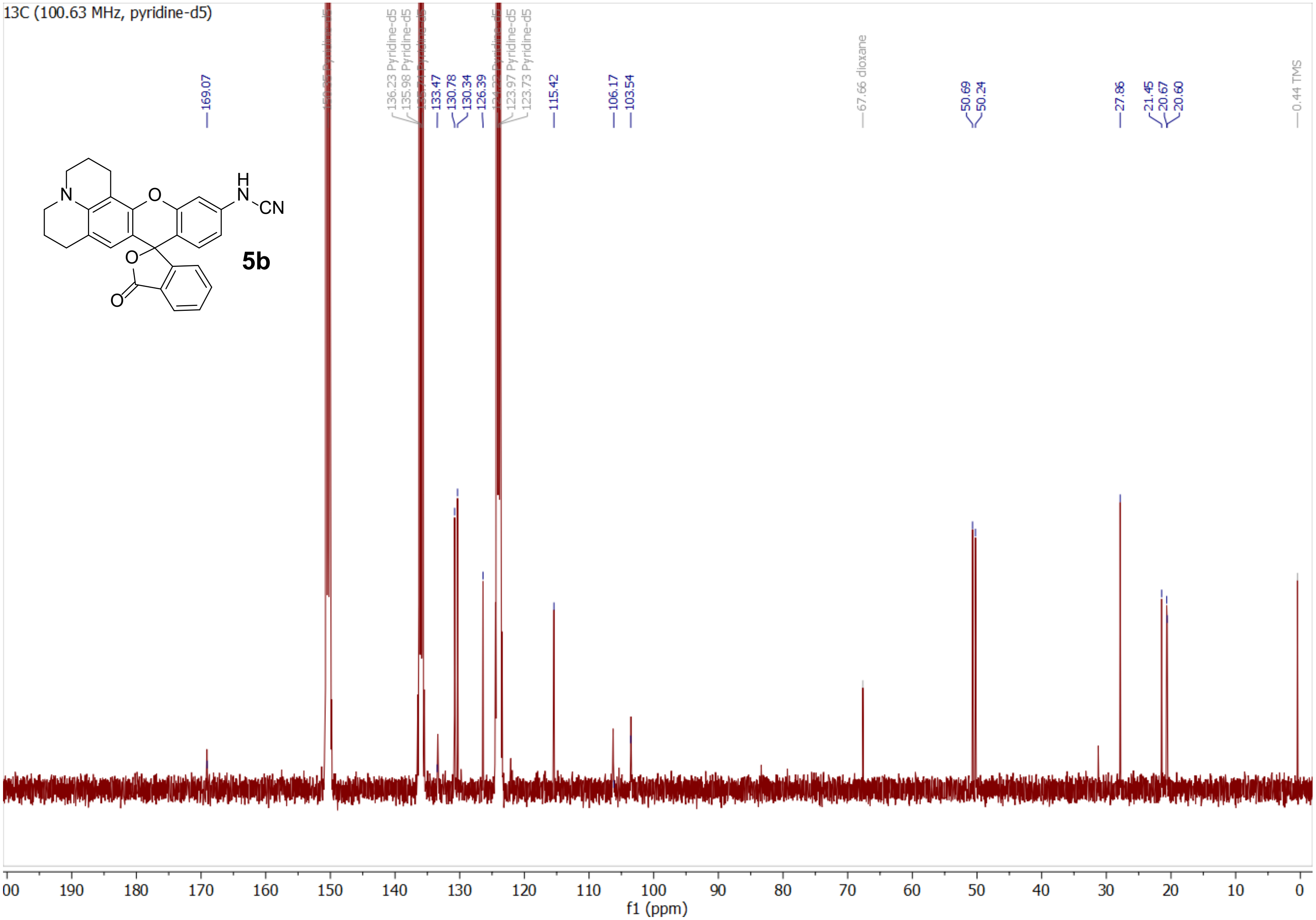
13C (100.63 MHz, pyridine-d5)
169.07
136.23 Pyridine-d5
135.98 Pyridine-d5
133.47
130.78
130.34
126.39
123.97 Pyridine-d5
123.73 Pyridine-d5
115.42
106.17
103.54
67.66 dioxane
50.69
50.24
27.86
21.45
20.67
20.60
0.44 TMS
H
N
CN
N
O
O
O
5b
f1 (ppm)

5c

1H (400.15 MHz, pyridine-d5)

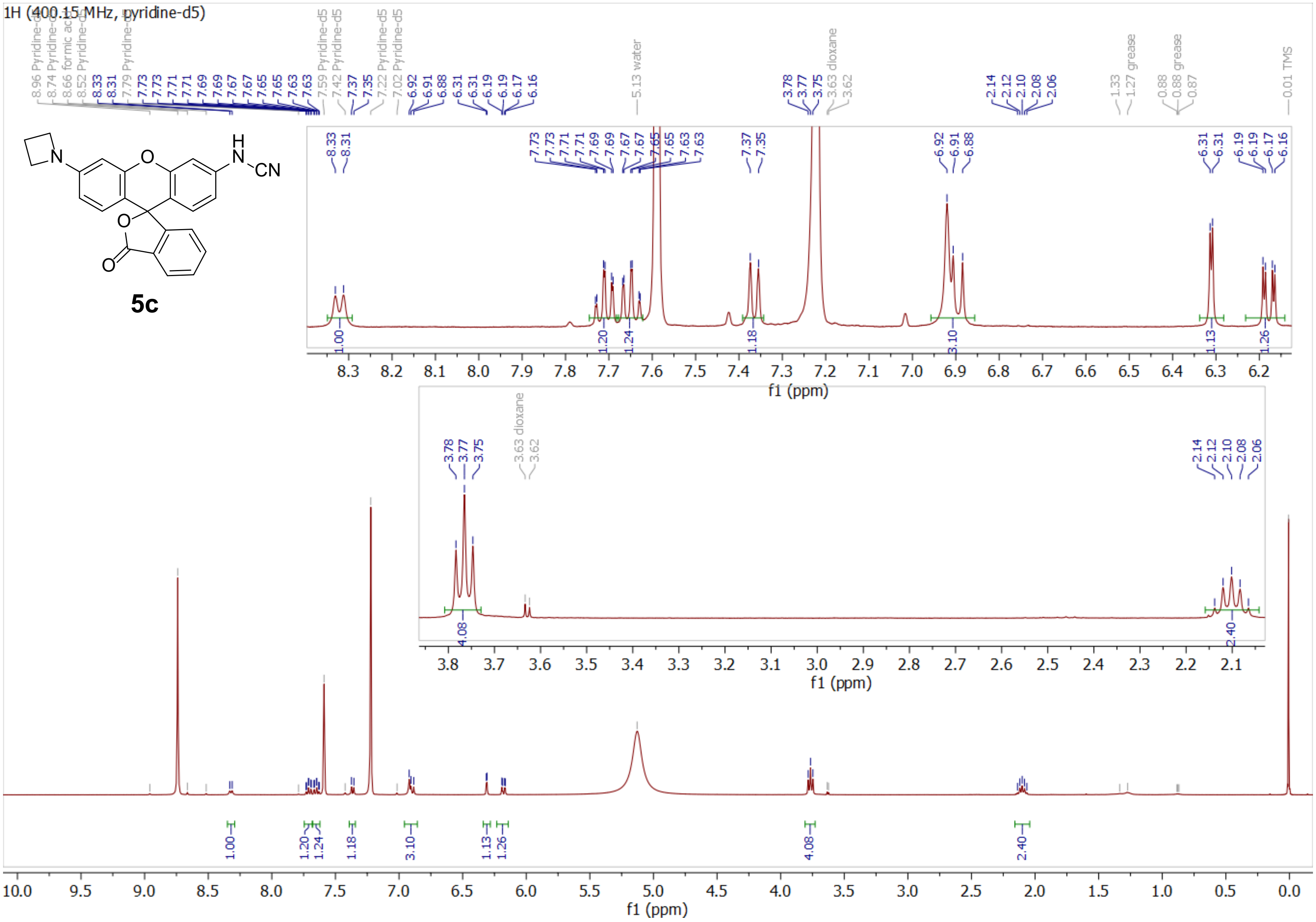

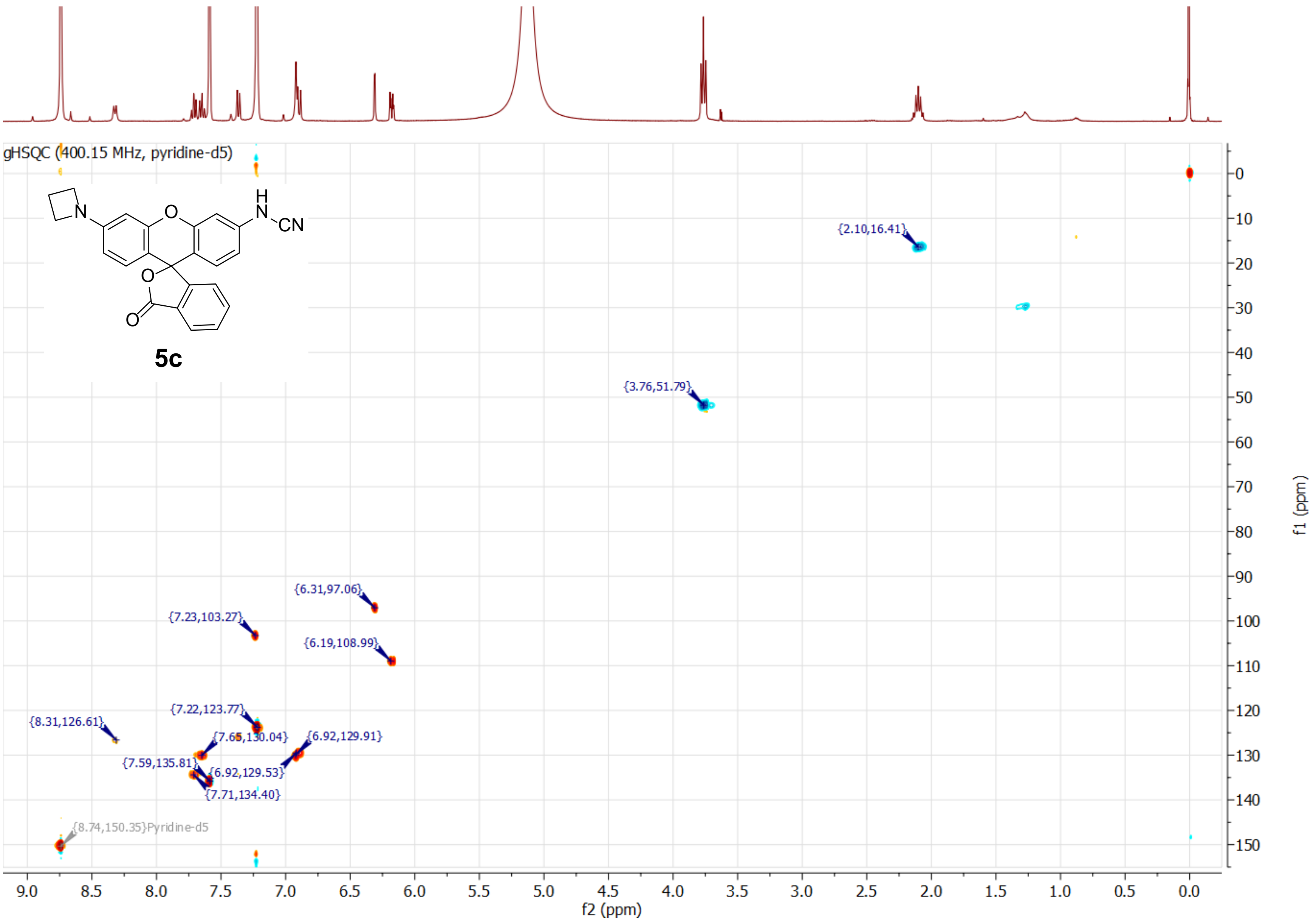
gHSQC (400.15 MHz, pyridine-d5)
5c
{2.10,16.41}
{3.76,51.79}
{6.31,97.06}
{7.23,103.27}
{6.19,108.99}
{7.22,123.77}
{8.31,126.61}
{7.65,130.04}
{6.92,129.91}
{7.59,135.81}
{6.92,129.53}
{7.71,134.40}
{8.74,150.35}Pyridine-d5
f2 (ppm)
f1 (ppm)

6a

1H (400.15 MHz, DMSO)

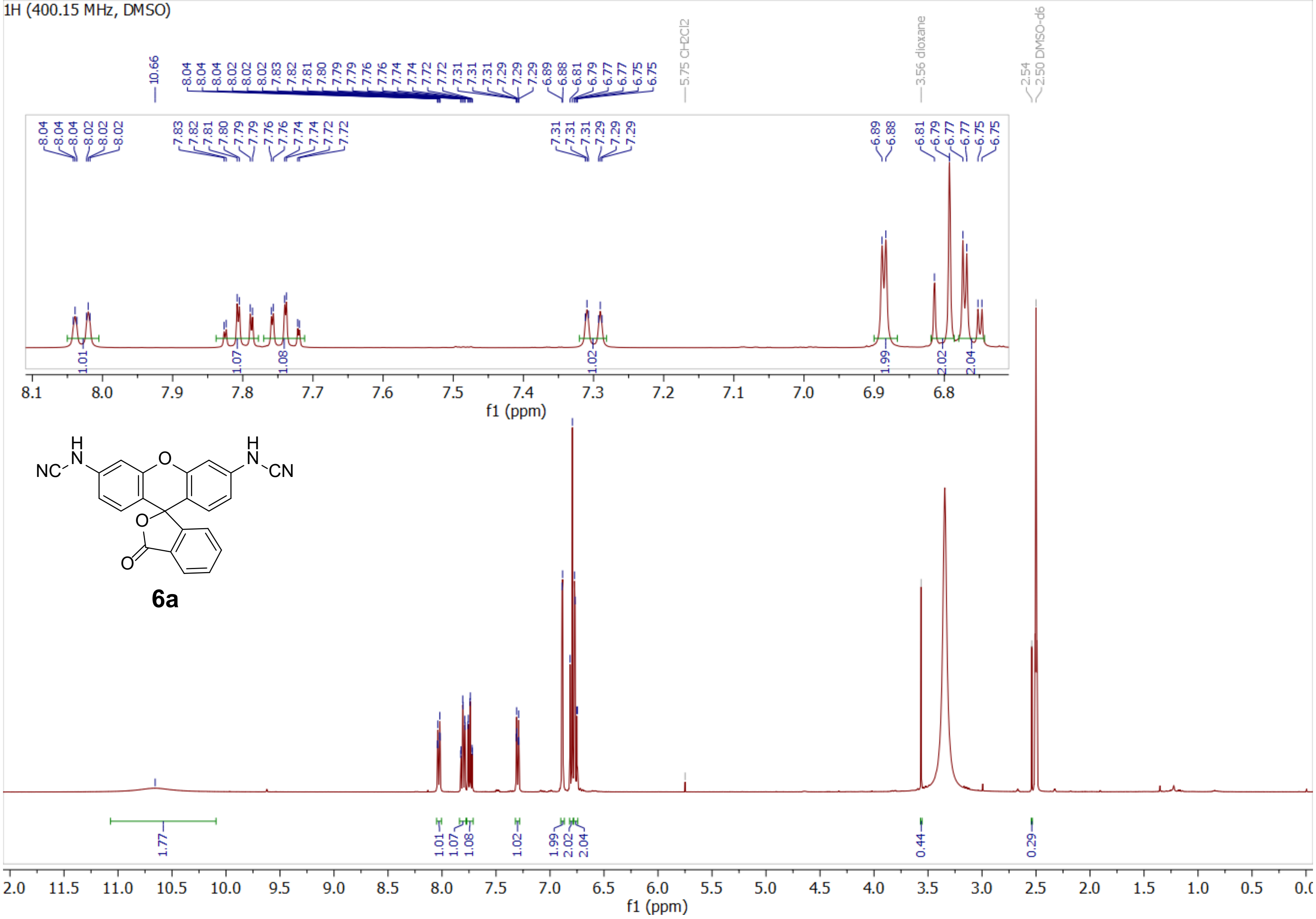

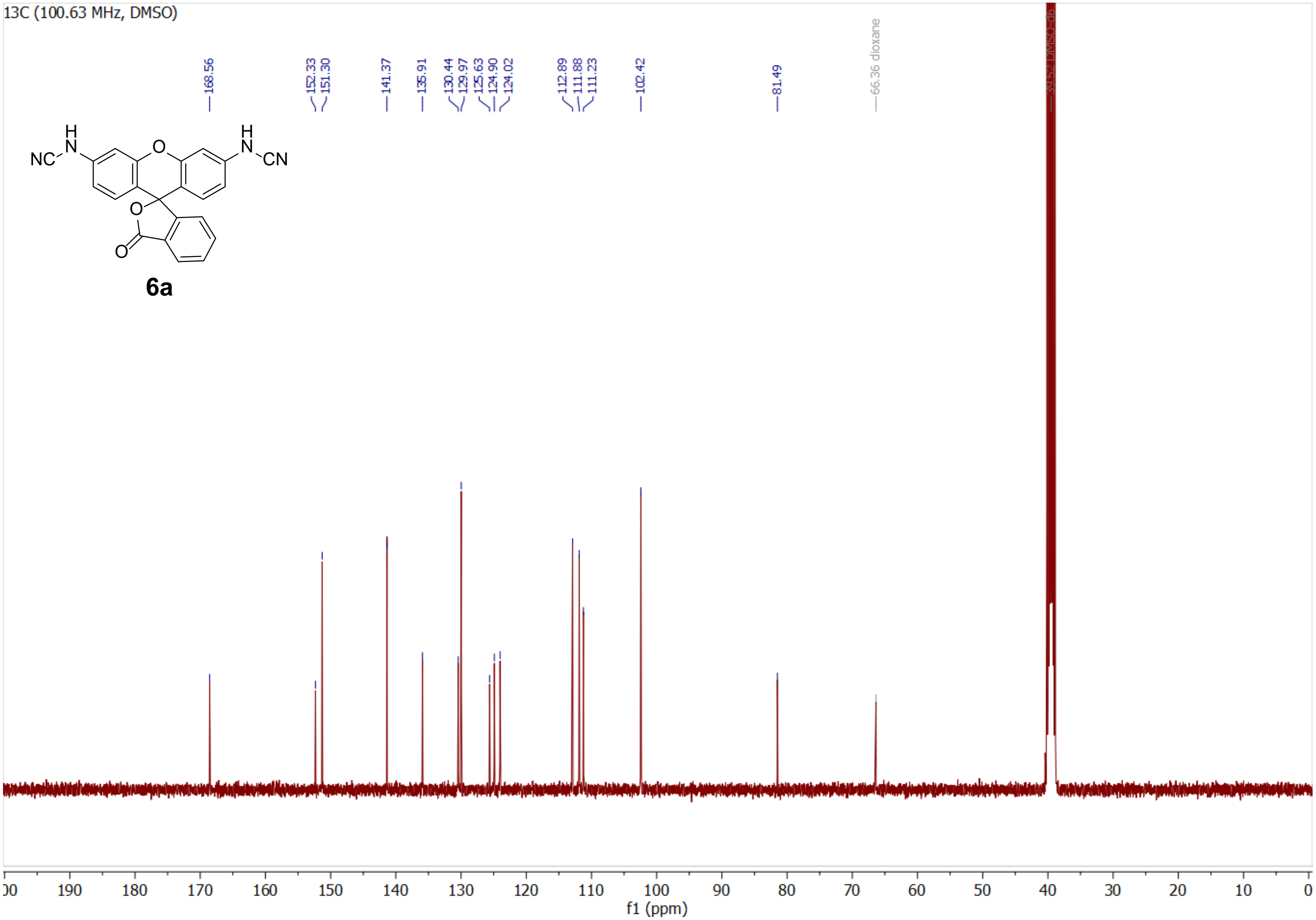
13C (100.63 MHz, DMSO)
168.56
152.33
151.30
141.37
135.91
130.44
129.97
125.63
124.90
124.02
112.89
111.88
111.23
102.42
81.49
66.36 dioxane
39.52 DMSO-d6
NC
H
N
O
N
H
CN
O
O
6a
f1 (ppm)

6b

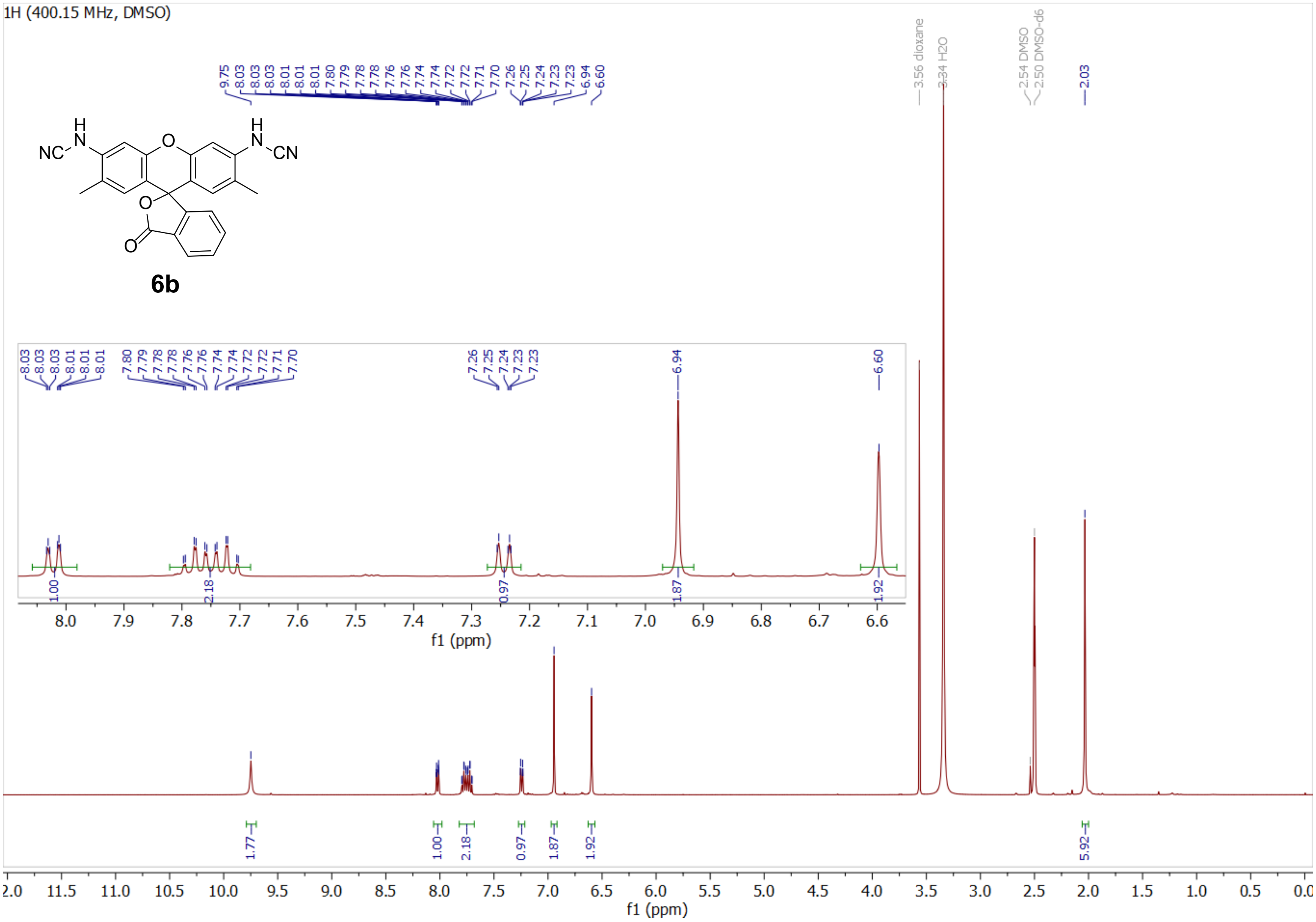
1H (400.15 MHz, DMSO)
6b
f1 (ppm)
3.56 dioxane
3.34 H2O
2.54 DMSO
2.50 DMSO-d6

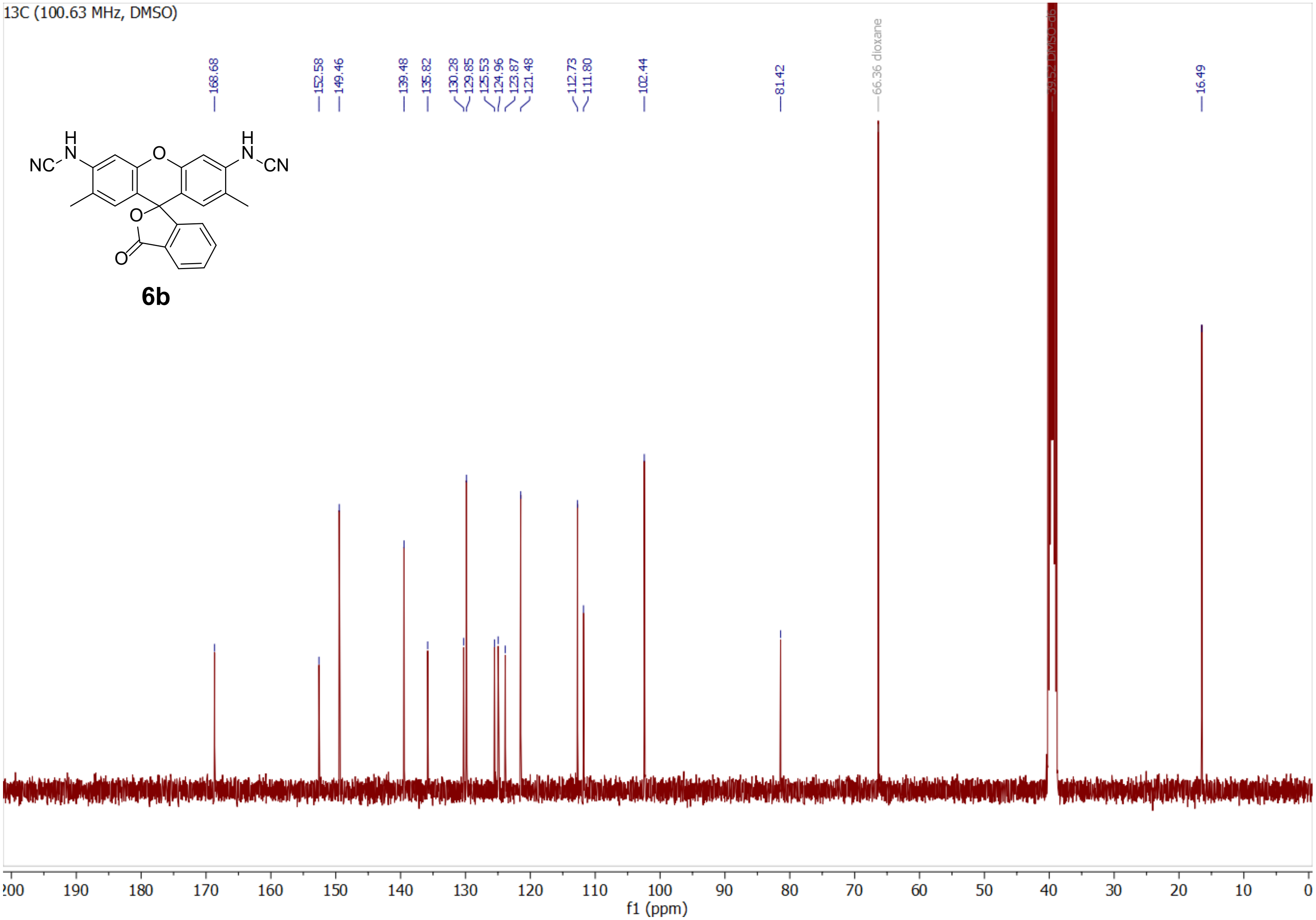

13C (100.63 MHz, DMSO)
168.68
152.58
149.46
139.48
135.82
130.28
129.85
125.53
124.96
123.87
121.48
112.73
111.80
102.44
81.42
66.36 dioxane
39.52 DMSO-d6
16.49
NC
N
H
O
N
H
CN
O
O
6b
200
190
180
170
160
150
140
130
120
110
100
90
80
70
60
50
40
30
20
10
0
f1 (ppm)

6c

1H (400.15 MHz, DMSO)

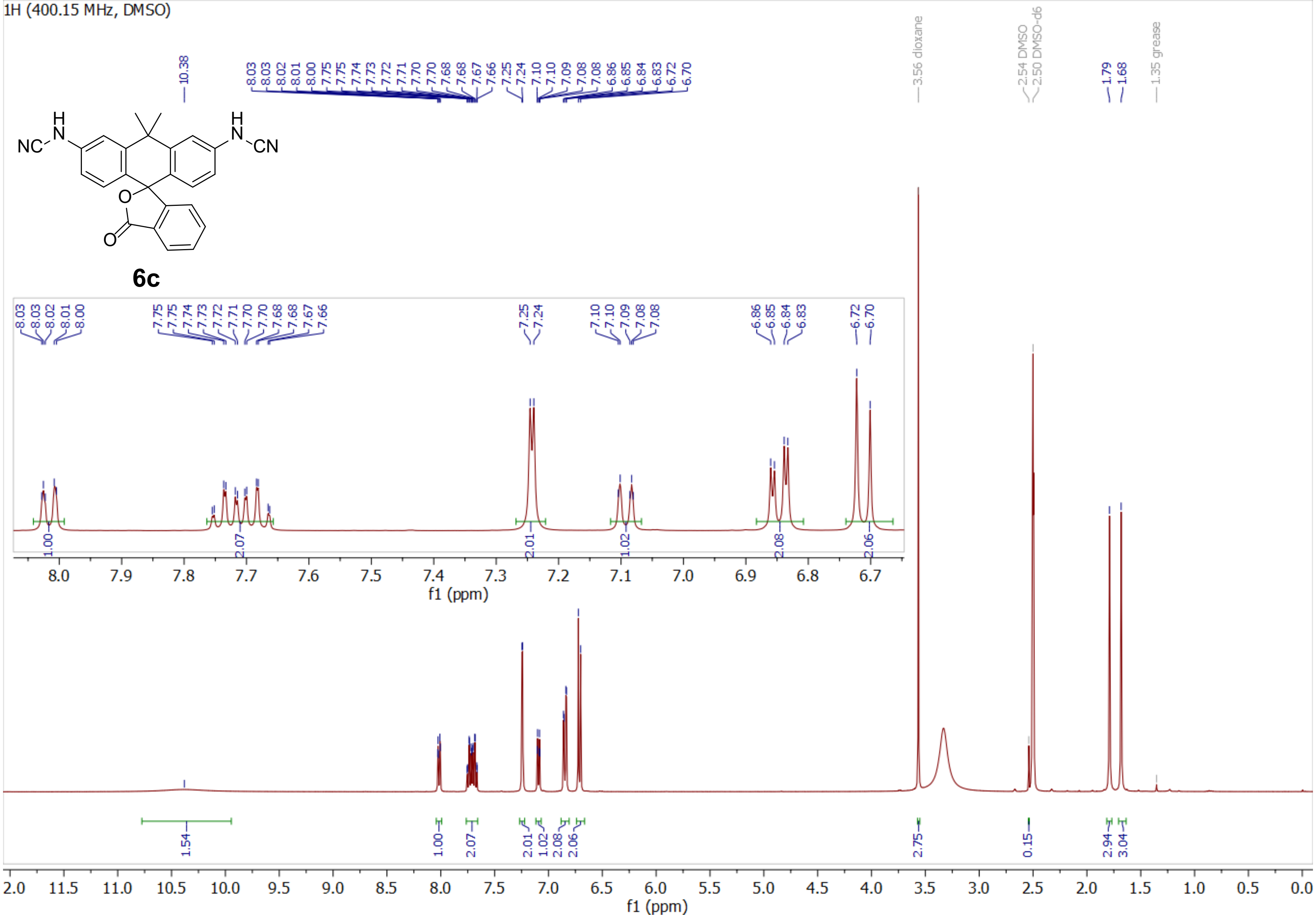

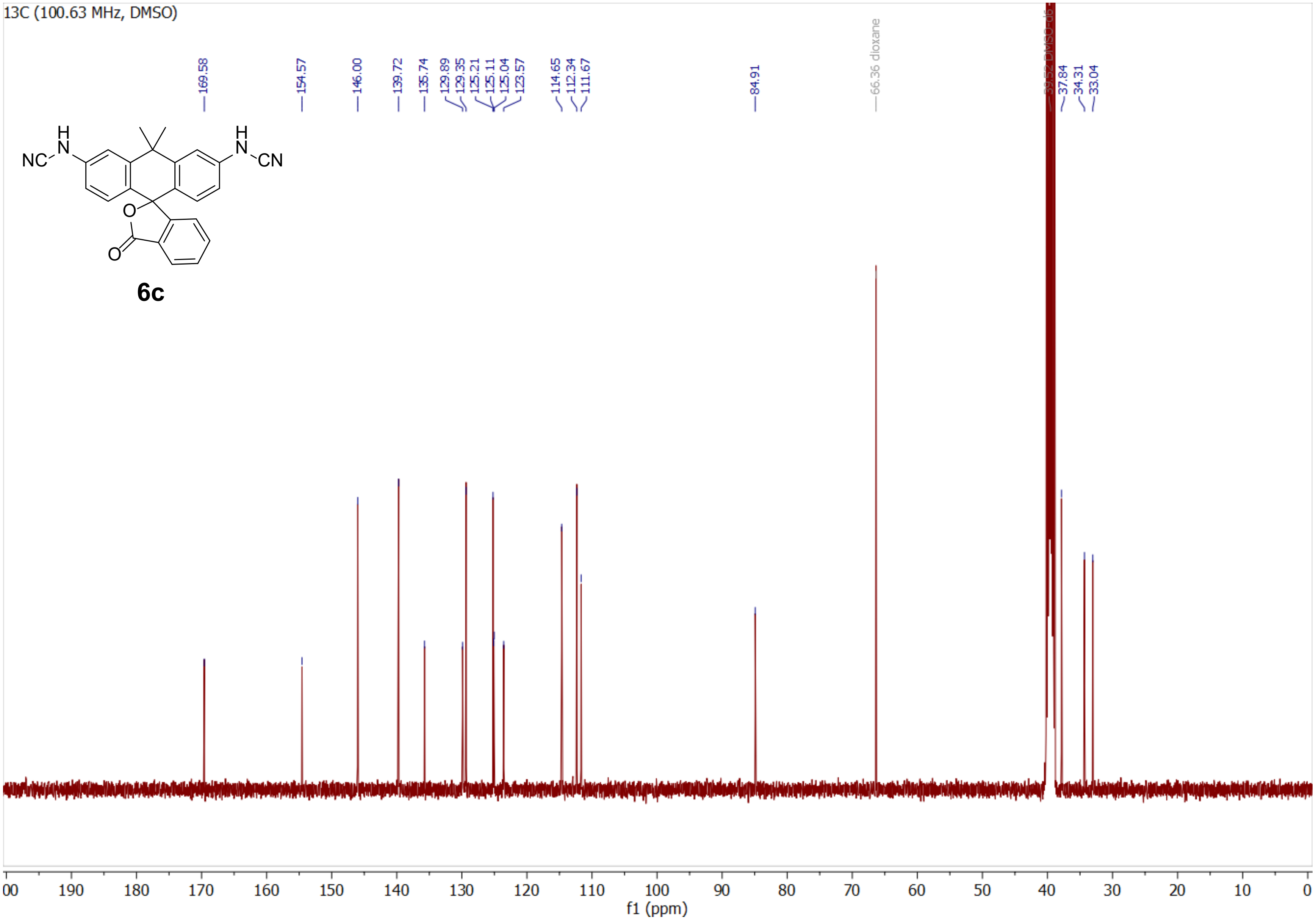
13C (100.63 MHz, DMSO)
169.58
154.57
146.00
139.72
135.74
129.89
129.35
125.21
125.11
125.04
123.57
114.65
112.34
111.67
84.91
66.36 dioxane
37.84
34.31
33.04
NC
H
N
H
N
CN
O
O
6c
00
190
180
170
160
150
140
130
120
110
100
90
80
70
60
50
40
30
20
10
0
f1 (ppm)

7

1H (400.15 MHz, DMSO)

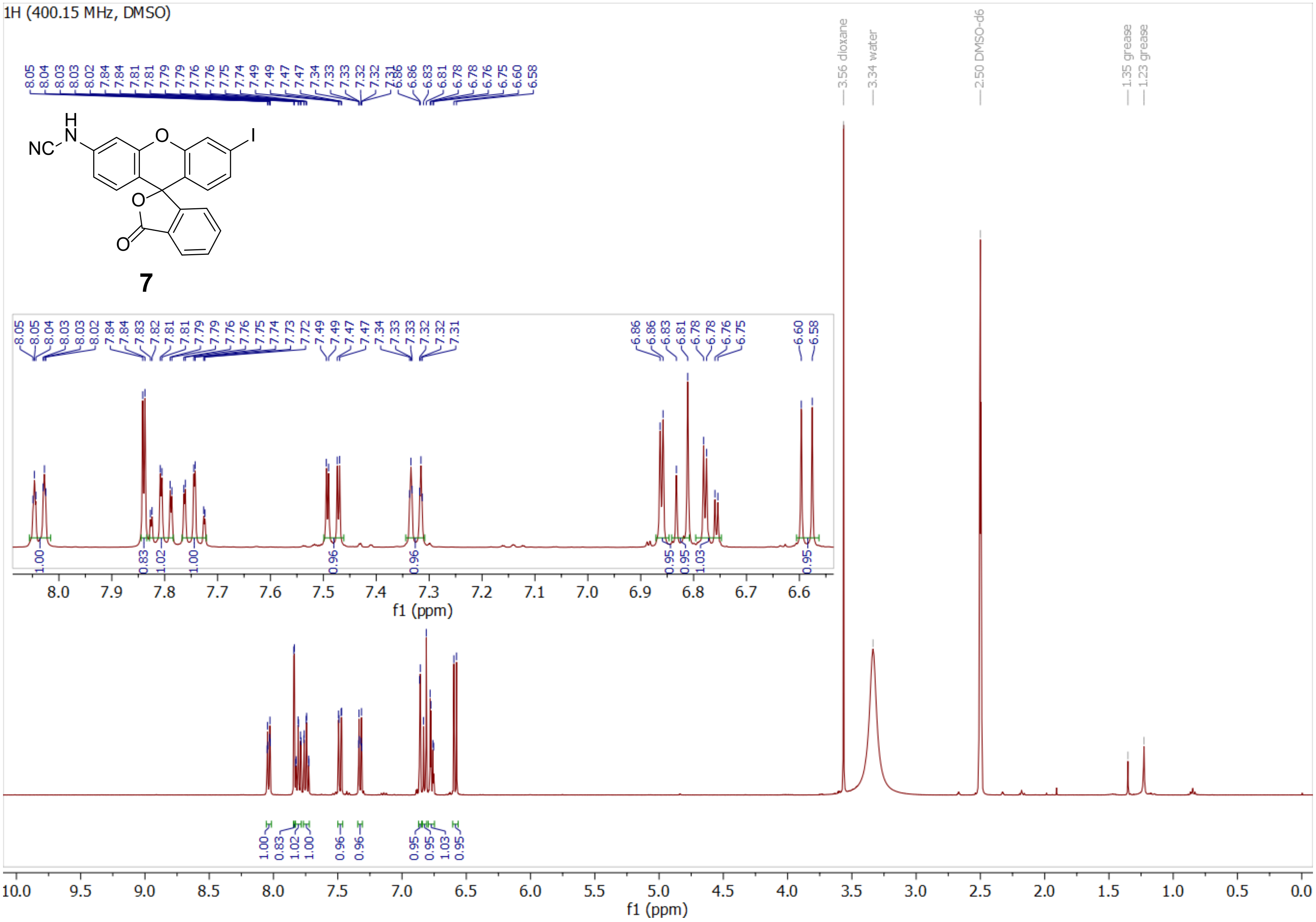

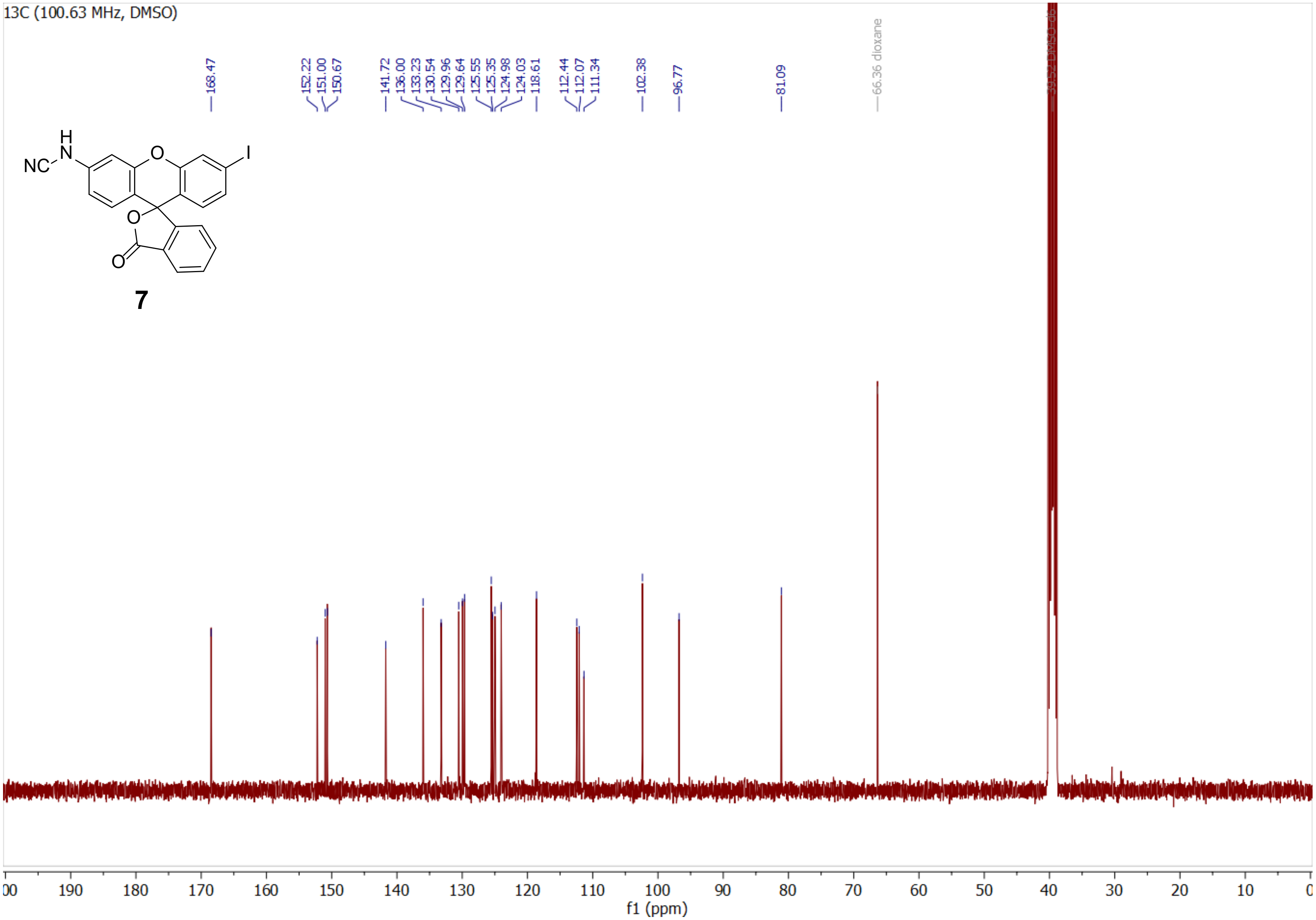
13C (100.63 MHz, DMSO)
168.47
152.22
151.00
150.67
141.72
136.00
133.23
130.54
129.96
129.64
125.55
125.35
124.98
124.03
118.61
112.44
112.07
111.34
102.38
96.77
81.09
66.36 dioxane
39.52 DMSO-d6
NC
H
N
O
I
O
O
7
f1 (ppm)

8a

1H (400.15 MHz, DMSO + 1% TFA-d)

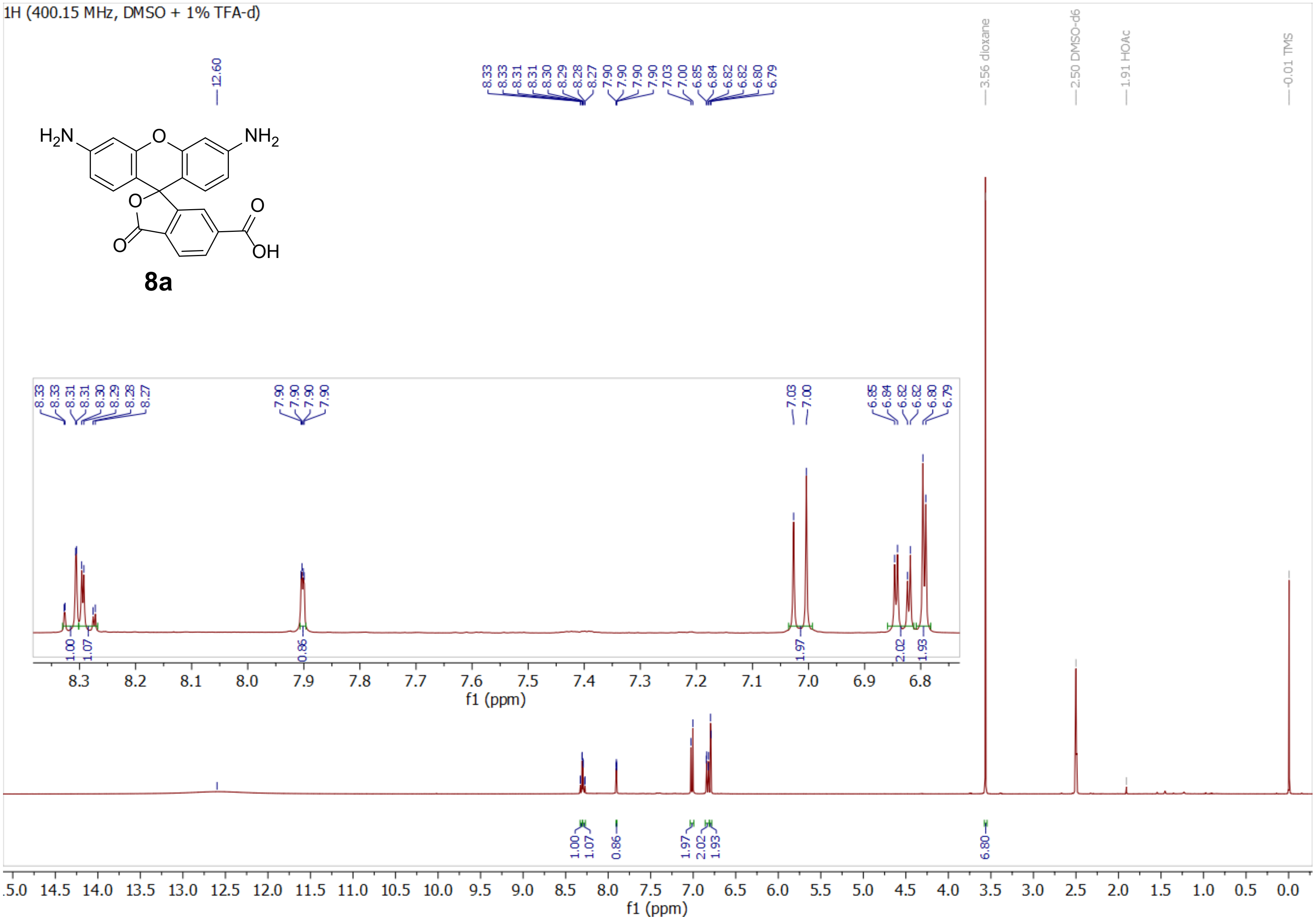

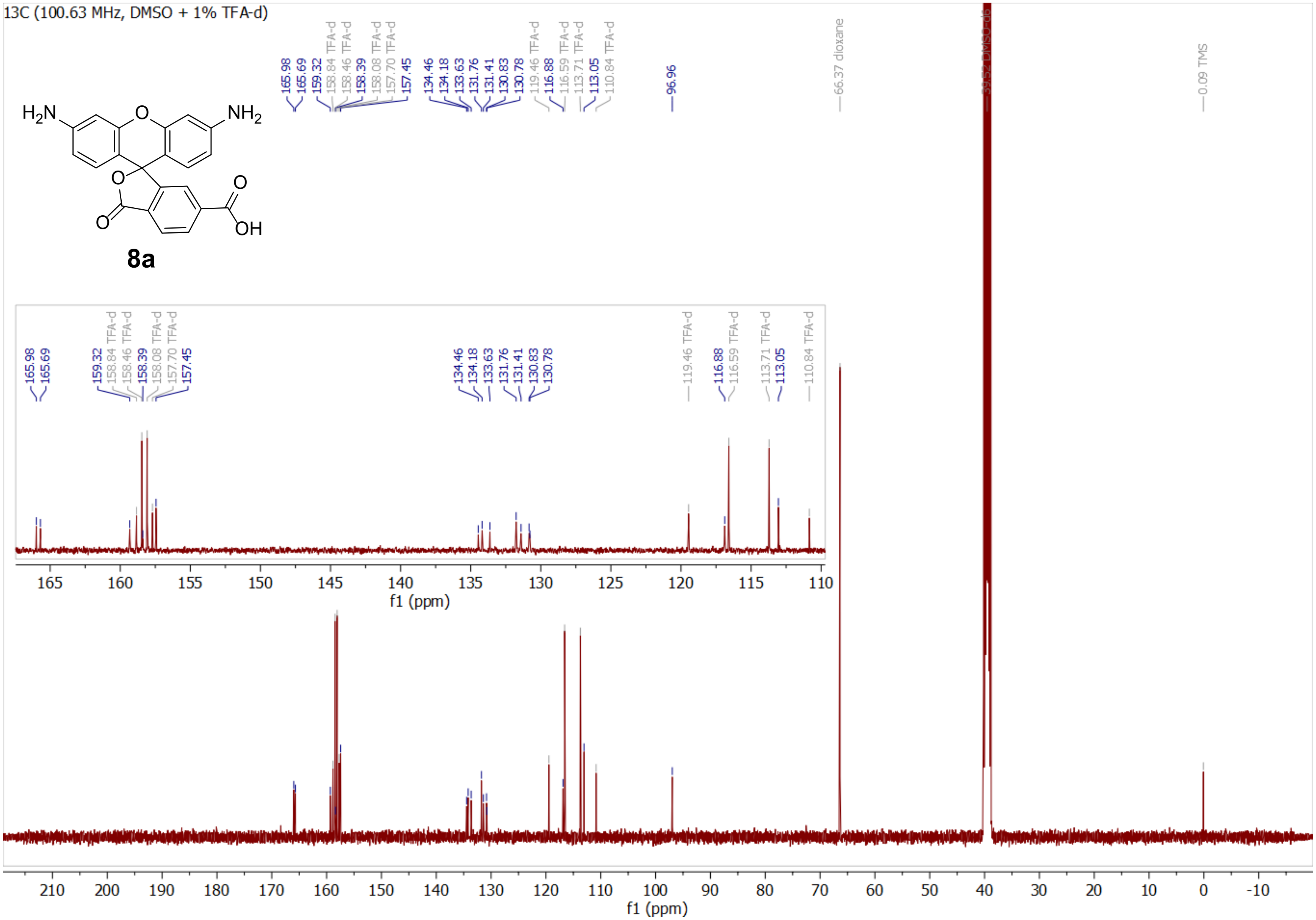
13C (100.63 MHz, DMSO + 1% TFA-d)
H2N
O
NH2
O
O
O
OH
8a
165.98
165.69
159.32
158.84 TFA-d
158.46 TFA-d
158.39
158.08 TFA-d
157.70 TFA-d
157.45
134.46
134.18
133.63
131.76
131.41
130.83
130.78
119.46 TFA-d
116.88
116.59 TFA-d
113.71 TFA-d
113.05
110.84 TFA-d
96.96
66.37 dioxane
0.09 TMS
f1 (ppm)

8b

1H (400.15 MHz, MeOD)

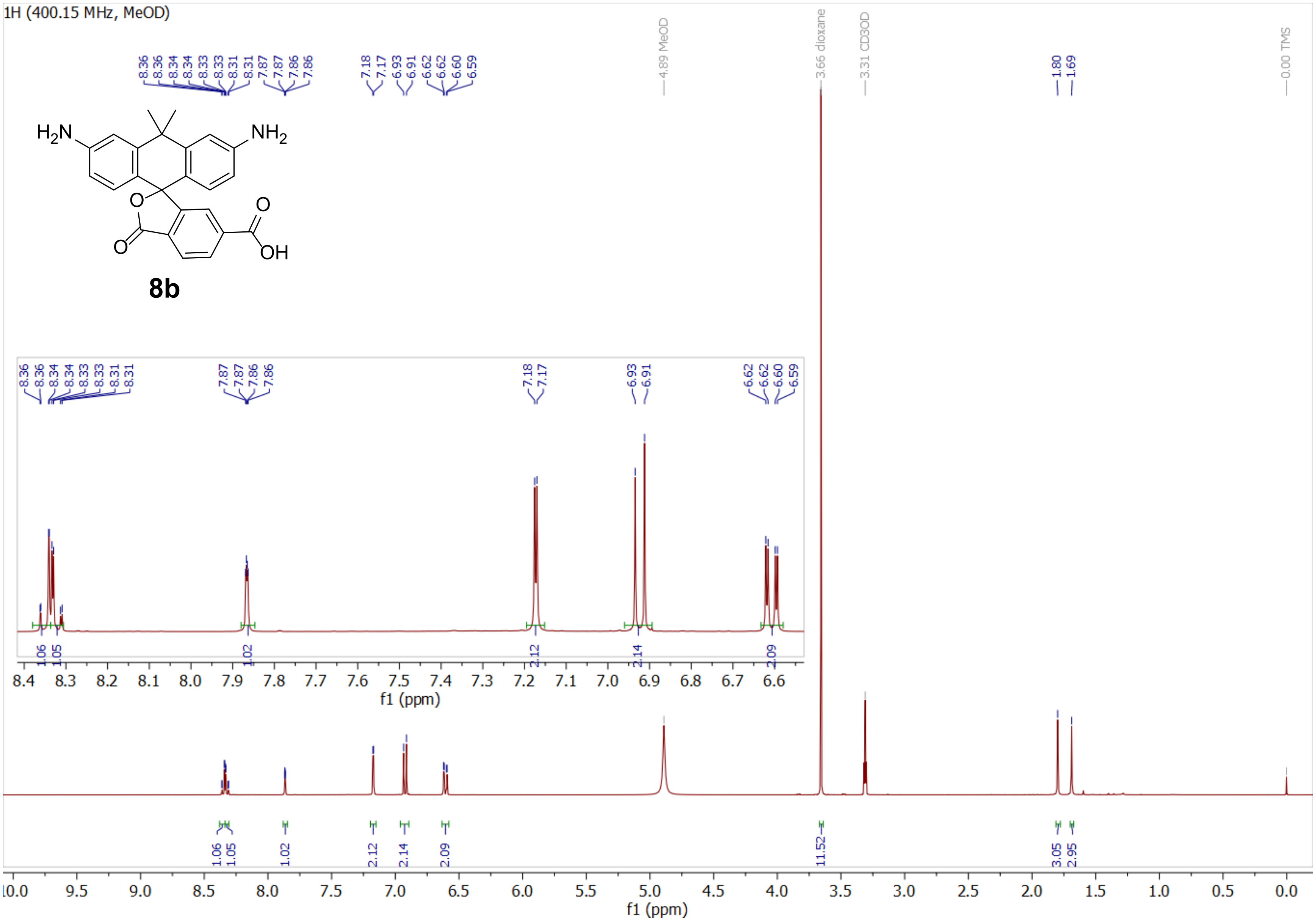

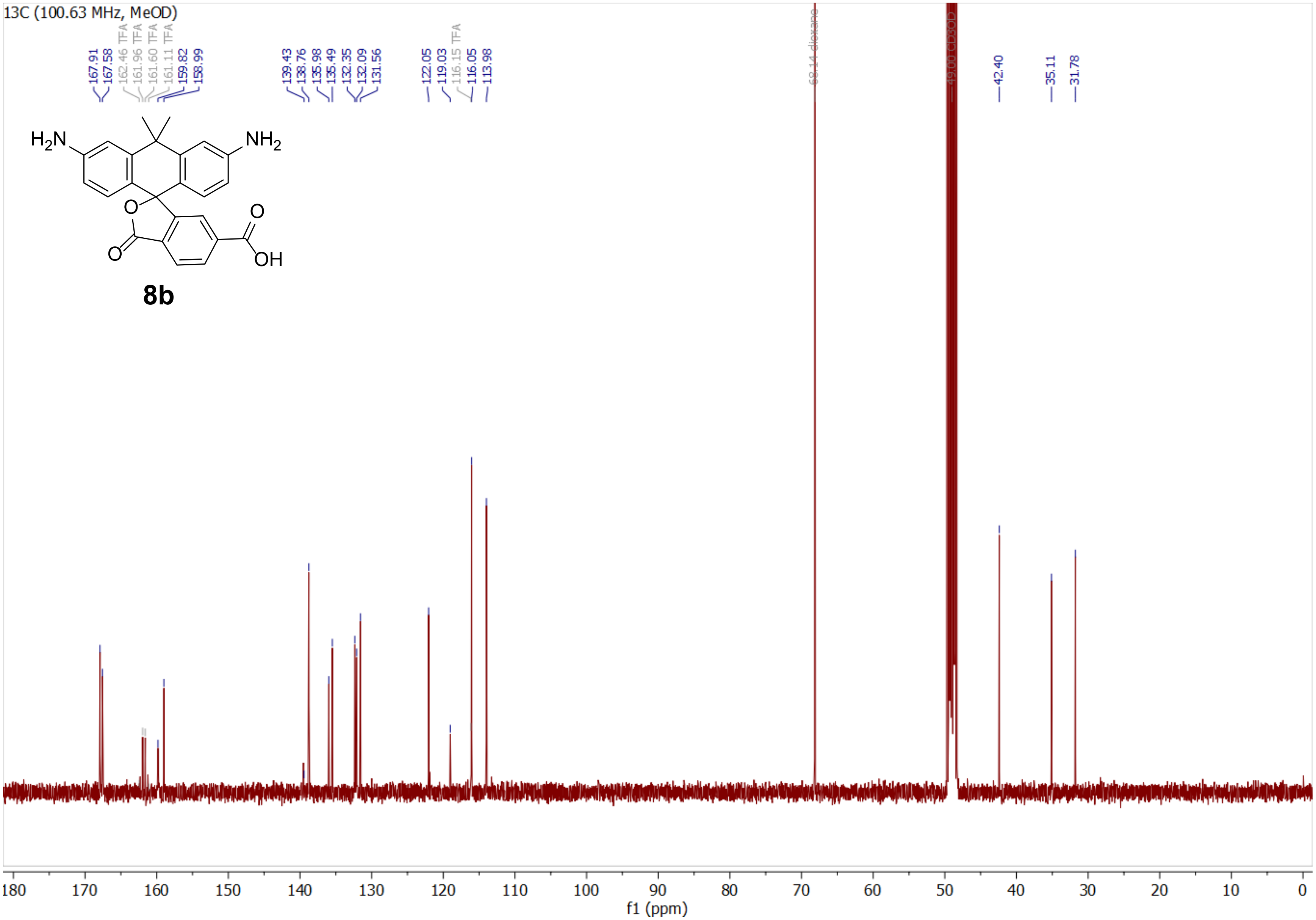
13C (100.63 MHz, MeOD)
167.91
167.58
162.46 TFA
161.96 TFA
161.60 TFA
161.11 TFA
159.82
158.99
139.43
138.76
135.98
135.49
132.35
132.09
131.56
122.05
119.03
116.15 TFA
116.05
113.98
68.14 dioxane
49.00 CD3OD
42.40
35.11
31.78
H2N
NH2
O
O
O
OH
8b
f1 (ppm)

9a

1H (400.15 MHz, DMSO + 1% TFA-d)

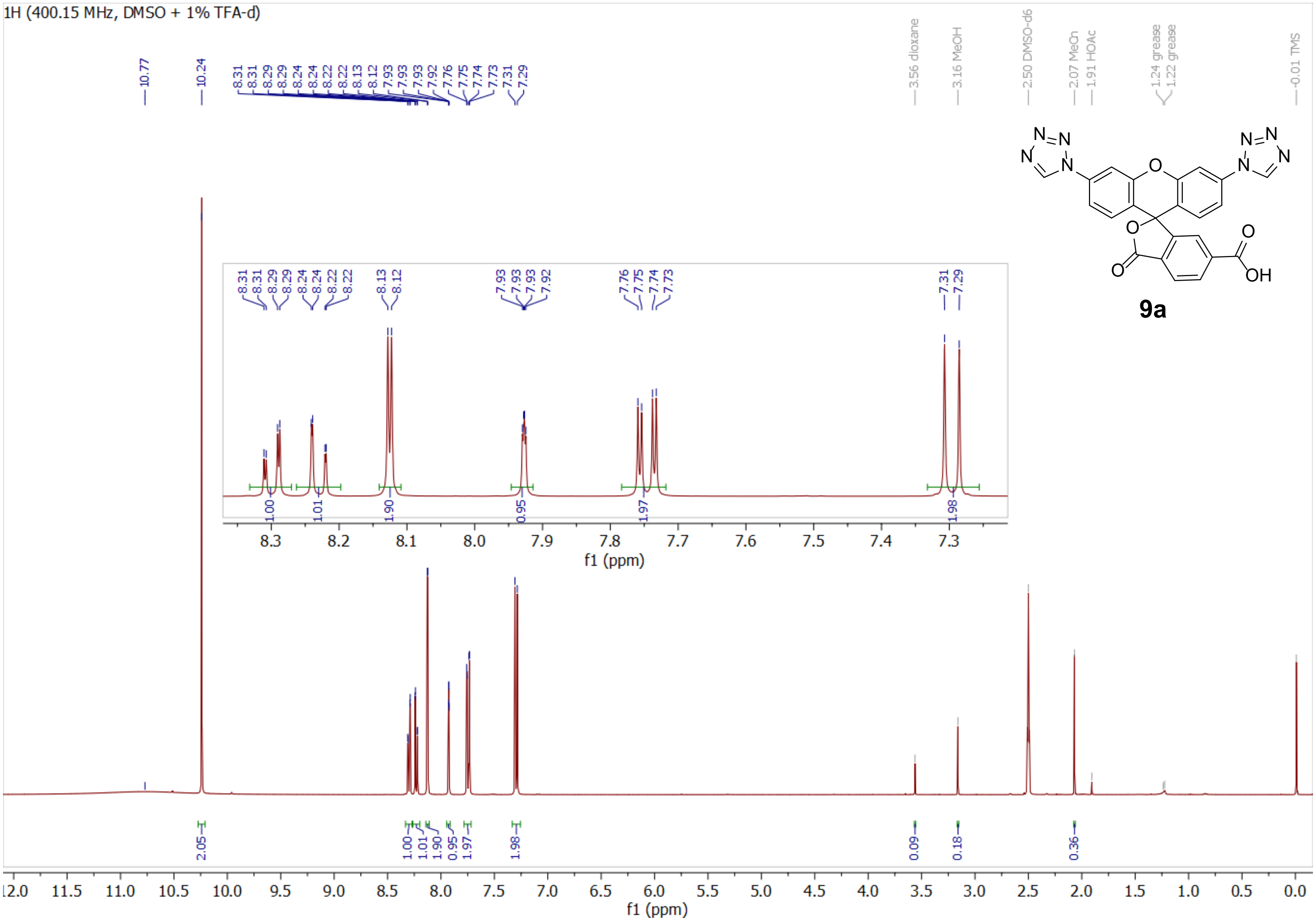

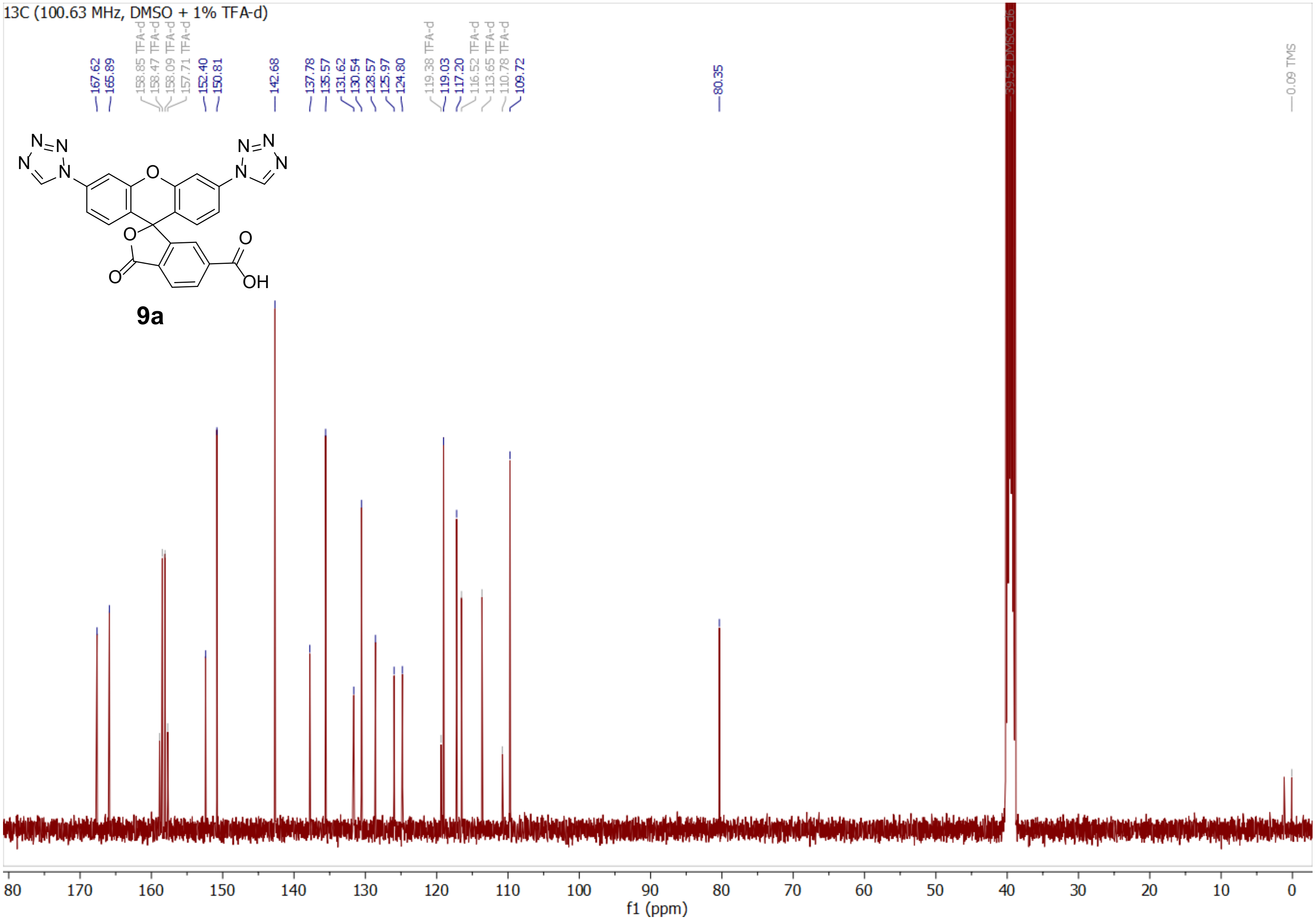
13C (100.63 MHz, DMSO + 1% TFA-d)
167.62
165.89
158.85 TFA-d
158.47 TFA-d
158.09 TFA-d
157.71 TFA-d
152.40
150.81
142.68
137.78
135.57
131.62
130.54
128.57
125.97
124.80
119.38 TFA-d
119.03
117.20
116.52 TFA-d
113.65 TFA-d
110.78 TFA-d
109.72
80.35
39.52 DMSO-d6
0.09 TMS
9a
f1 (ppm)

9b

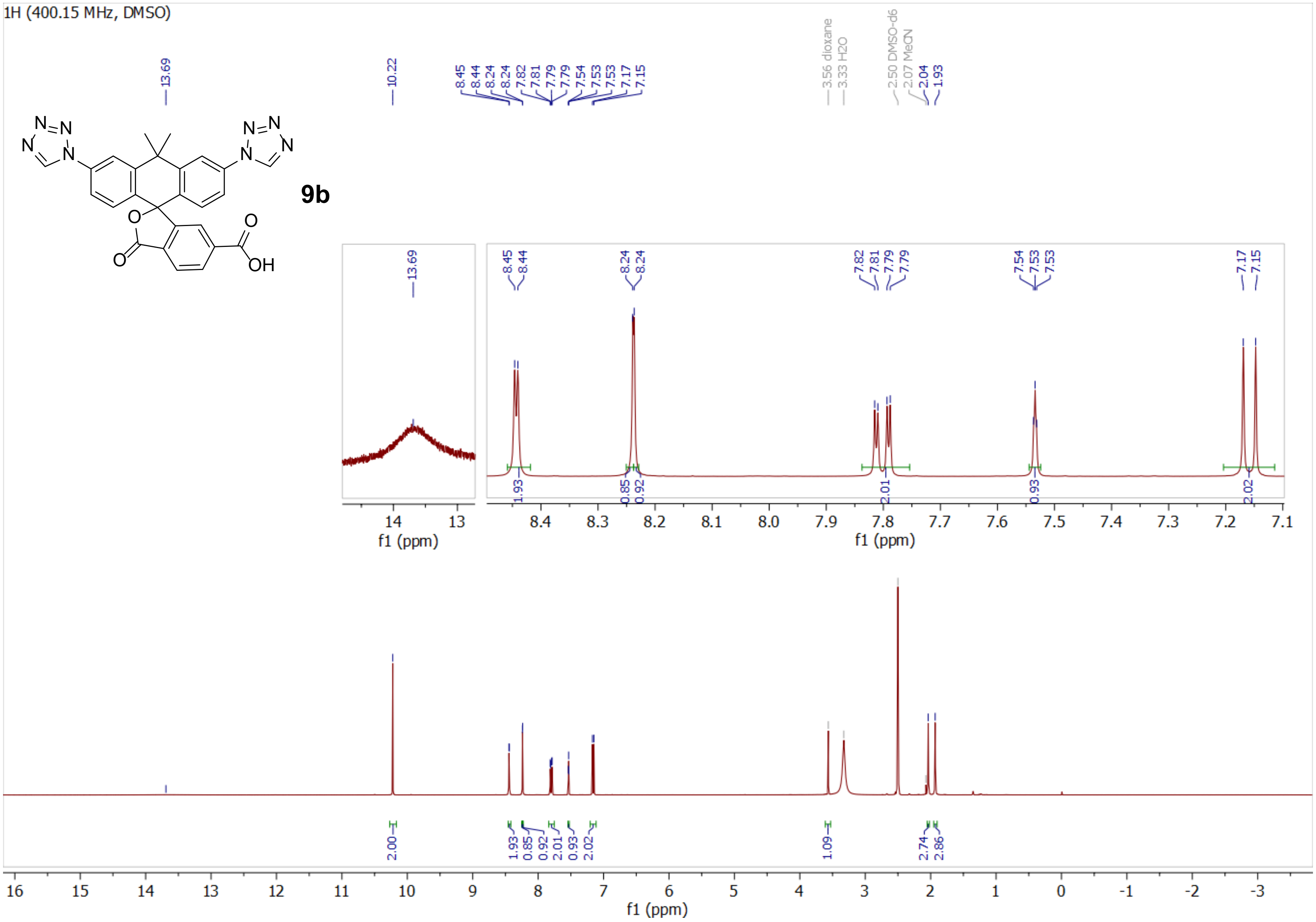

1H (400.15 MHz, DMSO)
13.69
10.22
8.45
8.44
8.24
8.24
7.82
7.81
7.79
7.79
7.54
7.53
7.53
7.17
7.15
3.56 dioxane
3.33 H2O
2.50 DMSO-d6
2.07 MeCN
2.04
1.93
9b
1.93
0.85
0.92
2.01
0.93
2.02
2.00
1.09
2.74
2.86
f1 (ppm)

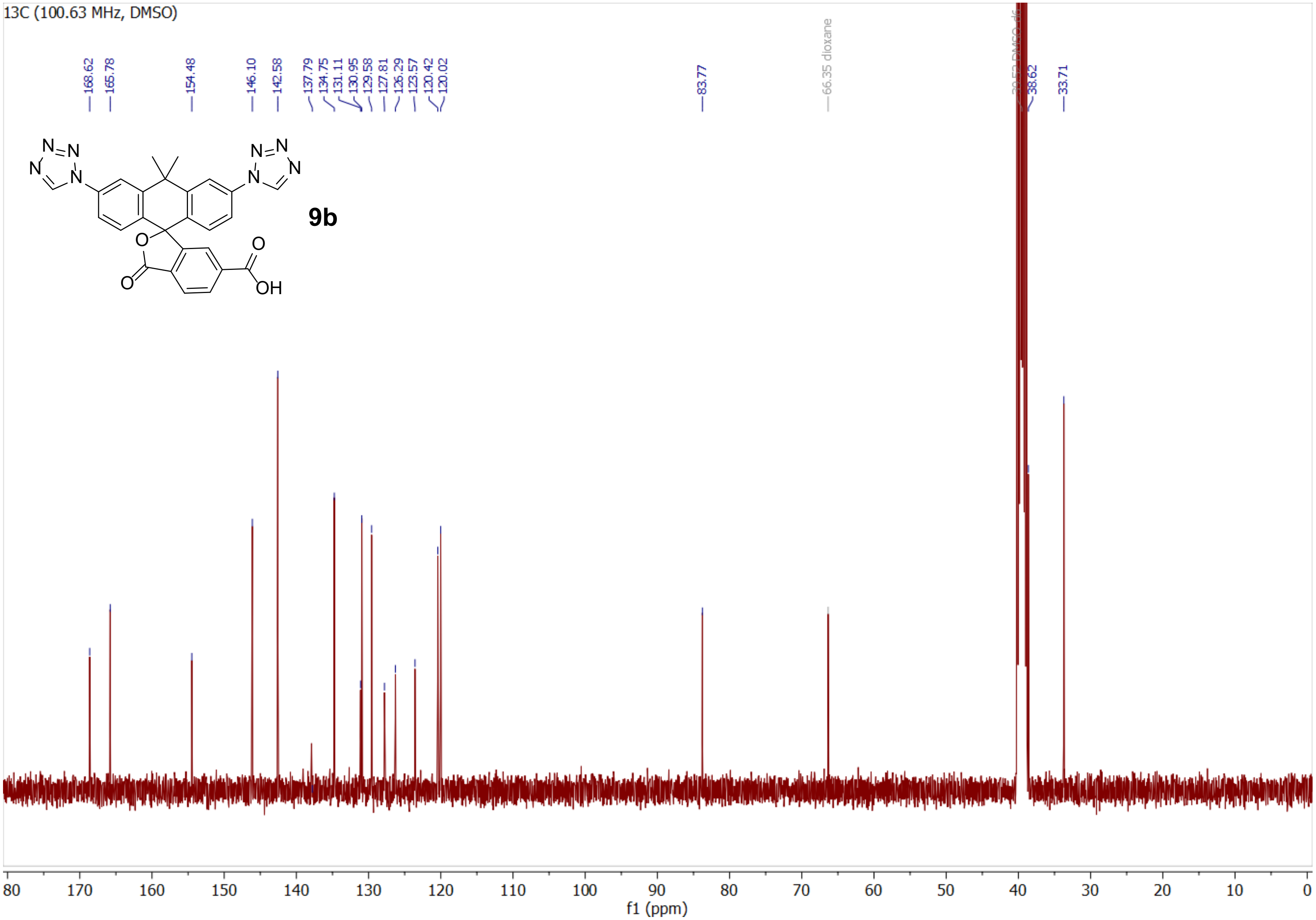
13C (100.63 MHz, DMSO)
168.62
165.78
154.48
146.10
142.58
137.79
134.75
131.11
130.95
129.58
127.81
126.29
123.57
120.42
120.02
83.77
66.35 dioxane
38.62
33.71
9b
O
OH
80
170
160
150
140
130
120
110
100
90
80
70
60
50
40
30
20
10
0
f1 (ppm)

9c

1H (400.15 MHz, DMSO)

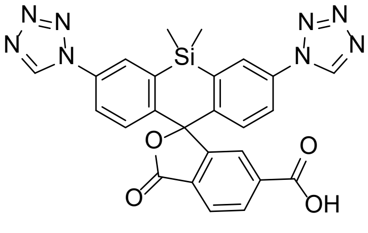

9c

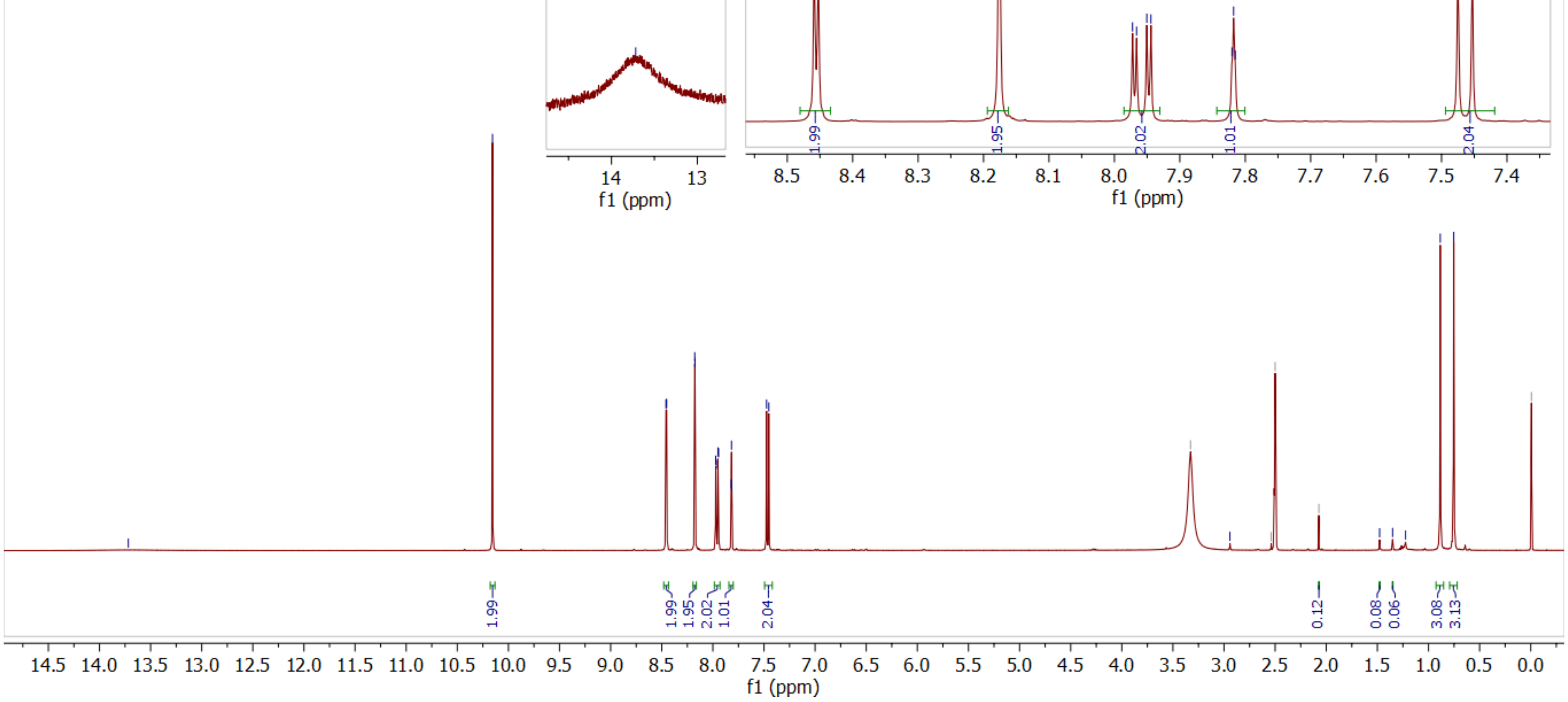

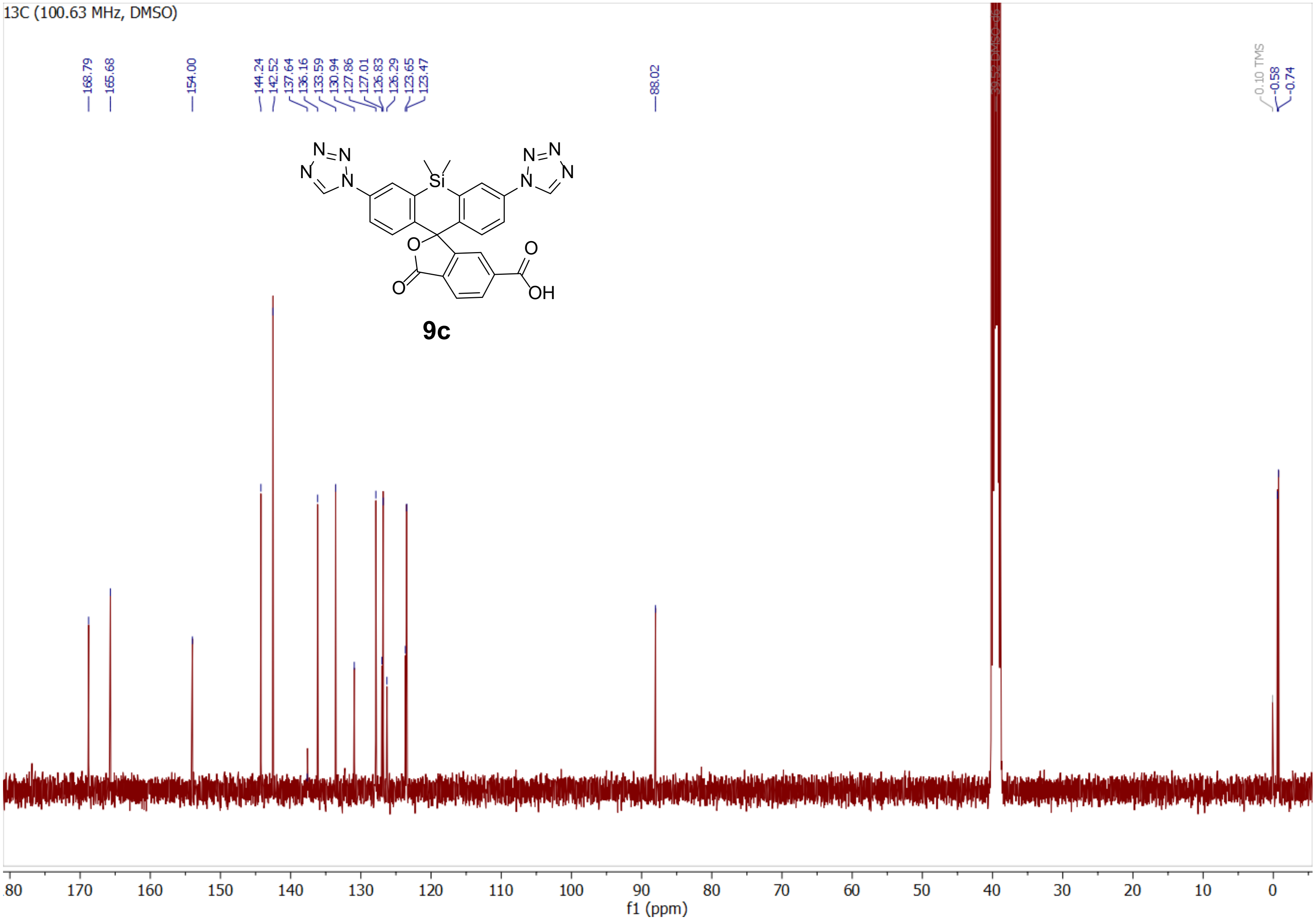
13C (100.63 MHz, DMSO)
168.79
165.68
154.00
144.24
142.52
137.64
136.16
133.59
130.94
127.86
127.01
126.83
126.29
123.65
123.47
88.02
39.52 DMSO-d6
0.10 TMS
-0.58
-0.74
Si
O
OH
9c
f1 (ppm)

9b-Halo

1H (400.15 MHz, DMSO)

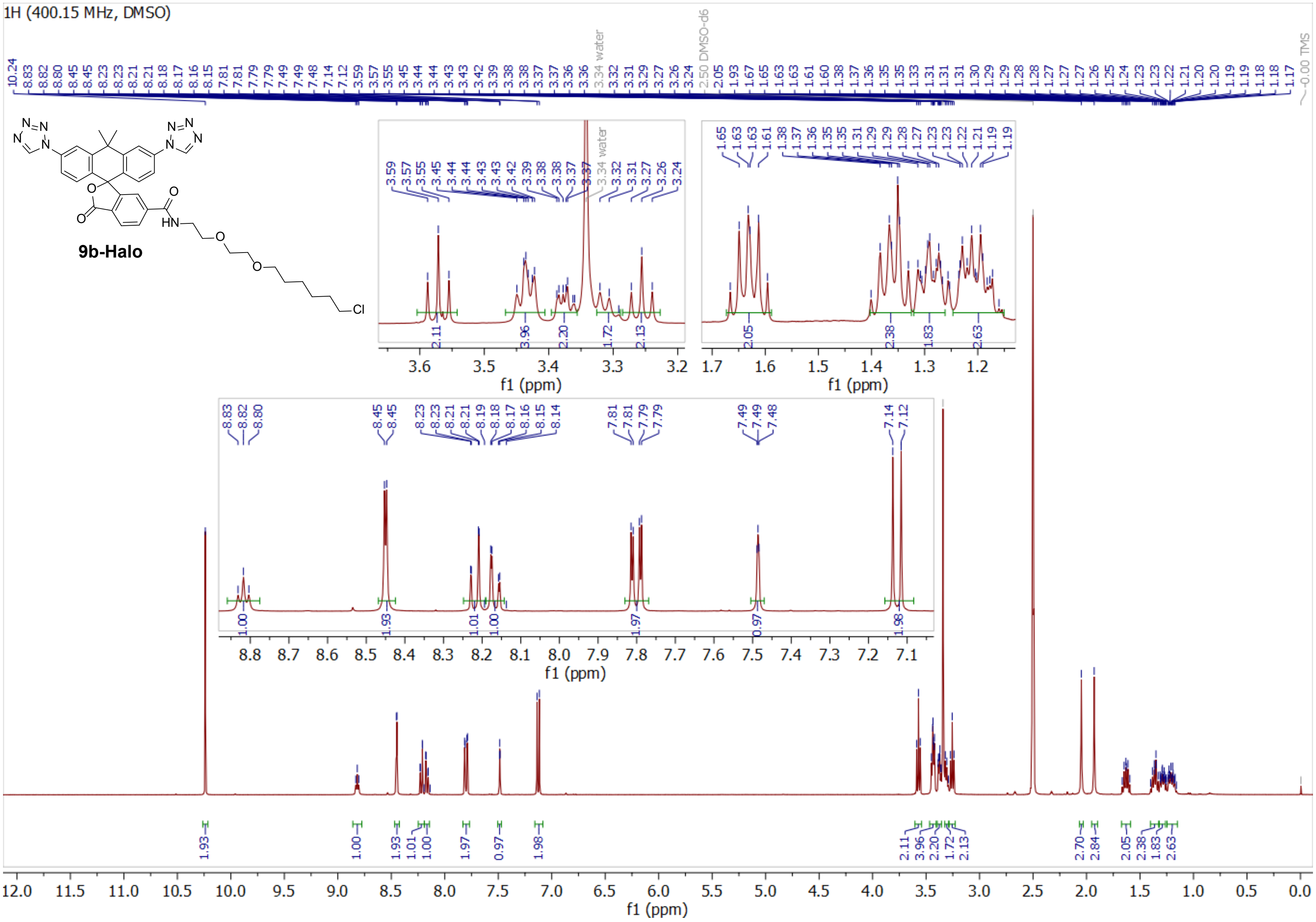

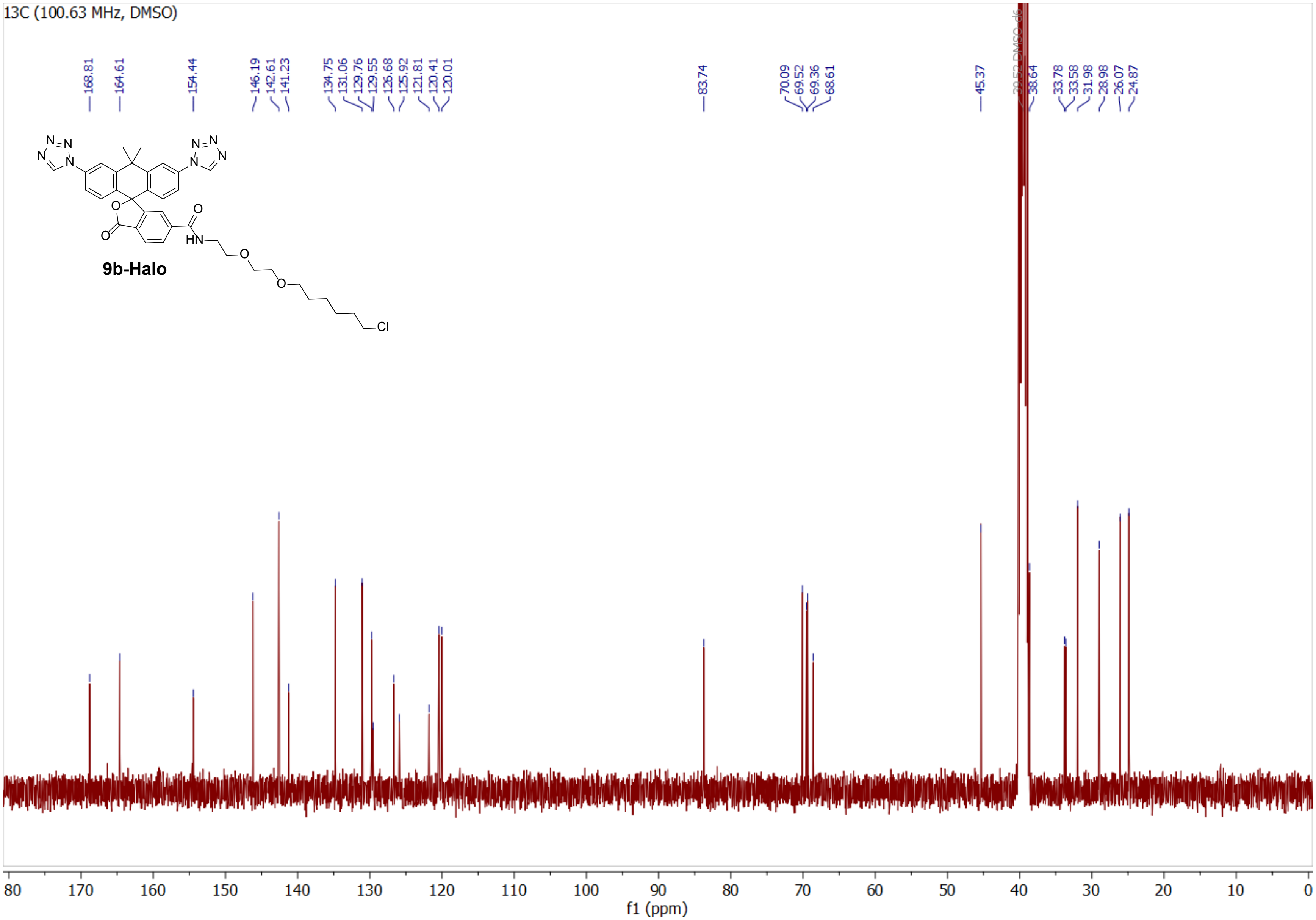
13C (100.63 MHz, DMSO)
168.81
164.61
154.44
146.19
142.61
141.23
134.75
131.06
129.76
129.55
126.68
125.92
121.81
120.41
120.01
83.74
70.09
69.52
69.36
68.61
45.37
39.52 DMSO-d6
38.64
33.78
33.58
31.98
28.98
26.07
24.87
9b-Halo
f1 (ppm)

9c-Halo

1H (400.15 MHz, CDCl3)

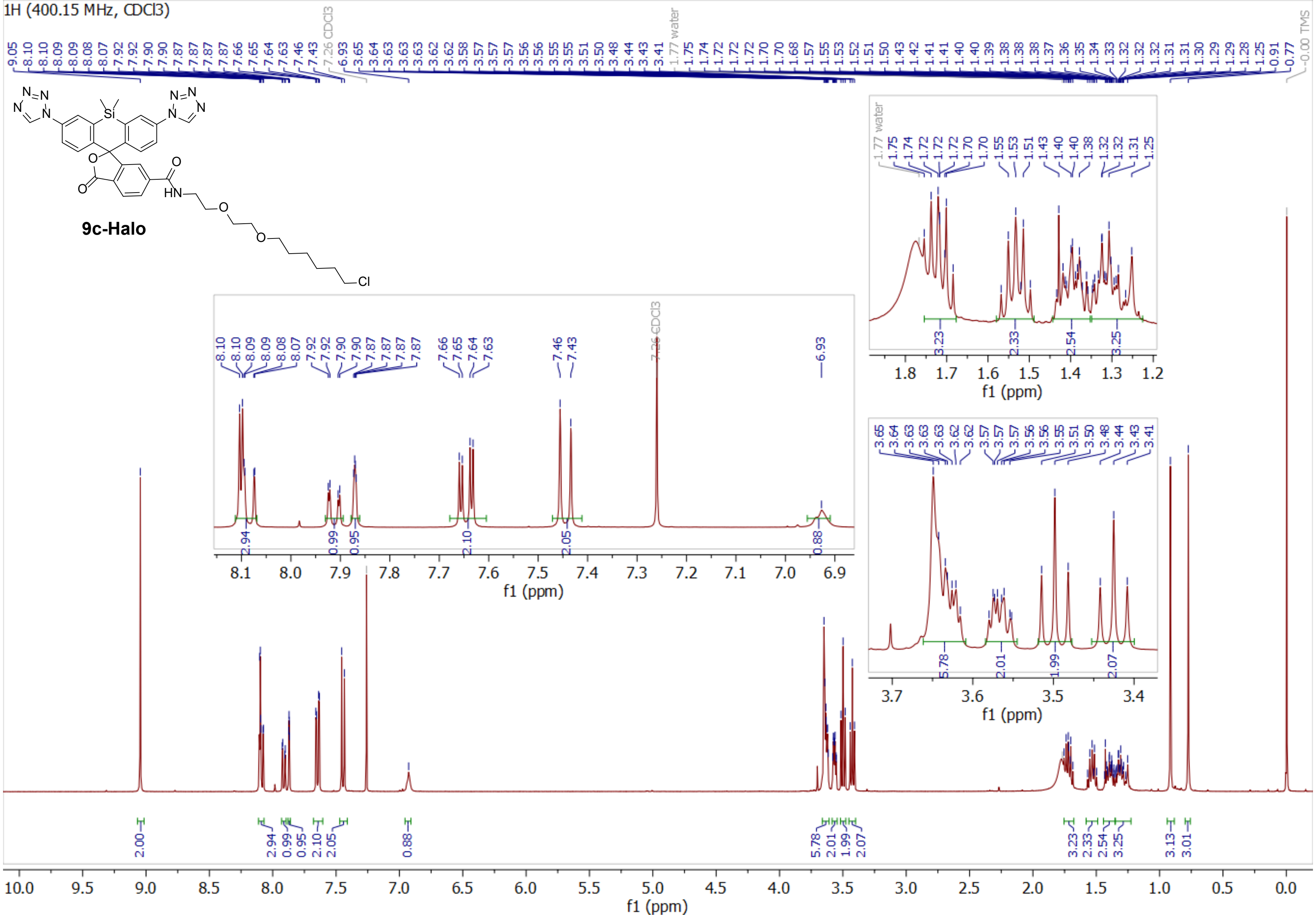

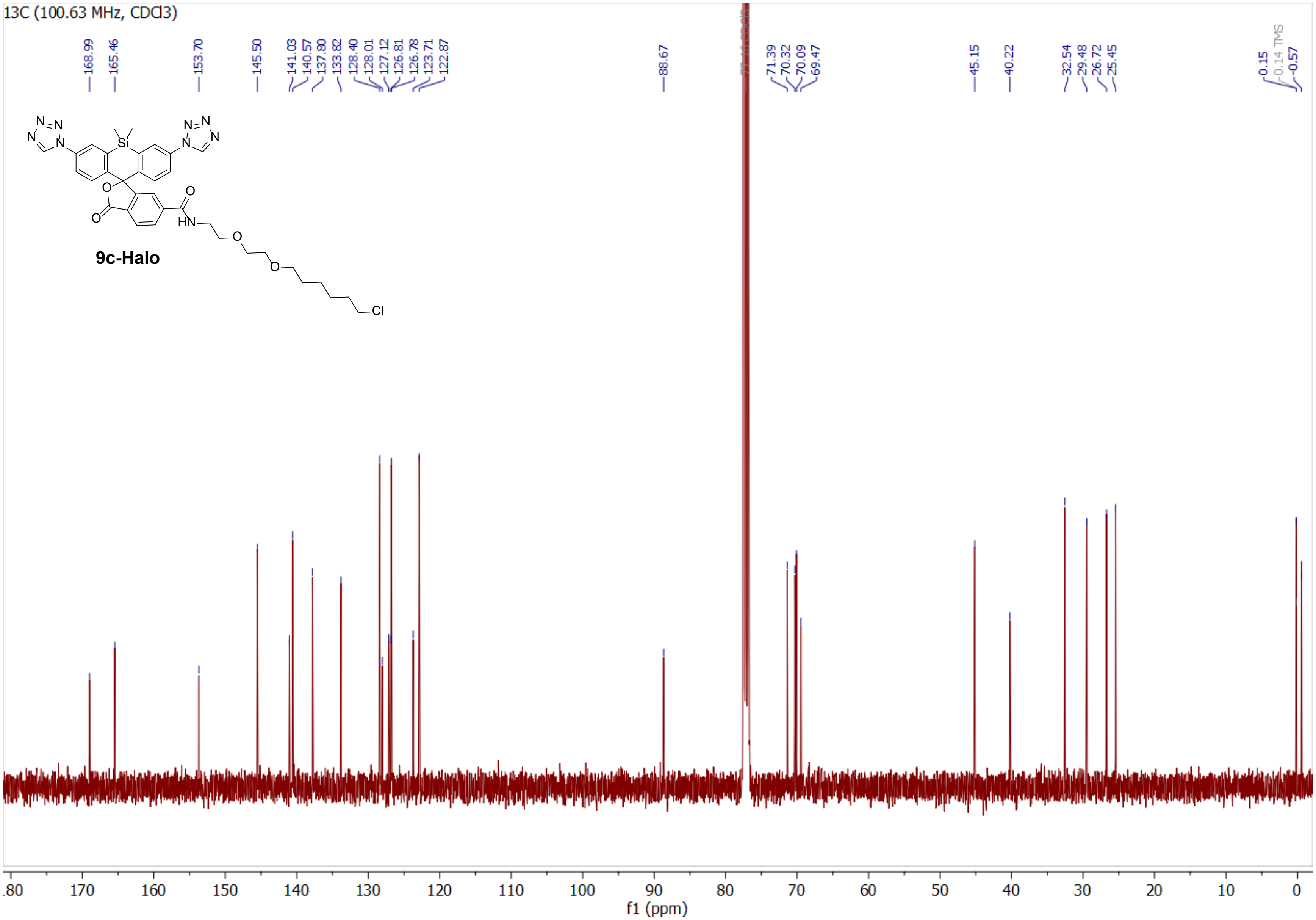

13C (100.63 MHz, CDCl3)
168.99
165.46
153.70
145.50
141.03
140.57
137.80
133.82
128.40
128.01
127.12
126.81
126.78
123.71
122.87
88.67
71.39
70.32
70.09
69.47
45.15
40.22
32.54
29.48
26.72
25.45
0.15
-0.14 TMS
-0.57
9c-Halo
f1 (ppm)

CR1

1H (400.15 MHz, DMSO)

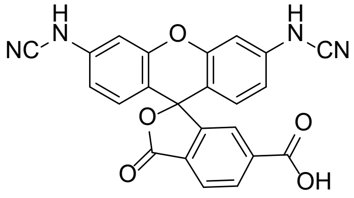

CR1

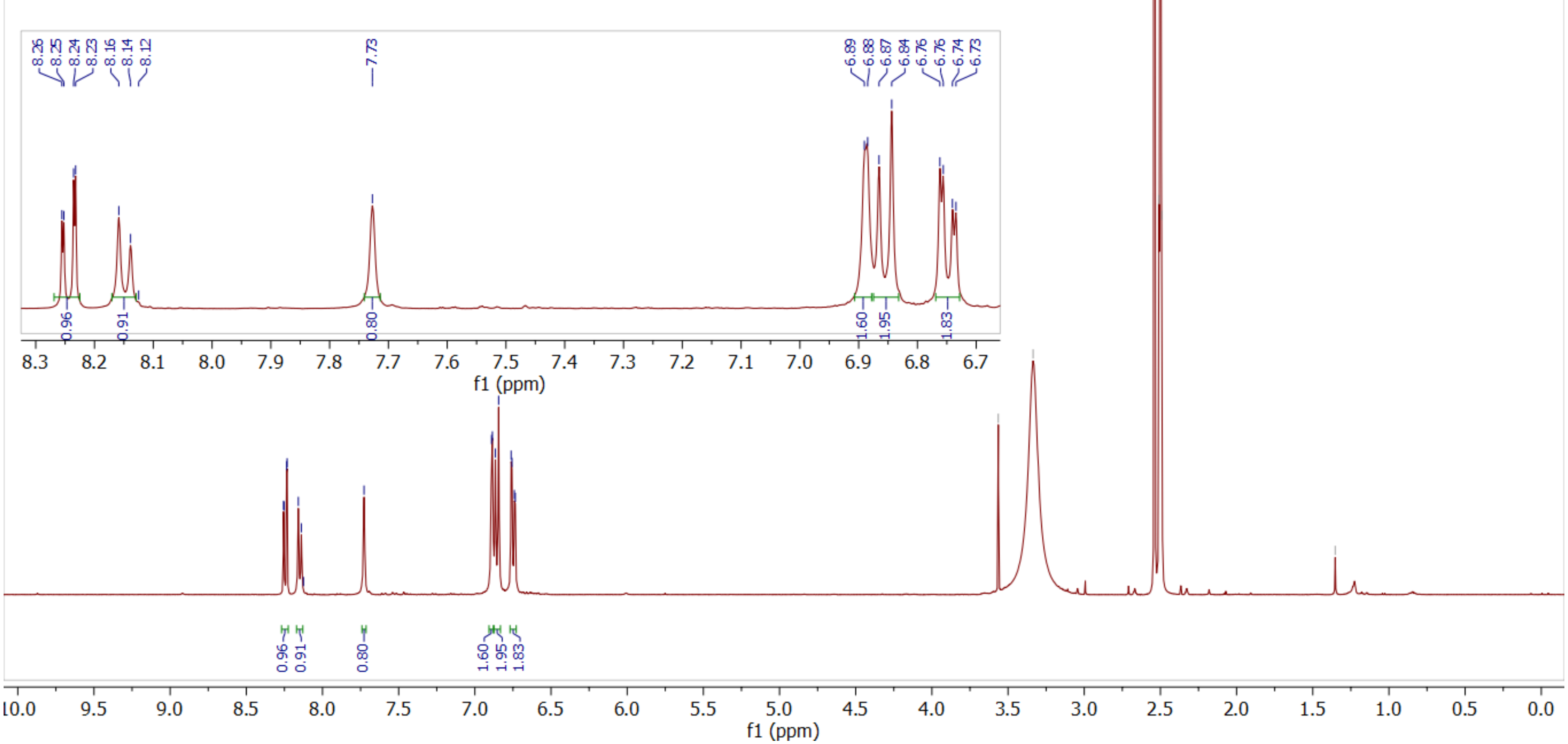

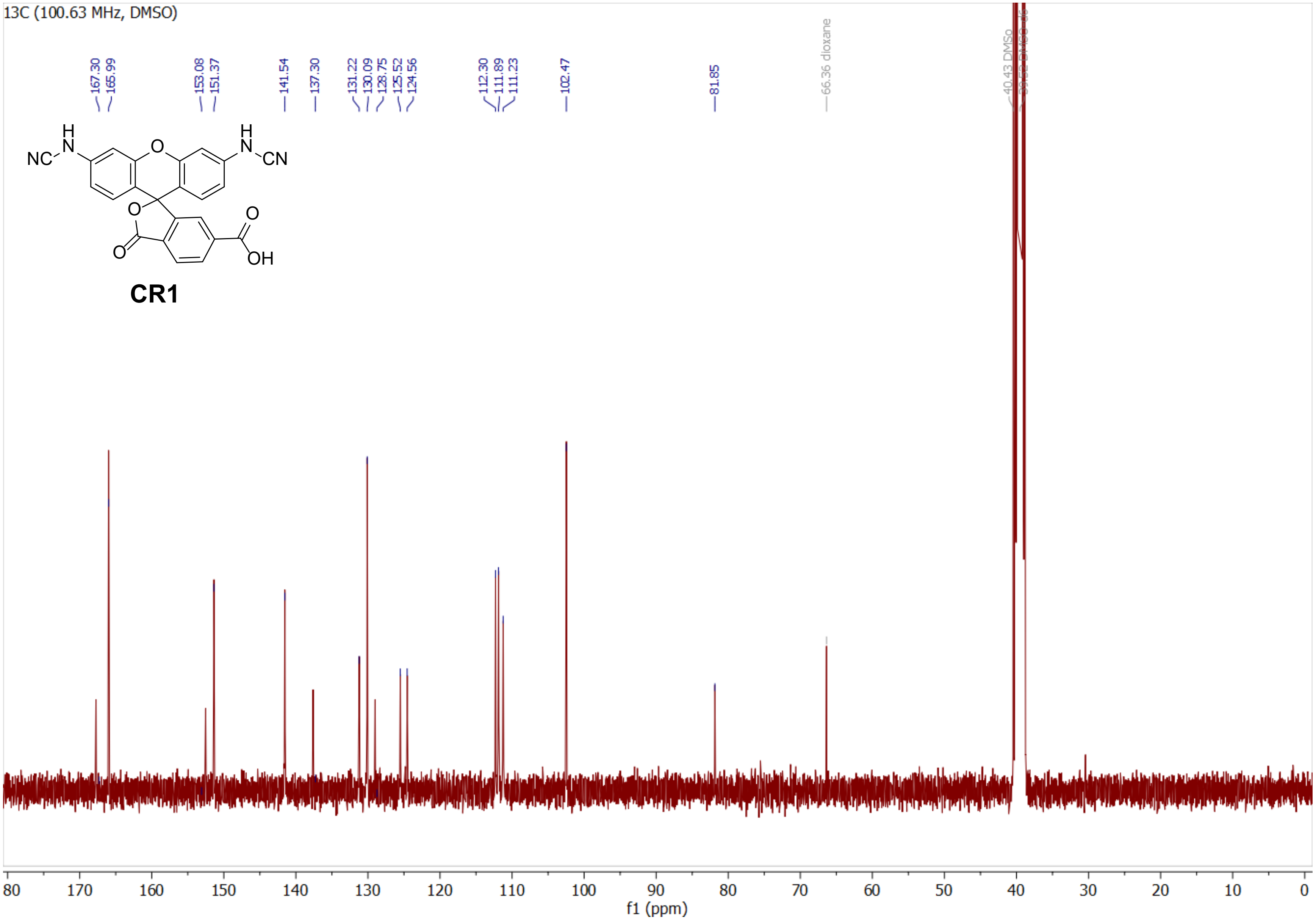

13C (100.63 MHz, DMSO)
167.30
165.99
153.08
151.37
141.54
137.30
131.22
130.09
128.75
125.52
124.56
112.30
111.89
111.23
102.47
81.85
66.36 dioxane
40.43 DMSo
NC
N
H
O
CN
O
O
O
OH
CR1
f1 (ppm)

CR2

1H (400.15 MHz, DMSO)

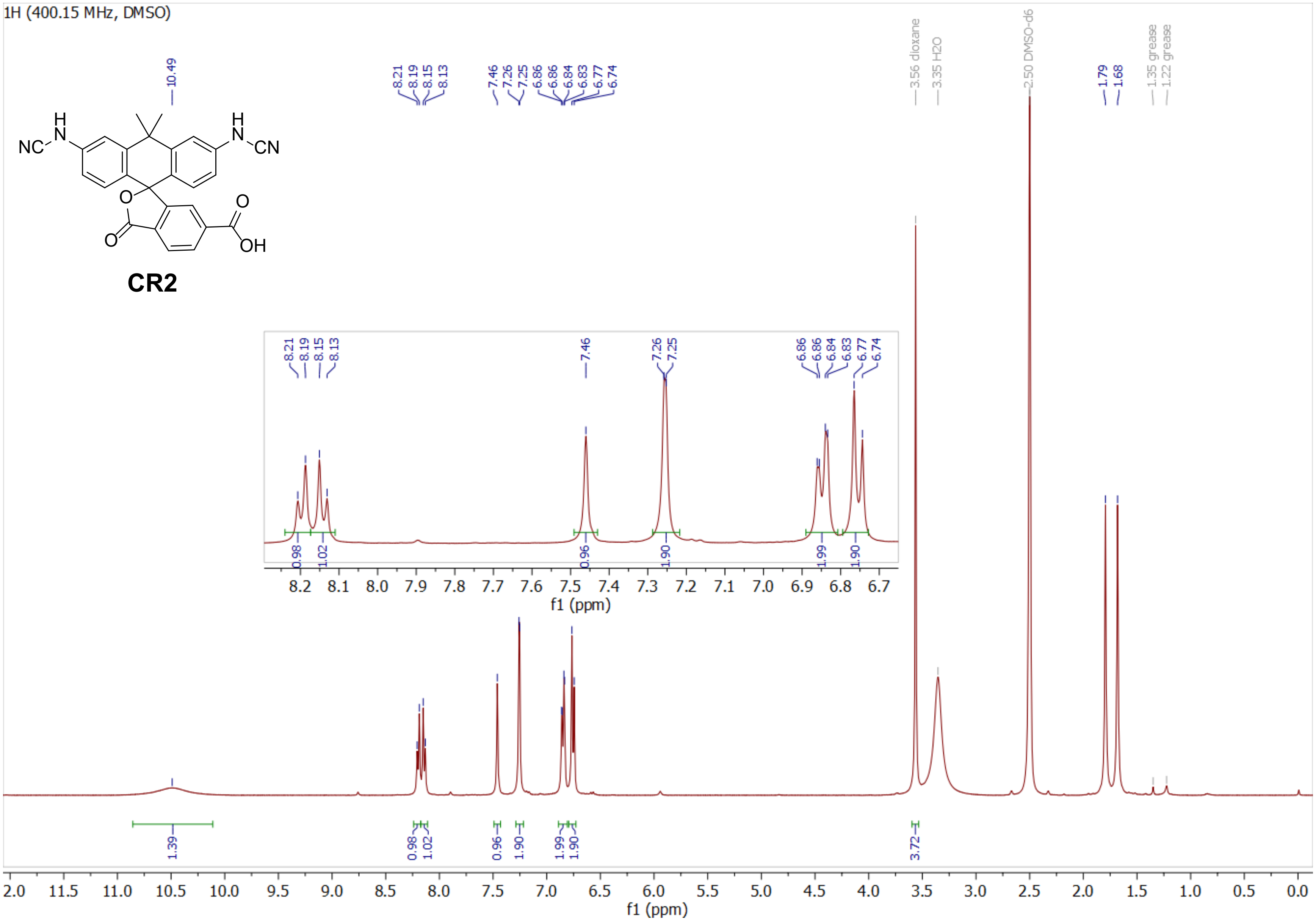

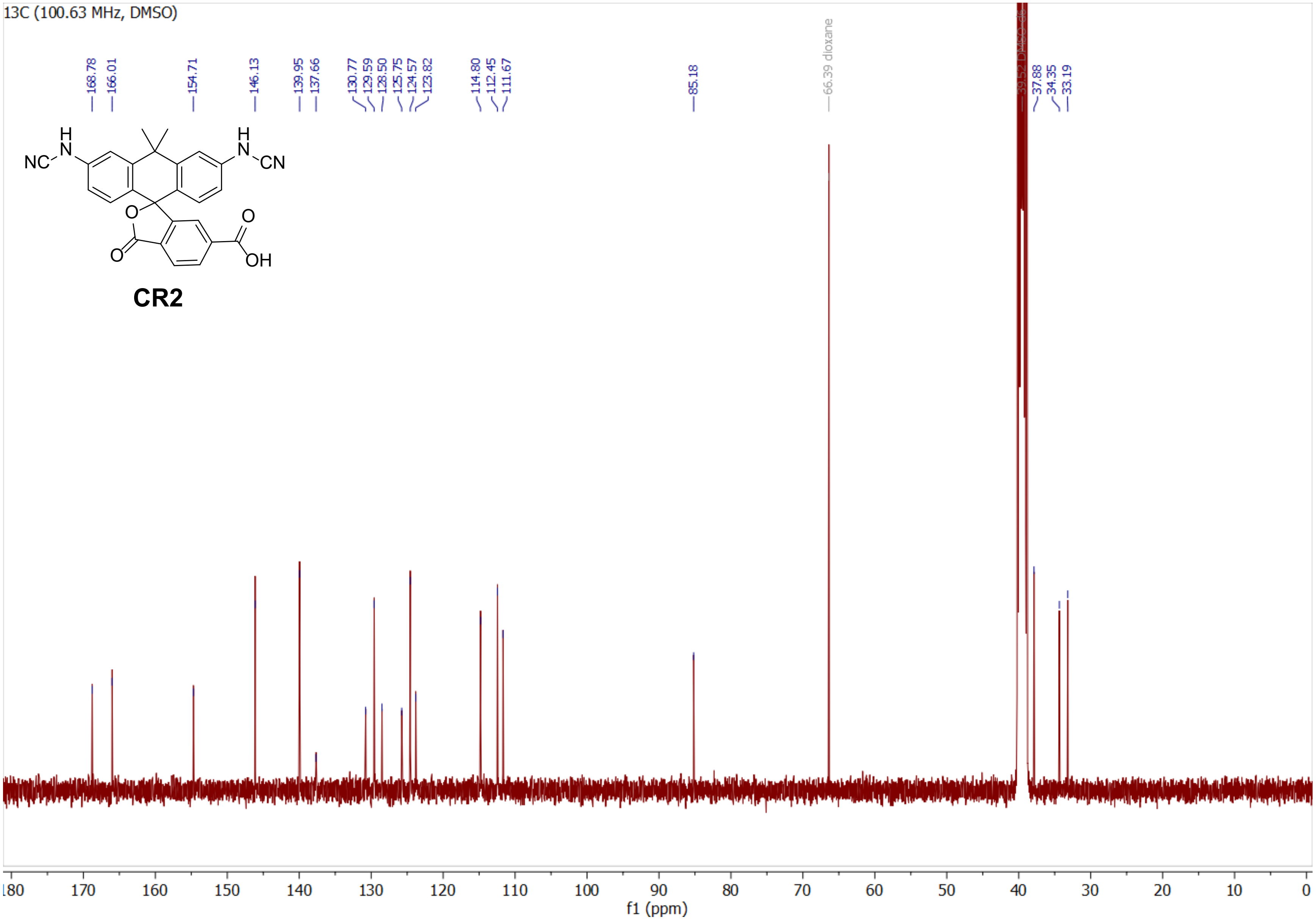
13C (100.63 MHz, DMSO)
168.78
166.01
154.71
146.13
139.95
137.66
130.77
129.59
128.50
125.75
124.57
123.82
114.80
112.45
111.67
85.18
66.39 dioxane
39.52 DMSO-d6
37.88
34.35
33.19
CR2
180
170
160
150
140
130
120
110
100
90
80
70
60
50
40
30
20
10
0
f1 (ppm)

CR3

1H (400.15 MHz, DMSO)

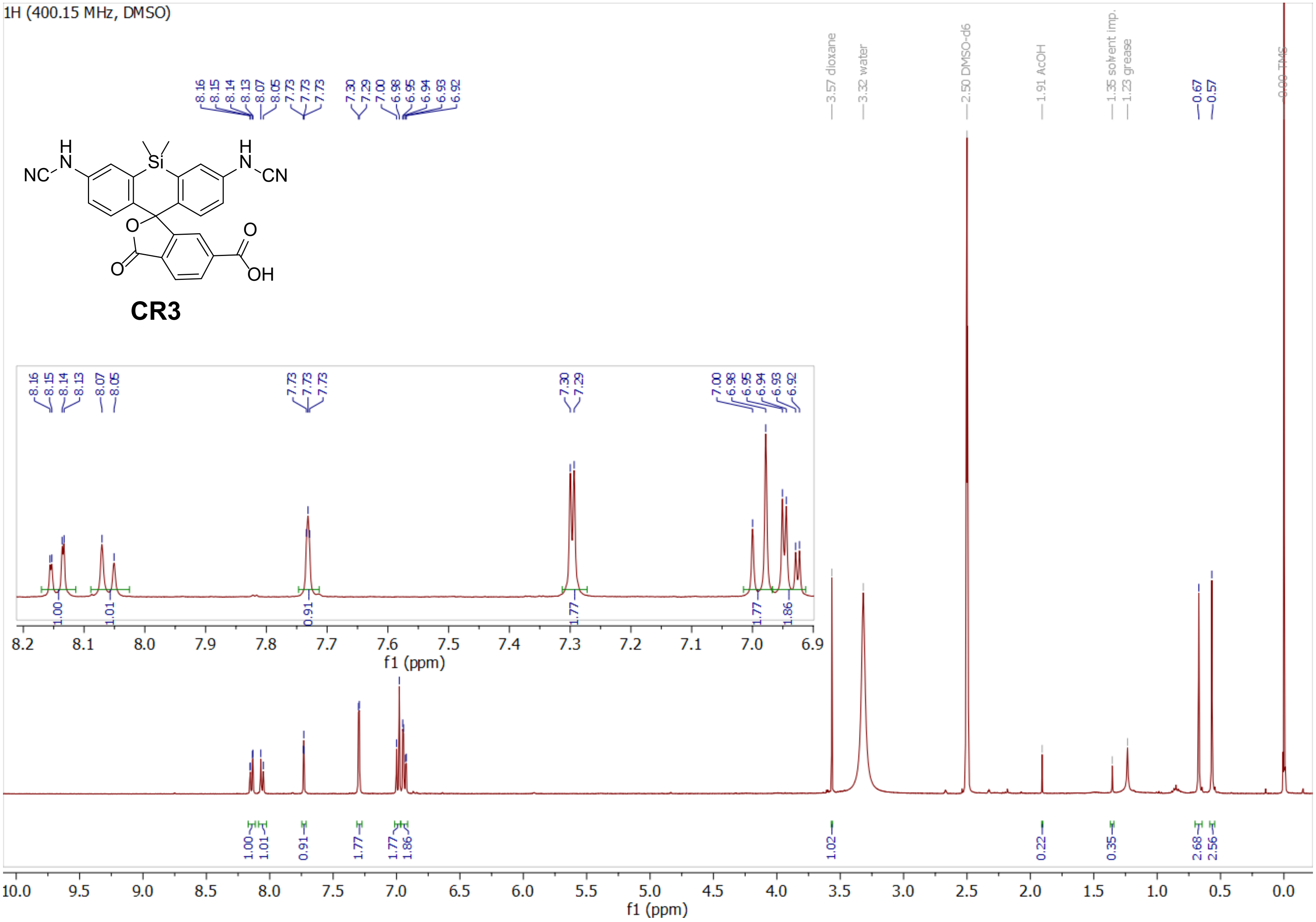

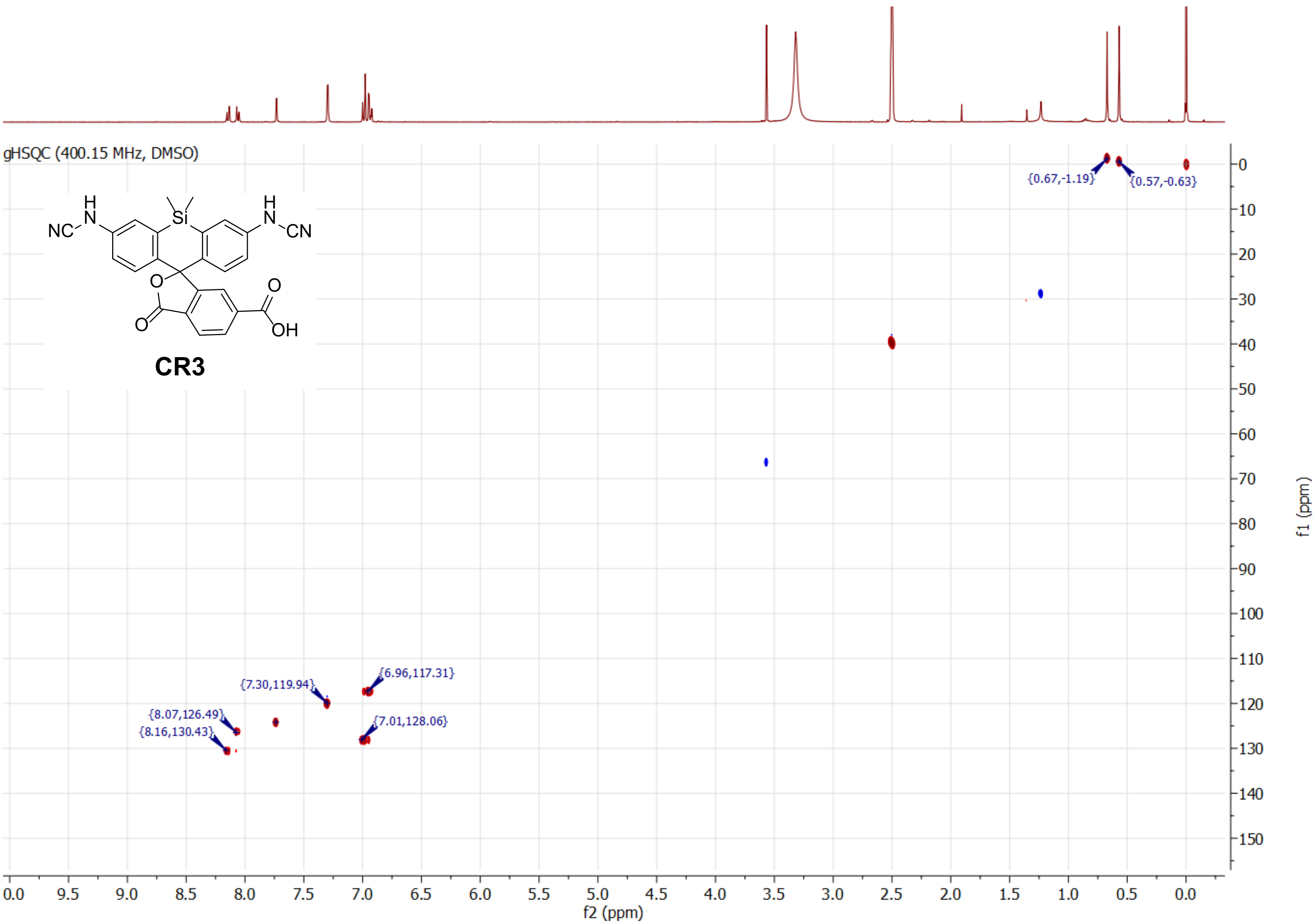
gHSQC (400.15 MHz, DMSO)
NC
N
H
Si
N
H
CN
O
O
O
OH
CR3
{0.67,-1.19}
{0.57,-0.63}
{7.30,119.94}
{6.96,117.31}
{8.07,126.49}
{8.16,130.43}
{7.01,128.06}
f2 (ppm)
f1 (ppm)

CR1-Halo

1H (400.15 MHz, DMSO)

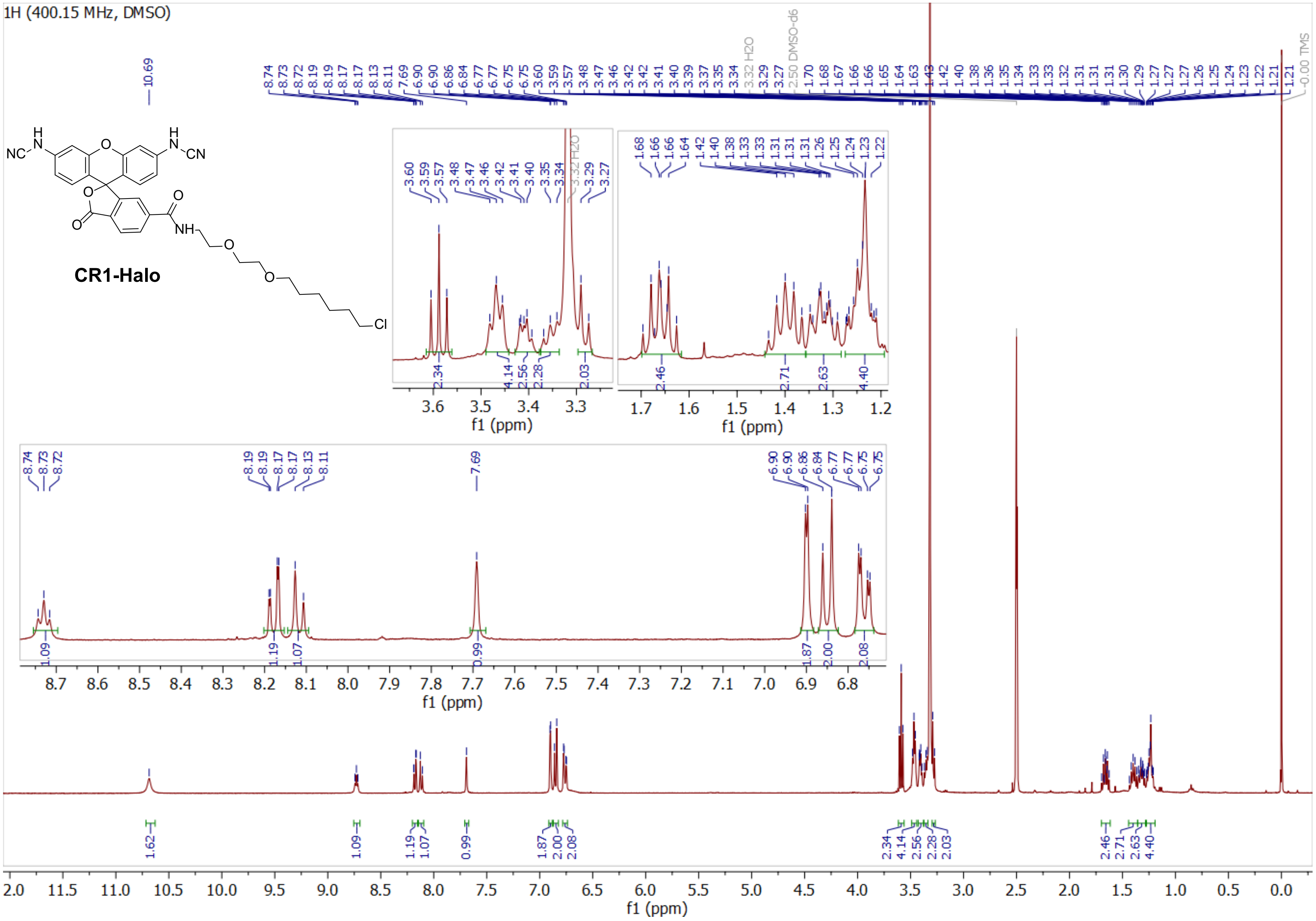

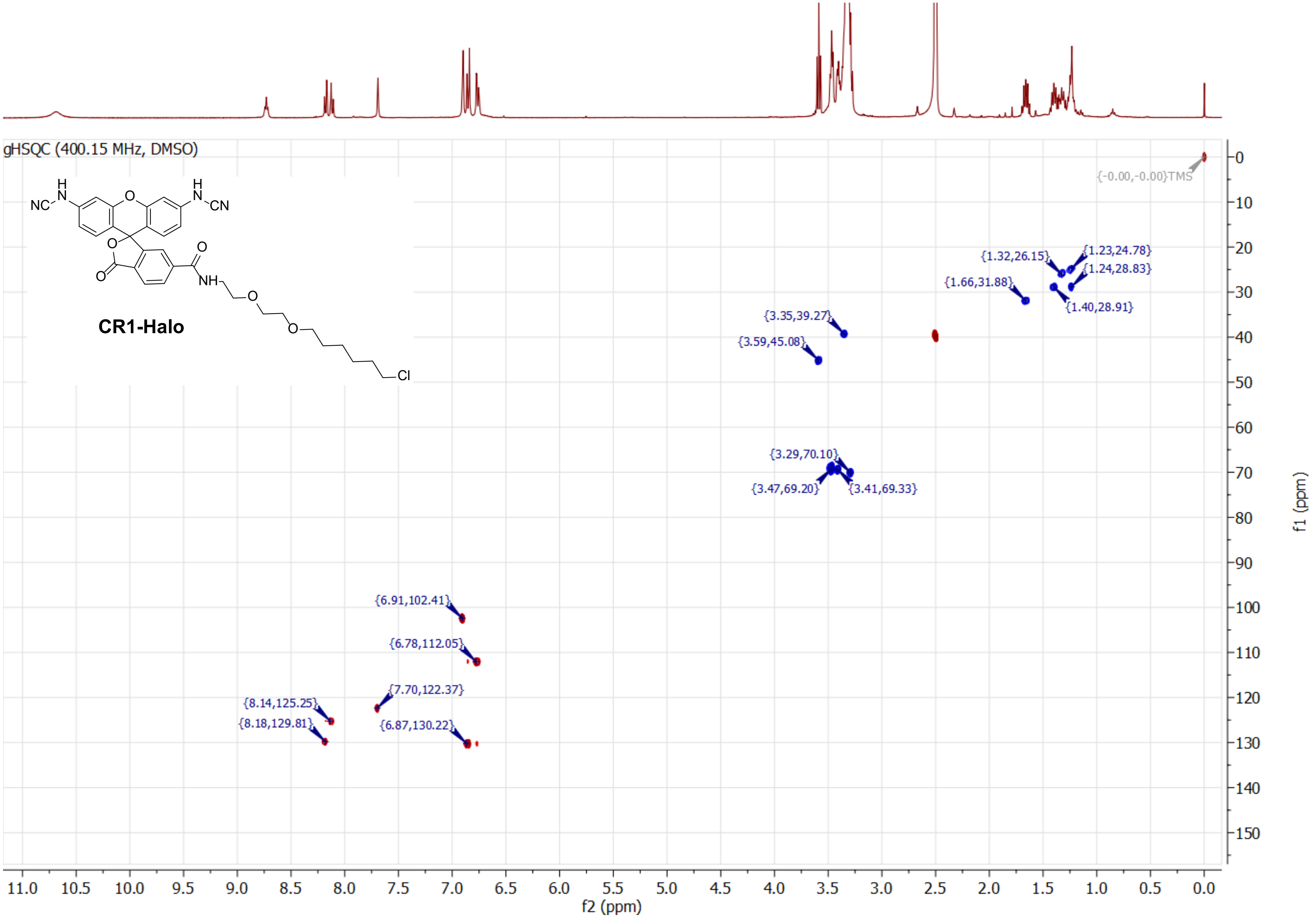
gHSQC (400.15 MHz, DMSO)
CR1-Halo
{-0.00,-0.00}TMS
{1.23,24.78}
{1.32,26.15}
{1.24,28.83}
{1.66,31.88}
{1.40,28.91}
{3.35,39.27}
{3.59,45.08}
{3.29,70.10}
{3.47,69.20}
{3.41,69.33}
{6.91,102.41}
{6.78,112.05}
{7.70,122.37}
{8.14,125.25}
{8.18,129.81}
{6.87,130.22}
f2 (ppm)
f1 (ppm)

CR1-BG

1H (400.15 MHz, DMSO)

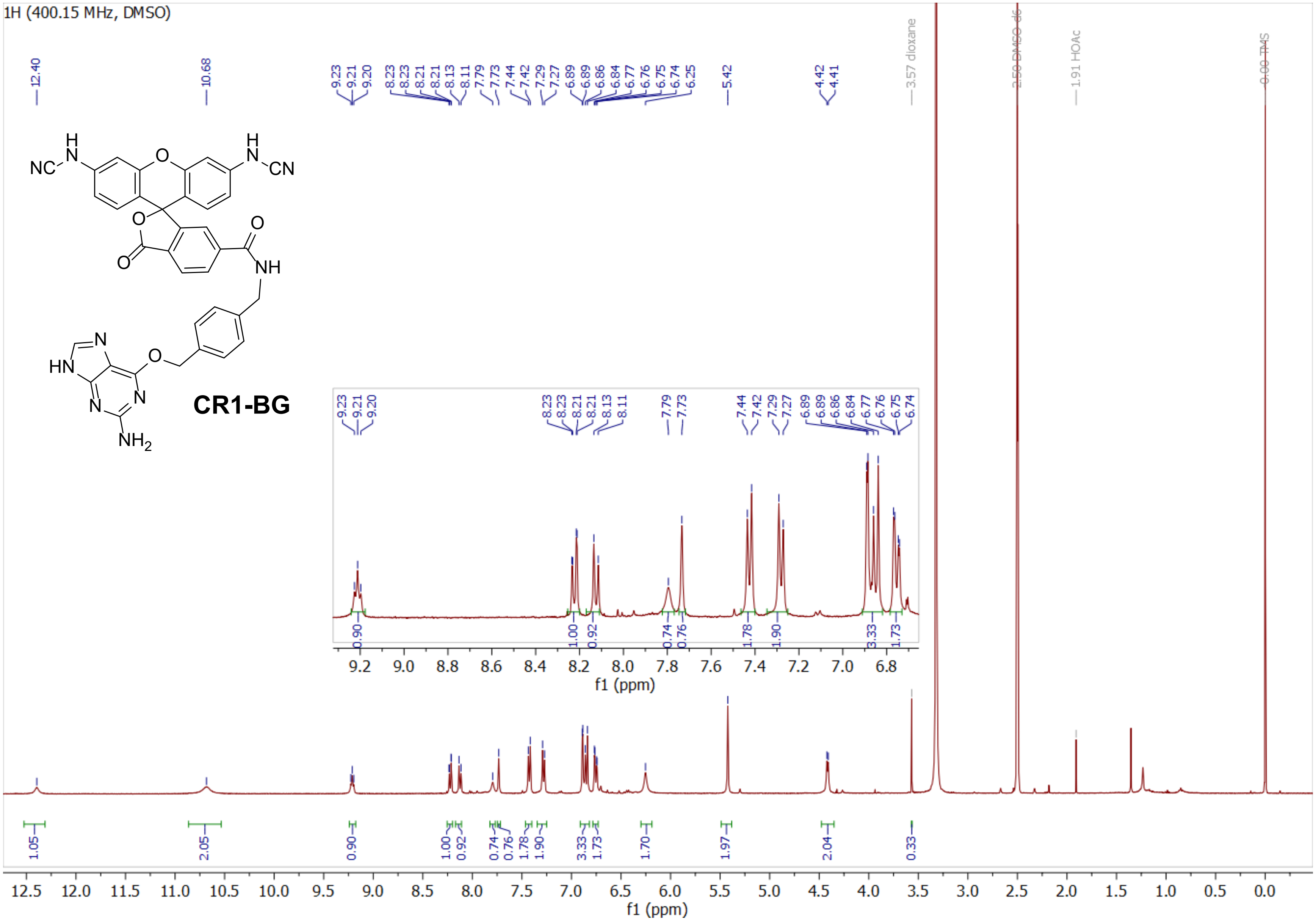

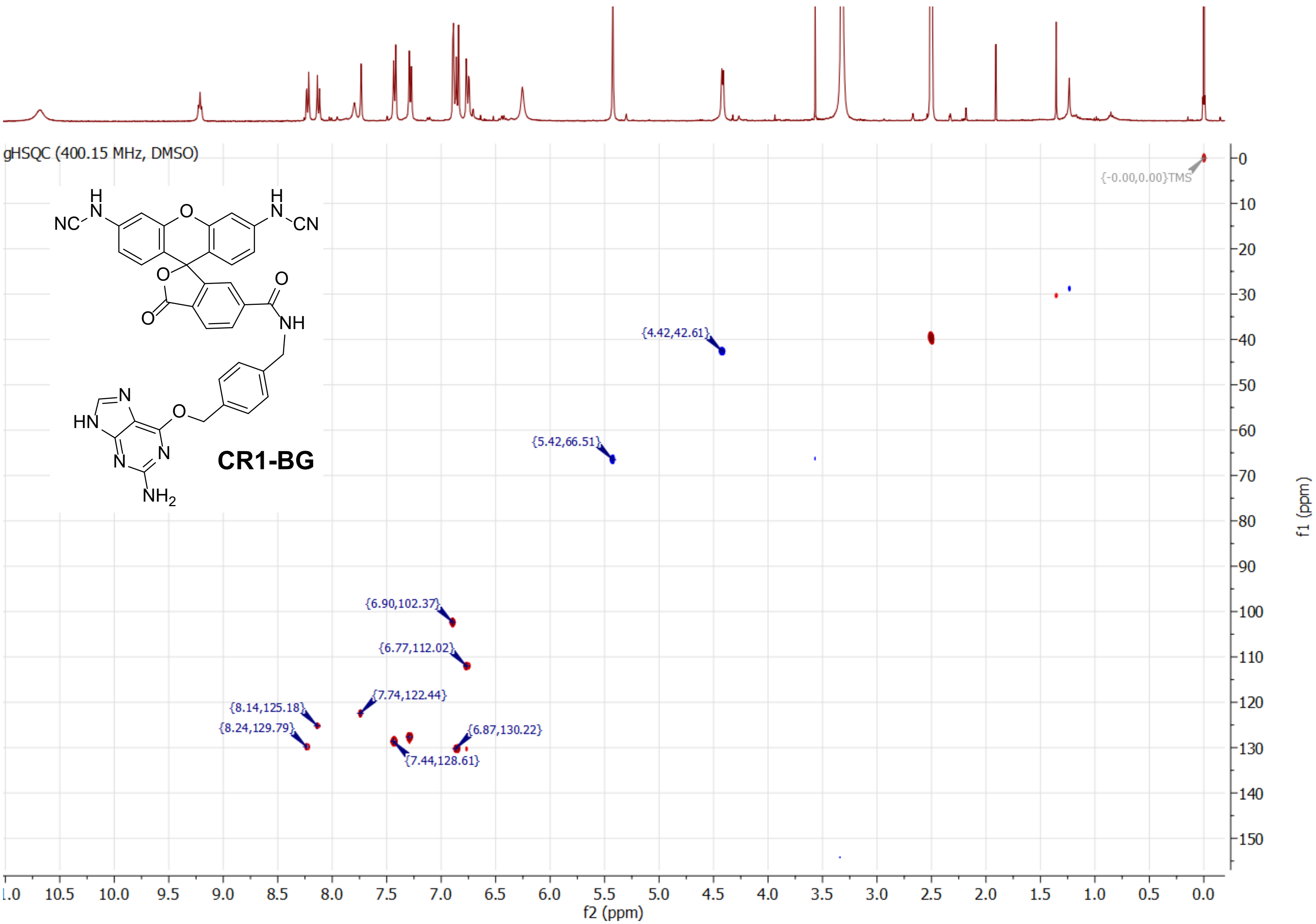
gHSQC (400.15 MHz, DMSO)
CR1-BG
{-0.00,0.00}TMS
{4.42,42.61}
{5.42,66.51}
{6.90,102.37}
{6.77,112.02}
{7.74,122.44}
{8.14,125.18}
{8.24,129.79}
{6.87,130.22}
{7.44,128.61}
f2 (ppm)
f1 (ppm)

CR2-Halo

1H (400.15 MHz, DMSO)

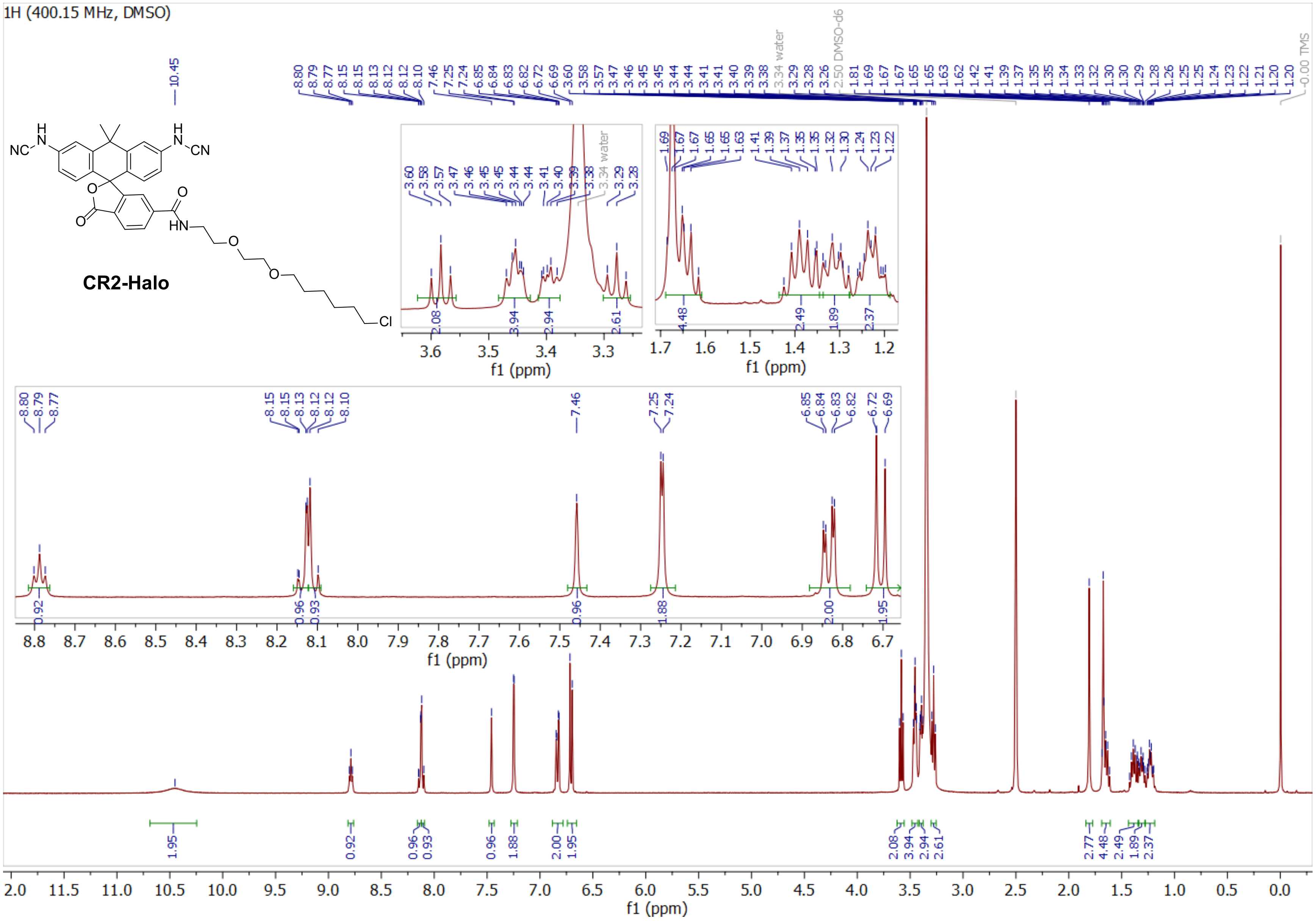

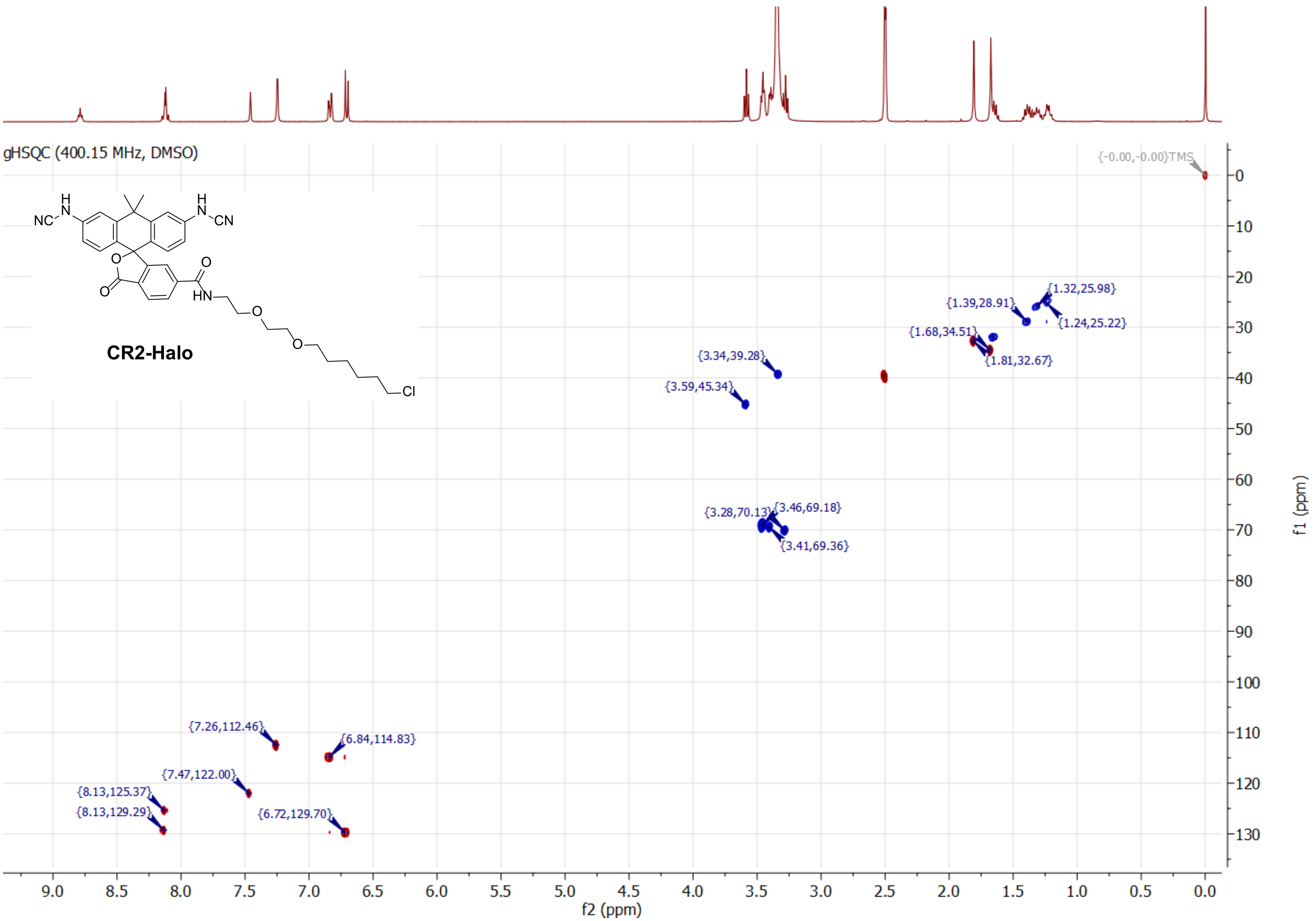

gHSQC (400.15 MHz, DMSO)
CR2-Halo
{-0.00,-0.00}TMS
{1.32,25.98}
{1.39,28.91}
{1.24,25.22}
{1.68,34.51}
{1.81,32.67}
{3.34,39.28}
{3.59,45.34}
{3.28,70.13}
{3.46,69.18}
{3.41,69.36}
{7.26,112.46}
{6.84,114.83}
{7.47,122.00}
{8.13,125.37}
{8.13,129.29}
{6.72,129.70}
f2 (ppm)
f1 (ppm)

CR3-Halo

1H (400.15 MHz, DMSO)

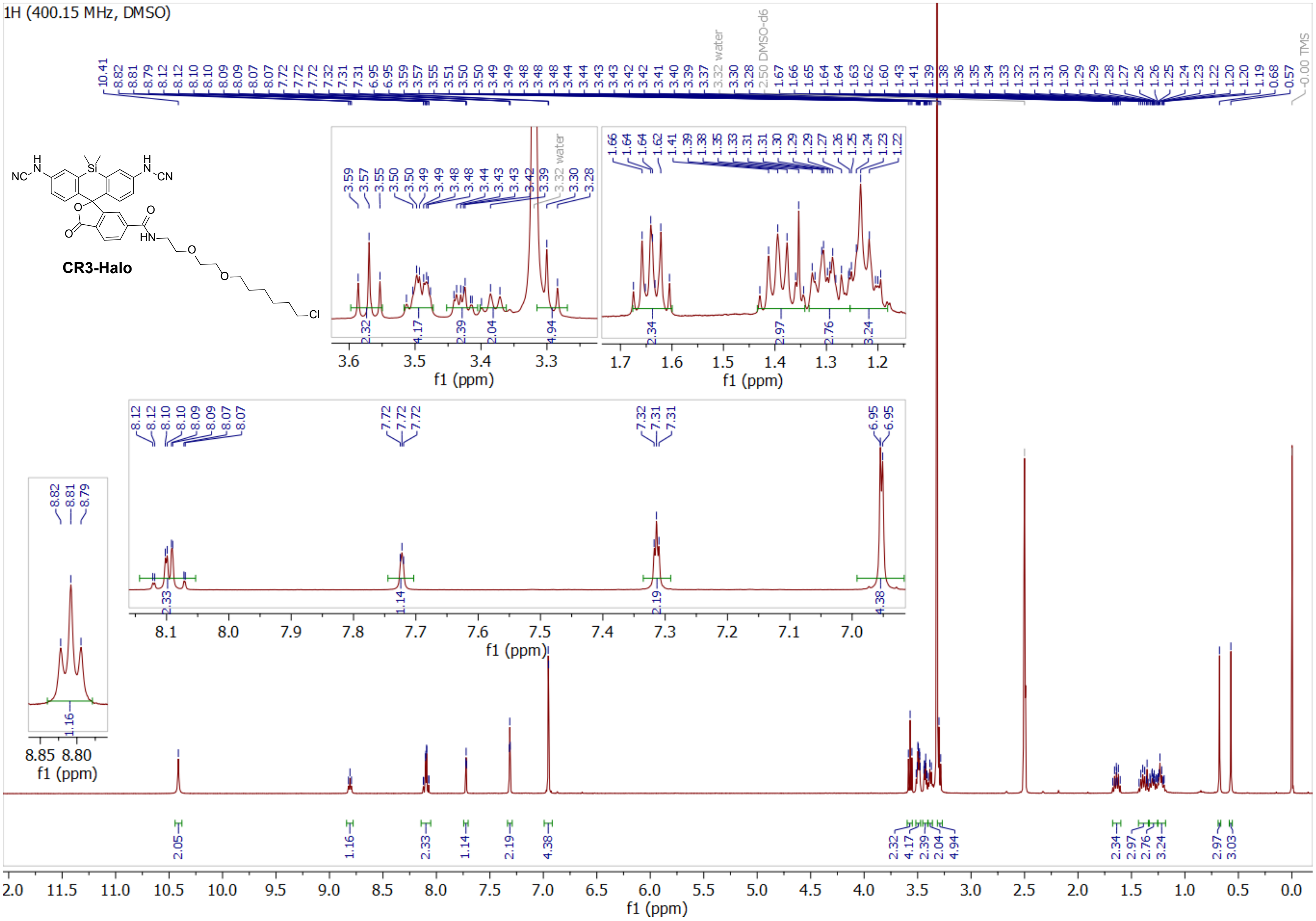

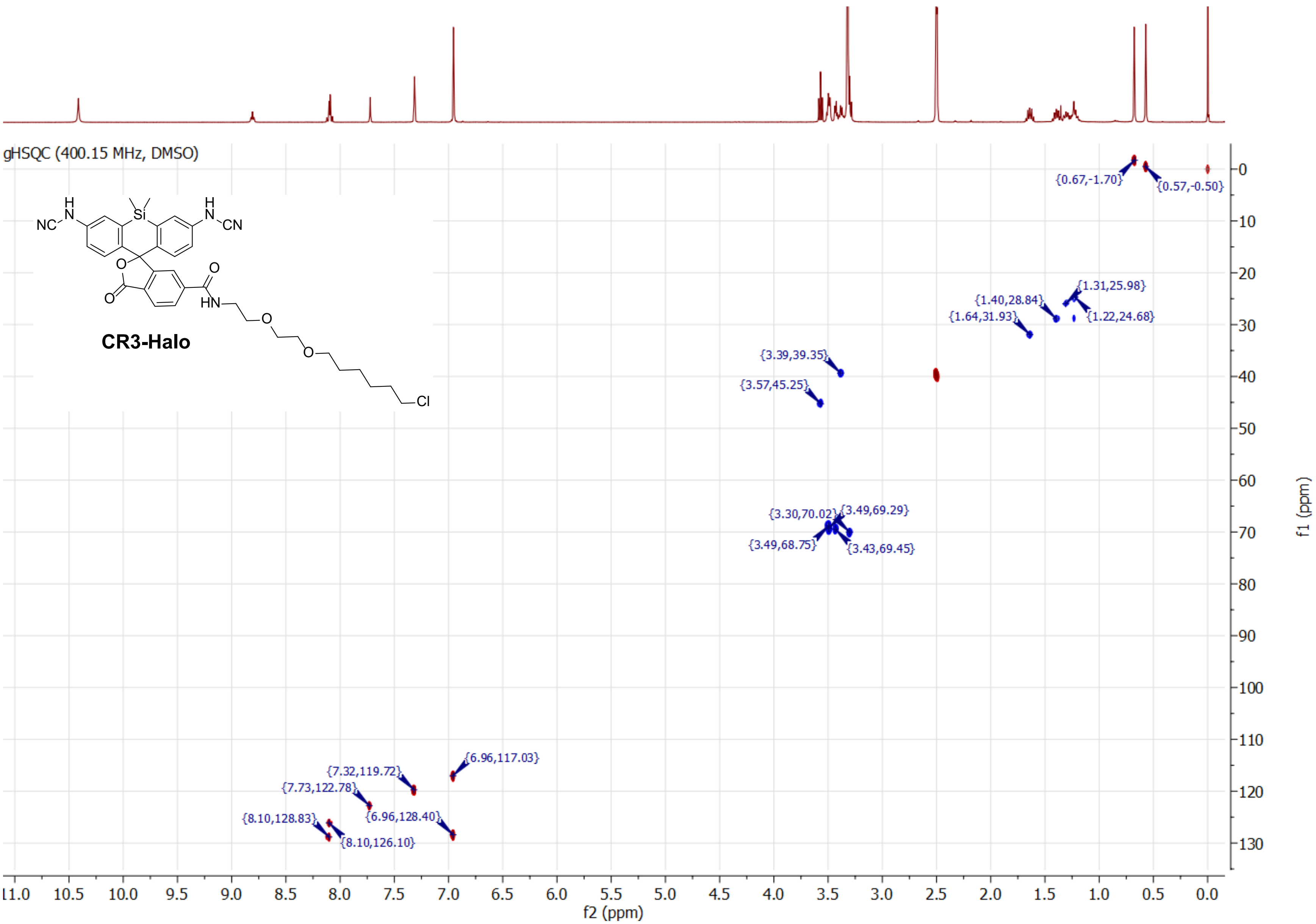

gHSQC (400.15 MHz, DMSO)
CR3-Halo
{0.67,-1.70}
{0.57,-0.50}
{1.31,25.98}
{1.40,28.84}
{1.64,31.93}
{1.22,24.68}
{3.39,39.35}
{3.57,45.25}
{3.30,70.02}
{3.49,69.29}
{3.49,68.75}
{3.43,69.45}
{6.96,117.03}
{7.32,119.72}
{7.73,122.78}
{6.96,128.40}
{8.10,128.83}
{8.10,126.10}
f2 (ppm)
f1 (ppm)
